# A phase III randomized crossover trial of plerixafor versus G-CSF for treatment of WHIM syndrome

**DOI:** 10.1172/JCI164918

**Published:** 2023-10-02

**Authors:** David H. McDermott, Daniel Velez, Elena Cho, Edward W. Cowen, John J. DiGiovanna, Diana V. Pastrana, Christopher B. Buck, Katherine R. Calvo, Pamela J. Gardner, Sergio D. Rosenzweig, Pamela Stratton, Melissa A. Merideth, H. Jeffrey Kim, Carmen Brewer, James D. Katz, Douglas B. Kuhns, Harry L. Malech, Dean Follmann, Michael P. Fay, Philip M. Murphy

**Affiliations:** 1 Laboratory of Molecular Immunology, National Institute of Allergy and Infectious Diseases,; 2 Dermatology Branch, National Institute of Arthritis and Musculoskeletal and Skin Diseases,; 3 Laboratory of Cancer Biology and Genetics and; 4 Laboratory of Cellular Oncology, National Cancer Institute,; 5 Department of Laboratory Medicine, Clinical Center,; 6 Office of the Clinical Director, National Institute of Dental and Craniofacial Research,; 7 National Institute of Neurologic Disorders and Stroke,; 8 Office of the Clinical Director, National Human Genome Research Institute,; 9 Otolaryngology Branch, National Institute on Deafness and other Communication Disorders, and; 10 Rheumatology Fellowship and Training Branch, National Institute of Arthritis and Musculoskeletal and Skin Diseases, NIH, Bethesda, Maryland, USA.; 11 Leidos Biomedical Research Inc., Frederick, Maryland, USA.; 12 Laboratory of Clinical Immunology and Microbiology and; 13 Biostatistics Research Branch, National Institute of Allergy and Infectious Diseases, NIH, Bethesda, Maryland, USA.

**Keywords:** Clinical Trials, Immunology, Genetic diseases, Innate immunity, Neutrophils

## Abstract

**BACKGROUND:**

Warts, hypogammaglobulinemia, infections, and myelokathexis (WHIM) syndrome is a primary immunodeficiency disorder caused by heterozygous gain-of-function CXCR4 mutations. Myelokathexis is a kind of neutropenia caused by neutrophil retention in bone marrow and in WHIM syndrome is associated with lymphopenia and monocytopenia. The CXCR4 antagonist plerixafor mobilizes leukocytes to the blood; however, its safety and efficacy in WHIM syndrome are undefined.

**METHODS:**

In this investigator-initiated, single-center, quadruple-masked phase III crossover trial, we compared the total infection severity score (TISS) as the primary endpoint in an intent-to-treat manner in 19 patients with WHIM who each received 12 months treatment with plerixafor and 12 months treatment with granulocyte CSF (G-CSF, the standard of care for severe congenital neutropenia). The treatment order was randomized for each patient.

**RESULTS:**

Plerixafor was nonsuperior to G-CSF for TISS (*P* = 0.54). In exploratory endpoints, plerixafor was noninferior to G-CSF for maintaining neutrophil counts of more than 500 cells/μL (*P* = 0.023) and was superior to G-CSF for maintaining lymphocyte counts above 1,000 cells/μL (*P* < 0.0001). Complete regression of a subset of large wart areas occurred on plerixafor in 5 of 7 patients with major wart burdens at baseline. Transient rash occurred on plerixafor, and bone pain was more common on G-CSF. There were no significant differences in drug preference or quality of life or the incidence of drug failure or serious adverse events.

**CONCLUSION:**

Plerixafor was not superior to G-CSF in patients with WHIM for TISS, the primary endpoint. Together with wart regression and hematologic improvement, the infection severity results support continued study of plerixafor as a potential treatment for WHIM syndrome.

**TRIAL REGISTRATION:**

Clinicaltrials.gov NCT02231879.

**FUNDING:**

This study was funded by the Division of Intramural Research, National Institute of Allergy and Infectious Diseases.

## Introduction

Warts, hypogammaglobulinemia, infections, and myelokathexis (WHIM) syndrome presents in childhood, with severe congenital neutropenia (SCN), lymphopenia, hypogammaglobulinemia, recurrent otosinopulmonary and skin infections, and warts. Warts and hypogammaglobulinemia are incompletely penetrant, and additional uncommon phenotypes continue to be discovered (1–8). In almost all cases the cause is autosomal dominant gain-of-function truncating mutation of the carboxy-terminal domain of CXCR4, a G protein–coupled leukocyte chemotactic receptor specific for the homeostatic chemokine agonist CXCL12. Two mutations, R334X and S338X, account for approximately 70% of cases (4, 9). CXCR4 normally promotes homing of circulating senescent neutrophils to bone marrow and inhibits egress of nascent bone marrow neutrophils to blood (10–13). WHIM mutations exaggerate both activities, causing neutropenia despite myeloid hyperplasia, two main features of myelokathexis.

Current treatments include granulocyte CSF (G-CSF), the standard of care for SCN (14), and/or supplemental immunoglobulin; however, breakthrough infections occur and warts persist. Moreover, G-CSF commonly causes bone pain, which affects compliance. G-CSF partly works by releasing neutrophil elastase, which inactivates CXCL12, and does not significantly affect blood levels of mature leukocytes other than neutrophils (15). Plerixafor (AMD3100, Mozobil; Sanofi-Genzyme) is a CXCR4 antagonist that rapidly, transiently, and nonselectively increases levels of most circulating leukocytes in both healthy individuals and patients with WHIM syndrome (16–20). It is FDA approved in combination with G-CSF to mobilize hematopoietic stem cells (HSCs) for autologous transplantation in patients with multiple myeloma and non-Hodgkin’s lymphoma (21). In a phase I trial, plerixafor was well tolerated and durably reversed panleukopenia in 5 patients with WHIM (19, 20). Infection frequency was low, and some warts regressed. We have now tested the hypothesis that plerixafor is superior to G-CSF for control of infection severity in WHIM syndrome.

## Results

### Patient characteristics and treatments.

**Figure 1:**
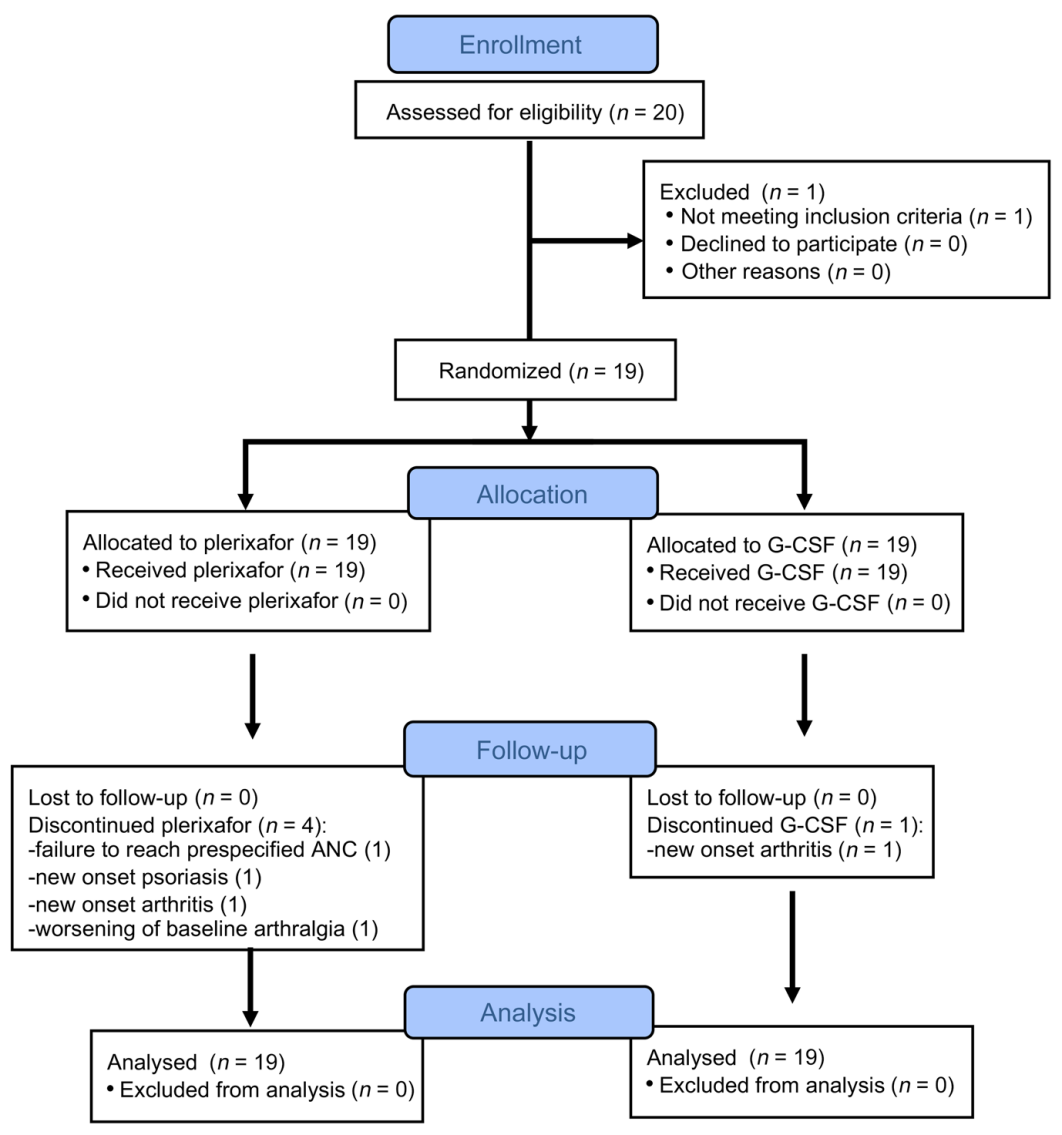
Flow diagram of the progress of the participants through the phases of the study. The diagram was created using CONSORT (http://www.consort-statement.org/consort-statement/flow-diagram).

We enrolled 20 patients with WHIM at the NIH Clinical Center (NIH-CC) from October 14, 2014, through November 6, 2017 (Figure 1, Table 1, and Supplemental Table 1; supplemental material available online with this article; https://doi.org/10.1172/JCI164918DS1). One patient was disqualified during screening and before randomization because of G-CSF intolerance from bone pain. The 19 randomized participants included 11 female and 3 male adults (aged 20–57 years) and 2 female and 3 male children (aged 10–16 years). There were 9 White, 6 Hispanic, 2 African American, and 2 White/Native American individuals. *CXCR4* mutations included R334X (*n* = 10), S338X (*n* = 2), E343X (*n* = 2), and 5 unique mutations (Supplemental Table 2). Patients M01–M18 have previously reported *CXCR4* mutations (3, 6, 9, 22). Patient M19 has a potentially novel p.V320fs342X frameshift mutation.

Thirteen patients had all 4 acronymic WHIM phenotypes. Two patients lacked only hypogammaglobulinemia, 2 lacked only warts, and 2 lacked both warts and hypogammaglobulinemia (Table 1). Sixteen patients had end-organ damage from recurrent infection, including 10 patients with bronchiectasis (Supplemental Table 3 and Supplemental Figure 1). Ten patients were employed full-time, and 6 were full-time students. Compared with the general population, physical composite scores calculated from patient responses at the baseline visit to the SF36 version 2 quality of life questionnaire were the “same or better” for 9 patients, “below” for 4 patients, and “well below” for 5 patients (Supplemental Table 4).

At enrollment, 8 of the 15 patients with a history of hypogammaglobulinemia were receiving supplemental immunoglobulin, and 2 patients were receiving prophylactic oral antibiotics; these treatments were continued (Table 1). Seventeen patients had been treated with G-CSF before enrollment, 12 chronically for approximately 1–27 years up to enrollment, and 5 only during infections. Patients M02 and M04 participated in our previous 6-month phase I study of plerixafor (19).

**Figure 2:**
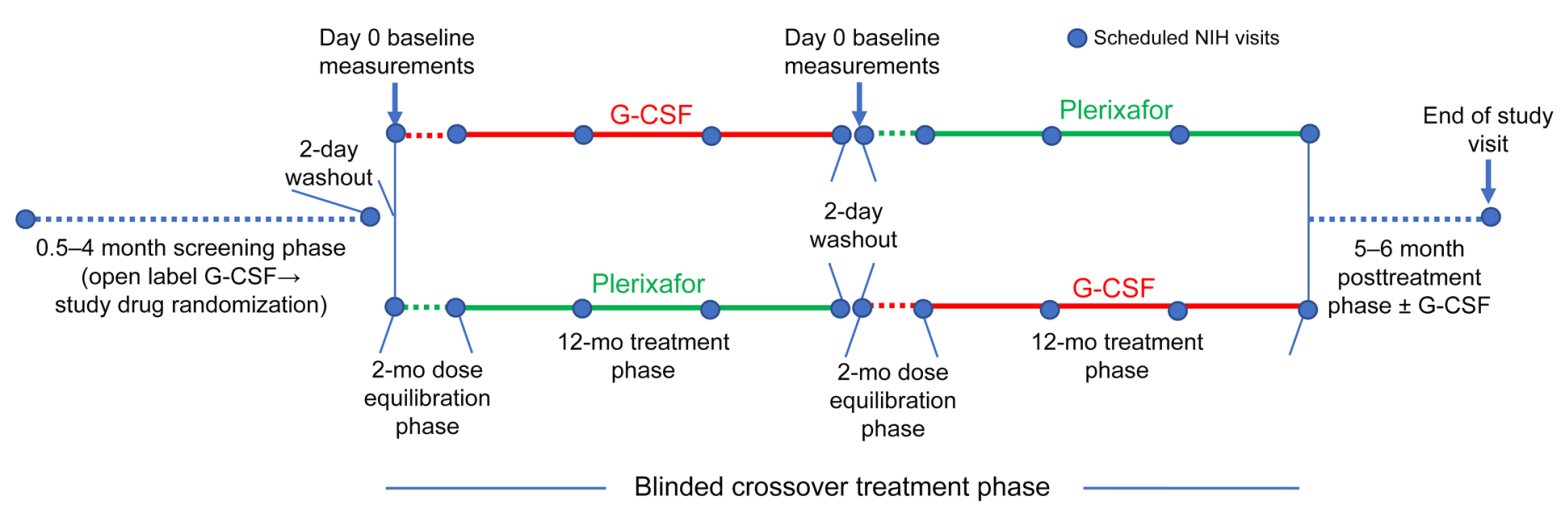
Trial design. Study phase durations, treatment, and interval NIH visits (designated by blue dots) are indicated.

**Figure 3:**
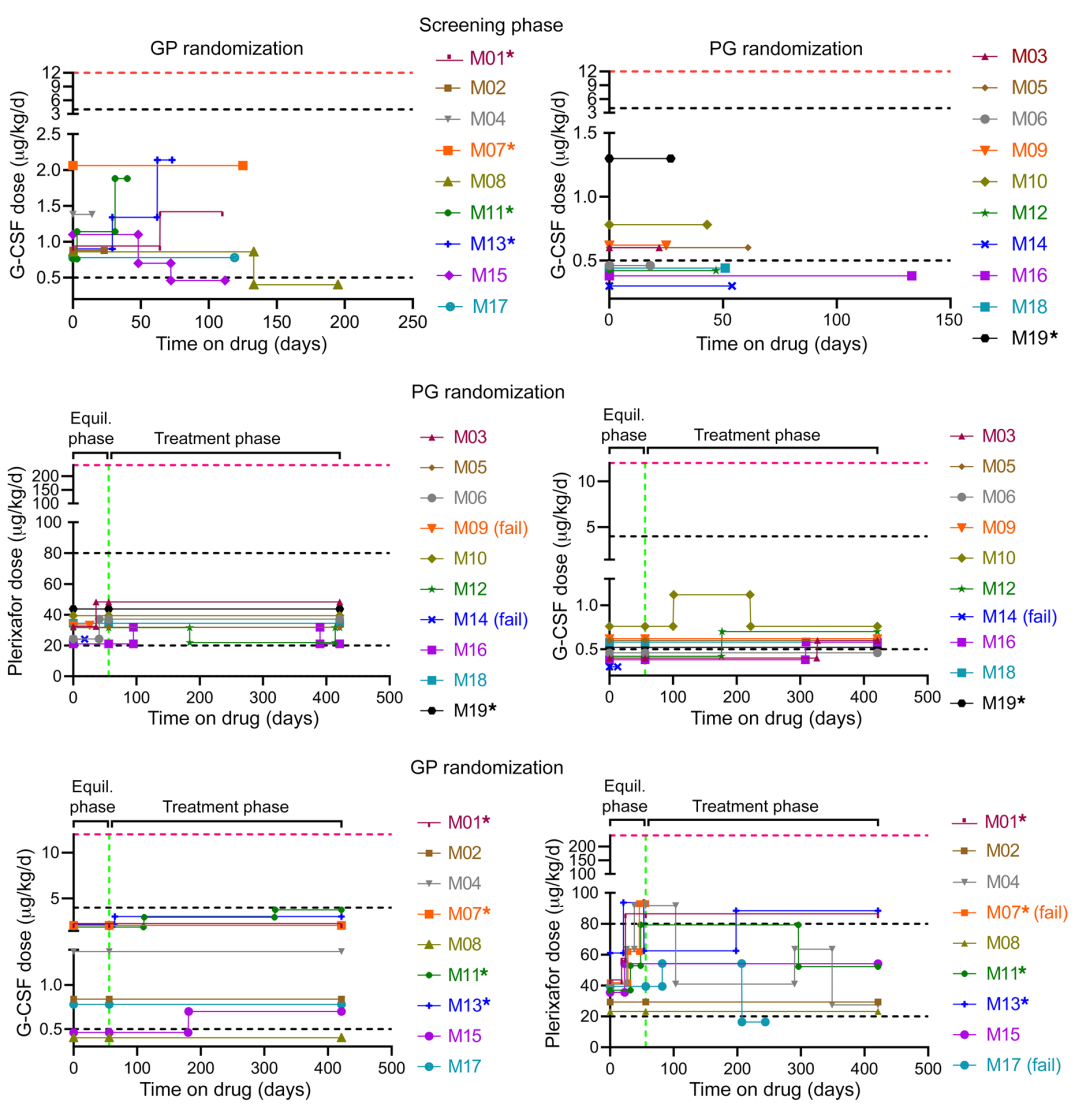
Low dose G-CSF versus plerixafor for 19 patients with WHIM. Drug doses for the 3 phases of the study are shown, stratifying patients by randomization order. P, plerixafor; G, G-CSF. Horizontal dashed red lines indicate the package insert-recommended total daily dosage of G-CSF for severe congenital neutropenia or the single injection daily FDA-approved dose of plerixafor for HSC mobilization. Vertical dashed green lines indicate day 56, the final day of the equilibration phase (Equil. phase). Horizontal dashed black lines indicate target total daily dose ranges for the study. Children are indicated by asterisks. Changes in drug dose were to stay within the target ANC range or to mitigate side effects. (fail), patient dropouts due to side effects or drug failure (see main text for details).

The protocol began with a 0.5- to 4-month prerandomization screening phase (Figure 2) to assess compliance with protocol requirements and tolerance of twice daily (BiD) G-CSF, as well as for dose-finding to increase the premorning dose trough absolute neutrophil count (ANC) to a predefined target range of 500–1,500 cells/μL (Figure 3 and Supplemental Table 5). The dosing rationale and procedure is detailed in Methods. The initial actual unmasked screening phase G-CSF dose ranged from 0.2 to 1.03 μg/kg BiD, close to the predefined target dose range of approximately 0.25–2.0 μg/kg BiD. The initial dose was increased by approximately 50%–100% during this phase in 3 patients (all children).

The screening phase concluded with randomization, followed by a 2-day washout of G-CSF to determine baseline blood cell counts, and then by a quadruple-masked 28-month crossover treatment period composed of two 12-month treatment phases, each preceded by a 2-month dose-finding drug equilibration phase (Figure 2). Ten patients received plerixafor first, and 9 received G-CSF first. The initial masked G-CSF dose during the equilibration phase usually equaled the final unmasked dose during the screening phase (Figure 3). The initial actual masked equilibration phase plerixafor dose was approximately 10–20 μg/kg BiD, with adjustments made within a predefined target dose range of approximately 10–40 μg/kg BiD to identify the lowest dose that increased the premorning dose trough ANC into the predefined target range of 500–1,500 cells/μL.

During the equilibration phase, no patients required adjustment of the G-CSF starting dose (Figure 3). In contrast, 8 patients required approximately 40%–225% increases of the plerixafor starting dose in 1–3 steps during the equilibration phase (Figure 3), as expected because 17 patients were plerixafor naive before the study. Patient M07 failed to reach the prespecified ANC threshold of 500 cells/μL during plerixafor equilibration and, therefore, did not enter the plerixafor treatment phase. Two other patients dropped out during an equilibration phase due to side effects, as described in detail in *Safety outcomes*: patient M09 during plerixafor equilibration and patient M14 during both G-CSF and plerixafor equilibration. Patient M17 dropped out at month 6 of the plerixafor treatment phase. Patient M06 became pregnant during month 8 of treatment phase 2. Unmasking revealed she was receiving G-CSF, which was continued.

Initial drug doses during the treatment phase were the same as the final drug doses during the preceding equilibration phase for each patient and were maintained within, slightly above, or slightly below the prespecified target ranges (Figure 3). Low ANC or an adverse event (mainly bone pain or rash) led to additional dose adjustments within or close to the target range during the treatment phase for 5 patients on the G-CSF arm and for 7 patients on the plerixafor arm (Figure 3). Analysis of the maximal G-CSF and plerixafor doses given during the treatment phase suggested 2 distinct groups of patients defined by relatively lower versus higher ANC responsiveness to both drugs. Eighty percent of the children were relatively low responders, whereas 93% of adults were relatively high responders (Supplemental Figure 2). The end-of-study visit for the final patient occurred on October 8, 2020. COVID restrictions overlapped only for patient M19, during the final 2 months of G-CSF treatment.

### Infection outcomes.

**Figure 4:**
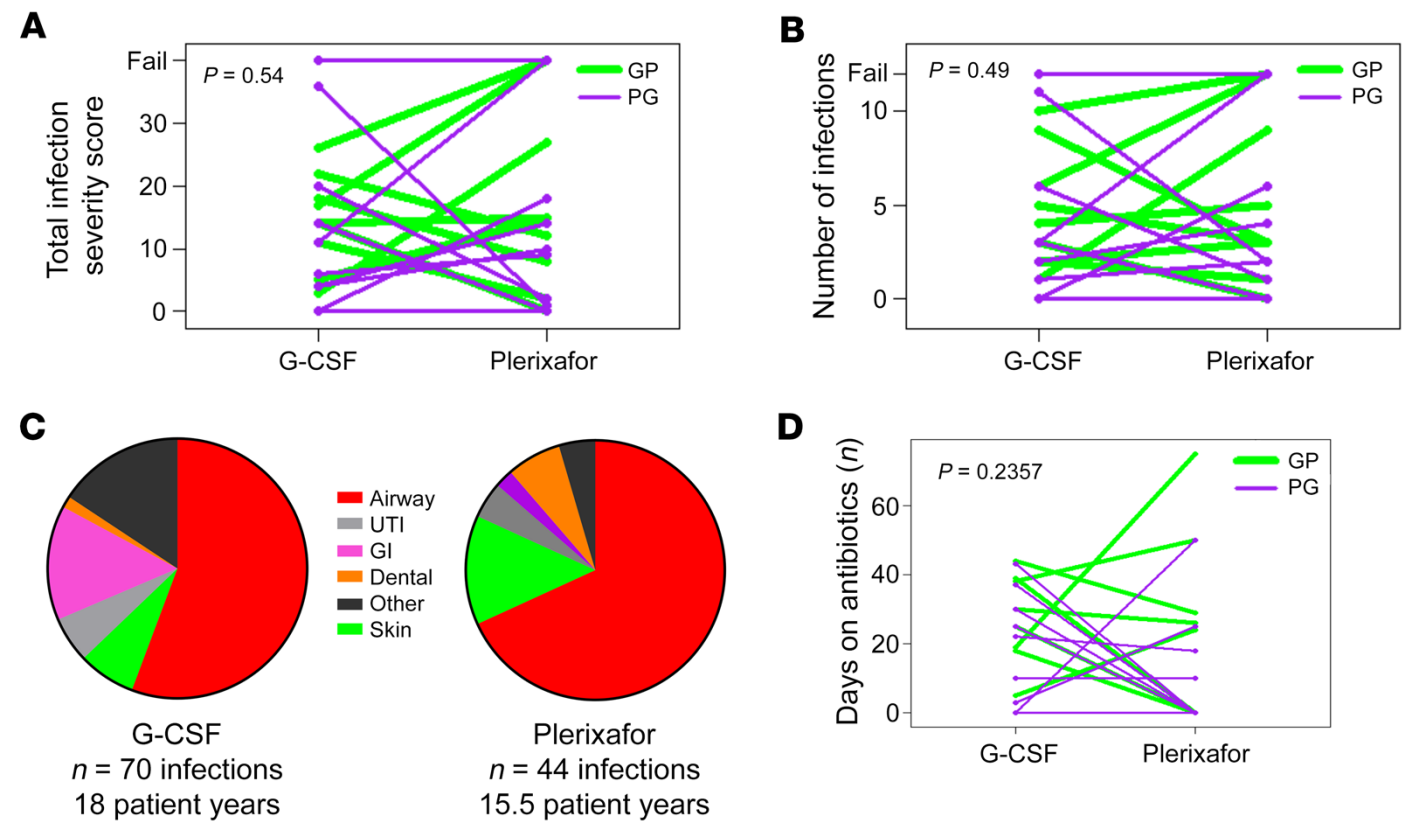
Infection severity and distribution for patients with WHIM during the plerixafor and G-CSF treatment phases. (**A**, **B**, and **D**) Drug order is color-coded in the top right corner. GP, G-CSF first followed by plerixafor; PG, plerixafor first followed by G-CSF. Each line represents a single patient, connecting results for each treatment phase. “Fail” in **A** and **B** refers to patients who dropped out because of drug intolerance or failure to meet the prespecified ANC threshold during the equilibration phase. (**C**) The infection distribution by site and the total number of patient-years of drug exposure for each treatment phase. UTI, urinary tract infection; GI, gastrointestinal infection. *P* values at the top left of each graph were determined using a Wilcoxon’s rank-sum analysis, as specified in the Supplemental Statistical Analysis Plan.

The primary endpoint was the difference between the two 12-month treatment phases for total infection severity score (TISS), a weighted composite of predefined infection frequency and severity parameters (number of infections, presence or absence of fever, sterile versus nonsterile site of infection, route of administration of antibiotics, and level of care needed) that are relevant to the participants’ experience. TISS was variable on each drug and between the two 1-year treatments and was not significantly lower for plerixafor than for G-CSF (median TISS = 11 on G-CSF and 10 on plerixafor, *P* = 0.54; Figure 4A and Supplemental Table 6). An analysis excluding the 4 treatment failures confirmed the primary analysis result (*P* = 0.6). The average number of infections was 3.89/patient-year on G-CSF and 2.84/patient-year on plerixafor, compared with the prestudy experience of 3 infections/patient-year in 11 patients with WHIM that was used to perform the primary endpoint power calculation. A ranked analysis of infection incidence as a secondary endpoint showed no difference between plerixafor and G-CSF (*P* = 0.49; Figure 4B).

Nonsterile barrier sites (skin/mucosa) accounted for 86% of 114 total infections occurring during the 2 treatment phases; 89% of all infections occurred in 5 sites: the upper respiratory tract (*n* = 69), gastrointestinal tract (*n* = 11), skin (*n* = 11), lower urinary tract (*n* = 6), and oral cavity (*n* = 4) (Figure 4C and Supplemental Table 6). The distributions of infection incidence and location were similar on plerixafor and on G-CSF (Supplemental Figure 3). Pathogens were identified for only 18 infections: influenza A, metapneumovirus, rhinovirus, enterovirus, *Moraxella* sp. and *Hemophilus sp*. for airway infections; *Pithomyces species* and *Trichophyton tonsurans* for tinea corporis and tinea capitis; HSV-1 dermatitis; *Cyclospora* enteritis and *Clostridium difficile* colitis; *Candida albicans* vaginitis; and a *S*. *aureus* skin abscess (Supplemental Table 6). All but 2 microbiologic diagnoses were made at the NIH.

No patient died, consistent with low mortality reported in the literature (3, 4). Three patients were hospitalized for 5 total infections. Two patients were hospitalized for 4 total infections during G-CSF treatment: patient M19 for 3 infections (acute appendicitis treated with surgical removal of a ruptured appendix and 7 days of intravenous antibiotics, followed by readmission 4 days later for intraabdominal abscess for 10 days of intravenous antibiotics, and later overnight for a possible urinary tract infection), and patient M07 overnight for gastroenteritis. The fifth hospitalized infection was a *S*. *aureus* axillary abscess in patient M13 on plerixafor for 1 week of intravenous antibiotics and drainage. Six additional patients visited an emergency department for 10 total infections (8 respiratory), 7 during G-CSF treatment and 3 during plerixafor treatment. The rate of hospitalization for infection/patient-year was 0.22 and 0.06 for G-CSF and plerixafor, respectively, and the rate of emergency department visits for infection/patient-year was 0.39 and 0.19 for G-CSF and plerixafor, respectively. Consistent with the G-CSF result, the rate of hospitalization for infection for the 19 patients for the 2 years preceding enrollment, when most patients were receiving G-CSF, was 0.24/patient-year. Lower respiratory tract infection is a major cause of hospitalization in patients with WHIM; however, no patients received a diagnosis of pneumonia on plerixafor, whereas 5 patients were diagnosed with pneumonia once each on G-CSF, all treated as outpatients. Antibiotics were prescribed for 89 nonhospitalized infections, 87% orally (*n* = 33 on plerixafor, *n* = 44 on G-CSF), and 12% topically (*n* = 5 on plerixafor and *n* = 6 on G-CSF) (Figure 4D and Supplemental Table 6).

Although evaluation of lung function was not a prespecified study endpoint, all 19 patients had chest computerized tomography at baseline. Of 13 patients with lung abnormalities, 10 had mild-to-severe bronchiectasis, and 7 of the 10 had mild-to-severe pulmonary function test abnormalities, particularly diminished diffusion capacity of the lung for carbon monoxide; however, neither study drug significantly improved pulmonary dysfunction (Supplemental Figure 1 and Supplemental Results).

### Immunologic outcomes.

**Figure 5:**
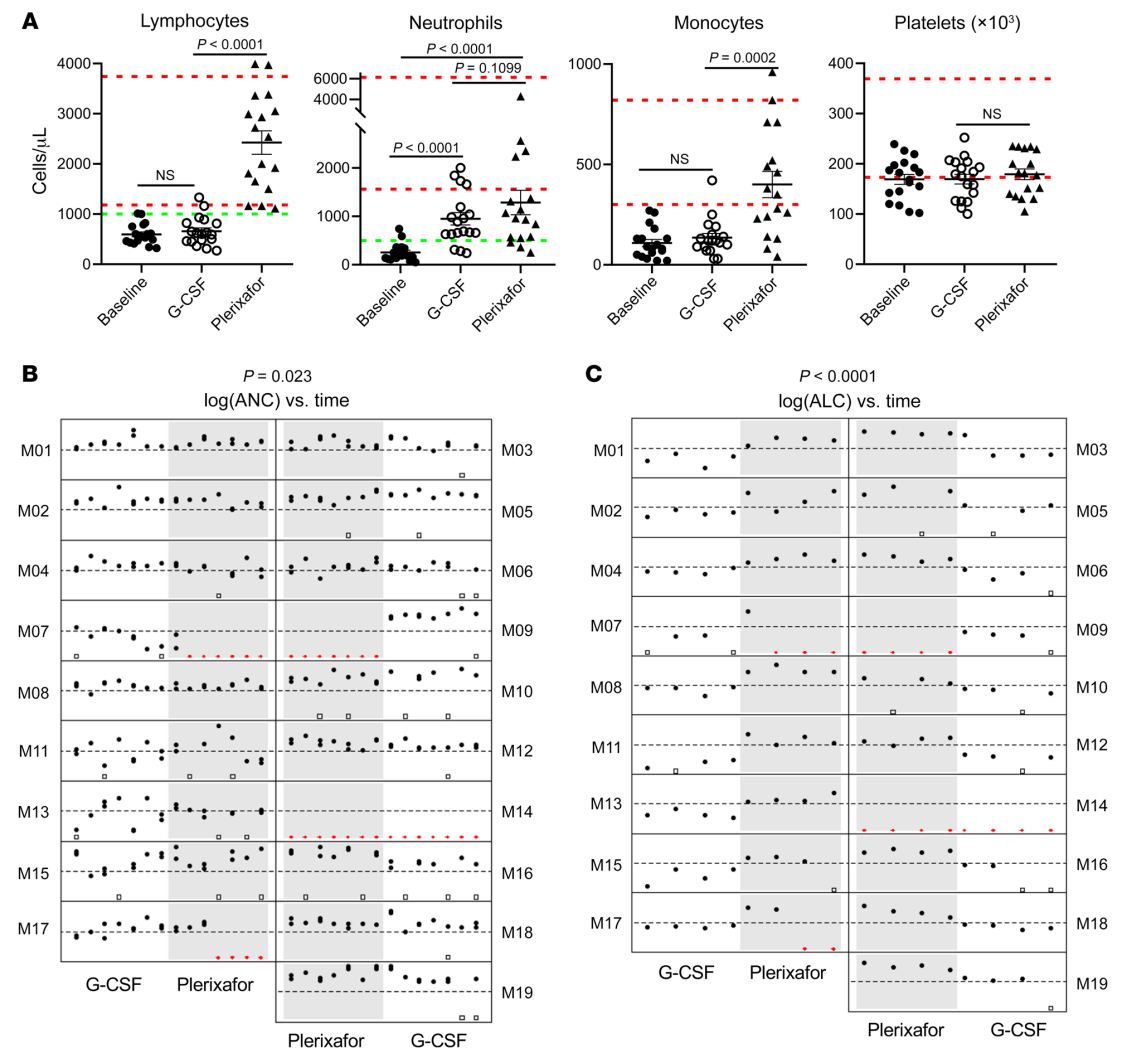
Effects of plerixafor versus G-CSF on circulating blood cell counts in patients with WHIM. (**A**) Plerixafor, but not G-CSF, reversed panleukopenia without affecting circulating platelet concentration. Baseline refers to the day 0 value of the equilibration phase after 2-day washout of G-CSF or plerixafor from the preceding phase. G-CSF and plerixafor values are the final values obtained for the approximately 3-hour postmorning dose at the end of each treatment phase. Dashed green horizontal lines indicate the prespecified thresholds for judging success for improving neutropenia and lymphopenia. Each symbol represents a different patient. Dashed red horizontal lines demarcate the normal range for adults for each parameter established by the NIH-CC Department of Laboratory Medicine. *P* values were determined by a 2-sided Wilcoxon’s matched pairs rank test. (**B** and **C**) Plerixafor is noninferior to G-CSF for durably reversing neutropenia and is superior to G-CSF for durably reversing lymphopenia for 1 year. Filled black circles indicate individual values at the indicated times; open black squares indicate missing values due to scheduling conflicts; and red triangles indicate missing data due to drug failure. The *y* axes are on a log scale from 50 to 5,000 for ANC and from 100 to 10,000 for ALC. The thresholds for judging success are indicated by dashed horizontal lines at 500 and 1,000 cells/μL for ANC and ALC, respectively. *P* values (at the top of each panel) were determined by a Wilcoxon’s matched pairs rank test, as specified in the Supplemental Statistical Analysis Plan and the Supplemental Statistical Analysis Plan Supplement.

All patients had severe baseline neutropenia and lymphopenia: the mean ± SEM ANC was 246 ± 42 (range, 50–740 cells/μL), and the mean ± SEM absolute lymphocyte count (ALC) was 597 ± 48 (range, 320–1,010 cells/μL) (Figure 5A).

Sixteen patients had the same predefined success outcome in maintaining at least 75% of planned measurements of the ANC above 500 cells/μL during both treatment phases (11 succeeded in both, and 5 failed in both), and 3 had success only on G-CSF, leading to a difference in proportion of success on G-CSF minus the proportion of success on plerixafor of 14/19 – 11/19 = 0.158 (95% CI, –0.081, 0.396), which is significantly less than the prespecified margin of 0.40 (*P* = 0.023) (Supplemental Statistical Analysis Plan). Hence, plerixafor was judged noninferior to G-CSF for maintaining the ANC above the prespecified threshold of 500 cells/μL for 1 year (Figure 5B). The ANC showed minor differences between the predose trough and approximately 3-hour postdose measurements during both plerixafor and G-CSF treatment (Figure 5B, Supplemental Figure 4A, and Supplemental Figure 5).

Regarding reversal of lymphopenia, 4 patients failed on both drugs, 1 succeeded on both drugs, and 14 succeeded only on plerixafor, leading to a difference in success proportions of 1/19 to 15/19 = –0.737 (95% CI, –0.909, –0.341); thus, plerixafor was judged superior for maintaining the ALC above the prespecified threshold of 1,000 cells/μL for 1 year (*P <* 0.0001) (Figure 5C). During plerixafor treatment, the ALC predose trough value was usually lower than the approximately 3-hour postdose value (Figure 5C, Supplemental Figure 4B, and Supplemental Figure 5).

The 4 patients who failed the ALC maintenance test during the treatment period for both drugs — M07, M09, M14, and M17 — all failed on plerixafor because they were study dropouts for that phase, as detailed previously. However, examination of data from the equilibration phase for all 4 patients and limited data from the treatment phase prior to dropout for patients M07 and M17 indicated that the ALC consistently exceeded 1,000 cells/μL on plerixafor for these patients (Supplemental Figure 5 and data not shown).

The only patients to fail the ANC maintenance test for both drugs were the 3 youngest children in the study, patients M07, M11, and M13, despite receiving the maximal allowable doses of both drugs (Figure 3). M11 and M13 passed the ALC maintenance test for plerixafor, whereas M07 failed because he did not advance beyond the starting visit of the treatment phase, having failed to raise the ANC during the equilibration phase. The adult patients M14 and M17 also failed the ANC maintenance test for both drugs. M14 dropped out during both equilibration phases because of adverse events; M17 dropped out from only the plerixafor arm but failed the ANC test on G-CSF with a score of 72%, close to the success threshold of 75%.

The 3 patients who were ANC successes on G-CSF but not on plerixafor (M04, M06, and M09) failed on plerixafor for different reasons. M09 had an adverse event during the equilibration phase that prevented entry into the plerixafor treatment phase. M04 and M06 both fell short of the prespecified 75% threshold for success (60% for M04 and 73% for M06). M04, M06, and M09 received a total actual daily dose of 92, 33, and 33 μg/kg/d of plerixafor, respectively, relative to the prespecified total target dose range for the protocol of 20–80 μg/kg/d.

**Figure 6:**
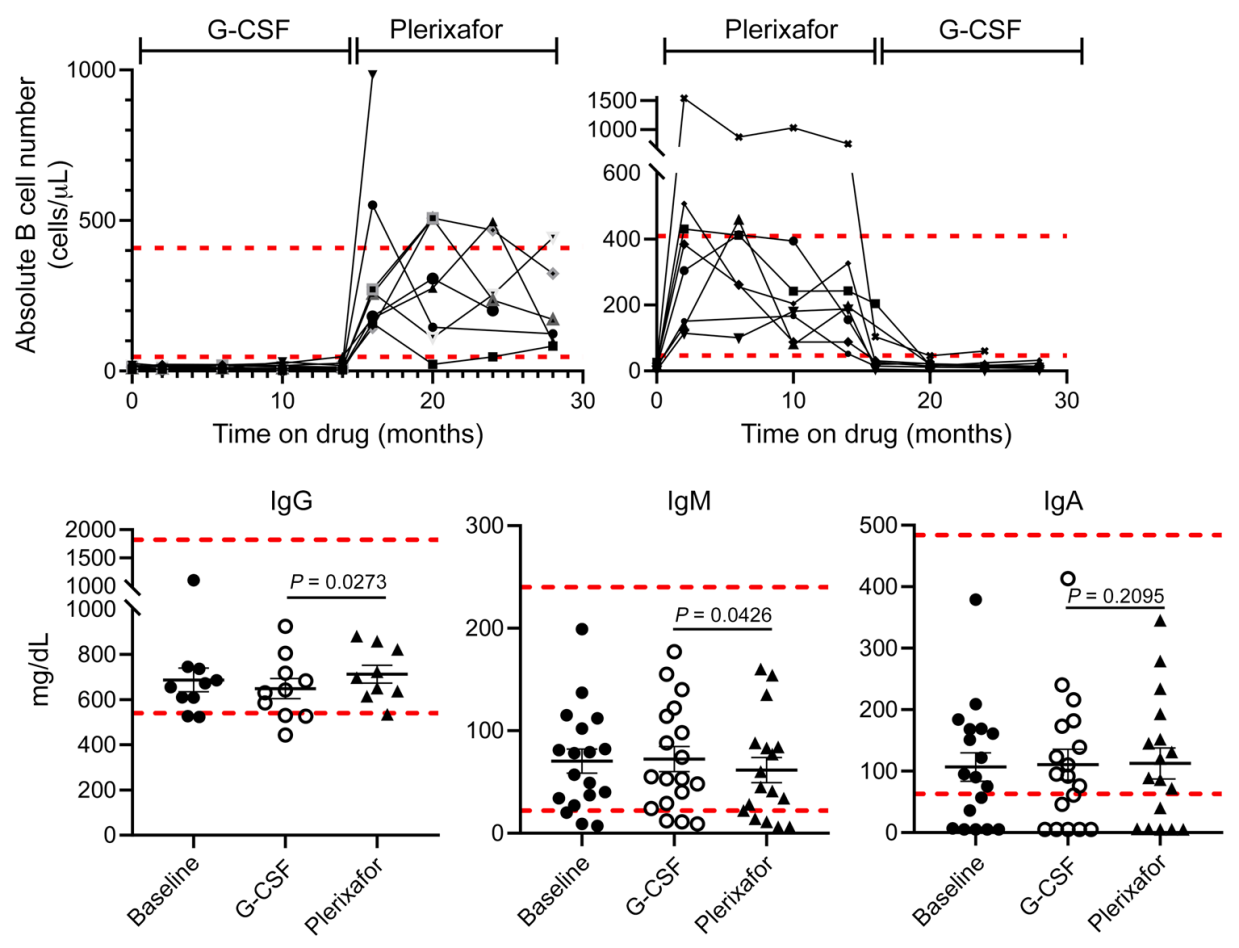
Normalization of circulating B cell levels by plerixafor did not affect immunoglobulin levels. Dashed horizontal lines demarcate the normal range for each parameter established by the NIH-CC Department of Laboratory Medicine. Top: B cells. The time on each drug includes both equilibration and treatment phases and is indicated at the top by brackets. Each graphed line represents data for a single patient. Bottom: Immunoglobulin levels. G-CSF and plerixafor data designate the final value obtained at the end of each treatment phase. IgG data are only shown for patients not receiving supplemental immunoglobulin. *P* values are only shown for the drug comparisons and were determined by a 2-sided Wilcoxon’s matched pairs rank test. Comparisons of day –0 baseline data to data on each drug were not significant.

The baseline absolute monocyte counts were below the lower limit of normal for all but 1 patient and normalized on plerixafor for most patients but did not increase on G-CSF (Figure 5A and Supplemental Figure 4C). Almost all patients had severe B lymphopenia that was unresponsive to G-CSF, whereas plerixafor durably increased B cells into the normal range for most patients (Figure 6). Circulating T cell levels followed the same pattern of being G-CSF unresponsive and plerixafor responsive; however, the details varied by subset (Supplemental Figure 4, D–L, and data not shown). Total CD8^+^ T cells matched the B cell pattern of severe baseline deficiency reversed by plerixafor but insensitive to G-CSF. Total CD4^+^ T cells were below the lower limit of normal for a subset of patients but could be increased to and maintained in the normal range for all patients by plerixafor. Central and effector memory CD4^+^ and CD8^+^ T cells followed the total CD4^+^ T cell response patterns. In contrast, although naive CD4^+^ and CD8^+^ T cell numbers were severely deficient at baseline in all but 1 patient, plerixafor was able to increase them in only 7 patients (Supplemental Figure 4, I and J): the adult siblings M03 and M05 and all 4 evaluable pediatric patients. In yet another pattern, the baseline absolute numbers of NK cells were normal for all but 5 patients, and early increases on plerixafor were not consistently sustained (Supplemental Figure 4K). Finally, the NK-T cell response pattern resembled that of total CD4^+^ T cells (Supplemental Figure 4L).

Baseline serum IgG levels were below the lower limit of normal for 2 of 11 patients not receiving supplemental IgG, and baseline serum IgM and IgA levels were low in only 2 and 7 of the 19 patients, respectively (Figure 6). Pediatric patients M07 and M11 and adult patients M12 and M15 consistently had IgA levels below the limit of detection (5 mg/dL). All 4 patients were receiving supplemental IgG and had IgM levels below or at the lower limit of normal and in the lower 25% of all patients, consistent with selective IgA deficiency, which has not previously been reported in WHIM syndrome. By contrast, the prevalence of selective IgA deficiency in White individuals is 0.2%–0.25% (23). IgA deficiency is typically associated with gastrointestinal and airway infections as well as conjunctivitis; however, the medical histories and study events for these patients included mainly airway and skin infections that were not distinguishable in frequency or severity from the other 15 study participants (Supplemental Tables 3 and 6). Correction of B lymphopenia by plerixafor did not correct IgG, IgM, or IgA deficiency in any patients (Figure 6 and data not shown). In this regard, young WHIM model mice have severe B lymphopenia but not hypogammaglobulinemia (24).

Neither drug affected patient hemoglobin levels, which were normal at baseline, nor the platelet count, which distributed below and at the lower limit of the normal range at baseline (Figure 5A and Supplemental Figure 4, M and N). At baseline and after both treatment phases, patient and healthy control PBMCs responded similarly to stimulation with IL-2, PHA, concanavalin A, pokeweed mitogen, *Tetanus* toxoid, and *Candida* antigen, and mixed lymphocyte reaction responses were similar (Supplemental Figure 6 and data not shown).

### Wart responses.

At baseline, 69 wart areas were defined for 13 patients with warts (average 5.3/patient; range 1–16/patient), 87% on the upper or lower extremities (Table 2, Supplemental Table 7, and Supplemental Figure 7). Two additional patients had a history of warts but could not be evaluated: patient M08, whose warts had been surgically removed and patient M16, who lacked the necessary clinical photography. Two patients with warts were children (M01 and M07). Eight patients had anogenital warts, including pediatric patient M07. Eight patients with warts had received chronic G-CSF before enrollment; in each case, serial photographs indicated that wart burden had been stable for 0.75–8 years before enrollment (Supplemental Figure 7).

**Figure 7:**
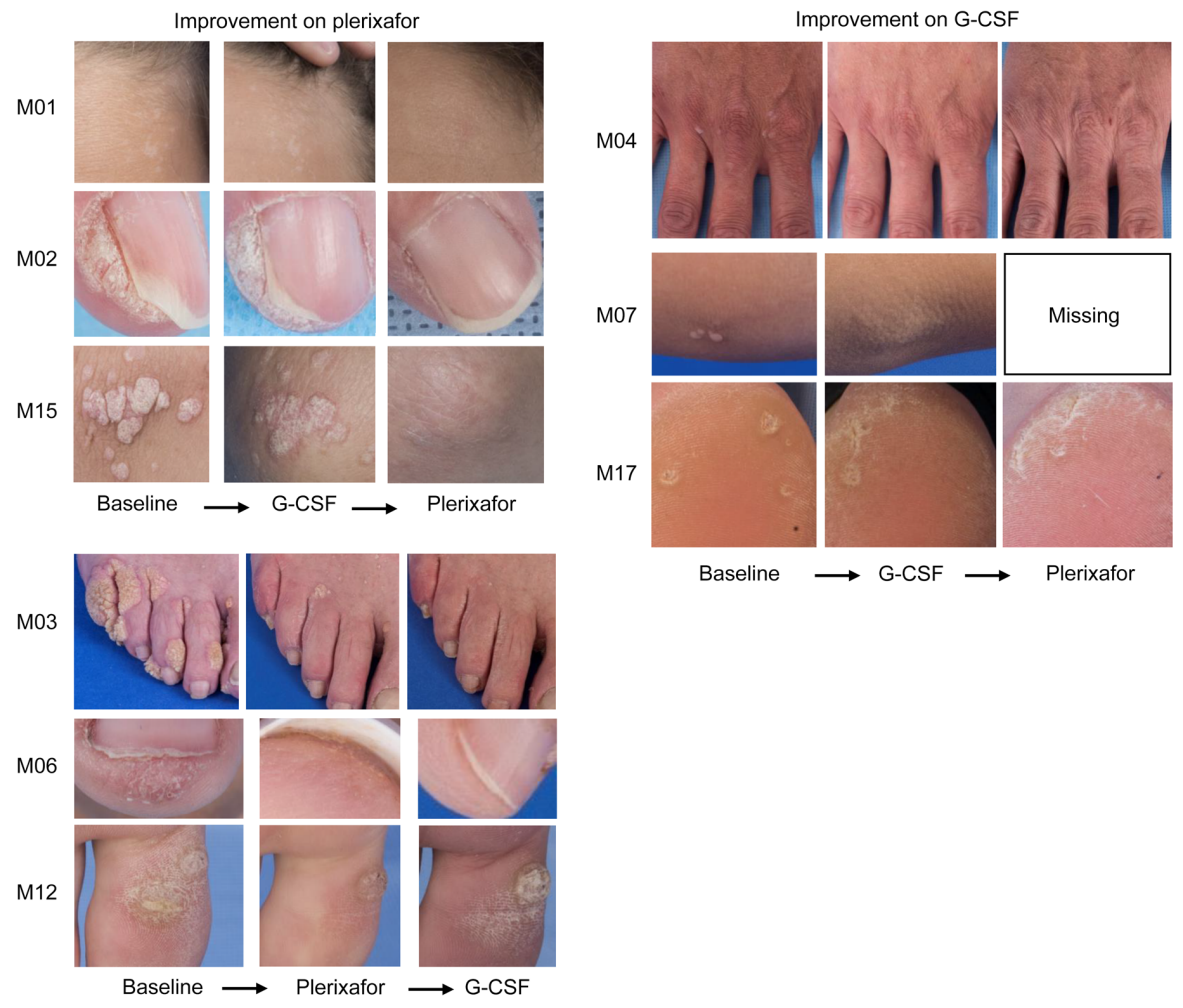
Plerixafor and G-CSF effects on wart burden in patients with WHIM. Representative images of warts at baseline and at the end of the indicated drug treatment period are shown for patients demonstrating improvement in wart areas during the study. Images are for the patient indicated to the left of the corresponding row. Comprehensive assessments of wart changes on drug treatment are detailed in Table 2, Supplemental Tables 7–13, and Supplemental Figure 7.

The statistical analysis plan limited analysis of wart regression to treatment phase 1 because of the possibility of carryover effects. In this phase, there was detectable wart regression in 3 patients given plerixafor (M03, M06, and M12) and in 3 patients given G-CSF (M04, M07, and M17) (Figure 7, Supplemental Figure 7, Table 2, and Supplemental Tables 7 and 8). Nevertheless, plerixafor induced complete regression of multiple large wart areas in patient M03 and 1 large wart area of patient M12 during this phase, whereas G-CSF induced complete but clinically insignificant regression of only 2 small elbow wart-like lesions of patient M07. Wart regression for patients M04 and M17 on G-CSF and for M06 on plerixafor during the first treatment phase involved partial regression of very small warts in patients with minor wart burdens (Figure 7 and Supplemental Table 8). Given this large disparity in effect size, we performed exploratory analysis of both treatment periods.

For 4 patients (M05, M09, M10, and M18) wart burden was unaffected by either drug. The other 9 patients (69%) improved on at least one treatment (Figure 7, Supplemental Figure 7, Table 2, and Supplemental Tables 7–9). Eight patients improved on plerixafor, 5 with complete regression of at least 1 large wart area. Of these 5 patients, 3 had received G-CSF first without improvement and 2 had received plerixafor first. Four patients improved on G-CSF, but major clinically substantial improvement on G-CSF occurred only in patient M03, in large verrucous areas on several fingers early in treatment phase 2 following complete regression and partial regression in multiple other wart areas on plerixafor during treatment phase 1, suggesting a possible carryover effect from plerixafor (Figure 7 and Supplemental Figure 7). Minor increases in wart burden were observed in 5 patients on G-CSF and in 1 patient on plerixafor.

Of the 69 wart areas identified at baseline in the 13 patients, 26 improved on plerixafor, 13 improved on G-CSF, 1 worsened on plerixafor, and 10 enlarged on G-CSF. Responses were highly heterogeneous in responding patients (Supplemental Tables 7 and 8). For example, patient M03 had complete regression of multiple large warts on both hands and feet but little to no change in other areas, including the genitalia. Most wart regression on his hands and feet was complete after the 12-month plerixafor treatment, whereas 1 finger wart began to regress soon after crossover to G-CSF. After wart regression began on plerixafor, he applied imiquimod to the genitalia, one hand, and one foot from month 8 to 12 of plerixafor treatment and not after crossover to G-CSF. Patient M15 also applied imiquimod but only to genital warts and only for the first 4 months on plerixafor, yet had major regression of all wart areas on her upper extremities starting at approximately month 8 of plerixafor treatment, without genital wart regression. Patient M05 also applied imiquimod but had no wart regression on either G-CSF or plerixafor. Anogenital wart regression occurred only on plerixafor but was minor and in only 2 patients (M02 and M04) (Supplemental Table 10). In 65% of cases, we first detected wart regression on plerixafor at the 8- or 12-month visits (Supplemental Table 11). Both large and small warts and different wart types regressed (Supplemental Table 12). The distribution of CXCR4 genotypes was similar for wart responders and nonresponders.

A plerixafor dose-response relationship for wart regression was not apparent (Supplemental Table 13). All 13 patients with warts received a full treatment course of G-CSF, whereas 10 patients with warts received a full treatment course of plerixafor (patients M07 and M09 received only 2 and 1 months of treatment, respectively, in the equilibration phase for plerixafor, and patient M17 dropped out at month 6 of the treatment phase for plerixafor; none of these patients experienced wart regression).

The 5 patients with major wart responses on plerixafor represented 71% of the subset of 7 patients with major wart burdens (Supplemental Tables 7, 8, and 13). These 5 patients had an average of 1.6 infections/patient-year on plerixafor and included the only 3 patients who had no infections while on plerixafor (patients M01, M03, and M12), whereas all patients who experienced no wart response or a minor wart response on plerixafor had at least 1 infection during that treatment phase and overall an average of 3 infections/patient-year (Supplemental Table 13).

We previously published a survey of HPVs infecting immunodeficiency patients, including 10 patients from the present study before enrollment (20, 25). Here, we considered further the data for the 10 patients with WHIM. We identified HPV sequences by rolling circle amplification in all skin and genital samples submitted from all 10 patients and from none of the blood samples submitted from any of 4 of the 10 patients. Ninety-five HPV types were identified in the samples (Supplemental Table 14). Of 62 unique HPVs, there were 10 α, 13 β, 37 γ, and 2 μ types, a distribution that is strongly skewed toward γ types compared with the HPV distribution in the general population. Follow-up samples were unavailable to test the effect of plerixafor or G-CSF treatment on baseline HPV distribution.

Of 62 HPVs identified, 16 were novel, all gammas, including 13 types and 3 species. HPV diversity correlated poorly with HPV disease burden. As extreme examples, patients M09 and M16 had low wart burdens yet harbored many HPV types, even in areas lacking warts. In particular, M09 had widespread plerixafor-induced psoriasiform lesions with no warts, from which we identified 8 different HPV types, including 5 γ and 3 β types. Likewise, M16 had a large pedunculated gluteal lipoma associated with 6 different HPV types, including 5 γ and one β type.

### Safety outcomes.

Seven serious adverse events (SAEs) occurred among 6 patients, 1 during the open-label G-CSF posttreatment phase and 6 during a treatment phase (1 during plerixafor treatment only, 4 during G-CSF treatment only, and 1 during both plerixafor and G-CSF treatment) (Table 3). Five treatment phase SAEs were infections requiring hospitalization and, therefore, were not unanticipated, as described in *Infection outcomes*.

A sixth SAE that was unanticipated and probably related to both study drugs was new onset reactive additive polyarthritis occurring in the wrists, hands, and knees of patient M14 on both plerixafor and G-CSF. He first noticed mild arthralgia in his hands 10 days after beginning open-label G-CSF during the screening phase. After starting plerixafor as study drug number 1, symptoms intensified and spread to involve the other joints. Plerixafor was stopped after 18 days. Workup revealed a friable urethra that was painful on gentle dacron swabbing, with bleeding and a yellowish discharge. *Chlamydia sp*. was detected by nucleic acid amplification of urethral exudate, which was treated with 1 g azithromycin. Arthritis resolved on a short course of prednisone and sulfasalazine, and repeat PCR 2 months later was negative for *Chlamydia sp*. He was then given masked G-CSF with return of disabling arthritis after 1 week. G-CSF was discontinued, and the symptoms resolved on prednisone and sulfasalazine. During 2.5 years of follow up, he was not treated with either G-CSF or plerixafor and had 3 mild flares limited to the wrists, which responded to short courses of low-dose prednisone. The patient was G-CSF and plerixafor naive prior to the study and did not have a prior history of arthritis, urethritis, or *Chlamydia* infection; rheumatoid factor was negative. His biological son has both juvenile rheumatoid arthritis and WHIM syndrome.

One unanticipated noninfectious SAE occurred on G-CSF and was judged unrelated to the drug: a transient ischemic attack in patient M05 during outpatient gynecologic surgery. There was no prior history of cerebrovascular events, and she has had no recurrence through June 2023.

Another 207 noninfectious adverse events in 58 descriptor categories occurred during the trial (an average of 0.33 versus 0.40 noninfectious adverse events/patient-month on G-CSF versus plerixafor, respectively) (Supplemental Table 15). Bone pain, joint pain, and transient rash were most common, affecting 15, 14, and 9 patients, respectively, and accounted for all adverse events considered definitely related to a study drug (Table 4). Bone pain was significantly more common on G-CSF, and transient eczematous rash mainly affecting the palms and soles was significantly more common on plerixafor. Patient M17 dropped out during month 6 of the plerixafor treatment period because of worsening of baseline arthralgia.

Patient M09, who had a family history of psoriasis, dropped out during the plerixafor equilibration phase because of extensive new onset psoriasis. The lesions completely resolved when plerixafor was discontinued and after a short course of topical corticosteroids and did not flare during treatment with G-CSF, which was given second. She subsequently experienced one other psoriasis flare over 5 years since the end-of-study visit, during a phase II clinical trial for WHIM syndrome of the chemically distinct small-molecule CXCR4 antagonist mavorixafor (26).

A second group of less frequent, transient, and tolerated grade 1 or 2 adverse events that were considered probably or possibly related to a study drug and were not significantly different in frequency on the 2 drugs included headache in 9 patients, hyperuricemia in 7 patients, weight gain in 7 patients, nausea in 7 patients, and injection site reactions in 4 patients. The remaining adverse events were transient minor laboratory abnormalities or were mild and affected only a few patients.

### Survey outcomes.

When asked at study completion and before unmasking, 10 patients favored plerixafor, 4 favored G-CSF, 3 had no preference, and 2 patients did not respond (Table 5). Two patients preferred G-CSF because of transient rash on plerixafor. M09 and M17, who dropped out on plerixafor because of rash and arthralgia, respectively, preferred G-CSF. Of 15 patients who completed treatment with both drugs, 10 preferred plerixafor, citing less bone pain, more energy, and/or reduced wart burden. This difference in drug preference was not statistically significant. Overall, quality of life, as assessed by the Short Form-36 question health survey version 2 questionnaire, was not significantly different between the 2 drugs (Supplemental Table 4 and Supplemental Results).

## Discussion

In this crossover treatment study for WHIM syndrome, plerixafor was not superior to G-CSF for the primary endpoint of total infection severity scored over 1 year for each drug. The study had limitations for demonstrating a potential plerixafor advantage over G-CSF for controlling infections, including (a) the primary endpoint, which includes subjective measures of medical decision-making and judgment (diagnostic accuracy, level of care, antibiotic usage), as well as the simple classification of infections (sterile versus nonsterile site), and which does not require a pattern of recurrence typical of WHIM syndrome or exclude common infections unrelated to immunodeficiency that might attenuate a benefit signal; (b) using G-CSF, the standard-of-care in SCN (14), as the comparator, which sets a high bar for demonstrating potential plerixafor superiority; (c) coadministration of immunoglobulin and prophylactic antibiotics in several of the patients; (d) potential for carryover effects due to the crossover design; and (e) the short period on drug, the low dose used and the few patients available for study.

Since WHIM syndrome is a type of SCN, and G-CSF is the SCN standard-of-care, we chose G-CSF as the comparator instead of placebo. Nevertheless, it is important to point out that G-CSF efficacy has not specifically been assessed by a placebo-controlled clinical trial in WHIM syndrome. Restricting the primary endpoint to a particular type of infection or level of severity or else to recurrent infections would have imposed a level of arbitrariness on how to set boundaries and made adequately powering the study impractical. However, not doing so imposed a potential cost of attenuating an infection efficacy signal. Strikingly, few severe infections occurred on either drug. No patient was hospitalized during a treatment phase for respiratory infection, the most common cause of hospitalization in patients with WHIM (1, 2, 4, 27). Moreover, there were no new persistent infections on the study, and all but 5 infections were treated in the outpatient setting, 2 of which were for overnight observation given the history of immunodeficiency. Only 18 infections resulted in a specific pathogen being identified, and most of these were common viruses. This highlights the difficulty under real world conditions of defining pathogens in the nonsterile sites typically infected in patients with WHIM.

Our exploratory results confirm previous reports of clinically significant wart regression in patients with WHIM treated with plerixafor or the unrelated CXCR4 antagonist mavorixafor (19, 26) and justify additional investigation of longer treatment courses and different dosing schedules of plerixafor to improve efficacy. Our study identified subgroups of plerixafor responder and nonresponder patients with warts, plerixafor responder and nonresponder wart areas in the same patient, and an apparent refractory state of genital HPV disease to plerixafor in patients demonstrating major regression of cutaneous warts on the drug. In this regard, we found that diverse and unusual HPV types infect patients with WHIM, with a predominance of β and γ types and multiple types infecting the same patient, the same wart, and even nonverrucous lesions and nonlesional skin (Supplemental Table 14) (20, 25). There is evidence that CXCL12 and CXCR4 are coexpressed in WHIM and non-WHIM warts, and CXCR4 has been detected on keratinocytes in warts but not in normal skin (28). Moreover, in model in vitro systems, CXCL12 signaling has been reported to promote HPV-mediated transformation of keratinocytes (29, 30). For this reason, blocking keratinocyte CXCR4 must be considered as a potential wart response mechanism in our study. In addition, a patient with WHIM with warts was cured as an adult of WHIM syndrome by chromothriptic deletion of the disease allele solely in her myeloid lineage, which suggests that plerixafor might also benefit warts by blocking CXCR4 on myeloid cells (31).

Our results also highlight the diverse mobilization sensitivity of different leukocyte subsets to plerixafor. Neutrophils appeared less responsive than monocytes, B cells, and memory CD4^+^ and CD8^+^ T cells, which were durably increased by plerixafor into the normal range for most patients. Additional studies will be needed, for example, of CXCR2 and CXCR4 expression, in both patients with WHIM and healthy individuals to investigate the apparent relative neutrophil mobilization insensitivity of patients with WHIM to plerixafor. Neutrophil responses in pediatric participants were weaker than those in adults, despite excellent lymphocyte responses, including for naive T cells, which conversely were poorly mobilized by plerixafor in most adults. Despite severe baseline lymphopenia, we documented normal proliferative T cell responses to mitogens, environmental organisms, and vaccine antigens at baseline and after plerixafor treatment (Supplemental Figure 6), consistent with the observation that, apart from HPV, infection with opportunistic pathogens is uncommon in WHIM syndrome.

Our study confirmed the disparity between severe circulating B cell deficiency in patients with WHIM and the relatively modest effect on circulating immunoglobulin levels affecting a subset of patients, with IgG more consistently and quantitatively affected than IgA or IgM. Nevertheless, we made a potentially new observation of unexpectedly high incidence of selective IgA deficiency at baseline in 4 of 19 patients on study, which may represent an incompletely penetrant WHIM phenotype. Despite sustained normalization of the circulating absolute total B cell count by plerixafor, hypogammaglobulinemia did not improve.

With regard to safety, both drugs caused one unexpected and idiosyncratic but reversible inflammatory side effect in patients with risk factors for the condition but no prior expression of it (psoriasis, reactive arthritis). We speculate that WHIM immunodeficiency may protect from immunologically mediated disease in such predisposed patients. Bone pain was common in patients receiving G-CSF, despite the low doses used. Eczematous rash of the palms and soles was common on plerixafor, but it was transient and did not result in any dropouts. Side effects, especially bone pain, were most commonly cited by patients in deciding their drug preference, which trended in favor of plerixafor. This result from the study will be important during informed decision making between physicians and patients.

The lower age limit for participation (10 years of age) precluded our ability to judge the safety and efficacy of plerixafor in younger children. Our findings, together with recent results suggesting that early diagnosis may improve outcomes in WHIM syndrome (3), may justify testing plerixafor in this age group. Importantly, the drug doses in our study were much lower than those recommended for G-CSF in SCN and for plerixafor in HSC mobilization.

In conclusion, plerixafor was not superior to G-CSF for control of infection severity, the primary endpoint. The study was not designed to answer whether plerixafor is noninferior to G-CSF for infection severity; however, no differences between the G-CSF and plerixafor arms were found for any infection outcome measures. The exploratory endpoints suggested that plerixafor may be noninferior to G-CSF for durably increasing the ANC and may have an advantage over G-CSF for elevating the ALC, for wart regression, and for limiting bone pain. Dermatitis or arthritis severe enough to stop treatment occurred in 3 patients while on plerixafor and 1 patient while on G-CSF.

## Methods

### Trial design and oversight.

The study has a randomized, quadruple-masked (participants, care providers, investigators, and outcome assessors), crossover design comparing plerixafor with G-CSF, and it was conducted at the NIH-CC. The trial was investigator initiated and designed and was sponsored by the National Institute of Allergy and Infectious Diseases Division of Clinical Research. Additional details can be found in the Supplemental Methods section. The protocol was composed of 6 phases (Figure 2). The first 5 phases (screening as well as 2 masked drug equilibration and treatment crossover phases) are described in the Results section. The sixth phase is a posttreatment phase, in which patients were offered open-label G-CSF and followed for approximately 6 months until the end-of-study visit. The primary and secondary endpoints were compared between the two 12-month treatment phases. The results we report follow the 2010 CONSORT guidelines (32). The full protocol can be accessed at Clinicaltrials.gov (NCT02231879).

### Patients.

Eligibility criteria included a 10- to 75-year age range, a baseline ANC of less than 1,500 cells/μL, a history of recurrent infections, and a *CXCR4* mutation damaging the *C*-terminus of CXCR4. A history of treatment with G-CSF or plerixafor was not disqualifying, and continuation of prophylactic antibiotics and immunoglobulin supplementation according to best medical practice was allowed. Additional details are provided in the Supplemental Methods.

### Treatment.

G-CSF was purchased from Amgen, and plerixafor was provided by the manufacturer, Sanofi-Genzyme. Both drugs come in similarly sized vials and are indistinguishable, clear, colorless, sterile, and nonviscous liquids (1.6 μL at 300 mg/mL for G-CSF and 1.2 μL at 20,000 μg/mL for plerixafor). Either the Pharmaceutical Development Section of the NIH-CC Pharmacy or Integrity Bio Inc. transferred the drugs undiluted under cGMP conditions into unmarked borosilicate syringes in 5 different predefined amounts: 15, 22.5, 36, 54, and 75 μg G-CSF (corresponding to 0.05, 0.075, 0.12, 0.18, and 0.25 mL, respectively) and 800, 1,200, 1,800, 2,600, and 3,800 μg plerixafor (corresponding to 0.04, 0.06, 0.09, 0.13, and 0.19 mL, respectively). These 2 ranges were predefined to deliver target doses ranging from approximately 0.25 to 2.0 μg/kg BiD for G-CSF and approximately 10 to 40 μg/kg BiD for plerixafor for the body weight range anticipated for the study participants. The predefined target dose ranges for both drugs were expected to be sufficient, based on previous experience in treating patients with WHIM, who do not have a myeloid block and have mobilizable leukocyte pools (16, 18–20), to increase the premorning dose trough ANC into a predefined target range of 500–1,500 cells/μL. The target threshold of 500 cells/μL was chosen because (a) although the relationship between ANC and infection susceptibility has not been established in patients with WHIM, 500 cells/μL is an established ANC safety threshold below which the risk of bacterial infection increases in patients with cancer receiving chemotherapy (33); (b) most patients with WHIM have a baseline ANC of less than 500 cells/μL; and (c) drug exposure would be limited to the lowest level compatible with the desired hematologic response. The latter consideration was important for plerixafor, because there was little preclinical or clinical experience with chronic administration and because *Cxcr4*-knockout mice are nonviable (34). It was important for G-CSF because high doses of the drug cause significant bone pain, potentially resulting in patient dropout. The predefined plerixafor target dose range of approximately 10–40 μg/kg BiD is less than the FDA-approved dose for HSC mobilization of 240 μg/kg daily for 4 days (21). BiD dosing was selected because peak plasma concentrations of plerixafor are observed at 30–60 minutes after subcutaneous injection in both healthy individuals and patients with WHIM and because the half-life is approximately 5 hours. The predefined G-CSF target dose range of approximately 0.25–2.0 μg/kg BiD is lower than the recommended initial dose for SCN of 6 μg/kg BiD (14) and was selected because our prestudy experience over 10 years in 16 patients with WHIM at the NIH had shown that low doses could be effective at raising the ANC to more than 500 cells/μL and were well tolerated.

For patients already taking open-label G-CSF at enrollment, the unmasked prefilled G-CSF syringe amount prescribed to start the screening phase was judged based on the preenrollment dose, the preenrollment ANC response, and patient weight. Those not already taking G-CSF at enrollment started the screening phase using the syringe containing either the lowest or second lowest G-CSF dose. Dose adjustments using the 5 available syringe amounts were made during the screening phase if necessary, guided by periodic blood count assessments, to increase the morning dose trough ANC into the predefined target range of 500–1,500 cells/μL. To start the masked and randomized equilibration phases, the masked principal investigator selected both a prefilled G-CSF syringe dose based on the patient’s screening phase response and a prefilled plerixafor syringe that would deliver a dose within the lower portion of the target dose range, i.e., approximately 10–20 μg/kg BiD. The unmasked pharmacist then dispensed the correct syringe option based on the randomization. The initial G-CSF dose in this phase was, in most cases, the same as the dose the patient had already been receiving at the end of the screening phase. From these starting doses, adjustments were made during the equilibration phase using the same prescribing procedure for the other 4 prefilled syringe amounts for each drug, guided by masked ANC assessments every 2 weeks before the morning dose, until the ANC reached at least 500 cells/μL. Patients who failed to reach this threshold by 8 weeks were declared drug failures and were switched to the second drug. Patients who succeeded during the equilibration phase were continued on the effective dose for a 12-month treatment phase. The dose could be further adjusted within the prespecified range for each drug during the treatment phase if the ANC fell below 500 or above 7,500 or to mitigate an adverse event thought to be related to the drug. Patients unable to tolerate a study drug or who met prespecified failure criteria were switched in the first treatment period to the alternate agent or in the second treatment period to open-label G-CSF.

### Assessments and end points.

The prespecified primary efficacy endpoint was the difference between the two 12-month treatment periods for the TISS, a weighted composite of predefined infection frequency and severity inputs defined for this study. Records of each medical encounter for infection were collected from the treating health care provider and scored by the study team according to the predefined point system shown in Table 6.

The points for each parameter in each column were added for each infection to produce an infection severity score, which could range from 1 to 10. For each patient, infection severity scores for all infections occurring during a treatment phase were added to generate a TISS for that phase that was then compared with the TISS for the second treatment phase. Additional details are provided in the Supplemental Methods.

Ordered secondary endpoints included sustained ANC and ALC improvement; infection incidence; antibiotic treatment duration; wart regression; and quality of life based on the 36-Item Short Form Survey, version 2 instrument (Supplemental Methods and Supplemental Statistical Analysis Plan). Sustained ANC and ALC improvement was defined on an intent-to-treat basis as a minimum of 75% of visit measurements during a treatment phase that met or exceeded prespecified thresholds (500 and 1,000 cells/μL for ANC and ALC, respectively), reasoning that even imperfect maintenance of the counts above the threshold would be clinically desirable. For ANC, we included in the analysis measurements before the morning dose and approximately 3 hours after the morning dose at NIH at month 0 (day 1 of the treatment phase), 4, 8, and 12 visits during each treatment phase as well as the measurements before the morning dose by the local laboratory at months 2, 6, and 10 of each treatment phase (measurements after the morning dose were not done at those visits). Because our phase 1 data showed that the ALC tended to approach baseline within 12 hours of administering plerixafor (19), to evaluate sustained ALC improvement we considered only ALC measurements after the morning dose at the 4 NIH visits during each treatment phase. Note that the month 0 visit measurements came after 2 months of treatment with the same drug during the equilibration phase of the study. Drug failures occurring during an equilibration phase and dropouts during a treatment phase were counted as maintenance failures during the treatment phase and given the worst scores in a ranked analysis.

Clinical photography and dermatologist assessments of warts were obtained at baseline on day 0 of the equilibration phase as well as at the month 0, 4, 8 and 12 visits during the treatment phase. Two masked dermatologists scored the percentage change in baseline wart area at each of the 4 visits during each treatment phase. A complete response was defined as visible absence at the month 12 visit of a wart area defined at the baseline visit. Because carryover effects on warts were possible during drug crossover, the prespecified statistical analysis plan compared improvement only for the first treatment phase. HPV identification was as previously reported (25, 35).

Blood for quantitative immunoglobulin and lymphocyte subset assessments was obtained at the NIH-CC before the first study drug dose on day 0 of each equilibration phase and approximately 3 hours after the morning dose at the month 0, 4, 8, and 12 visits of each treatment phase. Immunophenotyping was performed by the Department of Clinical Immunology at the NIH-CC using freshly isolated PBMCs. Cells were washed with PBS, fixed with 1.0% formaldehyde, resuspended in PBS, and then stained for cell surface expression of CD45, CD3, CD4, CD8, CD14, CD56, CD45RA, CD62L, and CD19 using directly labeled monoclonal antibodies (Supplemental Table 16). Lymphocyte proliferation assays assessed freshly isolated PBMCs from study patients and healthy donors obtained at the day –0 baseline and month 12 visits for each treatment, as detailed in the Supplemental Methods. Normal ranges for leukocyte and immunoglobulin subsets were those defined by the NIH-CC Department of Clinical Immunology. Clinical laboratory assessments complied with Clinical Laboratory Improvement Amendments standards.

We report all adverse events for all study phases.

### Statistics.

Participant sample size of 20 was defined from retrospective frequency and severity data on infections in 11 patients with WHIM treated with G-CSF at the NIH-CC for 1 year (as detailed in the Supplemental Protocol and the Supplemental Statistical Analysis Plan), which gave a power of 90%, assuming a reduction of 50% between the TISS score during the plerixafor and G-CSF treatment phases. Detailed rules for missing data and drug failures were prespecified in the Supplemental Statistical Analysis Plan, and all analyses were performed using an intent-to-treat paradigm. For the primary analysis we used a 2-sample Wilcoxon’s test under a ranking scheme, where we formed a score *S* for each participant, defined as follows: *S* = (TISS during treatment phase 1 – TISS during treatment phase 2), for a patient who did not have a drug failure and had complete follow up. Patients who failed to complete a treatment phase were given the worst score for that phase. Because the order of drug administration was randomized, if there were phase effects or carryover effects, the methods are still valid. A 2-sample Wilcoxon’s rank sum test analyzed TISS differences between plerixafor and G-CSF at the 2-sided 0.05 level. If significant, we would proceed to test the prespecified secondary outcomes in the predefined order using the fixed-sequence method, each at the 1-sided 0.025 level adjusting for multiple comparisons.

We used a noninferiority test to assess sustained ANC improvement because both drugs are known to increase the ANC, and we used a superiority test to assess sustained ALC improvement because plerixafor, but not G-CSF, is known to elevate the ALC. The effect parameter for ANC is the proportion of successes (at least 75% of measured ANC above 500 cells/μL) while on G-CSF minus the proportion of successes while on plerixafor, and we prespecified a noninferiority margin of 0.40, which was estimated to be about 50% of the size of the effect of G-CSF versus placebo. For quantifying CIs on differences in success proportions for maintaining the ANC and ALC above predetermined levels on drug, we used a method that accounts for the pairing due to the crossover design and is valid for small samples (36). We switched from a 2-sided test for the primary endpoint (α level 0.05) to a 1-sided test (α level 0.025) for the secondaries, because the primary was prespecified as 2 sided, and the secondary for the ANC endpoint is a noninferiority hypothesis, which is inherently 1 sided. Because the primary outcome was not significant, secondary endpoints were treated as exploratory, and *P* values were not adjusted for multiple comparisons. Exploratory endpoints not prespecified were analyzed by a Wilcoxon’s matched pairs rank test. Additional details are provided in the Supplemental Statistical Analysis Plan.

### Study approval.

The Institutional Review Board of the National Institute of Allergy and Infectious Diseases approved the study. All patients gave written informed consent prior to participation and for the use of clinical photographs.

### Data availability.

Values for all data points in graphs are reported in the Supporting Data Values file. Data are available upon request.

## Author contributions

DHM, DF, MPF, HLM, and PMM designed the study and developed the protocol. CBB and DVP acquired, analyzed, and interpreted HPV genomic data. KRC analyzed bone marrow data. DHM, PMM, PJG, EWC, JJD, MAM, PS, DV, EC, HJK, CB, and JDK acquired and analyzed clinical data. SDR supervised flow cytometry. DBK supervised T cell proliferation and antibody assays. DF, MPF, DHM, and PMM established the statistical analysis plan. DF, MPF, and PMM performed statistical analyses. PMM made the decision to publish the paper. The paper was written mainly by PMM and MPF with contributions from all authors.

## Supplementary Material

Supplemental data

Trial reporting checklists

ICMJE disclosure forms

Supplemental figure 7

Supporting data values

## Figures and Tables

**Table 6:**
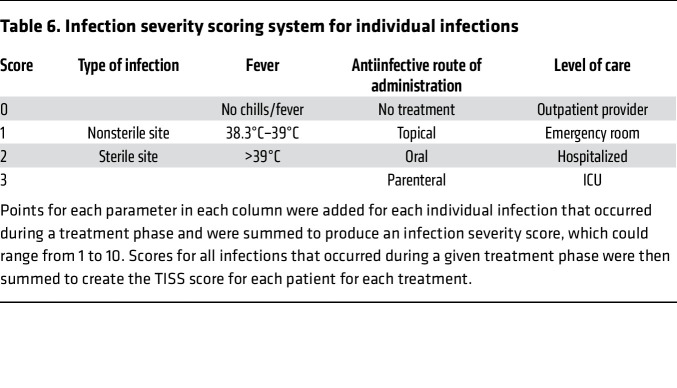
Infection severity scoring system for individual infections

**Table 5:**
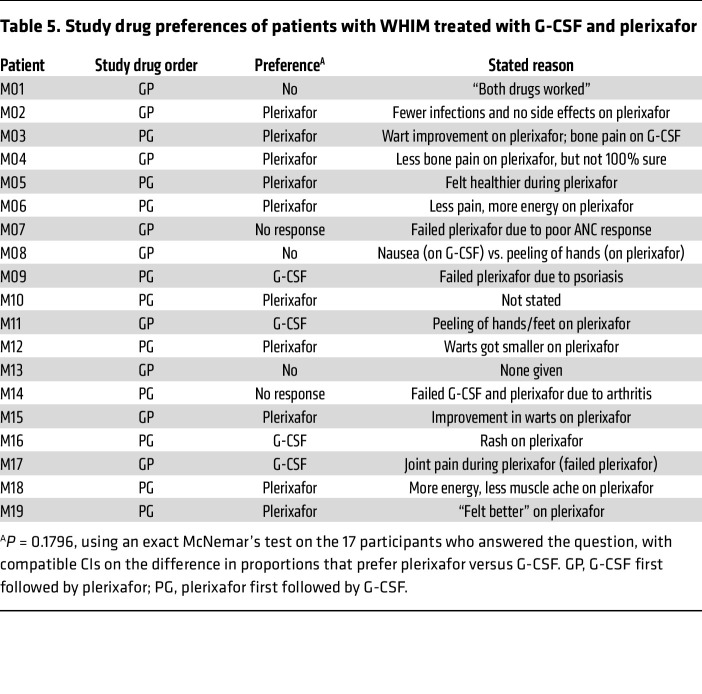
Study drug preferences of patients with WHIM treated with G-CSF and plerixafor

**Table 4:**
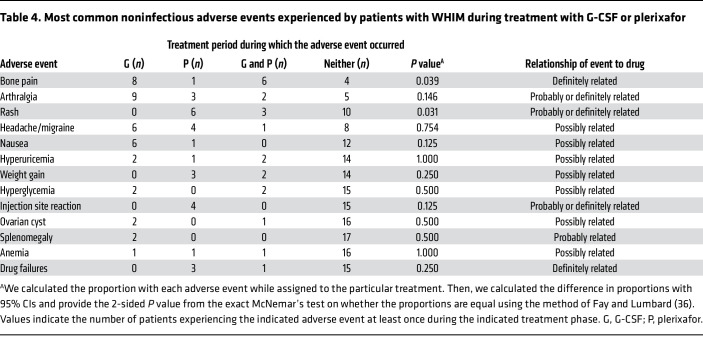
Most common noninfectious adverse events experienced by patients with WHIM during treatment with G-CSF or plerixafor

**Table 3:**
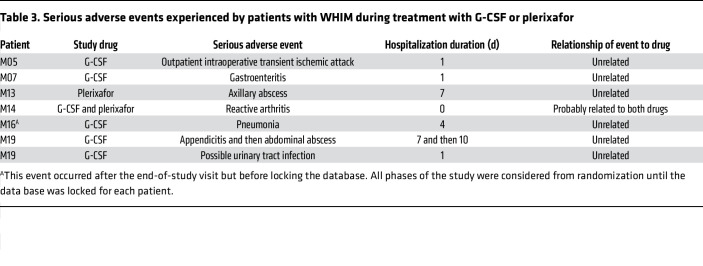
Serious adverse events experienced by patients with WHIM during treatment with G-CSF or plerixafor

**Table 2:**
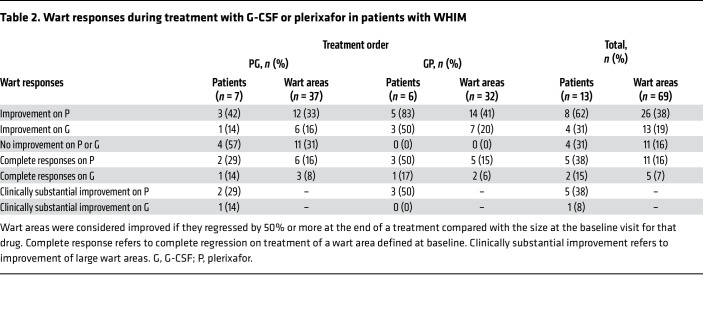
Wart responses during treatment with G-CSF or plerixafor in patients with WHIM

**Table 1:**
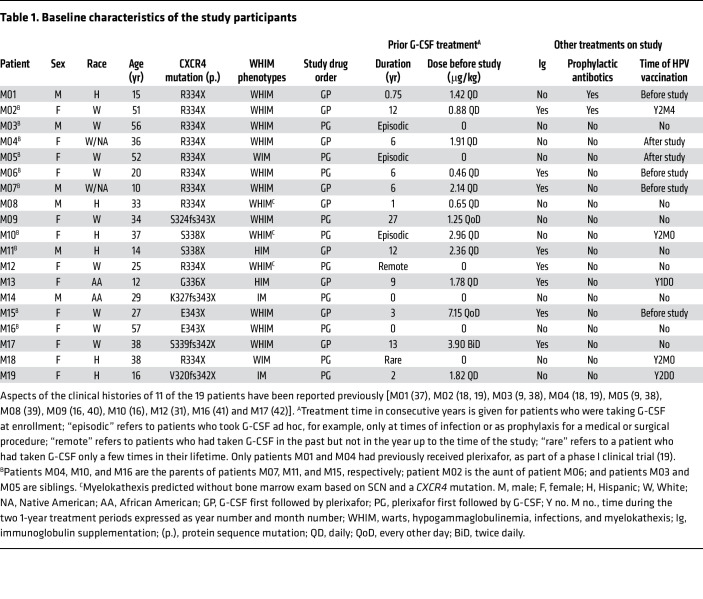
Baseline characteristics of the study participants

## Version 1 08/10/2023

In-Press Preview

## Version 2 10/02/2023

Electronic publication
