# Supplementary material for: A phase III randomized crossover trial of plerixafor versus G-CSF for treatment of WHIM syndrome
Source: J Clin Invest. 2023 Oct 2;133(19):e164918. doi: 10.1172/JCI164918 (PMC10541188; doi:10.1172/JCI164918)
Supplement: Supplemental data [file jci-133-164918-s097.pdf]

Supplementary Appendix

A Phase 3 Randomized Crossover Trial of Plerixafor versus G-CSF for Treatment of WHIM Syndrome
McDermott et al.

This Appendix contains the following items:

1. Supplemental Methods p 2-6
2. Supplemental Results p 6-10
3. Supplemental Figures S1-S7 p 11-26
4. Supplemental Tables S1-S16 p 27-63
5. Statistical Analysis Plan p 64-76
6. Statistical Analysis Plan Supplement, 10/23/20 p 77-86
7. Summary of protocol amendments from Version 1.0 to Version 6.0 p 87-88

Supplemental Methods

Trial Design and Oversight

The Scientific Review Committee of the Laboratory of Molecular Immunology, NIAID (Drs. Joshua Farber, Brian Kelsall and Michail Lionakis), the NIH Institutional Review Board, the NIAID DCR and an independent Data and Safety Monitoring Board provided oversight. A Safety Review and Communication Plan delineated safety and oversight responsibilities of all stakeholders. Monitors contracted with NIAID DCR provided protocol and regulatory compliance. Sanofi-Genzyme (Cambridge, MA) supplied plerixafor under a Research Support Agreement with the NIAID and reviewed the protocol, consent documents and manuscript.

The Statistical Analysis Plan (SAP) was reviewed by the Center for Drug Evaluation and Research, FDA. The method used to generate the random allocation sequence involved a computer generated randomization table that was created prior to study initiation by the NIH Clinical Center Pharmacy Pharmaceutical Development Section (PDS) and maintained solely by them (i.e. the principal investigator, study team, participants and outcomes assessors were masked until study completion and database lock). The random allocation sequence had blocks of 4 so that after every 4 subjects 2 subjects were assigned initial treatment with G-CSF (Neupogen) and 2 were assigned initial treatment with plerixafor. Only specific personnel in the NIH Clinical Center Pharmacy had knowledge of the allocation sequence. The Principal Investigator ordered doses of both medications at all time points in the study after randomization until study completion for each subject and pharmacy personnel delivered unmarked prefilled borosilicate syringes labelled with the subject's name and study drug #1 or #2 corresponding to the ordered doses at the appropriate times. Drs. George J. Grimes and Judy Starling of PDS generated the allocation sequence prior to study initiation. Dr. David H. McDermott (Principal

Investigator) enrolled all the participants and their sequential study enrollment (i.e. date of informed consent) created the randomization order per the computer generated randomization table. All subjects received both drugs labelled only as study drug #1 or #2 as above.

All analyses were conducted by the investigators. P.M.M. and M.P.F wrote the manuscript, with contributions from all authors who agreed to publish and attest to data accuracy and completeness and trial adherence to the protocol.

Patients

Participants were required to be willing to travel to the NIH-Clinical Center for scheduled study visits, and to have a local health care provider able to implement interim study assessments.

Treatment

Study subject visits to the NIH-CC were scheduled at the following times: 1) the start of the ~0.5-4-month screening phase for evaluation of patient compliance with protocol requirements as well as for dose-finding and evaluation of tolerance of open label twice daily G-CSF; 2) the start of each 2-month study drug equilibration phase (defined as day 0) for baseline assessments; 3) the start of each one-year treatment phase (defined as month 0); 4) every 4 months during each treatment phase (designated as months 4, 8 and 12 of treatment); and 5) ~6 months after the end of treatment visit for an End-of-Study visit. Comprehensive health and safety assessments were conducted at each visit, including pregnancy status in females of reproductive age. Between scheduled NIH visits, subjects visited their local provider for management of WHIM syndrome phenotypes and any new interim medical problems according to best medical practice. Participants maintained a Memory Aid in which they recorded daily treatments and any new symptoms to assist in the collection of information about all adverse events and medications.

Since the study has a crossover design, we did not hypothesize that there would be a difference during follow-up between G-CSF-plerixafor and plerixafor-G-CSF treatment orders, since both arms were offered the same amount of plerixafor during the study (albeit in different order) and were treated similarly after the trial was over, i.e. offered G-CSF, but not plerixafor. Therefore, the protocol did not include such a pre-specified follow-up period for analysis of study endpoints.

Since WHIM syndrome is a type of SCN and since G-CSF is the standard of care for SCN, G-CSF was selected as the comparator drug. It is important to note, however, that although the use of G-CSF in WHIM syndrome is common practice in the United States and is our standard practice, the safety and clinical efficacy of G-CSF in WHIM syndrome has never been tested directly. Including a third placebo arm in the present study was judged not to be feasible due to the rareness of the disease.

Both drugs were compounded in unmarked clear borosilicate sterile syringes under Good Manufacturing Practice conditions by either the Pharmaceutical Development Section of the NIH-CC Pharmacy or Integrity Bio (Camarillo, CA), were kept refrigerated until use, and were periodically tested to assure sterility and drug stability.

Endpoints and Assessments

For calculation of the TISS score, non-sterile site infections were defined as those which occur in areas of the body routinely exposed to and colonized by microorganisms such as the oral cavity, bronchioles and upper respiratory tract, nasopharynx, vagina, GI tract and skin, whereas sterile sites were the lower respiratory tract, blood, muscle, bone, joints, urinary bladder and other typically sterile locations. Fever refers to the maximum oral temperature recorded during the infection. Anti-infective treatment is scored based on the highest level of treatment, for example,

an intravenous antibiotic that is changed to oral would score as a 3, the highest score. Similarly, hospitalization refers to the highest level of care received at any point during the infection.

Immunophenotyping data were acquired on a BD FACSLyricTM flow cytometer (BD Biosciences, Franklin Lakes, NJ) and the results were analyzed using FCS Express 6 Flow Cytometry Clinical Edition (De NovoTM Software, Pasadena, CA). Gating for lymphocyte subsets was performed on $CD45^{+}CD14^{-}$ cells using forward and side scatter. $CD4^{+}$ T cells were defined as $CD3^{+}CD4^{+}$, $CD8^{+}$ T cells as $CD3^{+}CD8^{+}$, NK cells as $CD3^{-}CD56^{+}$, NKT cells as $CD3^{+}CD56^{+}$, effector memory $CD4^{+}$ as $CD3^{+}CD4^{+}CD62L^{-}CD45RA^{-}$, effector memory $CD8^{+}$ as $CD3^{+}CD8^{+}CD62L^{-}CD45RA^{-}$, central memory $CD4^{+}$ as $CD3^{+}CD4^{+}CD62L^{+}CD45RA^{-}$, and B cells as $CD19^{+}$.

For lymphocyte proliferation assessments, freshly isolated and cryopreserved PBMC were both studied with similar results. Cryopreserved PBMCs were first thawed and rested for 2 hours at 37°C in the presence of 30 U/mL of DNase (10,000 U/mL. Roche Cat. # 04716728001) in media containing RPMI 1640 (Invitrogen, Cat. #21807), 25 mM Hepes (Invitrogen, Cat. # 15630-080), 1X Pen/Strep-L-glutamine (100X Gibco BRL, Cat. #10378-016), and 20% human AB serum (Gemini, Cat. #100-512). Cells were washed 2 times, counted on a MUSE Cell Analyzer (Millipore Sigma, Burlington, MA) and adjusted to a viable $1x10^{6}$/ml in media containing RPMI 1640 (Invitrogen, Cat. #21807), 25 mM Hepes (Invitrogen, Cat. # 15630-080), 1X Pen/Strep-L-glutamine (100X Gibco BRL, Cat. #10378-016), 0.1 mM non-essential amino acids (Gibco BRL, Cat. #11140-050), 1 mM sodium pyruvate (Gibco BRL, Cat. #11360-070), 50 μM 2-mercaptoethanol (Sigma, Cat. #M7522) and 10% human AB serum (Gemini, Cat. #100-512). Rested PBMC were plated into 96-well round bottom plates at 100,000 cells/well in triplicates. PBMC were stimulated with either 100 U/ml IL-2 (Teceleukin), 2.5 μg/mL

phytohemagglutinin (PHA-P, Sigma, Cat. #L0917), 25 μg/mL concanavalin A (Sigma, Cat. #C5275), or 1.25 μg/mL pokeweed mitogen (Sigma, Cat. #L9379) for 3 days or with tetanus toxoid (Millipore, Cat. #582231), *Candida albicans* (Greer, Cat. #XPLM73X1A2), anti-CD3 (ThermoFisher, Cat, #16-0037-81) or an irradiated mixed lymphocyte pool for 6 days at 37°C in 5% CO_2. Cells cultured under similar conditions without any stimulation served as the negative control. After the stated incubation period, 20 μCi/mL [^{3}H]-thymidine (Perkin Elmer, Cat. #NET-027) was added to each well and incubated for 4 hours at 37°C in 5% CO_2 and then the plates were frozen at -20°C overnight. The plates were thawed and the cells harvested onto filtermats (Perkin-Elmer, Cat. #1450-421) and dried several hours. The filters were then placed into sample bags (Perkin Elmer, Cat. #1450-432) containing scintillation fluid (Perkin Elmer, Cat. #1205-440) and counted with a beta scintillation counter (MicroBeta Trilux, Perkin-Elmer). Proliferation responses were calculated as a Stimulation Index (SI), as determined by the mean ratio of antigen/mitogen-stimulated counts per minute (cpm) over background cpm. Two frozen normal donor controls with known responsive values to PHA at day 3 incubation and tetanus toxoid at day 6 incubation were run in parallel with each assay to assure quality control of the assay results. Freshly isolated PBMCs were tested for each patient and a healthy donor phlebotomized on the same day.

Supplemental Results

Pulmonary Function

Although evaluation of lung function was not a prespecified study endpoint in the protocol, we did collect data from many of the participants as indicated for clinical care (Supplemental Figure S1). All 19 patients had computerized tomography (CT) of the chest that established a pre-study baseline of lung radiographic findings. Six of the 19 (M07, M08, M13, M14, M17 and M19),

including 3 of the 5 children (M07, M13 and M19), had normal lungs by CT criteria and M07 and M08 also had normal pulmonary function test (PFT) results; the other 4 had normal flow-volume loops and very mild diffusion defects. Lacking a clinical indication, we did not obtain follow up PFTs on these 6 patients at the End-of-Treatment visits for either G-CSF or plerixafor. Of the 13 patients with lung pathology by CT criteria, 10 had bronchiectasis with varying degrees of severity and 3 had other abnormalities (mostly focal scarring and nodules). The 3 (M09, M10 and M12) with 'other' CT abnormalities (scarring and/or nodules) all had normal flow-volume loops, and M09 and M12 had mild-moderate diffusion defects. Only M12 had follow-up PFTs, and only at the end of plerixafor treatment given first, which revealed a slight decrease in diffusion capacity of the lung for carbon monoxide (DLCO). Of the 10 patients with CT-defined bronchiectasis, M04, M06 and M15 had only a 'mild' abnormality; M04 and M06 had normal PFTs at baseline which were not repeated during the two End-of-Treatment visits. Patient M06 had not had baseline PFTs performed.

Of the 7 CT-defined bronchiectasis patients with abnormal baseline PFTs, 5 had moderate CT abnormalities and mild-moderate PFT abnormalities. Of these 5, patients M03, M05 and M16 had minor changes after treatment with plerixafor given first but were not retested after G-CSF treatment given second; patient M18 had a minor decrease in FEV1 (forced expiratory volume in one second) and FVC (forced vital capacity) after treatment with G-CSF given second, but not after plerixafor given first; and patient M11 had a small increase in FEV1 after G-CSF treatment given first but was not retested after plerixafor given second.

The remaining two bronchiectasis patients with abnormal PFTs had the most severe obstruction, restriction and diffusion defects at pre-study baseline, and both had dyspnea during a 6-minute walk test (data not shown). One of these, patient M01, was a 15-year-old boy with

scoliosis who had had cardiac surgery as an infant for repair of Tetralogy of Fallot, a known cardiovascular phenotype affecting ~5% of WHIM patients. Despite his markedly abnormal pulmonary function, he was an equestrian athlete competing at the international level. The only change in his PFTs after G-CSF treatment was a small increase in DLCO; he was not retested after plerixafor treatment. The second patient, M02, had the most severe bronchiectasis in the study and was receiving supplemental oxygen at home. She did not show a significant change in her PFTs after either G-CSF given first or plerixafor given second.

HPV Distribution

Forty-five HPV types were each identified only once, each in only one sample from a single patient, and 17 HPV types were identified multiple times (Supplemental Table S14). Of the 17 HPVs identified multiple times, 11 were identified twice; one was identified 3 times (HPV80); 3 were identified 4 times (HPV3, 28 and 164); one was identified 6 times (HPV57); and one was identified 7 times (HPV27). Nine of the 17 HPVs identified more than once were identified either in multiple warts from the same patient or in two relatives. Regarding the relatives, HPVs 27, 28, 57 and 80 were all found in both patients M03 and M05, who are siblings. Of these, only HPV57 was found in other WHIM patients. HPV3 and 164 were both found in patients M15 and M16, a daughter and mother, as well as in one other unrelated WHIM patient.

The number of different HPVs isolated from a sample ranged from only one (HPV38) in the forehead warts of patient M01 at the baseline visit to a high of 15 in a mixed sample of skin and genital warts in patient M15 at the baseline visit. HPV diversity correlated poorly with the HPV disease burden of the patient. For example, patient M12 had extensive warts on her hands and feet, yet only two HPVs were identified, HPV57 and 136, and HPV57 accounted for 99% of the HPV reads from the sample. In other samples containing multiple HPVs, a dominant HPV

type was also apparent. Likewise, HPV27 represented 97% of the HPV reads in a swab of multiple wart areas from patient M03 at the end of plerixafor treatment that also contained small amounts of HPV28 and HPV80.

Only 2 of the HPV types found in the HPV-9 vaccine were identified in the baseline survey, HPV6 and 18. Both were found in a genital swab from patient M05, who had not been vaccinated and had a long history of known high risk HPV-associated genital disease. Four patients had received an HPV vaccination series with Gardasil 9 (Merck) before the study, two males (M01 and M07) and two females (M06 and M15). Both M07 and M15 had condyloma accuminata at the time of vaccination. Five other patients, all females, received HPV vaccination with Gardasil 9 (Merck) during the study (M02, M10, M13, M18 and M19). Three had evaluable warts at baseline (M02, M10 and M18). M10 and M18 were vaccinated at the start of the second study drug, and in both cases this was G-CSF. M18 had cutaneous warts that did not change after vaccination and M10 had genital warts but refused examination after vaccination.   M02 received vaccination with Gardasil 9 (Merck) at month 4 of study drug two, which was plerixafor, and had regression of some warts during this period.

Quality of Life Assessment

Quality of life was assessed at baseline and at the end of each treatment period using the Short Form-36 question (SF-36) health survey version 2 questionnaire (Supplemental Table S4).
To obviate carryover effects, Physical and Mental Composite Scores relative to the general population (PCS and MCS, respectively) were compared at baseline to the end of treatment period one and the differences were compared for plerixafor versus G-CSF. At baseline, PCS was the ‘same or better’ as the general population for 9 patients, ‘well-below’ the general population for 5 patients and ‘below’ the general population for 4 patients. Patient M14 had not

completed the questionnaire. After one year of drug treatment, there were two drug failures, and of the 17 patients with data 7 patients had a change in PCS. Three of these 7 patients had a worse score at this visit than at baseline; one had received G-CSF and 2 had received plerixafor. Of the 4 patients who had a better score at this visit than at baseline, one had received G-CSF and 3 had received plerixafor.

At baseline, MCS was the 'same or better' as the general population for 15 patients, 'well-below' the general population for one patient and 'below' the general population for 2 patients. Patient M14, who failed on both arms of the study because of arthritis, had not completed the questionnaire. After one year of drug treatment, there were two drug failures. Of the 17 patients with data, 2 patients had a change in MCS; one patient treated with G-CSF had an improved score and one patient treated with plerixafor had a worse score.

At the end of treatment period 2, PCS and MCS quality of life scores were only available for 11 and 10 patients, respectively, because of drug failures during the period and because 6 patients had not filled out the questionnaire at the end of this period. For the 11 patients with PCS data, there were 4 changes at the end of period 2 compared to the end of period one; one patient treated with plerixafor had an improved score and of 3 patients treated with G-CSF, two had improved scores and one had a worse score. There were also 2 changes in MCS at the end of period 2 compared to the end of period one; one patient treated with plerixafor had a worse score and one patient treated with G-CSF had an improved score. Overall, quality of life was not significantly different between the two arms of the study.

Supplemental Figures

Supplemental Figure S1. Pulmonary abnormalities in WHIM patients treated with plerixafor and G-CSF for one-year. Chest CT scans were obtained at the baseline visit for all 19 study subjects who were divided into three groups by lung radiographic findings, as defined in the inset. Eighteen patients underwent the pulmonary function tests indicated on the y-axis at the baseline visit and a subset of those had repeat evaluations for clinical care at the end of treatment 1 and 2. The red dashed line is the lower limit of the normal range for each test. The treatment is color-coded, as defined in the inset. Black lines connect results from different time points for the same patient. Isolated, unconnected data points are the result of missing data from other timepoints. EoT, end of treatment; DLCO, diffusion capacity of the lung for carbon monoxide; FEV1, forced expiratory volume in one second; FVC, forced vital capacity.

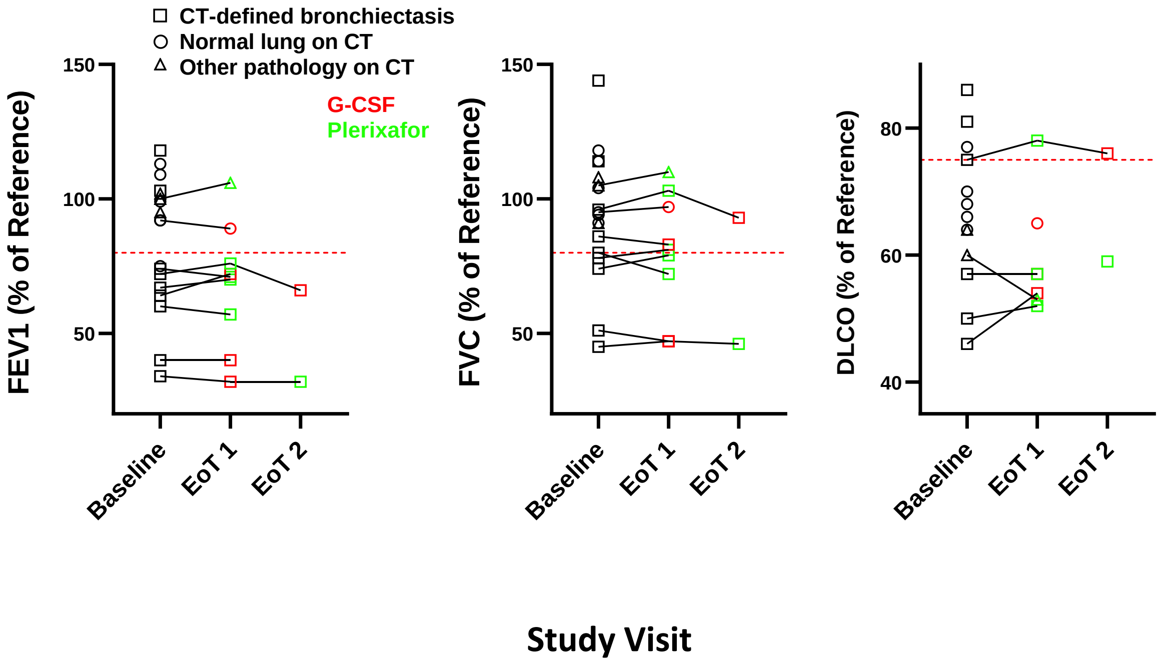

Supplemental Figure S2. Relationship of maximal study drug doses used during the treatment phases to maintain the ANC above 500 cells/microliter. Drug failures are color-coded according to the reason for failure, as shown in the inset. Adverse events are detailed in Table 3. Abbreviations: GP, G-CSF given first/plerixafor given second; PG, plerixafor given first/G-CSF given second; ANC, absolute neutrophil count in peripheral blood.

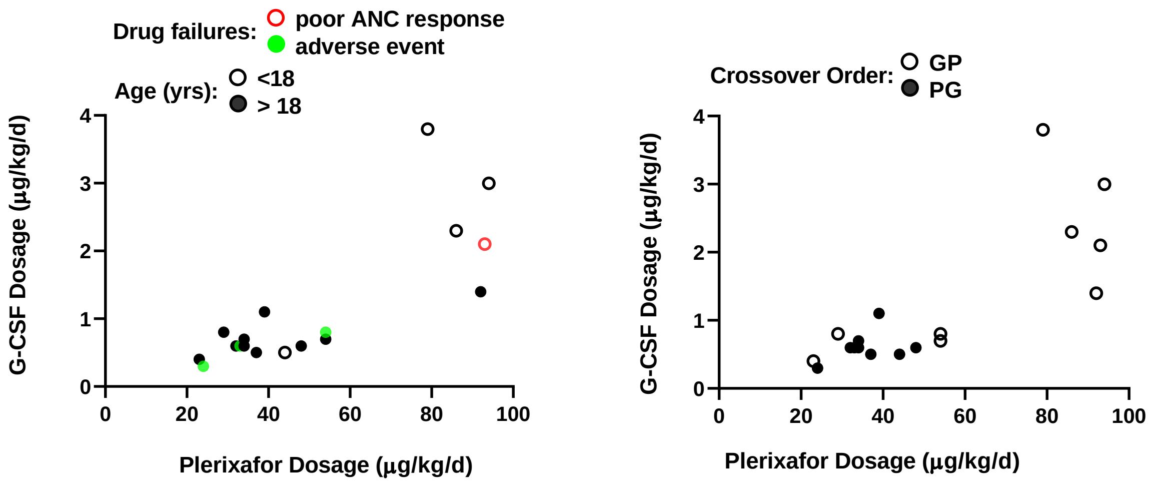

Supplemental Figure S3. Incidence of infection in WHIM patients treated with G-CSF and plerixafor. A) Incidence of infection stratified by site. Data are the number of infections per subject per treatment period (P, plerixafor; G, G-CSF) in the indicated compartments for the 15 subjects who did not fail in any period. The p-values are from Wilcoxon signed rank tests. B) Incidence of infection stratified by time on drug. Each symbol represents the number of infections for a single patient during the indicated treatment phase and includes all 18 patients with data from at least one treatment phase (excluding patient M14 who failed during both equilibration phases). Horizontal bars represent the mean +/- SEM for each distribution.

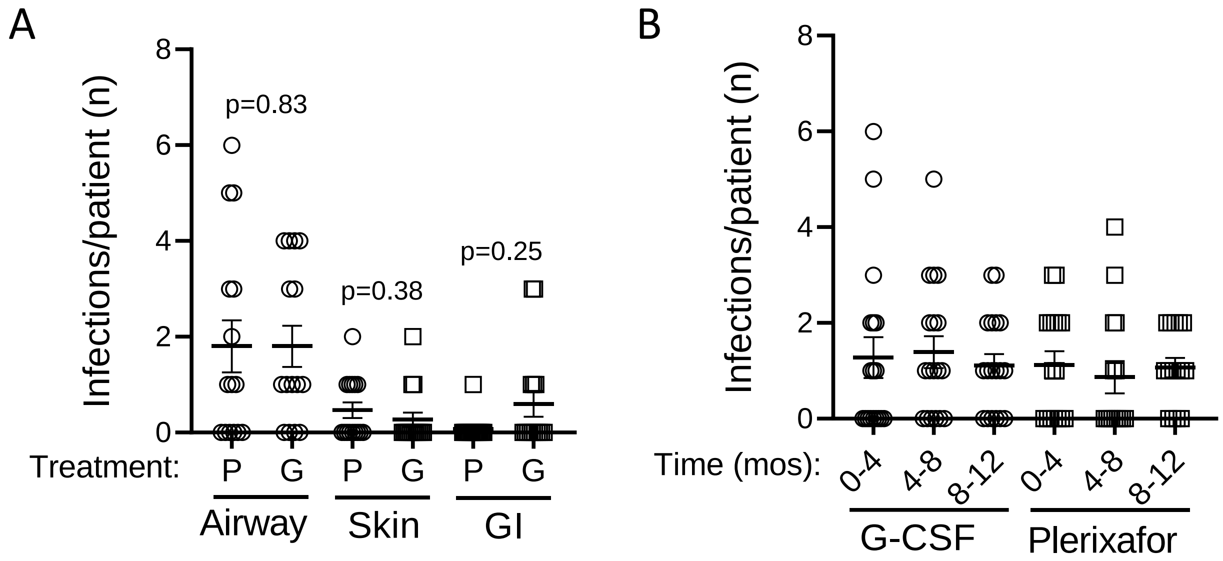

Supplemental Figure S4. Hematologic responses to G-CSF and plerixafor treatment in WHIM patients. Each line graph for the time course data represents data from a single patient. Each symbol in the scatter plot graph represents a single patient value. In panels A and B, data are graphed separately for ANC and ALC, respectively, just before a drug dose was given (trough) and from ~2-3 hours after a dose was given (post-dose). Time course data for all other parameters include only post-dose values. Time zero for all time course graphs is the baseline value obtained for each patient after the first 2-day washout of G-CSF before administering the first masked study drug and is replotted in the scatter plot graphs and labeled 'baseline'. G-CSF and plerixafor values in the scatter plot graphs are the final values obtained at the end of each treatment arm. Dashed red horizontal lines in panels A and B demarcate the predefined minimum target ANC and ALC levels, respectively, for defining hematologic success of each study drug, as scored in Figure 4B and C. Dashed red horizontal lines in all other panels demarcate the normal range for adults at the NIH Clinical Center for each parameter. The time on each drug is demarcated at the top of each time course graph. In panels D-H, the immunophenotype of each subset is given at the upper left. In panel I, the bottom graph shows the naïve CD4$^+$ T cell data as a function of patient age; the top of each color marks the value observed at baseline (black) and at the end of the G-CSF (green) and plerixafor (red) treatment periods. p values shown at the top of the scatter plots are for the drug data comparison and were determined by a Wilcoxon matched pairs rank test.

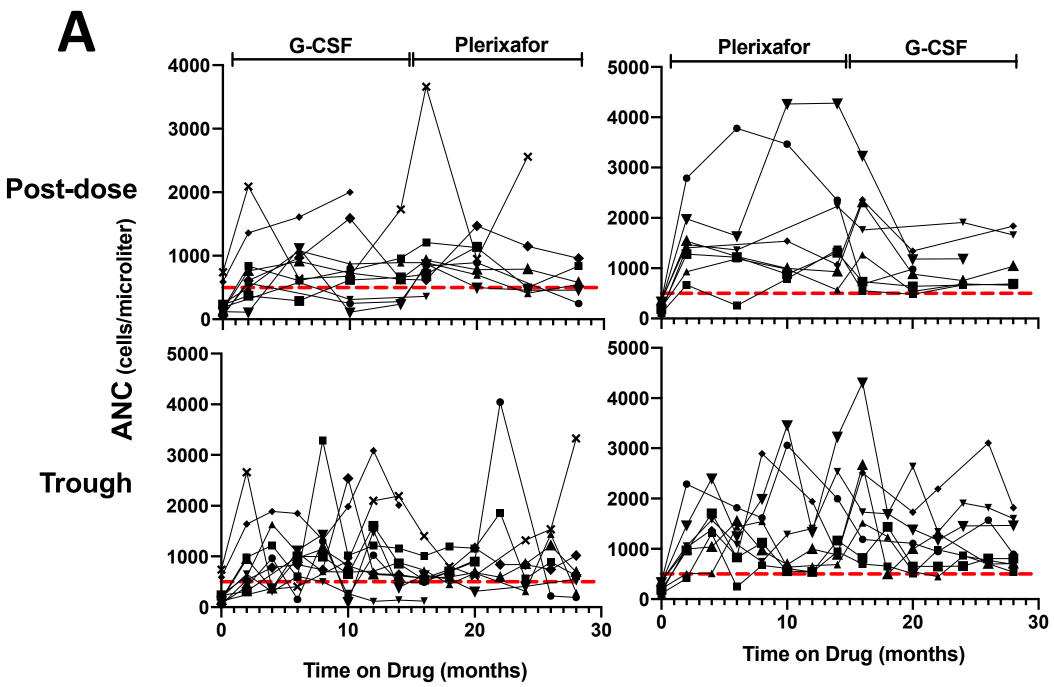

Supplemental Figure S4 (continued).

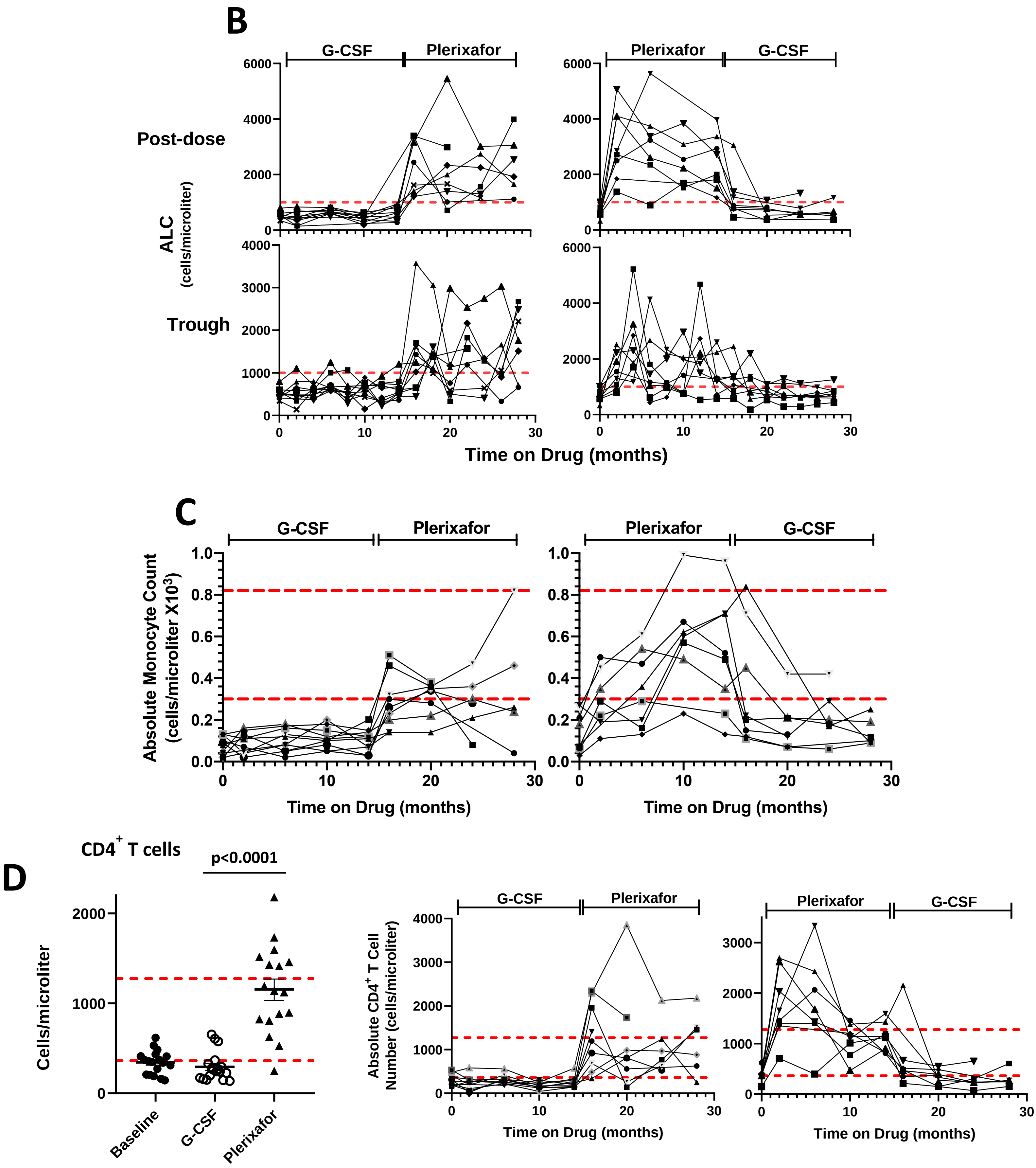

E

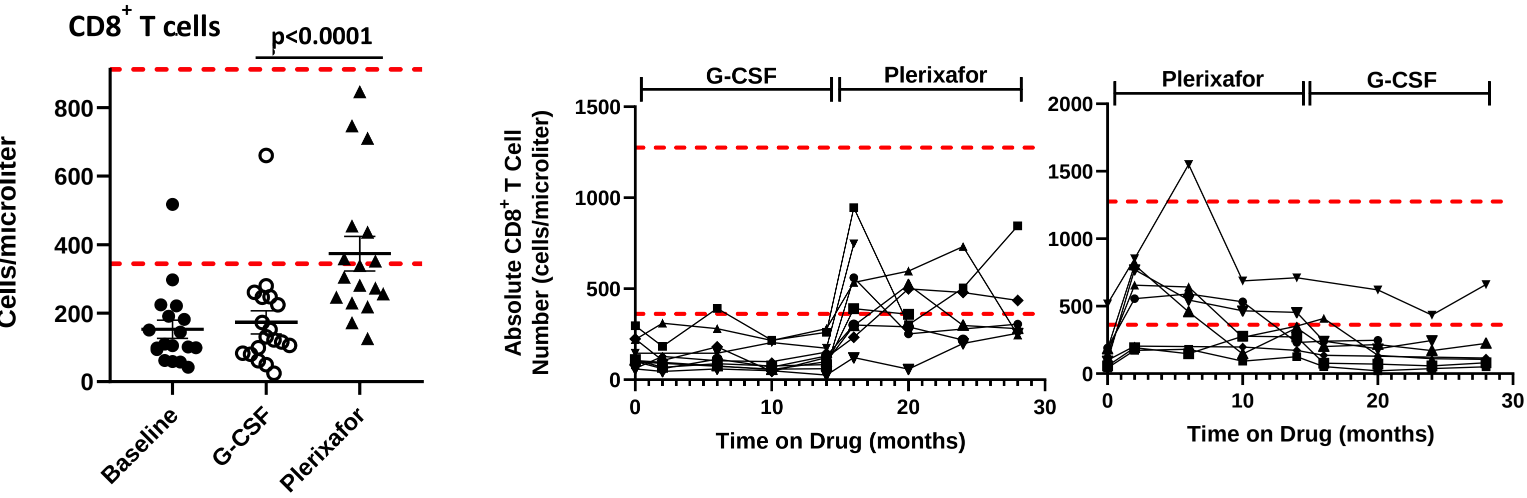
CD8+ T cells
p<0.0001
Cells/microliter
Baseline
G-CSF
Plerixafor
G-CSF
Plerixafor
Absolute CD8+ T Cell Number (cells/microliter)
Time on Drug (months)
Plerixafor
G-CSF
Time on Drug (months)

F

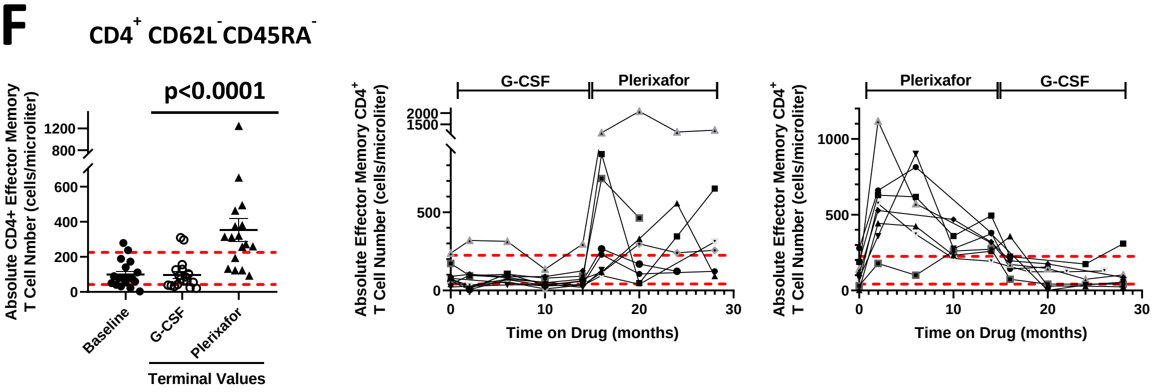
CD4+ CD62L- CD45RA-
p<0.0001
Absolute CD4+ Effector Memory T Cell Nmber (cells/microliter)
Baseline
G-CSF
Plerixafor
Terminal Values
G-CSF
Plerixafor
Absolute Effector Memory CD4+ T Cell Number (cells/microliter)
Time on Drug (months)
Plerixafor
G-CSF
Absolute Effector Memory CD4+ T Cell Number (cells/microliter)
Time on Drug (months)

G

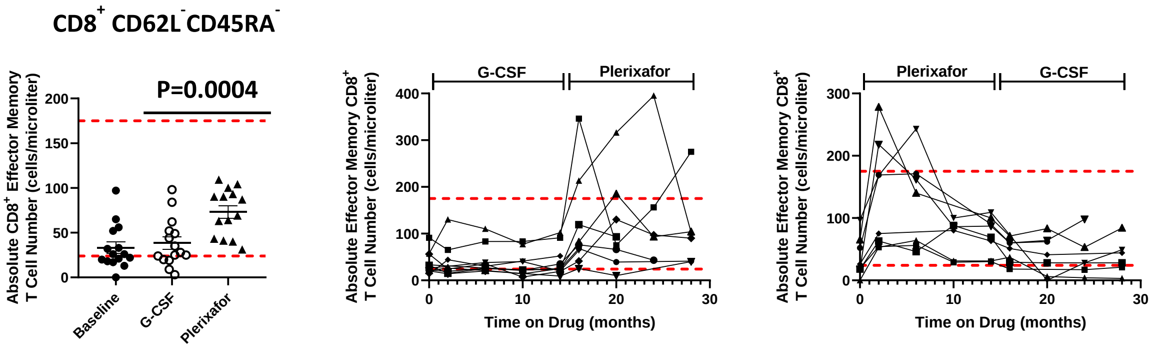
CD8+ CD62L- CD45RA-
P=0.0004
Absolute CD8+ Effector Memory T Cell Number (cells/microliter)
Baseline
G-CSF
Plerixafor
G-CSF
Plerixafor
Absolute Effector Memory CD8+ T Cell Number (cells/microliter)
Time on Drug (months)
Plerixafor
G-CSF
Absolute Effector Memory CD8+ T Cell Number (cells/microliter)
Time on Drug (months)

H

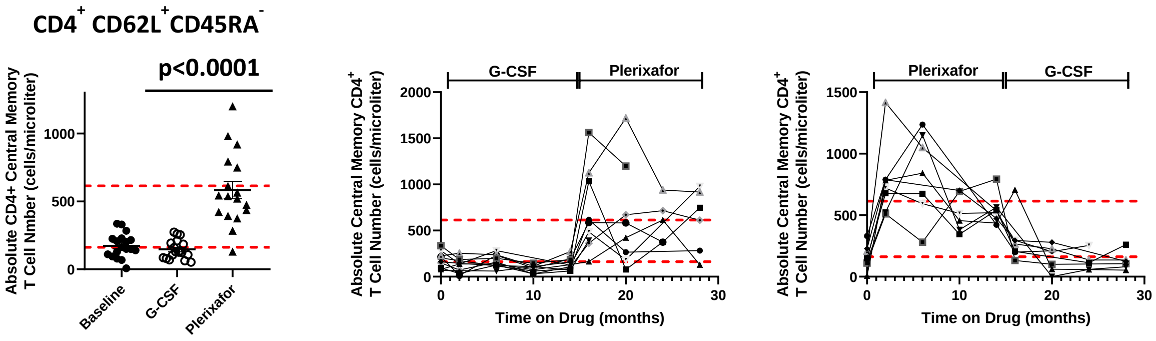
CD4+ CD62L+ CD45RA-
p<0.0001
Absolute CD4+ Central Memory T Cell Nmber (cells/microliter)
Baseline
G-CSF
Plerixafor
G-CSF
Plerixafor
Absolute Central Memory CD4+ T Cell Number (cells/microliter)
Time on Drug (months)
Plerixafor
G-CSF
Absolute Central Memory CD4+ T Cell Number (cells/microliter)
Time on Drug (months)

Supplemental Figure S4 (continued)

I

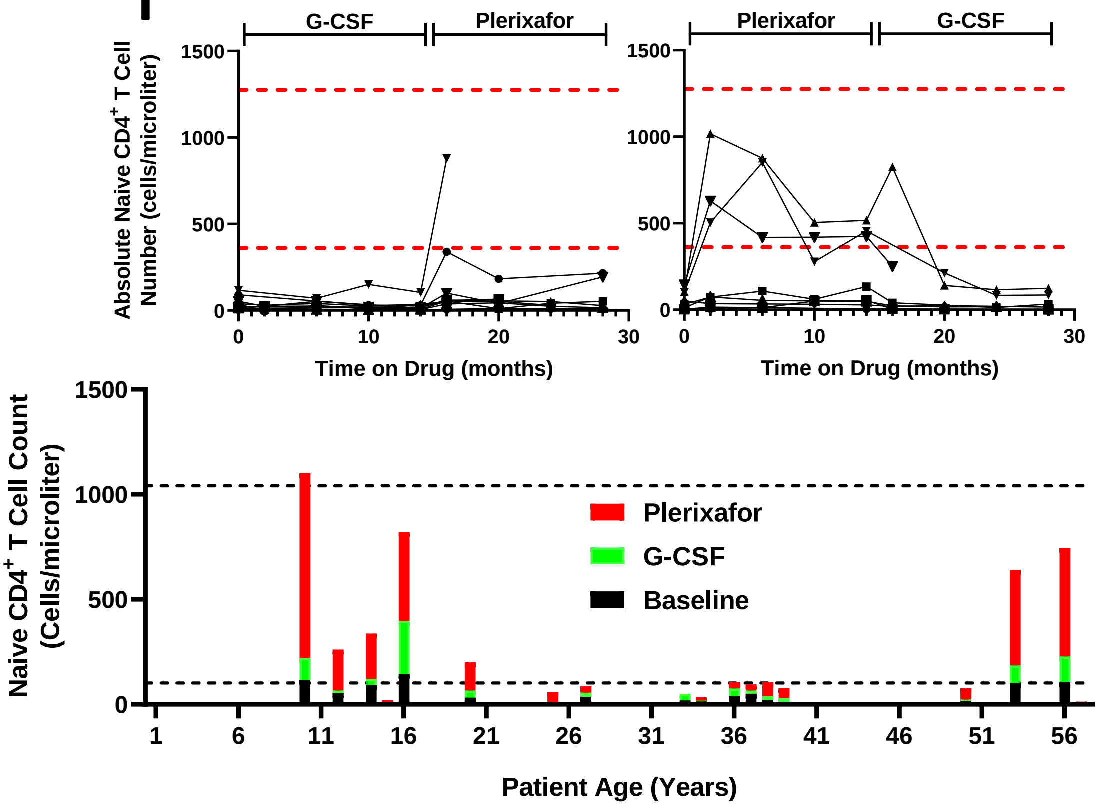

J

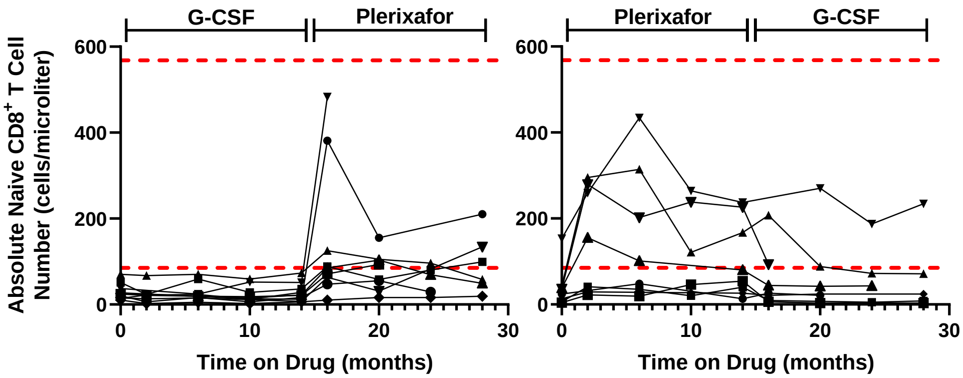

Supplemental Figure S4 (continued).

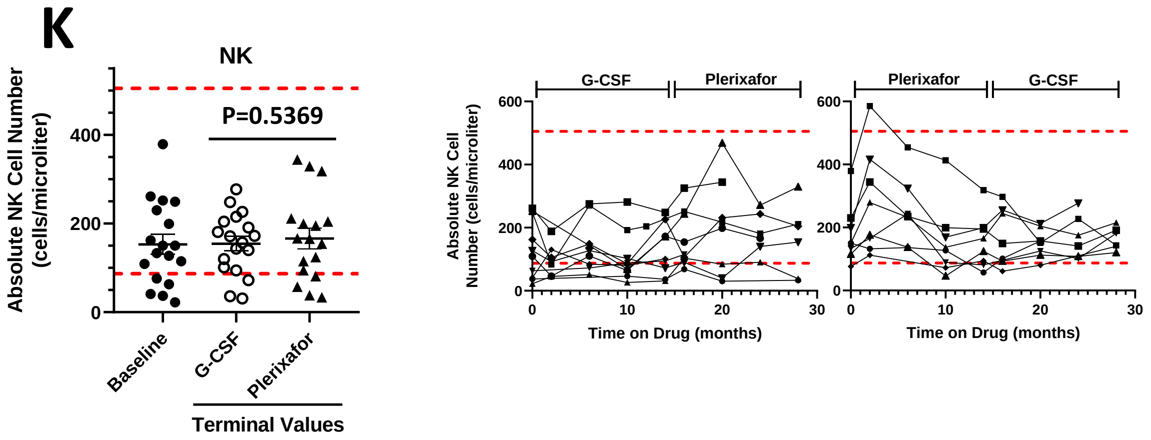

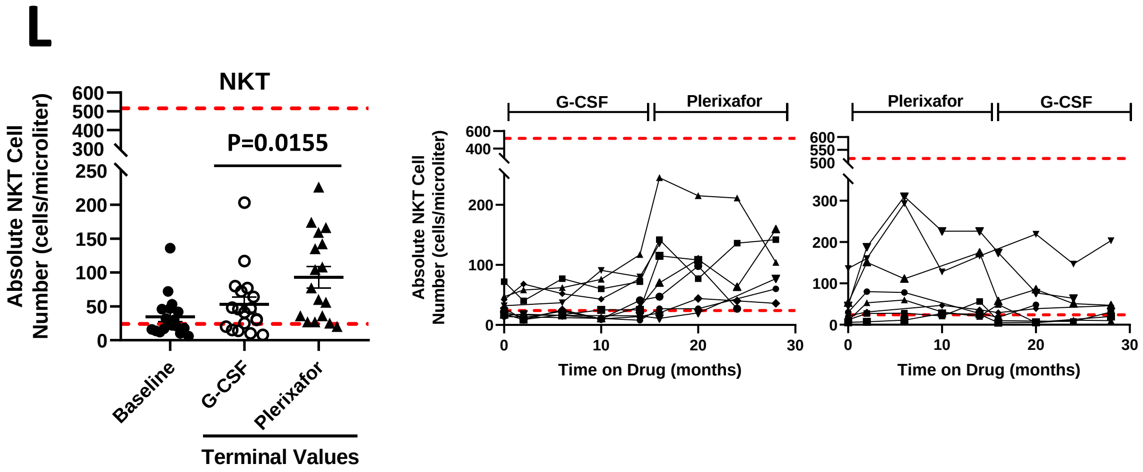

Supplemental Figure S4 (continued).

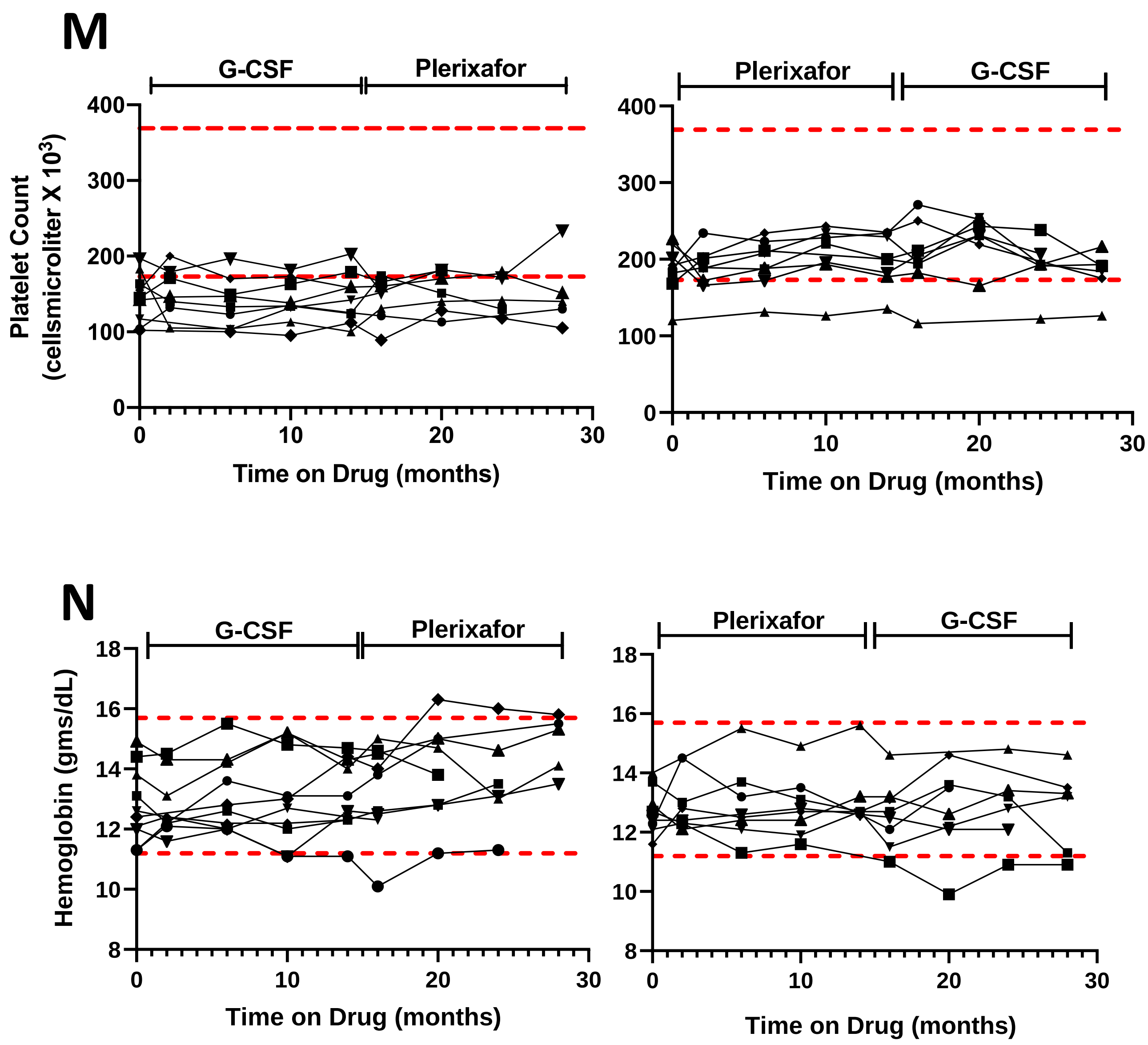

Supplemental Figure S5. Effects of G-CSF and plerixafor treatment on the circulating absolute neutrophil and lymphocyte counts in WHIM patients. Data obtained at blood draws scheduled at the NIH Clinical Center during the treatment phases just before a drug dose was given (trough) and from ~2-3 hours after a dose was given (post-dose) are presented together for each subset as separate graphs for each patient designated at the top of each pair of graphs. Time zero, colored in plum, is the baseline value obtained for each patient after the first 2-day washout of G-CSF at the end of the screening phase before administering the first masked study drug at the start of the first equilibration phase. Dashed horizontal lines, red for ANC and green for ALC, demarcate the predefined minimum target cell number for defining hematologic success, as scored in Figures 5B and C, respectively. The time on each drug is demarcated at the top of each graph, with prematurely terminated treatment (drug failure) colored red. Patient M14, who received only one week of each study drug and therefore did not generate time course data, is not included.

Supplemental Figure S5.

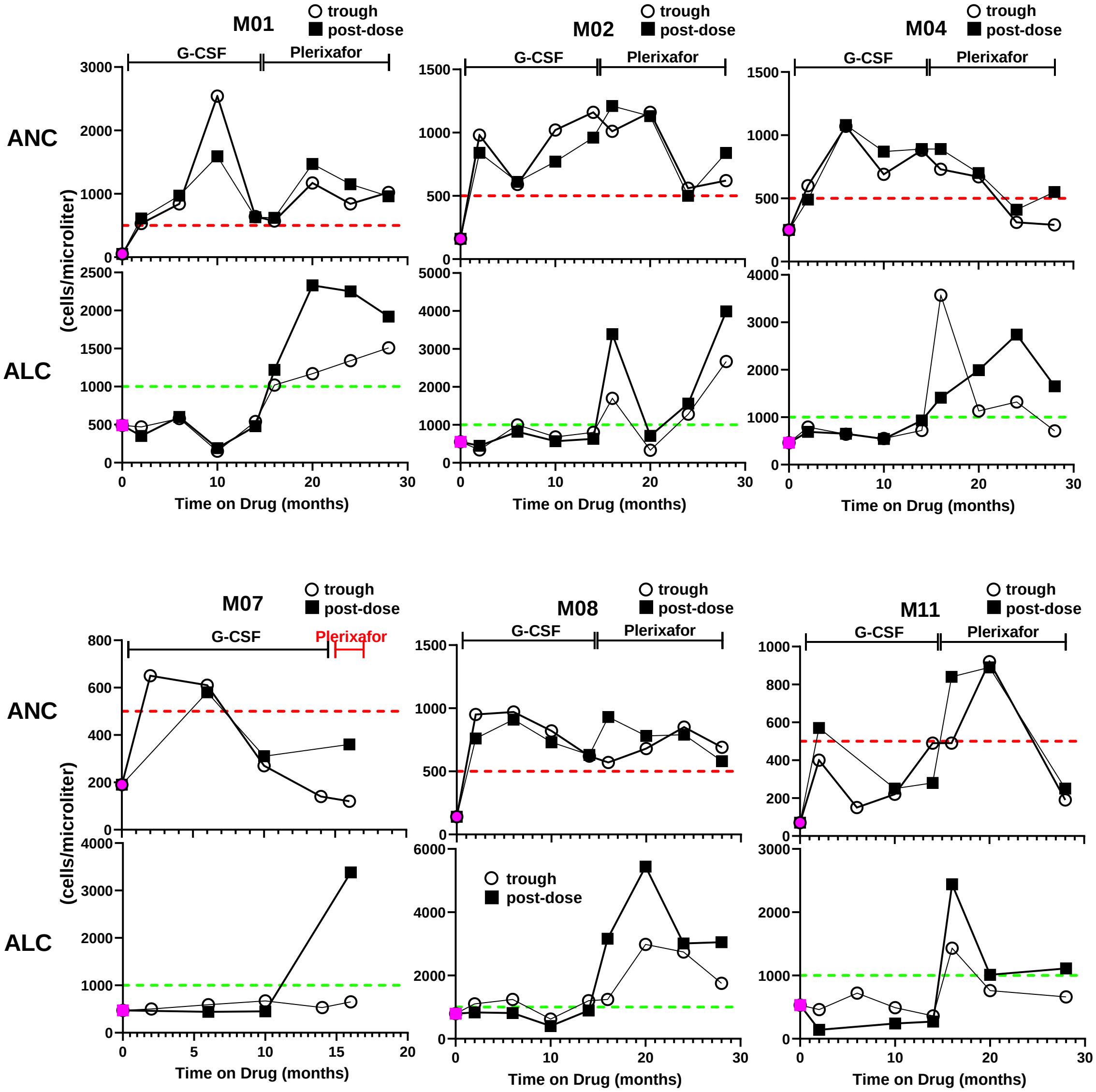

Supplemental Figure S5.

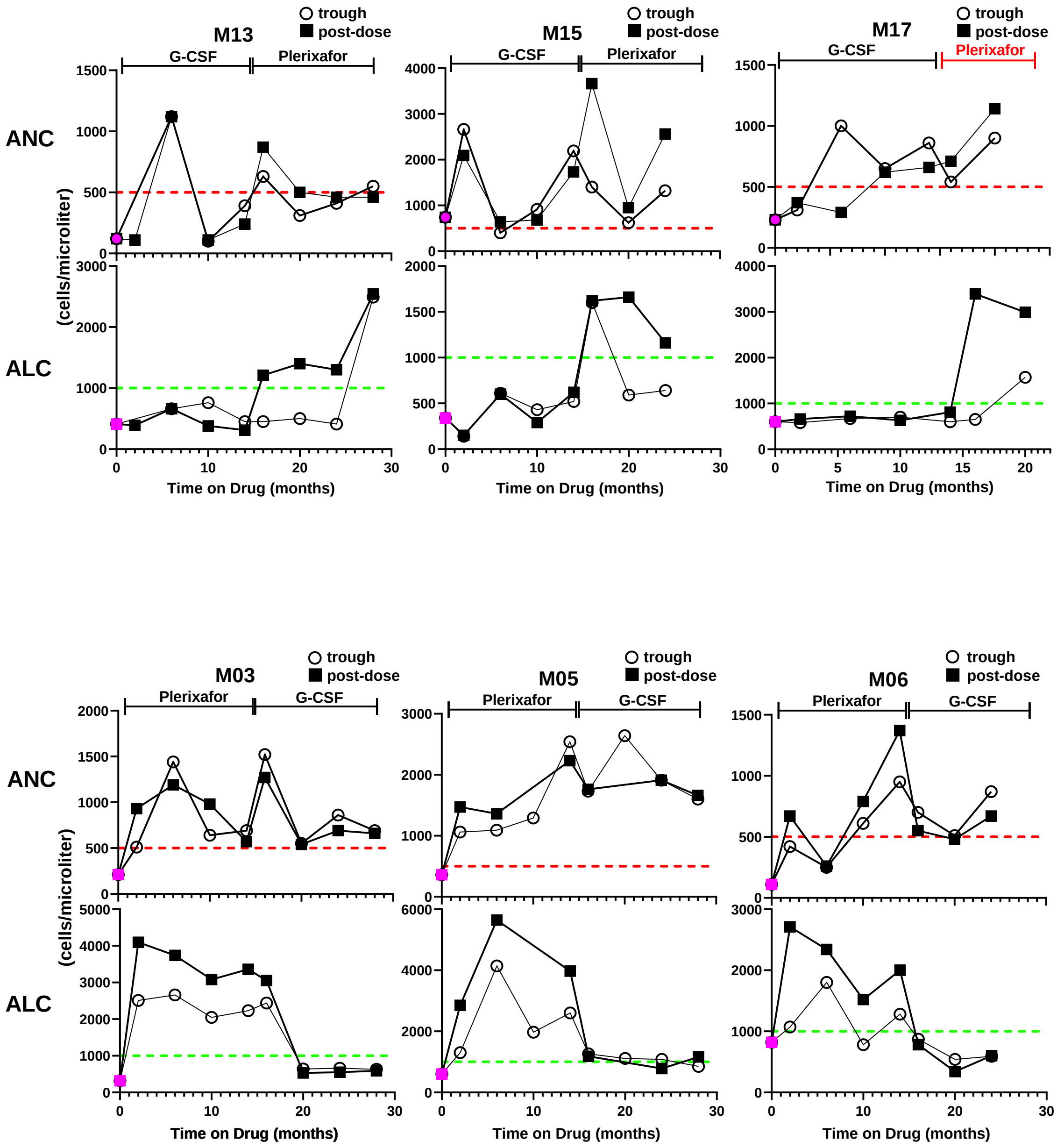

Supplemental Figure S5.

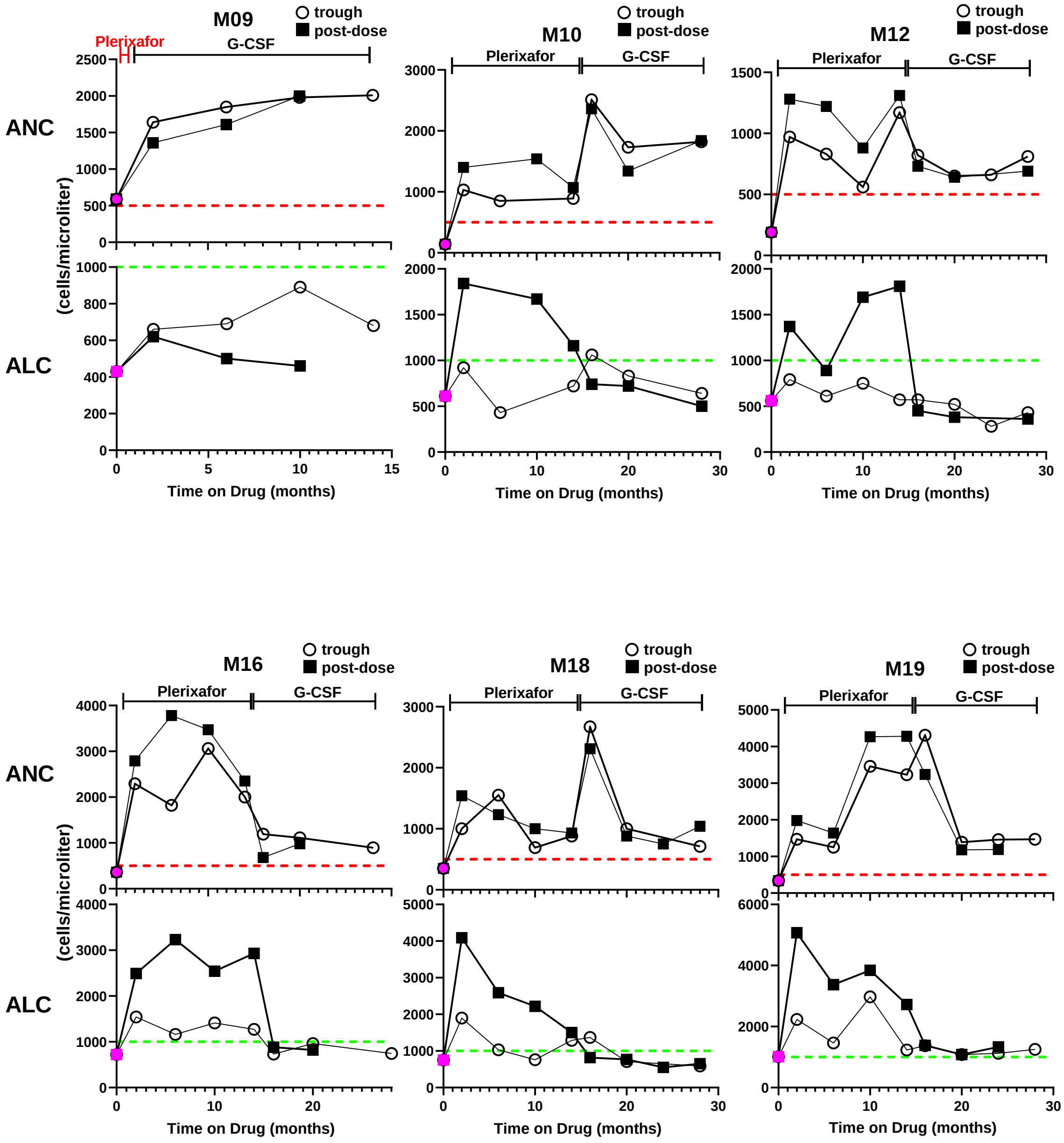

Supplemental Figure S6. T cell proliferation responses in WHIM patients treated with G-CSF and plerixafor. 100,000 freshly isolated PBMCs from the study subjects (Pt) and a healthy control subject (C) were stimulated for 3 days with (+) or without (-) IL-2 (100 units/ml), phytohemagglutinin (PHA, 2.5 μg/ml), Concanavalin A (ConA, 25 μg/ml) or Pokeweed Mitogen (PWM, 1.25 μg/ml), and for 6 days in a mixed lymphocyte reaction (MLR, 50,000 cells/well) or with Tetanus toxoid (Tt, 1 μg/ml) or *Candida albicans* antigen (20 μg/ml), and proliferation was measured at the endpoint by ^{3}H incorporation as counts per minute (CPM). Each data point is the average of 3 determinations for a single patient at the indicated visit for the indicated stimulus. Data for the G-CSF and plerixafor visit samples after stimulation were analyzed by a Wilcoxon matched pairs rank test for 9-10 pairs that had complete data for all three study visits; however, all data were plotted. ns, not significant; C, healthy control subject; Pt, patient; B, baseline visit after randomization at day 0 of the first equilibration phase; G, final visit during the G-CSF treatment phase; P, final visit during the plerixafor treatment phase; No stim, no stimulus.

Supplemental Figure S6 (continued)

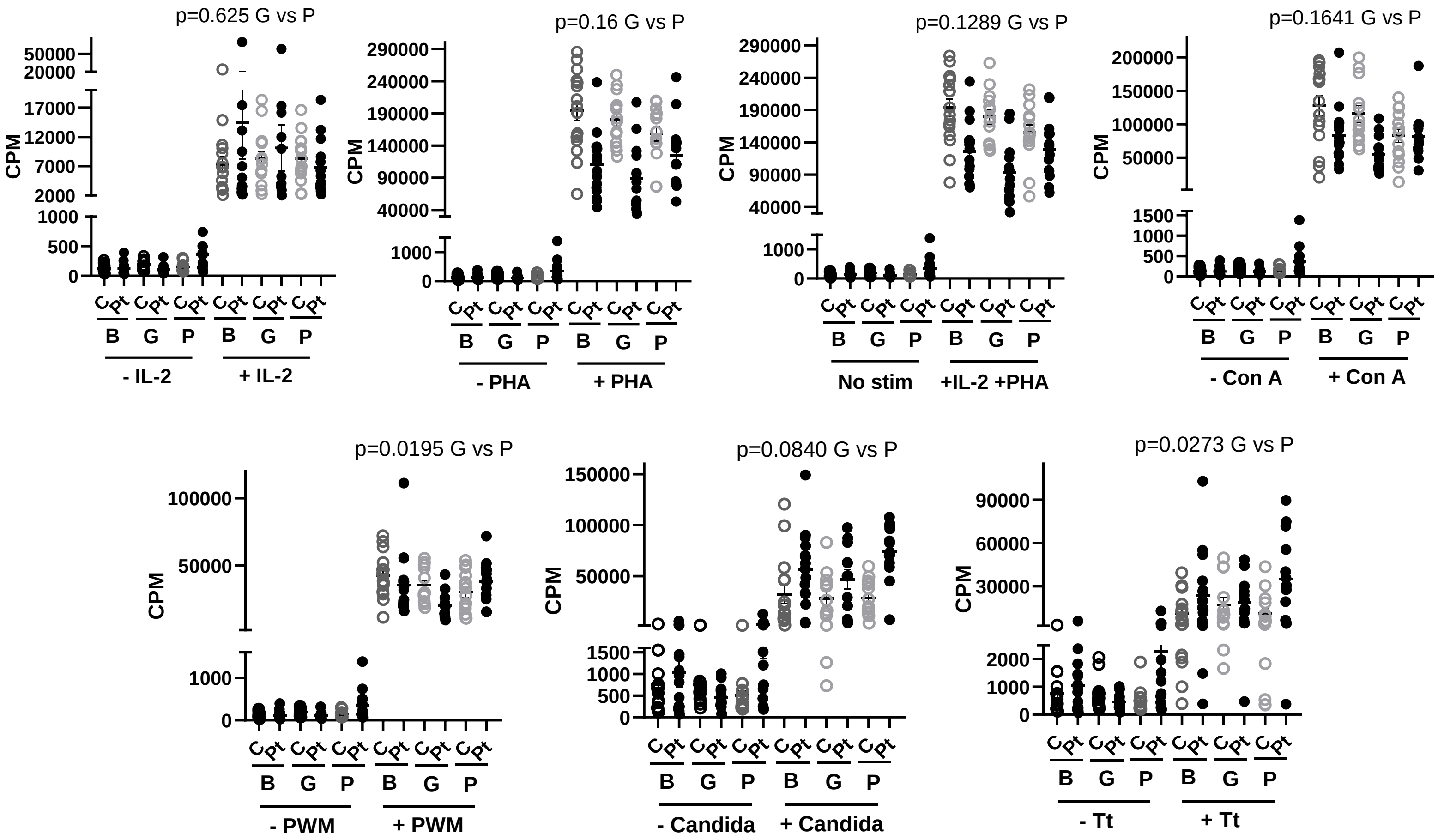

Supplemental Figure S7. Plerixafor versus G-CSF effects on wart burden in WHIM patients. Comprehensive images of warts at the baseline visits, interim drug treatment visits and, where available, before and after the trial are shown for patients with evaluable warts. The photographs are labeled with dates if taken before or after the study and by the study period for those taken within the study. d0 is the first day drug was administered in the first equilibration phase of the study. M0 is month zero or the first day of the indicated treatment phase. M4, M8 and M12 refer to visits at approximately months 4, 8 and 12 of the indicated treatment phase. P, plerixafor; G, G-CSF. Patient M10 had only genital warts and refused photography. The files holding these photographs are too large to be included in this Supplementary Appendix and are submitted in a separate supplemental file. Analyses and summary assessments for wart changes are detailed in Table 2 and Supplemental Tables S7-S13.

Supplemental Tables

Supplemental Table S1. Patient characteristics at the time of enrollment stratified by randomization group (PG or GP, where G is G-CSF; P is plerixafor).

		Study Drug Order		
		PG (n=10)	GP (n=9)	Total
Sex	Male	2	4	6
	Female	8	5	13
Age	<18	1	4	5
	>18	9	5	14
Race	C	6	3	9
	H	3	3	6
	AA	1	1	2
	C/NA	0	2	2
Genotype	R334X	5	5	10
	Other	5	4	9
WHIM Phenotypes	WHIM	6	7	13
	WIM	2	0	2
	HIM	0	2	2
	IM	2	0	2
Treatment	G-CSF	3	9	12
	Plerixafor	0	0	0
	Ig	2	6	8
	Antibx	0	2	2
Prior HPV Vaccination		3	6	9

Abbreviations: H, Hispanic; C, Caucasian; AA, African American; WHIM, Warts-Hypogammaglobulinemia-Infections-Myelokathexis; HPV, human papillomavirus; Ig, immunoglobulin supplementation; Antibx, prophylactic antibiotics

Supplemental Table S2. *CXCR4* mutations in study patients. Shown are the heterozygous mutations in the region of the open reading frame encoding the carboxy-terminus of CXCR4. Open reading frame nucleotide and protein sequence changes are designated based on numbering from the reference sequence NM_003467.3 published by the United States National Library of Medicine (translation start site=1).

	***CXCR4* mutation**	
**Patient**	**Nucleotide**	**Protein**
**M01**	1000 C→T	R334X
**M02**	1000 C→T	R334X
**M03**	1000 C→T	R334X
**M04**	1000 C→T	R334X
**M05**	1000 C→T	R334X
**M06**	1000 C→T	R334X
**M07**	1000 C→T	R334X
**M08**	1000 C→T	R334X
**M09**	969_970insG	S324fs343X
**M10**	1013 C→G	S338X
**M11**	1013 C→G	S338X
**M12**	1000 C→T	R334X
**M13**	1006 G→T	G336X
**M14**	979_980insG	K327fs343X
**M15**	1027 G→T	E343X
**M16**	1027 G→T	E343X
**M17**	1015_1016delTC	S339fs342X
**M18**	1000 C→T	R334X
**M19**	959_960delTG	V320fs342X

Abbreviations: C, cytosine; T, thymidine; G, guanine; ins, insertion; del, deletion; R, arginine; X, stop codon; S, serine, K, lysine; E, glutamate; V, valine; fs, frame shift; ins, insertion; del, deletion

Supplemental Table S3. Summary of study subject infection history prior to enrollment. Severity code: 0, no infections; 1+, non-recurrent infection; 2+, recurrent infection; 3+, recurrent infection with documented evidence of end organ damage (e.g. bronchiectasis, hearing loss, tooth loss, blindness). Note that only patients M01 and M02 were receiving prophylactic antibiotics during the study.

	Pre-study Treatments			Pre-study Infection Experience by Site and Severity							
**Patient**	**G-CSF[a]**	**Ig**	**h/o Prophyl. Antibx[b]**	**Sinus**	**Middle Ear**	**Lung**	**Skin**	**GU**	**Blood**	**Dental**	**Other**
**M01**	yes	no	yes	2+	2+	3+	0	0	1+	0	*T. gondii* chorioretinitis, endocarditis
**M02**	yes	yes	yes	3+	3+	3+	1+	1+	0	2+	
**M03**	episodic	no	no	2+	3+	3+	2+	0	1+	3+	
**M04**	yes	no	yes	2+	2+	3+	2+	1+	0	3+	Septic arthritis, osteomyelitis, meningitis
**M05**	episodic	no	yes	2+	3+	3+	1+	2+	1+	3+	Sepsis
**M06**	yes	yes	yes	3+	3+	3+	2+	2+	0	2+	parotiditis
**M07**	yes	yes	yes	2+	3+	3+	2+	0	1+	2+	Septic arthritis, Neonatal sepsis, Tinea capitis
**M08**	yes	no	no	1+	3+	2+	2+	0	0	2+	
**M09**	yes	no	yes	3+	2+	2+	1+	2+	0	2+	
**M10**	episodic	no	yes	0	1+	3+	0	1+	0	3+	
**M11**	yes	yes	yes	2+	2+	3+	2+	1+	1+	0	Neonatal sepsis, MCV, HSV gingivostomatitis
**M12**	remote	yes	no	2+	2+	2+	0	2+	1+	0	Brain abscess, osteomyelitis, endocarditis
**M13**	yes	yes	yes	0	2+	2+	2+	0	1+	1+	Enterococcal Sepsis, Tinea capitis
**M14**	no	no	no	1+	2+	2+	3+	0	1+	1+	Sepsis

**M15**	yes	yes	no	2+	2+	2+	3+	0	0	1+	HSV lymphadenitis
**M16**	no	remote	no	2+	3+	3+	2+	0	0	3+	meningitis
**M17**	yes	yes	no	3+	2+	2+	3+	2+	1+	2+	Sepsis
**M18**	rare	no	no	3+	2+	3+	2+	0	0	3+	HBV, HSV dermatitis
**M19**	yes	no	no	0	2+	1+	2+	1+	0	1+	MCV

[a]'Yes' and 'no' indicate patients who were or were not being treated with the indicated agents by their health care providers at the time of signing the informed consent. Pre-study dosages of G-CSF at enrollment are provided in Supplementary Table S5. 'Episodic' refers to patients who would take G-CSF only at times of infection; 'remote' refers to patients who had taken G-CSF in the past but not in the year up to the time of the study; 'rare' refers to a patient who had taken G-CSF once or only a few times in their lifetime.

[b]Abbreviations: MCV, molluscum contagiosum virus; HSV, herpes simplex virus; HBV, hepatitis B virus; GU, genitourinary tract; Ig, immunoglobulin supplementation; h/o Prophyl Abx, history of prophylactic antibiotic treatment.

Supplemental Table S4. Quality of life of WHIM patients at baseline visit and after treatment with G-CSF (G) and plerixafor (P). Physical and mental composite scores (PCS and MCS) were calculated from responses to the sf36 version 2 questionnaire using proprietary software and compared to the general population as follows: SB, same or better; WB, well below; B, below. ND, no data available; F, drug failure; Y(1 or 2)M12, treatment phase year (1 or 2), visit month 12; baseline, visit at the start of equilibration phase 1. Cells highlighted in red and green indicate a worse and a better score, respectively, compared to the assessment obtained at the previous visit.

						PCS			MCS		
**Patient**	**Sex**	**Age (yrs)**	**CXCR4 Mutation**	**WHIM Phenotypes**	**Study Drug Order**	Baseline	Y1M12	Y2M12	Baseline	Y1M12	Y2M12
**M01**	M	15	R334X	WHIM	GP	SB	SB	SB	SB	SB	SB
**M02**	F	51	R334X	WHIM	GP	WB	WB	SB	SB	SB	B
**M03**	M	56	R334X	WHIM	PG	SB	SB	SB	SB	SB	SB
**M04**	F	36	R334X	WHIM	GP	SB	B	B	SB	SB	SB
**M05**	F	52	R334X	WIM	PG	SB	SB	SB	SB	SB	SB
**M06**	F	20	R334X	WHIM	PG	WB	B	B	SB	SB	SB
**M07**	M	10	R334X	WHIM	GP	B	B	F	B	SB	F
**M08**	M	33	R334X	WHIM	GP	SB	SB	SB	SB	SB	SB
**M09**	F	34	G323fs	WHIM	PG	WB	F	SB	B	F	SB
**M10**	F	37	S338X	WHIM	PG	SB	B	ND	SB	SB	ND
**M11**	M	14	S338X	HIM	GP	WB	WB	ND	SB	SB	ND
**M12**	F	25	R334X	WHIM	PG	WB	WB	B	SB	SB	SB
**M13**	F	12	G336X	HIM	GP	SB	SB	SB	SB	SB	SB
**M14**	M	29	K327fs	IM	PG	ND	F	F	ND	F	F
**M15**	F	27	E343X	WHIM	GP	B	SB	ND	SB	SB	ND
**M16**	F	57	E343X	WHIM	PG	SB	B	ND	SB	WB	ND
**M17**	F	38	S339fs	WHIM	GP	SB	SB	ND	WB	WB	F
**M18**	F	38	R334X	WIM	PG	B	SB	WB	SB	SB	SB
**M19**	F	16	V320fs	IM	PG	B	SB	ND	SB	SB	ND

Supplemental Table S5. G-CSF doses at enrollment and at the start of the screening and equilibration phases. Syringes were prefilled in each of 5 predefined doses of G-CSF as detailed in the Methods section. Patients not receiving G-CSF at enrollment were initially given unmasked syringes containing either of the two lowest G-CSF syringe sizes (0.05 or 0.075 mls) at the start of the screening phase. The initial unmasked screening phase dose assignment for patients already taking G-CSF at enrollment was a judgment based on patient weight, the pre-study dose, the ANC associated with the pre-study dose and assessment of pre-study G-CSF-related side effects. The initial masked equilibration phase G-CSF dose was specified based on the ANC and side effects observed on unmasked G-CSF during the screening phase. Any changes to the initial dose were based on a response resulting in an ANC <500 cells/microliter and/or side effects and are graphed in Figure 3. Patients <18 years of age are identified by red text.

			Initial G-CSF Dose				
			Pre-study[a]	Screening Phase		Equilibration Phase	
**Patient**	**Drug Order**	**Initial Weight (kg)**	**µg/kg/d**	**syringe size (ml)[b]**	**µg/kg/d[d]**	**syringe size (ml)[b]**	**µg/kg/d[d]**
M03	PG[c]	74	0	0.075	0.6	0.05	0.4
M05	PG	50	0	0.05	0.6	0.05	0.6
M06	PG	66	0.46	0.05	0.46	0.05	0.46
M09	PG	48	1.25	0.05	0.62	0.05	0.62
M10	PG	41	0	0.05	0.76	0.05	0.76
M12	PG	76	0	0.05	0.42	0.05	0.42
M14	PG	100	0	0.05	0.3	0.05	0.3
M16	PG	76	0	0.05	0.38	0.05	0.38
M18	PG	70	0	0.05	0.44	0.075	0.58
M19	PG	55	1.82	0.12	1.3	0.05	0.52
M01	GP	32	1.42	0.05	0.94	0.12	2.26
M02	GP	87	0.88	0.12	0.88	0.12	0.84
M04	GP	52	1.91	0.12	1.38	0.12	1.38

M07	GP	35	2.14	0.12	2.06	0.12	2.06
M08	GP	115	0.65	0.075	0.4	0.075	0.4
M11	GP	38	2.36	0.05	0.76	0.12	1.88
M13	GP	34	1.78	0.05	0.9	0.12	2.14
M15	GP	66	7.2	0.12	1.1	0.05	0.46
M17	GP	91	3.90	0.12	0.78	0.12	0.78

[a] The listed pre-study dose is the dose the patient was receiving at the time of enrollment. Patients M09 and M15 were receiving the listed daily dose every other day; patient M17 received the listed dose in two divided doses.

[b] The concentration of G-CSF in each syringe is 300 micrograms/ml.

[c] P, plerixafor; G, G-CSF

[d] The listed dose is the total daily dose. i.e. Half the listed dose was given twice a day.

Supplemental Table S6. Distribution of infections that occurred on study during treatment with G-CSF (G) or plerixafor (P). Patient number designations are abbreviated from the M# format used elsewhere in the paper. Infection designations were those given by the diagnosing provider. 'H' indicates an infection resulting in hospitalization. na, not applicable; HSV, Herpes Simplex virus; URTI, upper respiratory tract infection; UTI, urinary tract infection. 'F' designates a drug failure. Three patients failed plerixafor due to side effects (M09 and M17) or failure to reach the prespecified ANC level during the equilibration phase (M07). M07 and M09 failed during the plerixafor equilibration phase; M17 failed at month 6 of the 12-month plerixafor treatment phase. Patient M14 failed during both the plerixafor and G-CSF equilibration phases. Thus, 18 patients received a full 12-month treatment course of G-CSF, whereas 15 patients received a full 12-month treatment course of plerixafor and one patient received a 6-month course of plerixafor.

		Number of Infections on G-CSF or Plerixafor																				
	**Drug Order**	**GP**	**GP**	**PG**	**GP**	**PG**	**PG**	**GP**	**GP**	**PG**	**PG**	**GP**	**PG**	**GP**	**PG**	**GP**	**PG**	**GP**	**PG**	**PG**	**Total**	
	**Patient M#**	**1**	**2**	**3**	**4**	**5**	**6**	**7**	**8**	**9**	**10**	**11**	**12**	**13**	**14**	**15**	**16**	**17**	**18**	**19**	**G**	**P**
**AIRWAY**	Otitis Media	2				1			3	2									1	1	6	4
	URTI					2		2	3[d]		1	1[d]/ 2[g]	1	1		2	1/1	3/3		1/3	12	15
	Sinusitis					2	1			1	1						1	1			3	4
	Flu-like illness	1[a]	1										1					1			3	1
	Acute Bronchitis		4/2									1	1			3	1[h]	2[h.n]	2[n]		10	6
	Pneumonia				*1*							2[d,h,i]				1			1		5	0
	**TOTAL**	3/0	4/3	0/0	1/0	0/5	1/0	2/F	0/6	3/F	1/1	4/2	3/0	0/1	F/F	1/5	1/3	7/3 (F)	4/0	4/1	**39**	**30**
**SKIN**	Skin infection													1/*1*[k] *H*		2			1[l]/ 1[l]		4	2
	Tinea				1[b]			1[c]				1[j]		1[c]			1				1	4
	**TOTAL**	0/0	0/0	0/0	0/1	0/0	0/0	1/F	0/0	0/F	0/0	0/1	0/0	1/2	F/F	2/0	0/1	0/0 (F)	1/1	0/0	**5**	**6**
**UTI**	UTI						0/1		0/1									2/0	1/0	*1H /0*	**4**	**2**
**GI**	Gastroenteritis		3					1H	1[e]/1			1					1[m]			1	8	1
	Appendicitis																			*1H*	1	0
	Abdominal abscess																			*1H*	1	0
	**TOTAL**	0/0	3/0	0/0	0/0	0/0	0/0	1/F	1/1	0/F	0/0	1/0	0/0	0/0	F/F	0/0	1/0	0/0 (F)	0/0	3/0	**10**	**1**
**Dental**	Tooth infection				1/0		0/1				0/2										**1**	**3**
	Conjunctivitis					1														2	2	1
	Thrush		1																		1	0
	Dacryocystitis		1																		1	0

**OTHER**	Otitis externa							2													2	0
	Herpes labialis										1			1		1[l]					2	1
	Vaginitis										1[f]									1	2	0
	Fever																	1			1	0
	**TOTAL**	0/0	2/0	0/0	0/0	0/1	0/0	2/0	0/0	0/0	1/1	0/0	0/0	1/0	0/0	1/0	0/0	1/0	0/0	3/0	**11**	**2**
																			**Totals**		**70**	**44**
**TISS**	**P**	**0**	**12**	**0**	**2**	**18**	**10**	**F**	**27**	**F**	**14**	**6**	**0**	**15**	**F**	**15**	**9**	**F**	**2**	**1**	**--**	**--**
	**G**	**14**	**22**	**0**	**11**	**0**	**4**	**17**	**3**	**11**	**4**	**17**	**14**	**5**	**F**	**14**	**6**	**26**	**20**	**36**	**--**	**--**
**# of Inf.**	**P**	**0**	**3**	**0**	**1**	**6**	**2**	**F**	**8**	**F**	**4**	**3**	**0**	**3**	**F**	**5**	**4**	**3**	**1**	**1**	**44**	**--**
	**G**	**3**	**9**	**0**	**2**	**0**	**1**	**6**	**1**	**3**	**2**	**5**	**3**	**2**	**F**	**4**	**2**	**10**	**6**	**11**	**70**	**--**
	**Total**	**3**	**12**	**0**	**3**	**6**	**3**	**6**	**9**	**3**	**6**	**8**	**3**	**5**	**na**	**9**	**6**	**12**	**9**	**12**	**114**	**--**
**# of Inf. with Fever**	**P**	**0**	**1**	**0**	**0**	**0**	**1**	**F**	**0**	**F**	**0**	**1**	**0**	**1**	**F**	**0**	**0**	**1**	**0**	**0**	**5**	**--**
	**G**	**1**	**1**	**0**	**1**	**0**	**0**	**0**	**0**	**0**	**0**	**1**	**2**	**0**	**F**	**0**	**0**	**2**	**0**	**3**	**11**	**--**
	**Total**	**1**	**2**	**0**	**1**	**0**	**1**	**0**	**0**	**0**	**0**	**2**	**2**	**1**	**na**	**0**	**0**	**3**	**0**	**3**	**16**	**--**
**Days on Antibx**	**P**	**0**	**29**	**0**	**0**	**50**	**10**	**F**	**75**	**F**	**25**	**0**	**0**	**24**	**F**	**50**	**18**	**F**	**0**	**0**	**281**	**--**
	**G**	**25**	**44**	**0**	**25**	**0**	**10**	**18**	**19**	**30**	**3**	**39**	**25**	**5**	**F**	**38**	**22**	**116**	**43**	**37**	**499**	**--**
	**Total**	**25**	**73**	**0**	**25**	**50**	**20**	**18**	**94**	**30**	**28**	**39**	**3**	**29**	**na**	**88**	**40**	**116**	**43**	**37**	**780**	**--**

[a]Influenza A, [b]*Pithomyces species*, [c]*Trichophyton tonsurans*, [d]rhinovirus, [e]*Cyclospora*, [f]*Candida albicans*, [g]metapneumovirus, [h]Moraxella, [i]Enterovirus, [j]Dematiaceous mold, [k]*S. aureus*, [l]HSV, [m]*C. dificile*, [n]*Hemophilus sp.*

Abbreviations: TISS, total infection severity score; Inf, infection; Antibx, antibiotic treatment; UTI, urinary tract infection; GI, gastrointestinal tract infection

Supplemental Table S7. Characterization of wart areas on WHIM patients treated with G-CSF and plerixafor. P, plerixafor; G, G-CSF; NR, no significant response; ne, non-evaluable; 1+, a single wart; 2+, a few warts in a group; 3+, a large wart area; 4+, a wart area extensively covering an entire body part; na, not available; PR, partial response (>=50% reduction in size); CR, complete response of a wart area; NR, no response; mos, months; yrs, years; ND, not determined; L, left; R, right. For wart areas that responded to drug, the approximate month on drug when the response was first observed is noted. Stability was determined by comparing photographs obtained at baseline to antecedent photographs obtained at NIH visits preceding enrollment. Photos are in Figure 6 and Supplemental Figure S7.

	Warts at Baseline Visit				Response to Study Drug			
**Patient**	**Location**	**Type**	**Stability (yrs)**	**Burden**	**P**	**G**	**Post-study change**	**Total wart areas**
**M01**	Forehead	flat	na	2+	CR, month 8	worse	no recurrence in 5 years on G	1
**M02**	R Palm	common	na	1+	CR, month 4	NR	recurrence after 3 years on G	9

R Finger 2	mosaic	5+	3+	NR	NR	regression by 5 months, then recurrence on G
R Finger 5	mosaic	5+	3+	PR, month 4	worse	Complete regression, then recurrence on G
L Finger 2	common	5+	3+	PR, month 8	NR	Complete regression by 5 months, no recurrence in 3 years on G
L Finger 5	mosaic	5+	3+	PR, month 8	NR	worse on G
L Toe 1	plantar	5+	2+	PR, month 8	NR	Complete regression by month 5, no recurrence in 3 years on G
R toe 1	plantar	5+	2+	CR, month 12	NR	no recurrence in 3 years on G
L Knee	common	na	1+	NR	NR	Complete regression by 1 year on G, no recurrence by 3 years

	Genitalia	common	7	2+	CR, month 4	worse	no recurrence in 3 years on G	
**M03**	R Finger 4	mosaic	2+	3+	CR, month 12	worse	ND	16
	R Thumb	mosaic	2+	3+	CR, month 12	na	ND	
	R Finger 2	mosaic	2+	3+	NR	CR, month 4	ND	
	L Finger 2	mosaic	na	1+	CR, month 12	worse	ND	
	L Finger 3	mosaic	2+	2+	ne	ne	ND	
	L Finger 4	mosaic	2+	2+	CR, month 12	na	ND	
	L Finger 5	mosaic	2+	2+	PR, month 12	CR, month 0	ND	

	R Dorsum foot	mosaic	2+	4+	PR, month 8	CR, month 0	ND	
	R Plantar foot	mosaic	2+	4+	PR, month 8	PR, month 4	ND	
	L Dorsum foot	mosaic	2+	4+	CR, month 12	NA	ND	
	L plantar foot	mosaic	2+	4+	NR	NR	ND	
	L Suprapubic	mosaic	2+	3+	PR, month 12	PR, month 4	ND	
	L Achilles	mosaic	2+	4+	NR	PR, month 12	ND	
	Neck	mosaic	na	2+	PR, month 12	NR	ND	
	Genitalia	condyloma accuminata	2+	4+	NR	NR	ND	
	chest	flat	5+	2+	NR	NR	ND	
**M04**	R hand	flat	5+	1+	NR	PR by month 12	complete regression by 3 years on G	2
	Genitalia	condyloma accuminata	5+	2+	PR, month 12	High risk HPV+, month 12	ND	

**M05**	R hand	common	2+	3+	NR	NR	NR after 2 years off G	6
	L hand	common	2+	3+	NR	NR	NR after 2 years off G	
	L foot plantar	plantar	2+	2+	NR	NR	NR after 2 years off G	
	Extremities	flat	2+	3+	NR	NR	NR after 2 years off G	
	Torso	flat	2+	3+	NR	NR	NR after 2 years off G	
	Genitalia	common	2+	2+	NR	NR	ND	
**M06**	R thumb	common	na	1+	PR, month 4	NR	ND	3
	L foot dorsum	common	na	2+	NR	NR	Stable for 2 years on G	
	R elbow	common	new on P	1+	worse	NR	Stable for 2 years on G	
**M07**	R Hand dorsum	common	3	3+	NR on 2 months of P	PR, month 8	Stable for 1 year on G	6

	L Hand dorsum	common	3	3+	PR on 2 months of P	PR, month 4	Stable for 1 year on G	
	L Elbow	common	1+	1+	na	CR, month 4	ND	
	R Elbow	common	1+	1+	na	CR, month 12	ND	
	L dorsal foot	common	na	1+	NR on 2 months of P	NR		
	Buttocks, genitals	condyloma accuminata	6+	3+	NR on 2 months of P	NR	Stable for 2 years on G	
**M08**	none							0
**M09**	L plantar	plantar	na	2+	NR after <1 mo on P	NR	ND	2
	R plantar	plantar	na	2+	NR after <1 mo on P	NR	ND	
**M10**	Genitalia	common	3+	3+	NR	ne	patient refused Gyn exam after G	1
**M11**	none							0
**M12**	R Thumb	common	na	1+	NR	NR	ND	6

	L Thumb	flat	4+	1+	NR	NR	ND	
	R Toes 2-5	mosaic	4+	4+	NR	worse, month 12	some spontaneous regression after 2 years	
	R Foot side	mosaic	4+	4+	NR	worse	ND	
	R Sole	mosaic	na	4+	NR	worse, month 12	ND	
	L Sole	mosaic	na	3+	CR, month 8	worse, month 12	ND	
**M13**	none							0
**M14**	none				<1 month on P	<1 month on G	ND	0
**M15**	R hand	mosaic	3+ mos	3+	PR, month 4; no month 12 visit	NR	ND	5
	L hand	mosaic	3+ mos	3+	PR, month 4; no month 12 visit	NR	ND	
	R elbow	mosaic	3+ mos	3+	CR, month 4; no month 12 visit	NR	ND	

	L elbow	mosaic	3+ mos	3+	PR, month 0; no month 12 visit	NR	ND	
	genitalia	condyloma accuminata	na	4+	NR; no month 12 visit	NR	ND	
**M16**	R knee	flat	na	1+	ne	ne	ND	4
	L knee	flat	na	1+	ne	ne	ND	
	R elbow	flat	na	1+	ne	ne	ND	
	L elbow	flat	na	1+	ne	ne	ND	
**M17**	R plantar	plantar	1+	2+	NR on 8 months of P	PR, month 4	ND	9
	L plantar	plantar	1+	2+	NR on 8 months of P	PR, month 4	ND	
	L axilla	common	na	1+	ne	ne	ND	
	R axilla	common	na	1+	ne	ne	ND	
	R lateral knee	flat	na	1+	ne	ne	ND	
	R proximal calf	flat	na	1+	ne	ne	ND	
	L medial calf	flat	na	1+	ne	ne	ND	
	R upper thigh	flat	na	1+	ne	ne	ND	
	L upper thigh	flat	na	1+	ne	ne	ND	

**M18**	Right buttock	common	na	2+	ne	ne	resected	3
	R Fingers 1-3	common	na	1+	ne	ne	no change after 1 year on G	
	L finger 2	common	na	1+	NR	worse, month 4	worse after 1 year on G	
**M19**	none						ND	0

Supplemental Table S8. Heterogeneous baseline distribution and improvement of HPV disease in WHIM patients treated in a crossover study of plerixafor (P) and G-CSF (G). L, left; R, right. Wart burden in each body site is denoted by + signs: 1+, a single wart; 2+, a few warts in a group; 3+, a large wart area or multiple discrete wart areas in the indicated body part; 4+, a wart area extensively covering an entire body part. Wart areas that improved on drug are denoted by red and green + signs. mos, months; yrs, years; ND, not determined; Some warts increased in size during the study (not shown; see Supplemental Table S7 for details).

		Evaluable Wart Distribution and Burden (+) at Baseline Visit (+, better on Plerixafor; +, better on G-CSF)									
**Patient**	**Drug Order**	**R hand**	**L hand**	**R foot**	**L foot**	**Anogenital warts**	**Knee**	**Elbows**	**Torso**	**Other**	**Notes**
**M01**	GP									+++ (forehead)	Warts increased on G
**M02**	GP	+++	++	+	+	++	+				
**M03**	PG	++++	+++	++++	+++	+++			+++	++ (neck)	Imiquimod to genitals & skin during mos 10-14 on P
**M04**	GP	+				++					
**M05**	PG	+++	+++			+++			+++	+++ (extremities)	Imiquimod to hands mos 10-14 on G
**M06**	PG	+			++	+		+ (new on P)			
**M07**	GP	++	++		+	+++		+			Failed P at mo 2
**M09**	PG			+	+						Failed P at 1 week
**M10**	PG					+++					Declined Gyn exam at end of G
**M12**	PG	+	+	++++	+++						Declined all Gyn exams
**M15**	GP	+++	+++			++++		+++			imiquimod to genitalia 1 mo on G, 4 mos on P

**M17**	GP			++	++			+		+ (axillae, calves, thighs)	Failed P at mo 8
**M18**	PG	+	+							+ (buttock)	

Supplemental Table S9. Wart status and responses during therapy with G-CSF or plerixafor in WHIM patients stratified by age.

	**Patient Age (yrs)**		**Total, *n***
	**>18, *n*[a]**	**<18, *n***	
**Patients on study**	14	5	19
**Wart status at randomization**			
Positive history of warts	13	2	15
Warts present at the time of randomization	12	2	14
Evaluable warts during G-CSF and plerixafor treatment[b]	11	2	13
**Wart responses to therapy[c]**			
Clinically significant improvement during G-CSF treatment	1[d]	0	1
Clinically significant improvement during plerixafor treatment	4	1	5
Increased wart burden during G-CSF treatment	4	1	5
Increased wart burden during plerixafor treatment	1	0	1
No improvement on either drug	4	0	4

[a]Abbreviations: *n*, number; yrs, years

[b]Patient M16 did not have photography of relevant wart areas

[c]Three patients with warts failed plerixafor due to side effects (M09 and M17) or failure to reach the prespecified ANC level (M07). M07 and M09 failed during the drug equilibration period; M17 failed at month 6 of the drug treatment period. No patients with warts failed G-CSF. Thus, of the 13 evaluable patients who had warts at the time of randomization, all 13 received a full 12-month treatment course of G-CSF, whereas 10 received a full 12-month treatment course of plerixafor.

[d]Improvement occurred within the first 2 months of switching to G-CSF in patient M03 who had major regression of multiple large wart areas during treatment with plerixafor given first, suggesting a possible carryover effect.

Supplemental Table S10. Anogenital HPV disease was common in WHIM patients but responded poorly to both plerixafor and G-CSF.

						Anogenital HPV Disease					
									Response to		
Patient	Age	Sex	Genotype	Drug Order	HPV Vax?	Duration (yrs)	History	Baseline Disease	P	G	Notes
**M01**	14	M	R334X	GP	yes	na	na	WNL	na	na	
**M02**	51	F	R334X	GP	yes	27	Anogen-ital warts	LSIL	CR of warts by month 4	new vagi-nal warts	
							CIS, cervix X2	LR/HR HPV+			
							CIS, rectum				
							TAH				
**M03**	56	M	R334X	PG	no	19	Condyl. accumin.	condyloma accuminata	none	none	imiquimod applied to skin and genitalia during months 8-12 of P arm
**M04**	36	F	R334X	GP	no	16	Condyl. accumin.	External genital warts	PR of warts by month 12	none	
							HR HPV+	Cytopath. negative			
								HR HPV negative			
**M05**	52	F	R334X	PG	no	33	warts	warts	none	none	imiquimod to hands during G; clobetasol applied to genitalia
							VIN-3	LSIL			
							CIN	HR HPV+			
							TAH				
							HR HPV+				
**M06**	20	F	R334X	PG	yes	ND	LSIL	LSIL	none	none	
							HR HPV+	HR HPV+			
**M07**	10	M	R334X	GP	yes	7	Condyl. Accum.	condyloma accuminata	none	none	P stopped after 2 mos
**M08**	33	M	R334X	GP	no	na	none	WNL	none	none	
**M09**	34	F	G323fs343X	PG	no	~10	warts	WNL	na	na	P stopped after 1 wk
							LGSIL				
							HR HPV+				
							TAH				
**M10**	37	F	S338X	PG	no	15	warts	warts	CIN-1	ne	Declined gyn exam after G arm
							LSIL	LSIL			
							HR HPV+	LR/HR HPV+			
**M11**	14	M	S338X	GP	no	na	none	WNL	na	na	

**M12**	25	F	R334X	PG	no	na	none	none by history	ne	ne	Declined gyn exams
**M13**	12	F	G336X	GP	yes	na	none	WNL	na	na	
**M14**	29	M	K327fs	PG	no	na	none	WNL	na	na	P and G stopped after 1 wk
**M15**	27	F	E343X	GP	yes	12	Condyl. accumin.	condyloma accuminata	none	none	Missed month 12 visit on P; Imiquimod to genitalia for 1 mo on G and 4 mos on P
							HR HPV+	LSIL			
							CIN-2	HPV HR+			
**M16**	57	F	E343X	PG	no	37	warts	ASCUS	none	ne	missing photos
								HR HPV+	p16- CIN-I		
**M17**	38	F	S339fs	GP	no	9	cervical dysplasia	WNL	na	na	P stopped after 8 months
							Condyl. accumin.				
**M18**	38	F	R334X	PG	yes	na	none	ASCUS	none	none	
								HR HPV neg			
**M19**	16	F	V320fs	PG	yes	na	none	WNL	na	na	

Abbreviations: ASCUS, atypical squamous cells of undetermined significance; CIN, cervical intraepithelial neoplasia; CIS, carcinoma in situ; Condyl. accum., condyloma accuminata; CR, complete response; F, female; G, G-CSF; HPV, human papillomavirus; HR, high risk; LR, low risk; LSIL, low-grade squamous intraepithelial lesion; M, male; mo, month; ne, not evaluable; P, plerixafor; PR, partial response; TAH, total abdominal hysterectomy; vax, vaccination; VIN, vaginal/vulvar intraepithelial neoplasia; wk, week; WNL, within normal limits; na, not applicable

Supplemental Table S11. Time to wart area improvement (>=50% reduction in size) in WHIM patients treated with G-CSF or plerixafor. Total time on drug includes 2 months on the equilibration phase and 12 months on the treatment phase for each drug. % refers to the percentage of the total 26 wart areas that showed improvement beginning in the indicated time interval on drug.

	**Wart Areas Improved on Study Arm, n (*%*)**	
**Time on Drug (months)**	**Plerixafor**	**G-CSF**
**0-2**	2 (*8*)	2 (*16*)
**2-6**	7 (*27*)	7 (*54*)
**6-10**	7 (*27*)	1 (*8*)
**10-14**	10 (*38*)	3 (*23*)
**Total**	26 (*100*)	13 (*100*)

Supplemental Table S12. Responsiveness of warts by type and size in WHIM patients to crossover treatment with plerixafor and G-CSF. *n*, total number of wart areas of the given type and size defined at baseline across all WHIM patients; P, regression of a wart during plerixafor treatment but not during G-CSF treatment; G, regression of a wart during G-CSF treatment but not during plerixafor treatment; Both, regression of a wart during both plerixafor and G-CSF treatment; NR, no significant response on either drug; ne, non-evaluable on either drug; 1+, a single wart; 2+, a few warts in a group; 3+, a large wart area; 4+, a wart area extensively covering a body part. Warts tabulated under 'P' and 'G' include warts that improved on both drugs.

			**Better on Drug**				**Worse on Drug**				
		n	**P**	**G**	**Both**		**P**	**G**	**Both**	**NR**	**ne**
**Types**	**Common**	22	4	4	1		1	2	0	6	4
	**Flat**	11	1	1	0		0	1	0	4	5
	**Mosaic**	25	13	1	4		0	7	0	2	1
	**Plantar**	7	2	2	0		0	0	0	3	0
	**Condyloma accuminata**	4	1	0	0		0	0	0	3	0
**Size**	**1+**	20	3	3	0		1	2	0	4	8
	**2+**	18	7	2	1		0	2	0	6	2
	**3+**	21	10	2	2		0	3	0	5	0
	**4+**	10	1	1	2		0	3	0	3	0
**Totals**		**69**	**21**	**8**	**5**		**1**	**10**	**0**	**18**	**10**

Supplemental Table S13. Summary of efficacy endpoints for each patient. The dosage values listed are for the highest doses given during the 2 one-year treatment phases. The ANC and ALC maintenance tests tested the ability of the equilibrated dose of each drug to maintain the ANC and ALC at or above prespecified thresholds of 500 and 1000 cells/microliter, respectively, during the 12-month treatment phase. Clinically significant wart regression refers to complete or near complete regression of large wart areas that improved patient quality of life. Infections occurring during the treatment phase are enumerated and the total infection severity score was computed according to the prespecified rules described in the Methods section.

				Maintenance S=success F=failure				Clinically Significant Wart Regression		Infections			
	Dosage in Treatment Phase (Failures)			ANC[a]		ALC				TISS		Number	
**Patient**	**G** (µg/kg/d)	**P** (mg/kg/d)	**Failure reason**	**G**	**P**	**G**	**P**	**G**	**P**	**G**	**P**	**G**	**P**
**M01**	2.3	86		S	S	F	S	No	Yes	14	0	3	0
**M02**	0.8	29		S	S	F	S	No	Yes	22	12	9	3
**M03**	0.6	48		S	S	F	S	Yes[b]	Yes	0	0	0	0
**M04**	1.4	92		S	F	F	S	No[c]	No[c]	11	2	2	1
**M05**	0.6	32		S	S	F	S	No	No	0	18	0	6
**M06**	0.5	37		S	F	F	S	No[c]	No[c]	4	10	1	2
**M07**	2.1	93	Poor ANC response in Equilibration Phase 2	F	F	F	F	No	No	17	F	6	F
**M08**	0.4	23		S	S	F	S	na	na	3	27	1	8

**M09**	0.6	33	Psoriasis in Equlibration Phase 1	S	F	F	F	No[c]	No[c]	10	F	3	F
**M10**	1.1	39		S	S	F	S	No[c]	No[c]	4	14	2	4
**M11**	3.8	79		F	F	F	S	na	na	17	6	5	3
**M12**	0.7	34		S	S	F	S	No	Yes	14	0	3	0
**M13**	3.0	94		F	F	F	S	na	na	5	15	2	3
**M14**	0.3	24	Arthritis in Equilibration Phases 1 & 2	F	F	F	F	na	na	F	F	F	F
**M15**	0.7	54		S	S	F	S	No	Yes	14	15	4	5
**M16**	0.6	32		S	S	F	S	ne	ne	6	9	2	4
**M17**	0.8	54	Arthralgia in mo. 6 of Treatment Phase 2	F	F	F	F	No[c]	No[c]	26	F	10	F
**M18**	0.6	34		S	S	F	S	No[c]	No[c]	20	2	6	1
**M19**	0.5	44		S	S	S	S	na	na	36	1	11	1

[a]Abbreviations: G, G-CSF; P, plerixafor; ANC, absolute neutrophil count; ALC, absolute lymphocyte count; TISS, total infection severity score; S, success; F, failure; mo., month; ne, not evaluable (insufficient clinical photography); na, not applicable (no warts)

[b]Several warts that had regressed partially during plerixafor treatment continued to regress after switching to G-CSF. One wart that had not regressed during plerixafor began to regress and regressed completely soon after switching to G-CSF.

[c]Patients with low wart burdens at baseline (see Supplemental Tables S7, S8 and S10).

Supplemental Table S14. HPV diversity in WHIM patients. Skin biopsies and swabs and peripheral blood samples were enriched for viruses and analyzed by rolling circle PCR. The sequences have previously been reported in reference 25 and are described here with respect to events during the present study. red, new HPV type; purple, new HPV species; blue, HPV vaccine types; PAVE, papillomavirus episteme; i, incomplete sequence; is, isolate; vax, vaccination; G, G-CSF; P, plerixafor; R, right; L, left; EOS, end of study visit. Six of the 10 patients were sampled only before treatment so that a systematic analysis of the effect of treatment on HPV diversity was not possible.

					**HPV isolates**			
**Patient**	**Drug Order**	**Study Period**	**Location**	**Sample type**	**Species group**	**type**	**HPV reads (% of total)**	**Time of HPV vax**
**M01**	GP	Baseline	Warts: forehead	Swab	Beta2	HPV38	98.6%	pre
**M02**	GP	EOS	Warts: R 5th digit, R index, R medial big toe, L big toe, L forearm	Swab	Alpha4<br>Gamma<br><br><br><br><br>Gamma07<br>Gamma12<br>Alpha10<br>Gamma07	HPV2<br>is915F<br>KN3_w01c05b (new name in PAVE: HPV-mKN3)<br>HPV109<br>HPV127<br>HPV44<br>HPV149	22%	Y2M4
			Warts: R index finger, R dorsal wrist, L ventral knee	Swab	Alpha4	HPV2	2%	
		Baseline	Blood	Blood	ND	ND	NA	
**M03**	PG	P month 8	Warts: abdomen,	Swabs	Beta2<br>Alpha4	HPV80<br>HPV57	46.3%	no

			right thumb		Gamma24<br>Gamma22<br>Gamma11<br>Gamma22<br>Gamma15	HPV197<br>w18c07<br>w18c25<br>w18c39<br>w18c134		
		P month 12	Warts: R foot, R toes, abdomen, R middle finger, L ankle)	Swabs	Alpha4<br>iAlpha2<br>Beta2	HPV27<br>HPV28<br>HPV80	83.1% (97% HPV27)	
			Blood	Blood	ND	ND	NA	
		G month 0	Pubic area	Scraping	Mu2<br>Beta2<br>Gamma19	HPV63<br>HPV22<br>w18c11d	8.8%	
			Sole	Scraping	Alpha4	HPV27	96.1%	
			Ankle	Scraping	Alpha4	HPV27b	95.3%	
			Finger	Scraping	Alpha4	HPV27	97.2%	
		EOS	R thumb, R index, L sole, R sole	Swabs	Mu2<br>Alpha4<br>Alpha4<br>Gamma	HPV63<br>HPV27<br>HPV57<br>is915F-w18c574 (new name in PAVE: HPV-mKN3)	ND	
			R dorsal 4th digit, R palm (no history of warts)	Swabs	Alpha4	HPV57	ND	
**M04**	GP	Baseline	R hand, L thumb wart and hand (clear skin), Buttocks, mons, vulva	Swabs	Alpha2	HPV03	72.5%	post

**M05**	PG	P month 4	R inner thigh	Biopsy	Alpha2	HPV28 (HPyV6)	18.0% (63.5% HPyV6)	post
			Warts: R knee, R shin, R ankle, abdomen, R forefinger, Forehead	Swabs	Beta2<br>Beta2<br>Alpha4<br>Gamma22<br>Alpha2<br>Gamma8<br>Gamma09<br>Gamma	HPV17<br>HPV80<br>HPV27<br>HPV172<br>HPV28<br>HPV164<br>w23c101c (73% ~ to HPV129)<br>w23c08c	87%	
			Blood	Blood	ND	ND	NA	
		G month 0	Warts: L & R hand, L sole, abdomen, Ankle, R knee, scalp, SCC	Swabs	Alpha4<br>Alpha2<br>Alpha4<br>Beta1	HPV57<br>HPV28<br>HPV27<br>HPV14	13%	
			R labial ulcer, L buttock verruca, introitus suture or verruca, L labia		Alpha1<br>Alpha10<br>Alpha07<br>Alpha14	HPV42<br>HPV06<br>HPV18<br>HPV90	8.5%	
			Blood	Blood	ND	ND	NA	
**M06**	PG	Baseline	vaginal LGSIL	Swab	Alpha5<br>Alpha14<br>Gamma06	HPV51<br>HPV90<br>w02c24a (83.97 ~ to HPV103)	77.9% (1% gamma)	pre
**M09**	PG	Baseline	R foot wart, right hand clear	Swabs	Alpha04<br>Gamma21<br>Gamma12<br>Gamma24	HPV57<br>HPV167<br>w07c68b (72.27 ~ to HPV127)<br>w07c74b (74.08 ~ to HPV178)	13.6%	no
			Blood	Blood	ND	ND	NA	
		P month 0	psoriasis (palm,	Swabs	Beta-is<br>Gamma-is<br>Gamma-is	HPV36<br>HPV197<br>HPV65	84%	

			abdomen, calf, sole)		Gamma7<br>Beta2<br>Beta-is<br>Gamma7<br>Gamma	HPV134<br>HPV23<br>HPV150<br>HPV139<br>DyskD_w07c34d (72% ~ to DyskD)		
**M12**	PG	Baseline	Warts: R thumb, R 4th toe, R soles, R foot dorsum	Swabs	Alpha4<br>Gamma11	HPV57<br>HPV136	94.3% (99% HPV57)	no
**M15**	GP	Baseline	Warts: R 2nd digit, L 2nd digit, R elbow, L elbow, vulva, cervix	Swabs	Alpha2<br>Beta3<br>Gamma15<br>Beta1<br>Gamma3<br>Beta2<br>Gamma23<br>Gamma1<br>Gamma8<br>Gamma<br>Gamma12<br>Gamma24<br>Gamma18<br>Gamma<br>Gamma	HPV3<br>HPV76<br>HPV146<br>HPV21<br>HPV50<br>HPV110<br>HPV175<br>HPV173<br>HPV164<br>USD2R.w34Ec07a (new name in PAVE: HPV-mSD2)<br>w34c11a(77% ~ to HPV148)<br>w34c28a (77% ~ to HPV116)<br>w34c34a(72% ~ to HPV156 or 75% to CH2)<br>w34c04a (61% ~ to HPV166, and 71% identical to isolate 915F but only 96% of query)<br>w34c14a (69% identical to HPV116)	87%	pre
			wartless w/o history: R	Swabs	Alpha2<br>Beta2<br>Gamma3	HPV3<br>HPV23<br>HPV50	80%	

			palm, R forearm, L upper arm, Forehead, L knee		Beta5<br>Gamma8<br>Beta5<br>Mu<br>Beta2	HPV150<br>HPV164<br>HPV5<br>HPV204<br>HPV110		
**M16**	PG	Baseline	Blood	Blood	ND	ND	NA	no
			Warts: R dorsal wrist, R 4th digit nail, L anterior knee (history of warts burned off), R anterior knee (history of warts), L anterior ankle (history of warts, L inner forearm (no history of warts)	Swabs	iAlpha2<br>iGamma7<br>iGamma	HPV3<br>HPV134<br>Ui915F (New name in PAVE: HPV-mKN3)	18%	
			Tissue skin tag	Swabs	Gamma<br><br>Beta1<br>Gamma8<br>iGamma<br><br>iGamma<br><br>iGamma7	Ui915F (New name n PAVE: HPV-mKN3)<br><br>HPV124<br>HPV164<br>UiSD2R _w35c51c (New name in PAVE: HPV-mSD2)<br>UiDysk4 _w35c15c (New name in PAVE: HPV-mKN1)<br>HPV149	80%	
Totals					30 Alpha			

					18 Beta<br>44 Gamma<br>3 Mu			

Supplemental Table 15. Non-infectious adverse events in WHIM patients treated with G-CSF or plerixafor. Events were tabulated for all the time on drug from patient randomization through the end of study visit. The number of events documented in each category is listed for each patient in black for the G-CSF (G) arm of the study and in red for the plerixafor (P) arm of the study. Totals give the number of patients having at least one event in the indicated category. Color codes for each adverse event in the EVENT column were assigned based on the presence of at least one instance of the following types of relatedness: Probably related to drug; Possibly related to drug; Definitely related to drug; Not related or Unlikely to be related to drug. Color code for event cells: gray, grade 2; yellow, grade 3; green, serious adverse event. Color code for patient number cells: light blue, patients who failed plerixafor (patients M07 and M09 during the equilibration phase, patient M17 at month 6 of the treatment phase); brick red, patient M14 who failed both plerixafor and G-CSF during the equilibration phases. Abnormal blood chemistries were grouped together. Those that were grade 2 (coded as gray cells) included 10 instances of increased bilirubin, 12 instances of hypophosphatemia, and 16 instances of elevated creatinine, all transient.

	Non-infectious Adverse Events on G-CSF (G) or Plerixafor (P)																				
	Drug Order and Patient Number																				
	GP	GP	PG	GP	PG	PG	GP	GP	PG	PG	GP	PG	GP	PG	GP	PG	GP	PG	PG	Total	
**EVENT**	M01	M02	M03	M04	M05	M06	M07	M08	M09	M10	M11	M12	M13	M14	M15	M16	M17	M18	M19	**G**	**P**
**Abdominal bloating**																1				**1**	**0**
**Abdominal cramps/pain**							1	1	1											**2**	**1**
**Abnl Bld Chem**	1/1	2/4	1	3/2	2		1/2	1/1	3	5/2	2	2	4	1/2	4/1	5/3	1	1		**14**	**12**
**Abnl BM Bx**		1									1									**2**	**0**
**Abnl CXR**																1				**0**	**1**
**Acne**																	1		1	**2**	**0**
**Anemia**						1				1		3/2								**2**	**2**
**Anxiety**															1	1				**1**	**1**
**Blurred vision**					1															**0**	**1**
**Bone Pain**		5	1/1	8	1/1	1/1	9	2/1	3		2	2	2/1			2	2/1	1	1	**14**	**7**
**Decreased Bone mineral content**								1		1		1								**2**	**1**
**Decreased plts**																		2/3		**1**	**1**
**Dehydration**				1																**1**	**0**

Diarrhea																1			1	1	1
Dizziness		1																		1	0
Dry Eye		1																		1	0
Ecchymosis				1						1										1	1
Elective Surgery				1	3										1/1		1			3	2
Foot pain										1										0	1
Ganglion cyst																	1			1	0
GERD															1					0	1
Gout								1/1												1	1
Headache	2	3	1/2	1		3								1				3	1	6	3
Hyperglycemia					1										1	2/2				3	1
Hyperuricemia				1/1	1			1/1								2	2			4	3
Hypoglycemia							1										1			2	0
Hypomania																		1		0	1
Increased ALC						1														0	1
Increased B12								1		1										1	1
Injection site rxn						1		1	1										3	0	4
Joint Pain	2	1	1	7			1	1	2	4	1	3		4/2		1	6/2	1		11	5
Leg Pain					1										1					1	1
Menstrual irregularity/pain																	1		2	1	1
Migraine					1											1	1			1	2
Nausea/vomiting		1		7		2		1	1	1			3							6	1
Oral aphthous ulcers																	1		2/7	2	1
Oral Lesion										2										0	1
Ovarian Cyst				8					2/1										1	3	1
Palpitations				1																1	0
Peripheral Edema					1											1				1	1
Photosensitivity																		1		0	1
Pituitary Adenoma					1															0	1
Rash		2		1/1				1	2/2		1/2		1		1	2		1		3	9
Scabies				1																0	1
Skin Disorder										1										1	0
Sleep Disorder							1									1				0	2
Splenomegaly	1			1																2	0
Stye																	1			1	0
Subcutaneous nodule																		1		0	1
Syncope													1							0	1
Tearing																1				0	1
TIA					1															1	0
Tinnitus					1/1	1														2	1
Tooth Extraction				1			1								1			1		2	2
Trauma		2/1		1			2				2		1		2			1/1		5	4
Weight gain			1	1/1	1			1										1/1		2	5
Wheezing				1		1														2	0
Zenker diverticulum																		1		1	0
																		Totals		116	91
Totals																				Totals	
P	2	4	3	7	6	3	3	9	4	5	2	4	4	2	6	9	5	8	5	91	
G	3	8	4	15	9	6	6	7	6	6	5	2	3	3	5	7	9	8	4	116	
Total P+G	5	12	7	22	15	9	9	16	10	11	7	6	7	5	11	16	14	16	9	207	

Abbreviations: P, plerixafor; G, G-CSF; TIA, transient ischemic attack; Abnl Bld Chem, abnormal blood chemistry; Abnl BM bx, abnormal bone marrow biopsy; Abnl CXR, abnormal chest X-ray; plts, platelets; GERD, gastro-esophageal reflux disorder; rxn, reaction; ALC, absolute lymphocyte count

Supplemental Table S16. Cell surface markers used for immunophenotyping with monoclonal antibodies. The specific antibody clones and fluorophores are available from the indicated Manufacturer website using the indicated Catalog number.

Cell Surface Marker	Manufacturer	Catalog #
CD14	Becton Dickinson	340585
CD14	ThermoFisher	MHCD1401
CD62L	Becton Dickinson	559772
CD45	Becton Dickinson	347464
CD45	ThermoFisher	47-0459-42
CD45RA	Beckman Coulter	IM0584U
CD3	Becton Dickinson	564713
CD3	Becton Dickinson	341091
CD3	ThermoFisher	MHCD0301-4
CD3	ThermoFisher	MHCD0331
CD4	Becton Dickinson	562658
CD4	Becton Dickinson	565997
CD4	Becton Dickinson	557852
CD4	ThermoFisher	17-0049-42
CD4	ThermoFisher	MHCD0431
CD19	Becton Dickinson	562947
CD19	Becton Dickinson	562653
CD19	ThermoFisher	MHCD19014
CD19	ThermoFisher	17-0198-42
CD8	Becton Dickinson	565165
CD8	ThermoFisher	MHCD0805
CD8	ThermoFisher	MHCD0831
CD56	Becton Dickinson	340685
CD56	Becton Dickinson	562780
CD56	BioLegend	318310

Statistical Analysis Plan for: A Phase III, Double-blind Randomized, Crossover Study of Plerixafor Versus G-CSF in the Treatment of Patients with WHIM Syndrome

Protocol Number: 14-I-0185

October 19, 2020
Michael Fay, Biostatistics Research Branch, NIAID

This statistical analysis plan (SAP) is based on NIAID IRB approved protocol version 6.0, and subsequent communications with the FDA from February to October 7, 2020. Since Dean Follmann, the primary statistician, has been unblinded from some preliminary analyses, this SAP was written by Michael Fay, who like the rest of the study team is blinded to patient treatment.
As of October 19, 2020 all subjects had been enrolled in the study, and there is no further efficacy data expected to be collected from this study. This version of the SAP is based on blinded data.

1. Overview of the Study

This is a double-blinded, randomized, crossover study comparing the efficacy of treatment with the chemokine receptor CXCR4 antagonist plerixafor (P) to G-CSF (G) in subjects with a clinical diagnosis of WHIMS, a panleukopenic form of severe congenital neutropenia and immunodeficiency caused by gain-of-function mutations in the C-terminus of CXCR4 that promote retention of mature leukocytes in the bone marrow. Nineteen subjects were randomized to 1 year of treatment with either P or G, followed by a crossover to the second drug for 1 year. Each one-year treatment arm period is preceded by a 2-day washout period followed by an 8- week equilibration period during which study drug dosing is initiated and adjusted to establish an absolute neutrophil count (ANC) of approximately 500-1500 cells/µL. A subject’s ANC is monitored every 2 months during the one-year treatment periods and study drug dosage adjusted when ANC ≤500 cells/µL or ≥7500 cells/µL. Participants maintain a study Memory Aid in which they record daily treatments and any new symptoms. After completing both treatments, subjects are offered open-label G and enter a post-treatment observation period during which they continue to submit the study Memory Aid. The study completion visit occurs 4-8 months after the last day of the second year of treatment. The protocol defined, prespecified primary endpoint is the total infection severity score (TISS), which is based on the number and intensity of the infections during each treatment period. The statistical analysis plan presented here for TISS is that which is specified in the protocol.

A new secondary endpoint not previously specified in the protocol based on ANC has been developed through discussions with the FDA after the study was essentially finished but before the data were unblinded. This ANC endpoint was suggested because it is a more objective endpoint than the TISS. Within each one-year treatment period, there are 11 blood samples scheduled to be taken for each participant: 2 samples (a trough sample and a peak sample) taken at each of 4 visits at the National Institutes of Health Clinical Center (NIH-CC) (months 0, 4, 8, and 12), and 3 trough local blood samples measured at a local laboratory. If logistical obstacles prevent a scheduled visit to the NIH-CC, a local laboratory reading of ANC may be substituted for a missed NIH-CC trough visit reading. These replaced local readings are not counted as missing, and for ease of exposition will be called NIH-CC readings after their original planned place of collection. All 11 measurements within each treatment period (if not missing for reasons unrelated to the health of the patient) are used to create the secondary response for ANC, and only the 4 peak measurements are used within each treatment period to create another secondary response for absolute lymphocyte count (ALC). The literature has shown (McDermott, Liu, et al, 2011, McDermott, et al 2014, 2019) that the ALC trough after a dose of plerixafor approximates the baseline, whereas the ANC trough does not return to baseline. The treatment of these data is provided in subsequent sections. Other secondary endpoints are described in Section 3.

2. Analysis for the Primary Endpoint of the Study

Since WHIMS is an immune deficiency disease, a clinical ramification is multiple infections. To measure these multiple infections we use the original protocol's primary endpoint: the total infection severity score (TISS). In order to define the TISS, we first define the infection severity score (ISS), which measures the severity of each infection. To compare a patient's ISS responses on one drug compared to on the other drug, we use a two-sample Wilcoxon rank sum test using scores based on total ISS within each period, where the total ISS (TISS) for any period is the sum of all the infection severity scores within that period. The score for a patient who does not have a drug failure and has complete follow up will be TISS for the period on P minus the TISS for the period on G-CSF. Because subjects are randomized to PG (P in period 1 and G in period 2) or GP (G in period 1 and P in period 2), if there are period effects or carryover effects (i.e., the drug given in period 1 has residual effects that carry over into period 2), the methods will still be valid. The complete details are in protocol in Section 14.4.1 (detailed definition of the ISS) and Section 14.4.2 (detailed description of the analysis). Section 14.4.2 includes details on how to handle patients that have drug failure on one or both of the drugs, or who drop out of a study arm for other reasons. The primary endpoint will be tested at the 2-sided 0.05 level.

3. Analysis of the Secondary Endpoints

We will test the primary endpoint and an ordered set of secondary endpoints using the fixed-sequence method (see e.g., FDA, 2017, p. 29). We test the primary endpoint at a 2-sided 0.05 level. If it is significant, then we go on to test the first secondary endpoint at a 1-sided 0.025 level; if the primary test is not significant, then we stop. We continue in this manner through the secondary endpoints in a predefined order, testing each at the 1-sided 0.025 level only if all of the previous secondary endpoint tests were significant at that same level. (See FDA, Jan 2017, Multiple Endpoints in Clinical Trials: Guidance or Industry, p. 29, Section 5). This is a slight modification of the usual fixed-sequence method in that we test at a 2-sided 0.05 level for the primary, but test at 1-sided 0.025 levels for the secondary endpoints. This does not create any type I error rate problems, because a 1-sided test rejected at the 0.025 level has equal strength of evidence as a 2-sided test rejected at the 0.05 level that was created by doubling the 1-sided p-value. We switch from 2-sided for the primary to 1-sided for the secondary because the primary was prespecified as 2-sided, and the secondary for the ANC endpoint is a non-inferiority hypothesis, which is inherently 1-sided.

The ordered list of secondary outcomes is as follows:

1.) Success on ANC: Proportion of ANC >500 cells/microliter is 75% or more.
2.) Success on ALC: Proportion of peak lymphocyte response >1000 cells/microliter is 75% or more.
3.) Incidence of infection
4.) Days of oral antibiotic/antifungal/antiviral treatment

The details on each secondary outcome are listed in Section 3.1-3.6.

3.1 ANC Score Based on Difference in Success

The first secondary endpoint will be a score based on ANC measurements. The score is calculated as the difference in the indicator of success on G minus the indicator of success on P, where those indicators are equal to 1 for success or 0 for failure, and success is defined in detail in Section 3.1.1. The primary hypotheses will be noninferiority ones based on the difference in probability of success in the two treatments, Δ=Pr(succ, G) – Pr(succ,P), testing the null hypothesis H0: $\Delta \geq M$, against the alternative H1: $\Delta < M$. Section 3.1.2 defines the hypotheses and motivates the margin used: M=0.40. Section 3.1.3 defines and motivates the primary analysis methods.

3.1.1 Definition of the ANC Endpoint:

Here are the details for defining the scores based on ANC measurements. First, We define the proportion of the ANC measurements (specifically, the proportion of 11 measurements: the 4 peak and 4 trough measurements done at the NIH Clinical Center [or replacement local lab] at months 0, 4, 8, and 12 and the 3 trough measurements done at the local lab) above the threshold of 500 cells/μL (proportion of measurements above threshold=PMAT). Let that proportion for the period on plerixafor be PMAT(P), and similarly let the proportion for the period on G-CSF be PMAT(G). Missing measurements will be handled in the following way. Since the study is finished, we know there are only four reasons for missing data in the study (see Table 1):

(1) patient termination due to drug failure or severe adverse event,
(2) patient missed primarily due to health of the patient,
(3) patient missed a visit primarily due to a scheduling conflict, or another reason unrelated to the health of the patient,
(4) investigator error in scheduling a test,
(5) missed visit due to COVID-19.

For ease of exposition, we refer to reason (1) as “missing due to treatment intolerance”, we refer to reason (2) as “missing due to patient health”, and we refer to reasons (3), (4), and (5) as “missing due to scheduling issues”. Missing ANC measurements due to treatment intolerance will be treated as a failure, and that ANC value and all subsequent ANC values within that period will be treated as failures. Missing due to patient health will be treated as failures only for the visit missed. Missing ANC values due to scheduling issues (i.e., reasons (3), (4), and (5)) will not be counted in the proportion. For example, if a subject misses one visit due to a scheduling issue, then the PMAT(P) will be the proportion of the 10 non-missing measurements above the threshold. If a participant misses more than 5 ANC values in any one period, then that participant will be removed from the analysis for the ANC endpoint. We define a successful treatment on plerixafor for a subject as having PMAT(P)>=0.75. Defining success this way ensures that an effect must be durable (since at least 75% of the measurements taken throughout the 12-month period must be above the threshold), yet it allows for non-perfect control, since even partial control is useful. Let I_i(success,P)=1 if the ith individual succeeds in the P arm, and I_i(success,P)=0 otherwise, and similarly define I_i(success,G)=1 if the ith individual succeeds in the G arm, and I_i(success,G)=0 otherwise. Let S_i be the score for the ith individual, which is the difference in indicators of success: S_i = I_i(success,G) – I_i(success,P). Supplemental Section S1 gives a worked example (including R code) for calculating the ANC score using simulated responses.

3.1.2 Noninferiority Margins Motivation for the Noninferiority Margin

Let Pr(succ, P) be the probability that a randomly chosen individual in the study population would have success on P (as defined in Section 2.1). Similarly define Pr(succ, G). The hypotheses will be based on the difference in the two success probabilities: Δ = Pr(succ, G)-

Pr(succ,P). We will test the noninferiority null hypothesis H0: $\Delta \geq M$, against the alternative H1: $\Delta < M$, where the noninferiority margin is M=0.40.
We now explain the noninferiority margin, M. Typically, the margin is defined based on a percentage of the treatment effect of the control drug (G) compared to placebo (see FDA Guidance on Noninferiority Trials, 2016, p. 30, Section D). Our proposal of M=0.40 is based on 50% of an estimated treatment effect of G compared to placebo of 0.80. Both the control treatment effect and the percentage chosen depend on the application. Thus, there are two distinct steps in choosing the noninferiority margin. First, we estimate the treatment effect of the control (G versus placebo). Second, we decide on what percentage of that effect is acceptable for the margin.

Consider first the estimation of the treatment effect of G-CSF compared to placebo. Although that drug is approved, many of the clinical trials used for its approval were on cancer patients receiving chemotherapy. We know of no randomized trials of WHIMS patients comparing G-CSF and placebo. Because of this we propose using data from Dale et al (1993) which was a randomized study comparing G-CSF to delayed start of G-CSF in a population with severe chronic neutropenia. In that study, all 120 of the patients started out with severe chronic neutropenia, and 90% (108) had a complete response on G-CSF after starting on G-CSF therapy. We treat this as a paired study where each patient has two responses, their response under no therapy (the delayed time, before any therapy was started) and their response under G-CSF. We use the delayed time with no treatment as an estimate of a placebo effect. We estimate Pr(succ,G) – Pr(succ,placebo) and get an exact 95% confidence interval by treating the data as paired binary responses and using the method of Fay and Lumbard (2020) and the *mcnemarExactDP* function in the *exact2x2* R package. The estimate is 0.90 (=108/120 – 0/120) with 95% confidence interval (0.806, 0.947). To be conservative, we use 0.80 for the treatment effect of G over placebo. Although the definition of success was different from that study (median ANC >= 1.5 X 109/L) and the population was different, we will use that number, 0.80, as our estimate of the effect of G-CSF compared to placebo for our proposed definition of success. In other words, we assume that in the WHIMS population defined by our study, the difference between the probability that an individual will be successful on G-CSF minus the probability that an individual will be successful on placebo will be at least 0.80, where success is defined in Section 2.1. Another justification for a treatment effect of G-CSF of at least 0.80, is that for our study 18/19 had baseline ANC < 500 cells/µL and responded to G-CSF prior to randomization with ANC>500 cells/µL, while only 1/19 had baseline ANC>500 cells/µL as well as ANC>500 cells/µL when on G-CSF prior to randomization. Treating the baseline as a surrogate for a placebo arm, we estimate Pr(succ,G) – Pr(succ,placebo) using 19/19 – 1/19 = 0.947. This estimate is also greater than 0.80 (although estimated with a smaller sample size).

Now consider the problem of the percentage of the control treatment effect that is acceptable for setting the margin. The FDA (2016) Guidance on Non-inferiority Trials (p. 30, Section D) states the margin is usually based on a percentage of the treatment effect of the control compared to placebo. Our proposed percentage is 50%, which is the traditional percentage that is used in cardiovascular trials. FDA (2016, p. 28) states "…in large cardiovascular studies, it is unusual to have [a margin, M,] that reflects a loss of less than 50% of the control drug effect,

even if this might be clinically reasonable, because doing so will usually make the study size infeasible." Because this is a very rare disease, we have a similarly motivated need for a wide margin. Additionally FDA (2016, p. 30, Section D) states that wide margins are acceptable when (1) the endpoint does not involve an irreversible outcome such as death, or 2) the test product (in our case, plerixafor) is associated with fewer serious adverse effects or better tolerability than other therapies already available, or 3) the test product has another advantage over available therapies that warrants use of a less stringent margin. Thus, since plerixafor has other advantages over G-CSF (e.g., better outcomes with respect to infection incidence and severity and wart response, and less frequent bone pain as a side effect; see McDermott, et al 2014 and McDermott, et al 2019), then this margin should be acceptable. Further, this secondary endpoint will only be tested if there is a significant treatment effect on the primary endpoint, total infection severity score.

3.1.3 Noninferiority Analysis Method

To test the hypotheses, we will use 95% exact central confidence intervals on Δ with their compatible p-values, as detailed in Fay and Lumbard (2020). Thus, the one-sided p-value for testing the noninferiority hypothesis will reject the null if the one-sided p-value is less than or equal to p=0.025, which will occur if and only if the upper 95% confidence limit is less than M. Consider first the case when the success in each treatment-period is clear, meaning there is no missing data due to scheduling issues (recall that missing ANC data due to treatment intolerance or patient health is a failure, so is not treated as missing, see Section 3.1.1). Under the clear success situation, the Fay and Lumbard method can be shown to be valid, meaning it retains the type I error rate to be less than α=2.5% and both one-sided error rates on the confidence interval are bounded at 2.5%. The details on the validity with clear successes are in Fay and Lumbard (2020, p. 5, Section 4), where calculations using the *exact2x2* R package (the same software that will be used in the final analysis) show that regardless of the true parameter values Pr(succ, G) and Pr(succ,P), the 95% exact central confidence interval for Δ will not have lower error greater than 2.5%, nor upper error greater than 2.5%. A graphical representation showing both confidence interval errors are less than 2.5% when n=19 and when the successes are clear is given in Supplemental Section S4.

It is more difficult to show the validity of the Fay-Lumbard method when the successes are not clear, meaning that there is some missing data due to scheduling issues. In this case, we demonstrate the validity using a simulated model. In Supplemental Section S3 we describe the simulation model for the ANC values. Briefly, that model uses lognormal distributions for the ANC values, with changes in the geometric mean due to peak vs trough and due to treatment. The model has a certain proportion of the subjects that cannot tolerate each treatment, and for simplicity that toleration is assumed independent of the ANC values. Finally, the model has a certain proportion of the ANC visits (one of the 4 NIH visits or of the 3 local measurements) that are independently missing due to scheduling issues. Because the missing ANC measurements due to scheduling issues are independent of all other variables (including the missing ANC values themselves), they are missing completely at random (MCAR).

The first simulation considers a case where Δ=0.40, which is the margin. We find that model in the following way. First, for our study, 3/19 were missing one arm for treatment intolerance, and 1/19 was missing both arms for treatment intolerance. Although the data are still blinded, we can model a worst-case scenario where 16% (3/19) of the population cannot tolerate plerixafor and can tolerate G, and 5% (1/19) of the population cannot tolerate either plerixafor or G. We then find the treatment effect on ANC such that the resulting value for Δ equals the margin M=0.40. For details see Supplemental Section S3.1. Using that parameterization, we add on missingness due to scheduling issues (which is MCAR). For this first simulation, we set the proportion missing visits due to scheduling issues to equal 6% (this is similar to the actual proportion missing for that reason). Then we analyze the data as we have proposed (so that missing for treatment intolerance is set to failure and missing due to scheduling issues is removed from the proportion calculations). The simulation rejects the null hypothesis 2.27% out of 10,000, which is less than or about equal to the nominal 2.5% (95% CI on simulated rejection rate: 1.99%, 2.58%). The details are in Supplemental Section S3.3.

The next set of simulations demonstrates that treating the missing data that is MCAR as failures may inflate the type I error rate. We use the same set of parameters that give Δ=M=0.40 as described in Section S3.1, but now we assume that 20% of the ANC visits are missing due to scheduling issues (i.e., missing completely at random). For this set of simulations we analyze the data in two ways: first, as proposed, where we do not count missing due to scheduling issues in the proportions, and second, setting to failures the ANC values missing due to scheduling issues to failures. We find that doing the analysis as proposed retains the type I error rate, rejecting 2.21% of the 10,000 replications (close to the nominal 2.5%; 95% CI: 1.93%, 2.52%), while treating the MCAR missing data as failures does not retain the type I error rate, rejecting 12.06% of the 10,000 replications (much greater than the nominal 2.5%; 95% CI: 11.4%, 12.7%). Details are in Supplementary Section S3.4.

It may seem counter-intuitive that setting the missing values to failure is not conservative. That approach may be conservative in a typical superiority trial; however, recall that the analysis based on ANC is a non-inferiority analysis. In a superiority trial, if missing values in both arms are set to the same value when missing for a reason unrelated to the response then this will bring the average response in both arms closer together and make the study less likely to falsely claim superiority. For a non-inferiority analysis, replacing missing values with the same value is not a conservative strategy because that will bring the average response (proportion of successes) in the two arms closer together and make the non-inferiority null hypothesis easier to reject. This easier rejection is because bringing the proportion of successes closer together will bring the estimate of Δ closer to 0, and 0 is well within the alterative hypothesis space (recall the alternative hypothesis is H1: Δ< 0.40). In other words, treating the missing due to scheduling issues as failures will inflate the type I error rate in this situation. See FDA guidance on non-inferiority trials for a more general discussion of this issue (FDA, November 2016, p. 31, Section F).

3.2. Success on ALC: Proportion of ALC Above Threshold at least 75%

We measure the endpoint of ALC similarly to how it was measured for ANC but with some differences. We define success within a period as having the proportion of peak ALC values above a threshold of 1000 cells/microliter equal to 75% or higher, and the endpoint is the difference in indicators of success.
Here are the details. Let the proportion of NIH-Clinical Center peak ALC measurements above a threshold on each treatment be PMAT(P) and PMAT(G). We define the threshold as 1000 cells/microliter (the lower limit of normal for absolute lymphocyte count). We use only the peak ALC data on the 4 visits done at the NIH Clinical Center. The literature has shown (McDermott, Liu, et al, 2011, McDermott, et al 2014, 2019) that the ALC trough after a dose of plerixafor approximates the baseline, and that the ALC is not affected significantly by G-CSF. Therefore, the only meaningful measurement that we have made to demonstrate an effect of drug on ALC is the peak. We handle missing data the same way as was done for the definition of PMAT for ANC. Thus, individuals that are missing data due treatment intolerance or patient health will be treated as failures, and the missing due to scheduling issues (as defined in Section 3.1.1) will be treated as missing completely at random and not counted in the proportions. Then we can define a successful treatment during a treatment period as having PMAT≥0.75. For the ith individual, let I_i(succ,G)=1 if successful in the G period, and I_i(succ,G)=0 otherwise, and similarly let I_i(succ,P)=1 if successful in the P period, and I_i(succ,P)=0 otherwise. The score for the ith individual is
$S_i = I_i(succ,G) - I_i(succ,P)$.

We use a superiority hypothesis, tested at the one-sided 2.5% level. The null hypothesis is Pr(succ,G)-Pr(succ,P) ≥ 0 versus the alternative hypothesis that Pr(succ,G)- Pr(succ,P) <0, where Pr(succ,G) and Pr(succ,P) denote the true probability that an individual will be counted as a success with respect to being above the ALC threshold under each treatment.

We test whether there is a significant difference in the proportion of subjects with success under P compared with success under G using an exact one-sided McNemar's test (a paired test for binary responses). We will use the confidence intervals of Fay and Lumbard (2020) for this effect estimate that are compatible with the exact McNemar's test. As with the primary endpoint with ANC, we use the *mcnemarExactDP* function in the *exact2x2* R package. Because the test is exact, it is valid for all sample sizes even if there are only 4 or fewer measurements per subject per arm used to define the sign for each subject. Having few observable measurements may affect the power of the test, but it will not affect the validity.

3.3. Incidence of Infection

The incidence of infection will be measured like the TISS (see Section 2), except instead of measuring infection severity, we will only measure an indicator of infection (yes/no). The

analysis and methods will be the same as for the TISS endpoint, except instead of assigning each infection with a severity score, it will be assigned a score of 1.

3.4. Days of Oral Antibiotic/antifungal/antiviralmight

The next secondary endpoint is the number of days on a prescribed medication with an antibiotic, antiviral, or antifungal (oral or IV). Topical treatments or treatment through eye drops will not count. If more than one medication is prescribed during the same day, that will count only as one day on treatment. The duration of antibiotic treatment is the number of days a subject is prescribed treatment with an antibiotic/antiviral or antifungal (oral or IV), regardless of whether the subject actually took the prescribed treatment. A day where there is no record of a prescribed treatment will be counted as not receiving any prescribed treatment on that day. The number of days on treatment will be determined prior to the unblinding of the data.

To compare treatment arms, for each patient the response is the number of days on prescribed medication in the year on G minus the number of days on prescribed medication in the year on P. We will use a one-sided exact Wilcoxon signed-rank test (e.g., *wsrTest* in the *asht* R package), which tests the null hypothesis that the difference is less than or equal to 0 (and equal or more medication is prescribed on the P arm than on the G arm) versus the alternative hypothesis that the difference is greater than zero (and more medication is prescribed on the G arm than on the P arm). This will be an intent-to-treat analysis, comparing periods randomized to G versus periods randomized to P, so if a participant cannot tolerate a treatment during the period assigned to it, they will still be counted as on that treatment.

3.5. Quality of Life Scores as Defined by a 36 Point Questionnaire (SF-36)

The subjects will complete a validated Quality of Life (QOL) questionnaire (SF-36 version 2) during the 4, 8, and 12 month visits in the Treatment period at the NIH-CC, and the non-missing responses will be averaged and compared (average on G minus average on P). This will be an intent-to-treat analysis, so anyone that is allocated to a treatment during a period will be counted for that treatment regardless of whether they continue their treatment over the entire

period. A one-sided exact Wilcoxon rank-sum test (also called a two-sample Wilcoxon paired difference test) will be used for the analysis.

4 Exploratory Endpoints

Other endpoints may be examined as exploratory. We discuss one possibility here, the change in wart burden, but other exploratory analyses may be done.

Existing warts will be documented at baseline visits prior to treatment with either the study drug or the comparator agent via clinical photographs, if the subject consents. Every 4 months during the first treatment period clinical photography will be repeated in areas with new or existing warts. Our main endpoint will be the ratio of the wart burden at the end of the first treatment period over the wart burden at baseline, where the wart burden is determined by blinded dermatology judges. The wart burden is measured by total area of affected skin, so that the ratio is (final wart area)/(baseline wart area), where final wart area is the area of the warts at the end of period 1. We compare the two groups in the first period by comparing the geometric means of the ratios in the two groups only among those who had any baseline warts
Statistical Analysis Plan

recorded. If any final wart burden areas are not measured, we will use the last recorded measurement of wart burden instead. The second period data will not be used in this comparison because there will likely be carryover effects. We compare these first period GM ratios using a ratio of ratios: (GM ratio Group P)/(GM ratio Group G). We use a two-sample t-test (Welch's version) on the log of the ratios with the associated confidence interval on the difference in mean log ratios transformed back into the ratio of GM ratios.

References

Fay, MP and Lumbard, K (2020). Confidence Intervals for Di¨erence in Proportions for Matched Pairs Compatible with Exact McNemar's or Sign Tests. (unpublished manuscript).

FDA (2016). Non-Inferiority Clinical Trials to Establish Effectiveness: Guidance for Industry. November 2016.
FDA (2017). Multiple Endpoints in Clinical Trials: Guidance for Industry. January 2017.

McDermott, D.H., Liu, Q., Velez, D., Lopez, L., Anaya-O'Brien, S., Ulrick, J., Kwatemaa, N., Starling, J., Fleisher, T.A., Priel, D.A.L. and Merideth, M.A. A phase 1 clinical trial of long-term, low-dose treatment of WHIM syndrome with the CXCR4 antagonist plerixafor. Blood, The Journal of the American Society of Hematology, 123(15), pp.2308-2316. 2014.

McDermott, D.H., Liu Q., Ulrick J., Kwatemaa N., Anaya-O'Brien S., Penzak S.R., Filho J.O., Priel D.A., Kelly C., Garofalo M., Littel P., Marquesen M.M., Hilligoss D., Decastro R., Fleisher T.A., Kuhns D.B., Malech H.L. and Murphy P.M. The CXCR4 antagonist plerixafor corrects panleukopenia in patients with WHIM syndrome. Blood 118(18): 4957-62. 2011.

McDermott, D.H., Lopez J., Deng F., Liu Q., Ojode T., Chen H., Ulrick J., Kwatemaa N., Kelly C., Anaya-O'Brien S., Garofalo M., Marquesen M., Hilligoss D., Decastro R., Malech H.L. and Murphy P.M. AMD3100 is a Potent Antagonist at CXCR4R334X, a Hyperfunctional Mutant Chemokine Receptor and Cause of WHIM Syndrome. J Cell Mol Med 15(10): 2071-81. 2011.
McDermott D.H. , Pastrana D.V., Calvo K.R., Pittaluga S., Velez D., Cho E., Liu Q., Trout H.H., Neves J.F., Gardner P.J., Bianchi D.A., Blair E.A., Landon E.M., Lopes Silva S., Buck C.B., and Murphy P.M. Plerixafor for the Treatment of WHIM Syndrome. N Engl J Med 380(2): 163-170. 2019.

Table 1: Missing data counts and percentages with reasons

1) Enrollees with missing data:

	NIH	*Outside*	*Combined*
	14	11	15

2) Enrollees with each type of missing data (out of 19 enrollees):

Statistical Analysis Plan

	NIH	*Outside*	*Combined*
Patient Action (not health)	8	6	9
Patient Action (Health related)	0	1	1
Drug Off Study	4	4	4
NIH Error	4	0	4
COVID-19 Closure	1	1	1

3) Reasons for missing data (percent out of scheduled measurements):

	NIH	*Outside*	*Combined*
Patient Action (not health)	4.6%	7.9%	5.7%
Patient Action (Health related)	0	0.9%	0.2%
Drug Off Study	11.8%	12.3%	12.0%
NIH Error	1.6%	0.0%	1.2%
COVID-19 Closure	0.3%	0.9%	0.5%
All Reasons	**18.4%**	**21.9%**	**19.4%**

Definitions for reasons:

-Patient Action (not health): the primary reason for missing the lab measurement is due to some patient action (e.g., transportation difficulties, conflicting appointment, etc) that is not related to the health of the patient.
-Patient Action (health related): a primary reason for missing is the health of the patient.
-Drug off study: the patient missed because of drug intolerance, or serious adverse events.
- NIH error: labs were not ordered, drawn, or processed at the NIH. Local labs were not requested by study team.
- COVID-19 closure: unable to visit NIH or local labs closed due to pandemic.

Statistical Analysis Plan

Appendix D (Copied Verbatim from Version 6.0 of the Protocol).

APPENDIX D: INFECTION SEVERITY SCORE (ISS)

Type of Infection	***Fever***	***Anti-Infective***	***Hospitalization***	***Total***
	0: No chills/fever	0: No Treatment	0: No Hospitalization	
1: Non-sterile site	1: 38.3 -39° C	1: Topical	1: Emergency Room	
2: Sterile site	2: > 39° C	2: Oral	2: Hospitalized	
		3: Parenteral	3: ICU	
1 to 2	*0 to 2*	*0 to 3*	*0 to 3*	***1 to 10***

These parameters will be used to develop a score for each infection. Non-sterile site infections are those which occur in areas of the body routinely exposed to and colonized by microorganisms such as the oral cavity, bronchioles and upper respiratory tract, nasopharynx, vagina, GI tract, and skin; while, sterile sites would include the lower respiratory tract, blood,

muscle, bone, joints, urinary bladder, and other typically sterile locations. Fever will refer to the maximum oral temperature recorded during the infection. Anti- infective treatment is scored based on the highest level of treatment i.e. intravenous antibiotic that is changed to oral would score a 3. Similarly hospitalization will refer to the highest level of care received at any point during the infection. Scores for each parameter will be added and thus the score for any given incidence of infection can range from 1-10.

Supplementary Material to Statistical Analysis Plan for: A Phase III, Double-blind Randomized, Crossover Study of Plerixafor Versus G-CSF in the Treatment of Patients with WHIM Syndrome, Protocol Number: 14-I-0185

Summary

This is a supplement to accompany the statistical analysis plan. It contains details for how the analysis will be done, including the computer code. There are several sections describing: an example analysis, a model for simulating data sets, a simulated data set and its analysis, and several simulations or calculations to examine the properties of the statistical methods.

This is an R markdown document, which is a way to create reproducible research. The report is automatically generated from a text file that has R code embedded within it. During the computer compiling of the report, the R code is run, and the results are returned to the proper place in the report. Thus, there are 3 types of files associated with this report:

1. The .doc file is the actual report. This contains the results of the R code (statistics, graphs, etc) after it has been compiled.
2. The .Rmd file is the file that contains the report descriptions, and the R code, but not the results.
3. The .R file contains only the R script used to perform the calculations. This file can be created from within R using the purl function in the knitr R package on the .Rmd file.

S1. Example Analysis

Here is a made-up example of the ANC data for one subject. Within each treatment-period There are 11 measurements at 7 visits (t0=trough at month 0, p0=peak at month 0, t2=trough at month 2, etc.). We mark missing data as either missing due to scheduling conflicts (marked in the data as NA), or missing due to unable to tolerate the treatment or the health of the patient (marked in the data as -99). If a subject stops taking a treatment because of not being able to tolerate it, then for the purposes of the primary endpoint, all subsequent scheduled ANC measurements will be marked as -99 (i.e., unable to tolerate treatment). Thus, even if a blood sample is taken and ANC is measured later in the treatment-period after the subject has stopped taking the allocated treatment, the primary endpoint ANC will still be listed as -99, regardless of the actual ANC measured value. The value -99 denotes failure.

```
##   trt period  t0   p0  t2  t4  p4  t6  t8   p8 t10 t12  p12
## 1   G      2 560  883 775  NA  NA 600 636 1222 500 760 1171
## 2   P      1 824 1396 -99 -99 -99 -99 -99  -99 -99 -99  -99
```

Statistical Analysis Plan Supplement

For this made-up example data, in the first period (second row of data matrix) the subject got trt=P, but could not tolerate that treatment after the first visit at month 0. For that first period, there are 2 successful measurements out of the 11 scheduled measurements so the proportion above the threshold on P, PMAT(P) = 2/11 = 0.182. Here the 9 measurements missing due to intolerance (marked as -99) are counted as failures. Since PMAT(P) < 0.75, that subject is marked as a failure for P, $I(success, P) = 0$. For the second period (first row of the data matrix), the subject got trt=G. There was one missed visit at month 4 due to a scheduling conflict, and both measurements (trough and peak) are missing for month 4. Of the remaining 9 measured values, 8 were above 500 (500 counts as a failure), so PMAT(G) = 8/9 = 0.889. Since PMAT(G) ≥ 0.75, that subject is marked as a success for G, $I(success, G) = 1$. So the overall score for that subject is S = I(success,G) - I(success,P) = 1-0=1.

S2. Defining the Non-inferiority Hypotheses and Testing with Clearly Defined Successes

This section assumes a clear definition of success within each treatment period. Our hypothesis is defined using the difference in the probability of success in each of the two treatments: Pr(success,G) - Pr(success,P). We test the null hypothesis,

$$H_0: Pr(success, G) - Pr(success, P) \geq M$$

versus the alternative,

$$H_1: Pr(success, G) - Pr(success, P) < M.$$

We can break up the study population into 4 types of responders:

1. GO: Success under G only
2. PO: Success under P only
3. B: Success under both treatments
4. N: Success under neither treatment

Now consider the population parameters. Define the probabilities that a randomly selected individual in the population of interest responds in each of the 4 categories as: p_{GO}, p_{PO}, p_B, and p_N. We can write our parameter of interest as:

$$\Delta = Pr(success, G) - Pr(success, P) = (p_{GO} + p_B) - (p_{PO} + p_B) = p_{GO} - p_{PO}.$$

Consider the case where within each time period for each individual, the success is clearly defined as a binary variable. For this study, that means there is no missingness due scheduling issues (i.e., missing for reasons (3),(4), or (5) as defined in the Statistical Analysis Plan, Section 3.1.1). Then the ith individual has a pair of binary variables, and we are interested in the sign of the difference in those binary variables, say $S_i = I_i(success, G) - I_i(success, P)$. Standard

analysis of paired binary data uses McNermar's test, but for a noninferiority hypothesis we need to generalize that to allow for one-sided hypotheses with non-zero boundaries (i.e., non-zero noninferiority margins). Fay and Lumbard (2020) details how to create an exact central confidence interval based on a melding-type method. We relate our notation to that of Fay and Lumbard, who use $\theta = p_{GO} + p_{PO}$ and $\beta = p_{GO}/(p_{GO} + p_{PO})$, so that $\Delta = \theta(2\beta - 1)$. We use the same R function (the mcnemarExactDP function in the exact2x2 R package) for our analysis that was used in Fay and Lumbard. Section 4 (p. 5) of Fay and Lumbard (2020) detailed calculations that showed that for all n from 1 to 100, and for all values of $\beta \in \{0,0.01,0.02,...,1\}$ and $\theta \in \{0,0.01,0.02,...,1\}$ the 95% confidence interval for Δ based on their method had lower and upper error no larger than 0.025. We give this R code to do those calculations for $n = 19$ in Section S4 of this document. The R script for the full set of calculations is in the exact2x2 R package in the package file directory "slowTests" in the file "mcnemarExactDPtestsVerySlow.R").

Section S3 gives some simulation results, including giving the details of how one data set is analyzed.

S3. Simulating ANC data

We break the simulation section into several subsections. Section S3.1 describes the model for simulating ANC data. This includes finding parameters for that model such that (to the nearest hundredth) $\Delta = 0.40$ and the probability of failure on P but not on G is 16%, while the probability of failure on both G and P is 5%. The 16% (3/19) and 5% (1/19) values come from the sample proportions under the worst case scenario for the blinded data. Section S3.2 simulates one data set, and shows how the primary endpoint would be analyzed. Section S3.3 simulates from the parameters of Section S3.1 that give $\Delta = 0.40$ with 6% missing due to scheduling conflict (close to the actual values). We see that the simulated 95% confidence interval covers the true value over 95% of the time. Finally, in Section S3.4 we simulate when there is 20% missing ANC values due to scheduling conflict, and treat the missingness in two ways. First, we treat the missingness due to scheduling conflict as missing completely at random (MCAR, in other words, we ignore the missing values in the calculation of the PMAT values). Next, we treat the missing values due to scheduling conflict as failures. In that latter case, the type I error rate is inflated because when the missing is really MCAR (as in this simulation), there is an equally likely chance that values will be missing and set to failure in the G period as in the P period. Thus, there are more paired indicators of success where both are zero. That biases the estimate of Δ towards 0, and makes it easier to reject the null hypothesis that $\Delta \leq 0.40$.

S3.1 Model for Simulating ANC data

We simulate ANC data using the following model. We model the ANC data on the log10 scale. Let y_{itjk} be the log10(ANC) for the ith individual, for the tth treatment period (t=0 is G-CSF, t=1 is plerixafor), at the jth visit day within the treatment-period, where the measurement is either a trough ($k = 0$) or a peak ($k = 1$). The model is

$$Y_{itjk} = \mu_i + t * \phi + k * \gamma + \epsilon_{ij},$$

where the error terms are independent and normally distributed with $\epsilon_{ij} \sim N(0, \sigma^2)$, the subject specific means μ_i represent a random subject effect distributed $\mu_i \sim N(\mu, \tau^2)$ where μ repesents the mean (on the log10 scale) of ANC responses on control (G), so that 10^{μ} is the geometric mean of the trough ANC values on G. The standard deviation of the random subject effect is τ, the random error standard deviation is σ, the peak effect is γ, and the treatment effect is ϕ, representing the change in mean log10(ANC) from G to P. For the simulations, we use $\gamma = 0.3$ (i.e., the geometric mean of the peak ANC value is approximately double that of the trough ANC value), and $\sigma = 0.1$. Those parameters are defined to approximately match the ANC measurements after a few days on plerixafor, in McDermott, et al (2014, Figure 1). The parameters μ, τ and ϕ will be chosen to give different success probabilities (p_{GO}, p_{PO}, p_B, and p_N).

To be a success in a treatment period, a subject should have 9 or more ANC measurements out of 11 greater than 500 (since success is defined as PMAT ≥ 0.75, and there are 11 measurements per treatment period, and since 9/11> 0.75, but 8/11 < 0.75). Given μ_i (and σ and γ), we can approximate the probability that a subject will be marked as successful in a period. Since the peak values are typically much larger than the trough values, we can approximate the success probability as the probability of observing 5 or more out of 7 trough ANC measurements greater than 500. First, assume that all subjects can tolerate the treatment, so success is determined by ANC values only.

We calculate $Pr[X \geq 5 | X \sim Binomial(7, p)]$ where p is the probability of success for a trough measurement. From the model, given μ_i and σ, the parameter p on G is

$$p = Pr[Y_{i0j0} > \log_{10}(500)] = 1 - \Phi((\log_{10}(500) - \mu_i)/\sigma),$$

where Φ is the cumulative distribution of a standard normal random variable. For that same subject on P, the probability of success is

$$p = Pr[Y_{i1j0} > \log_{10}(500)] = 1 - \Phi((\log_{10}(500) - (\mu_i + \phi))/\sigma).$$

For example if $\mu_i = \log_{10}(600) = 2.7782$ then the probability that the ith subject will be successful on G is 0.827.

Let π_G and π_P be the probability that a subject will tolerate each of the two treatments, for simplicity we will assume that the tolerability for each treatment is independent of the ANC values on either treatment and the tolerability of the other treatment.

Given the full set of parameters (μ, σ, τ, γ, ϕ, π_G and π_P), we can calculate the probability of a subject falling into any of the 4 types of responders, GO, PO, B, or N (G only, P only, both, or neither).

We simulate using $NSIM =$ 10^{6} simulated individuals to estimate the parameters p_{GO}, p_{PO}, p_B, and p_N given the other parameters in the model. In the simulation results, pG0,pPO,pB, and

pN are the simulated proportions in the 4 groups, and pG=pGO=pB and pP=pPO+pB. Here are the results for three different sets of parameters:

```
##      ten.power.MU SIGMA TAU GAMMA    PHI PIBf PIPfo      pGO     pPO
## [1,]         1000   0.1 0.2   0.3 -0.224 0.05  0.16 0.398030 6.0e-06
## [2,]         1000   0.1 0.2   0.3 -0.225 0.05  0.16 0.399881 1.1e-05
## [3,]         1000   0.1 0.2   0.3 -0.226 0.05  0.16 0.401248 1.0e-05
##            pB       pN       pG       pP    Delta
## [1,] 0.458426 0.143538 0.856456 0.458432 0.398024
## [2,] 0.456865 0.143243 0.856746 0.456876 0.399870
## [3,] 0.455430 0.143312 0.856678 0.455440 0.401238
```

So when the margin is $M = 0.40$ then the parameters

```
## ten.power.MU        SIGMA          TAU        GAMMA          PHI
##     1000.000        0.100        0.200        0.300       -0.225
##         PIBf        PIPfo
##        0.050        0.160
```

gives a simulated estimate of Δ of 0.39987 (95% CI: 0.3989097 , 0.4008309). Thus, we can use these parameters to define a probability model on the boundary of the parameter space between the null hypothesis and the alternative hypthesis. In other words, we can use these parameters to check for a violation of the type I error rate by simulation.

S3.2. Simulate 1 Data Set

We simulate data sets using the parameters. We give the R code for the simulations. We start with a set of parameters that gives a Δ value of approximately 0.40 (i.e, at the margin, which is the boundary between the null and alternative hypotheses). We additionally simulated missing visits due to scheduling conflicts or other matters unrelated to the ANC values or the health of the subject. To start we assume that the probability for those missed visits will be 6% for every scheduled visit day, regardless of the period or treatment. This simple missingness mechanism demonstrates how the missingness of that type will be handled in the data.

Here is a simulated data set based on the parameters that give $\Delta = 0.40$ from Section 3.

```
##       ID TRT PERIOD   t0   p0   t2   t4   p4   t6   t8   p8  t10  t12  p12
##  [1,]  1   0      2  627 1963  406  500 1345  903  915 1686  822  627 1310
##  [2,]  1   1      1  478 1380  539  486  753  573  568  968  427  500 1233
##  [3,]  2   0      2 1847 4126 1074 1164 2394 1216   NA   NA 1534 1278 2099
##  [4,]  2   1      1  514  865  570  327 1145  642  394 1228  603  443  969
##  [5,]  3   0      1  741 2544  -99  -99  -99  -99  -99  -99  -99  -99  -99
##  [6,]  3   1      2  643 1088  -99  -99  -99  -99  -99  -99  -99  -99  -99
##  [7,]  4   0      1 1151 1796 1118  741 1433  548  717 1906  842  982 1069
##  [8,]  4   1      2 1145 3037  -99  -99  -99  -99  -99  -99  -99  -99  -99
##  [9,]  5   0      2 1494 5333 2397 2076 4650   NA 1886 5473 2504 1934 3888
## [10,]  5   1      1  817 1839 1170 1035 1339  819  605 1123 1213  995 2628
## [11,]  6   0      1 1597 1826 1022 1612 1817 1219 1516 2703 1032   NA   NA
## [12,]  6   1      2  719 1235  645 1047 1194  646  894 1656  750  829 1635
## [13,]  7   0      1 1050 1267   NA  712 1946  786  787 1916   NA  791 1663
```

```
## [14,]  7   1      2  307  857  419   NA   NA  366  432  656  301  312  724
## [15,]  8   0      2  858 3413 1289  900 3553  982 1275 2056  962 1145 2392
## [16,]  8   1      1  666 1352  873  901 1629  627  529 1256  729  912 1724
## [17,]  9   0      2  759 1444  535  769 1345  740  810  845   NA  830 1065
## [18,]  9   1      1 2731 3112 1648 1265 2741 1545 1107 3201 1471   NA   NA
## [19,] 10   0      1 1645 2811 1865 1374 2807 1509 1796 3569 1955 2132 4001
## [20,] 10   1      2  442 1038  698  376  755  475  499  414  529  409  829
## [21,] 11   0      2   NA   NA  419  752 1508  745  692 2386  729 1152 1107
## [22,] 11   1      1   NA   NA  706  539 1497  423  745  995  505  612 1214
## [23,] 12   0      1 1601 3419 1552 1524 2357 1537 1480 2439  929 1724 2095
## [24,] 12   1      2 1193 1827 1399 1173 2027  661  772 2251  950  987 1420
## [25,] 13   0      2 1290 2113 1272  818 1549 1569 1081 1422 1343 1153 3566
## [26,] 13   1      1  715  894  -99  -99  -99  -99  -99  -99  -99  -99  -99
## [27,] 14   0      1 1591 2912 1768  972 3481 1579 1943 1987 1506 1223 2900
## [28,] 14   1      2  389  807  850  728 1748  384  532 1133  410  672 1056
## [29,] 15   0      1  654 1934  584  641 2244  572  671 1621  720  641 1332
## [30,] 15   1      2  784 1289  -99  -99  -99  -99  -99  -99  -99  -99  -99
## [31,] 16   0      2 1941 6548 2539 2523 4235 2814 3521 7603 2082 2912 7534
## [32,] 16   1      1  615  776   NA  542 1200  644  591 1361  529   NA   NA
## [33,] 17   0      1  886 1728  943  948 1432  855 1156 1683   NA  990 1048
## [34,] 17   1      2   NA   NA 1104  784 1768  800  815 2225  869  681 2240
## [35,] 18   0      1 1336 1421   NA 1108 3610  941 1188 2612 1421 1050 1552
## [36,] 18   1      2 2466 1793 1126  813 1960 1126  624 2091  674 1433 2077
## [37,] 19   0      2   NA   NA  562  548 1186  519  747 1118  595  681  695
## [38,] 19   1      1  734 1581  490  594 1112  809  653 1414  475  804 1510
```

We can calculate the scores for each subject (see the scoring example section). Here are the scores for the simulated data set:

```
##  id=1  id=2  id=3  id=4  id=5  id=6  id=7  id=8  id=9 id=10 id=11
##     1     1     0     1     0     0     1     0     0     1     0
## id=12 id=13 id=14 id=15 id=16 id=17 id=18 id=19
##     0     1     1     1     0     0     0     0
```

Then we use the Fay and Lumbard (2020) method to get the estimate of Δ with 95% confidence interval. Here are the results for the simulated data set.

```
##    estimate     lowerCL     upperCL one.sided.p two.sided.p
##   0.4210526   0.0881591   0.6649944   0.6674810   1.0000000
```

S3.3 Simulate Coverage of Confidence Interval Method

We simulate when we have 6% missingness due to scheduling, and we treat that data as missing completely at random (and hence do not use those missing values in calculating the proportions in the PMAT calculations). This simulation has 10^{4} replications. Here is a plot of the confidence intervals from the 10^{4} simulated data sets with a true $\Delta = 0.40$ (red dotted

line).

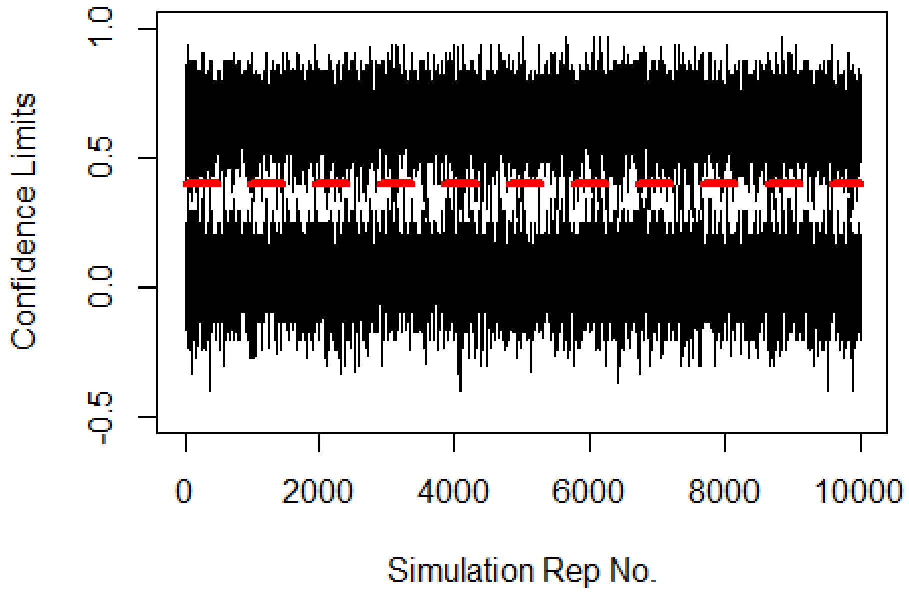

The simulated coverage is 0.9785, with the simulated lower error equal to 0.0006 and simulated upper error equal to 0.0209. The upper error rate is the important one for our hypotheses, so we give the 95% confidence interval on the upper error rate: the estimate is 0.0209 with 95% CI: 0.0182, 0.0239.

S3.4. Simulate Setting Missing for Scheduling to Fail

We repeat the simulation, but now we set the missing due to scheduling to 20%, and we set those missing values to failures. This simulation has 10^{4} replications.

Here is the plot of the simulation when missing due to scheduling conflict are deleted from data set:

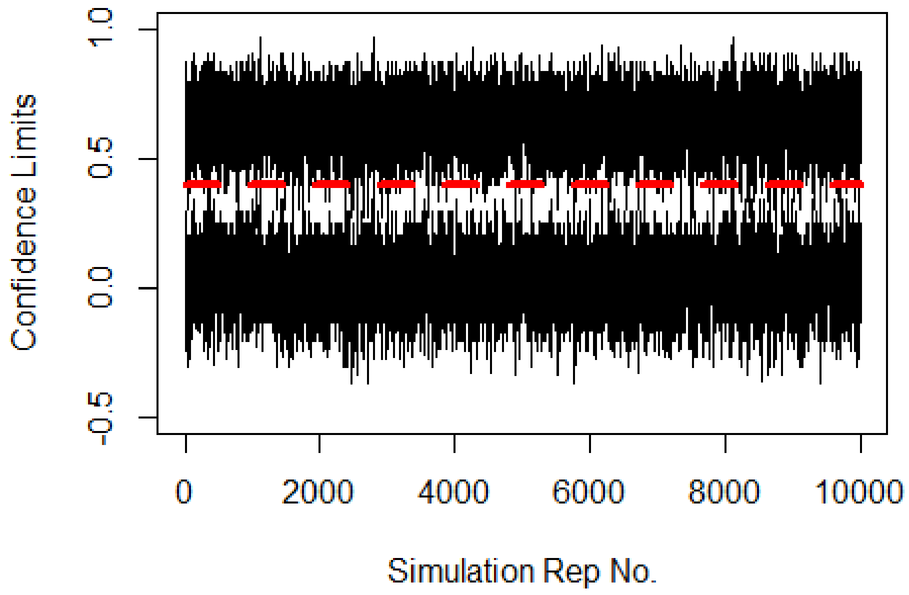

Here are the simulation stats (proportion out of 10^{4} replications):

```
## coverage lowerErr upperErr
##   0.9765   0.0003   0.0232
```

The upper error rate is approximately the nominal 0.025, simulated estimate is 0.0232 with 95% CI: 0.0203, 0.0263.

Here is the plot of the simulation when missing due to scheduling conflict are set to failures:

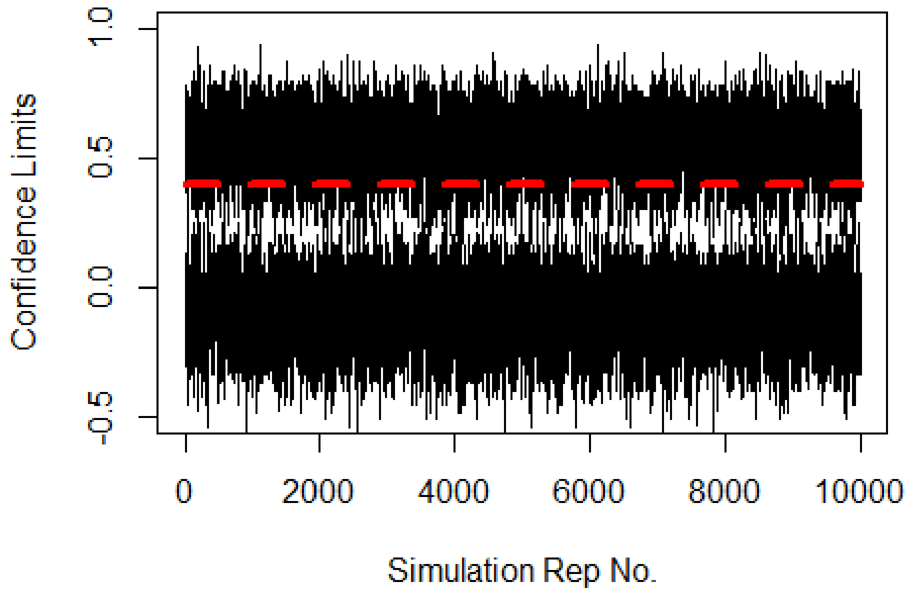

Here are the simulation stats (proportion out of 10^{4} replications):

```
## coverage lowerErr upperErr
##   0.8753   0.0000   0.1247
```

The upper error rate is much larger than the nominal 0.025, simulated estimate is 0.1247 with 95% CI: 0.1183, 0.1313.

S4. Calculation Showing the Validity of the Confidence Interval when Success Definitions are Clear

Using the mcnemarSim function (copied from the exact2x2 R package in the package file directory slowTests in the file mcnemarExactDPtestsVerySlow.R), we calculate the maximum lower error (the probability that the lower confidence limit is greater than Δ) and maximum upper error (the probability that the upper confidence limit is greater than Δ) for the 95% central confidence interval and see that they are less than 2.5%.

From the calculation, the maximum lower error of any of the values of β and θ tried was:

```
##  Beta Theta LowErrorProb
##  0.00  0.94   0.02429942
##  0.06  1.00   0.02429942
```

and the maximum of the upper error of any of the values of β and θ tried was:

```
##  Beta Theta HighErrorProb
##  1.00  0.94    0.02429942
##  0.94  1.00    0.02429942
```

References

Fay, MP and Lumbard, K (2020). Confidence Intervals for Difference in Proportions for Matched Pairs Compatible with Exact McNemar’s or Sign Tests. (unpublished manuscript).

McDermott, David H., et al. "A phase 1 clinical trial of long-term, low-dose treatment of WHIM syndrome with the CXCR4 antagonist plerixafor." Blood, The Journal of the American Society of Hematology 123.15 (2014): 2308-2316.

Summary of Amendments to the Protocol (deposited at www.clinicaltrials.gov, identifier # NCT02231879)

1. April 2015:
   - Eliminate exclusion criteria for patients with history of hematopoietic cancer (sec 4.3).
   - Editorial changes for consistency and clarity (sec 6.3.12, 4.4.2, 6.4.5, 12.3 Appendix A).
   - Operational efficiencies: Simplify labs drawn at patient's home locale (Complete Chemistry components at home locale need not precisely match NIH's chemistry lab; ESRs are no longer required and may be replaced with CRPs). Eliminate requirement for Height measurement at every NIH visit. Eliminate need for vital signs and blood draws at 3 hours post start of study drug – Day 0.
2. June 2016 (no change to protocol, addendum to consent):
   - Describes possible contamination of study syringes. Follow up investigation failed to demonstrate contamination.
3. October 2016:
   - Personnel Changes: removed consultant Dr. Stratton because she was no longer an employee of the NIH, replaced pharmacist with Michael Kolf in place of George Grimes and added Elena Cho as study coordinator.
   - Descriptive Changes regarding manufacture and supply of study syringes (sec 5.2 & 5.3, 5.6, 11.7),

- Clarification regarding Recording the Quantity and Severity of Warts, adding that clinical photography is required at each visit “if applicable” since some enrollees do not have warts (sec 7.7)

4. June 2017:

- Describe Unexpected Adverse Events in the study – psoriasis, arthralgia, and reactive arthritis (sec 1.5, 8.1, 8.2).
- Editorial change describing Time of Day Measurement for ANC (sec 7.1.1).

5. May 2018 (no change to protocol): sharing of study samples.
