# Supplementary material for: A phase III randomized crossover trial of plerixafor versus G-CSF for treatment of WHIM syndrome
Source: J Clin Invest. 2023 Oct 2;133(19):e164918. doi: 10.1172/JCI164918 (PMC10541188; doi:10.1172/JCI164918)
Supplement: Supplemental figure 7 [file jci-133-164918-s100.pdf]

# M01 L sole Acetyl Salicylic Acid pretreatment (5/2014→3/2015)

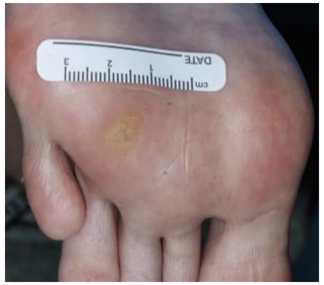

5/2014

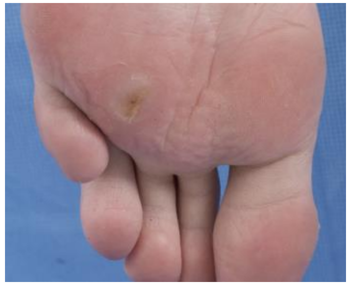

G d0 2/4/15

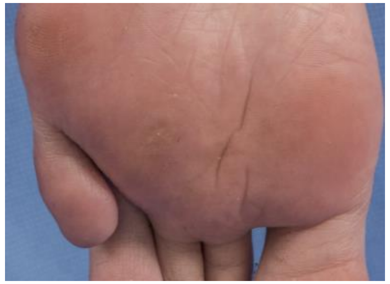

G M0

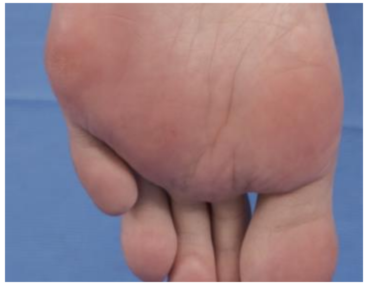

G M4

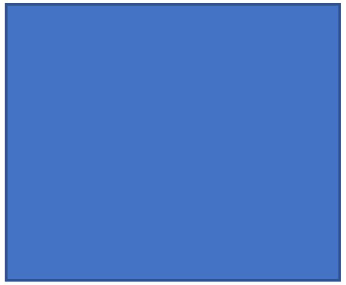

G M8

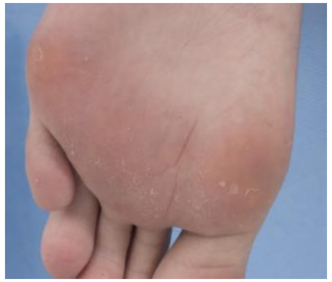

G M12

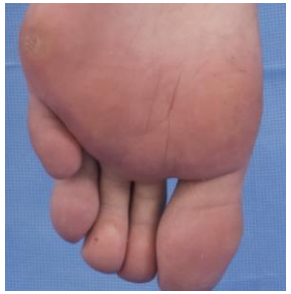

P M0

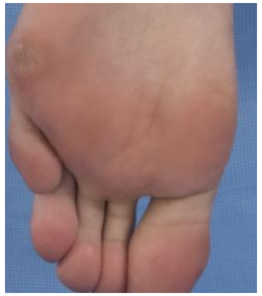

P M4

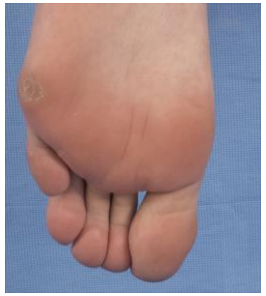

P M8

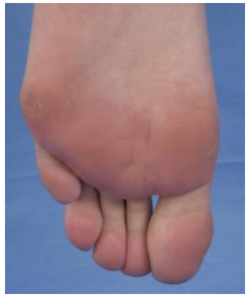

P M12

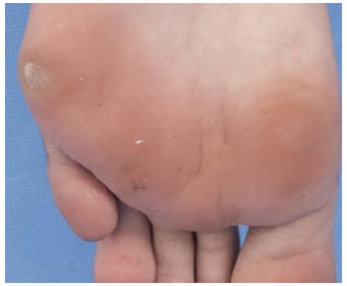

1/2018

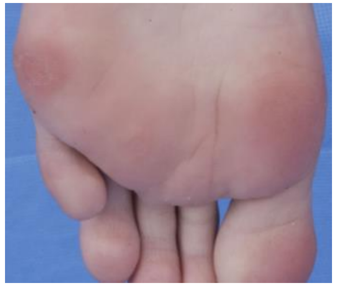

10/2019

# M01 Forehead

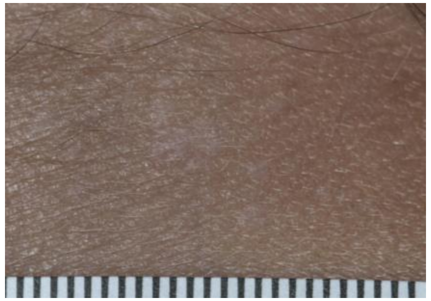

5/2014

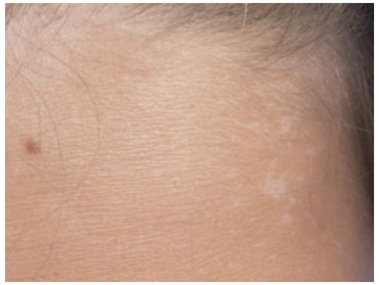

G d0
10/14/14

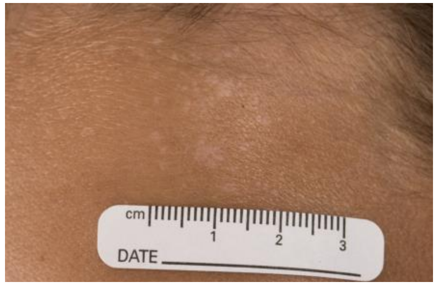


G M0

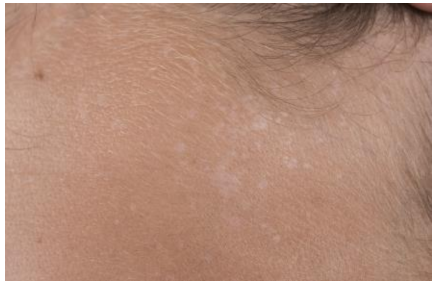

G M4

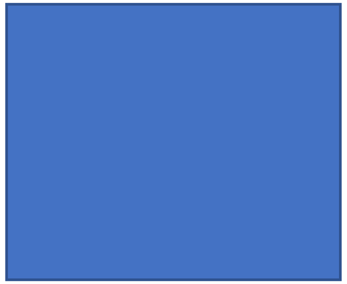

G M8

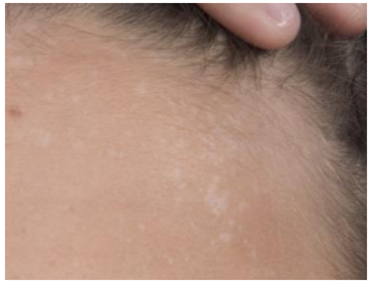

G M12

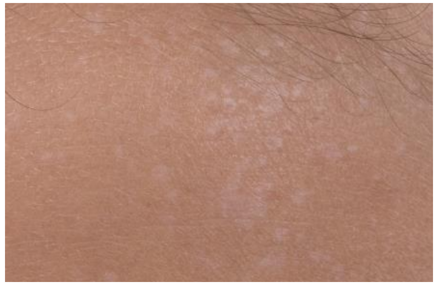

P M0

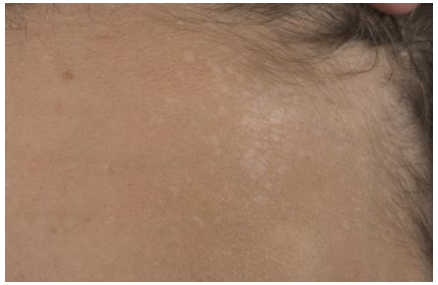

P M4

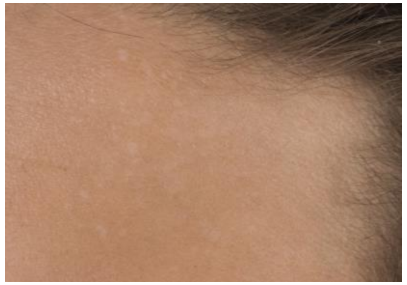

P M8

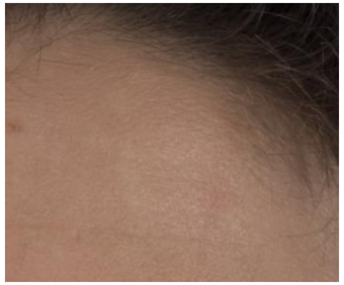

P M12

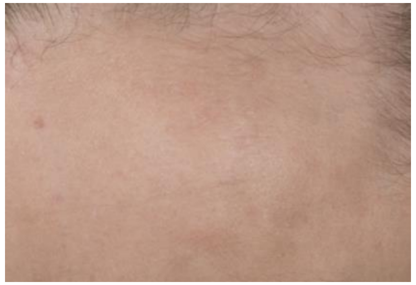

1/2018

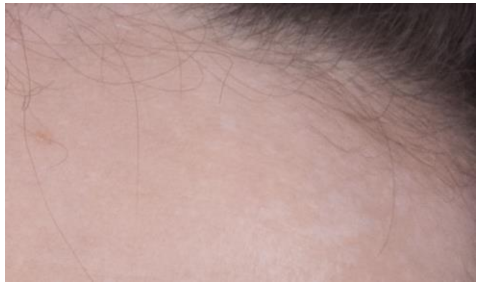

10/2018

# M01 Right sole

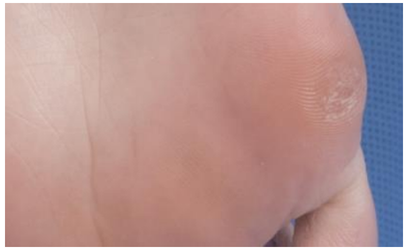
G d0

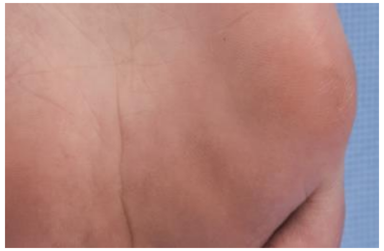
G M0

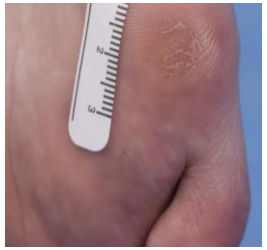
G M4

G M8

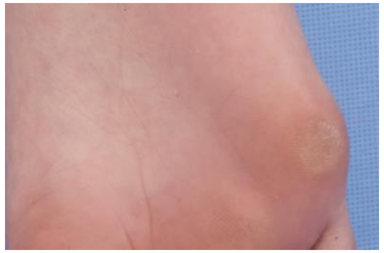
G M12

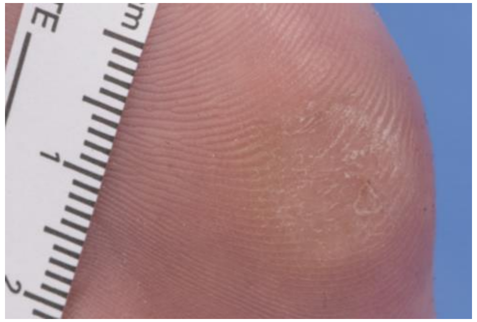
P M0

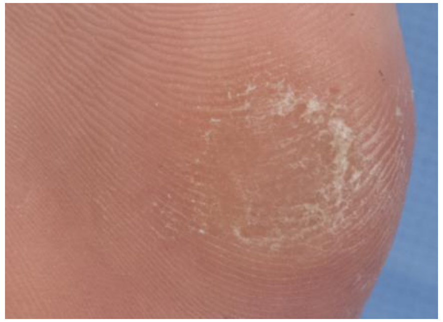
P M4

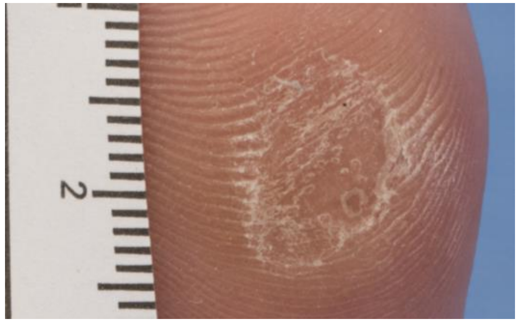
P M8

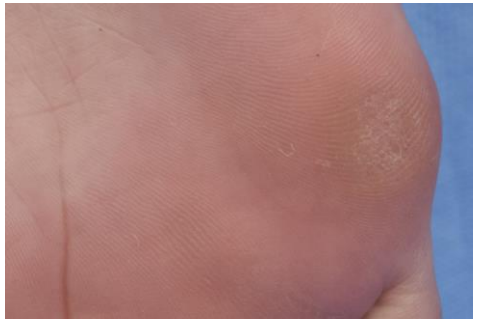
P M12

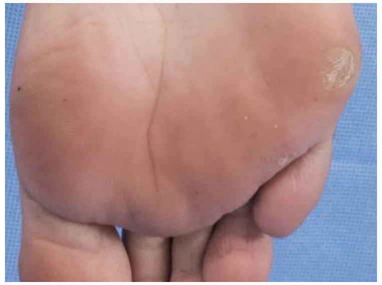
1/2018

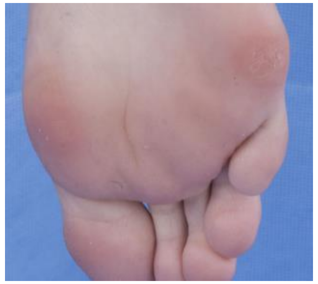
10/2019

# M02 Right palm

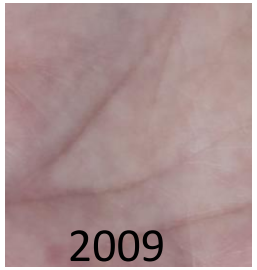


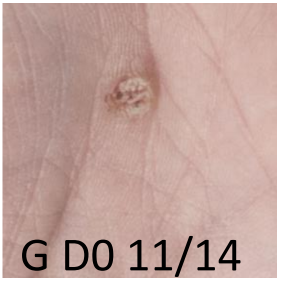


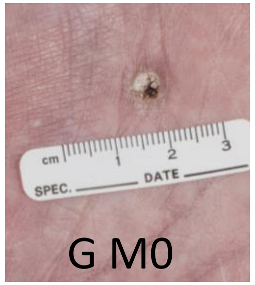


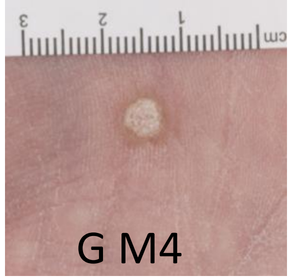


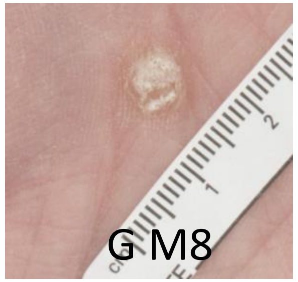


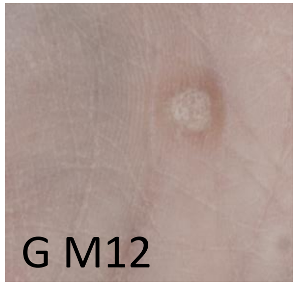


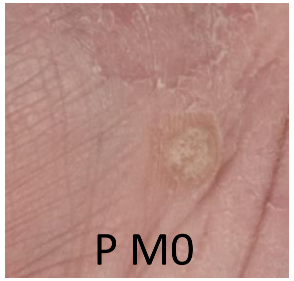


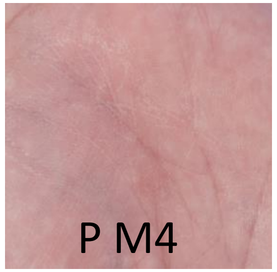


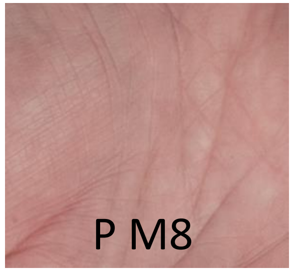


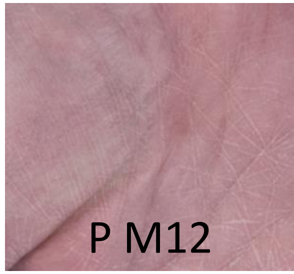


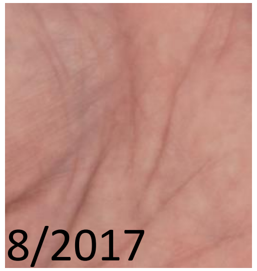


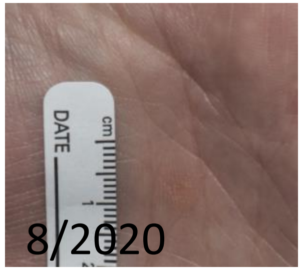

# M02 Right hand, finger 2, PIP and DIP joints

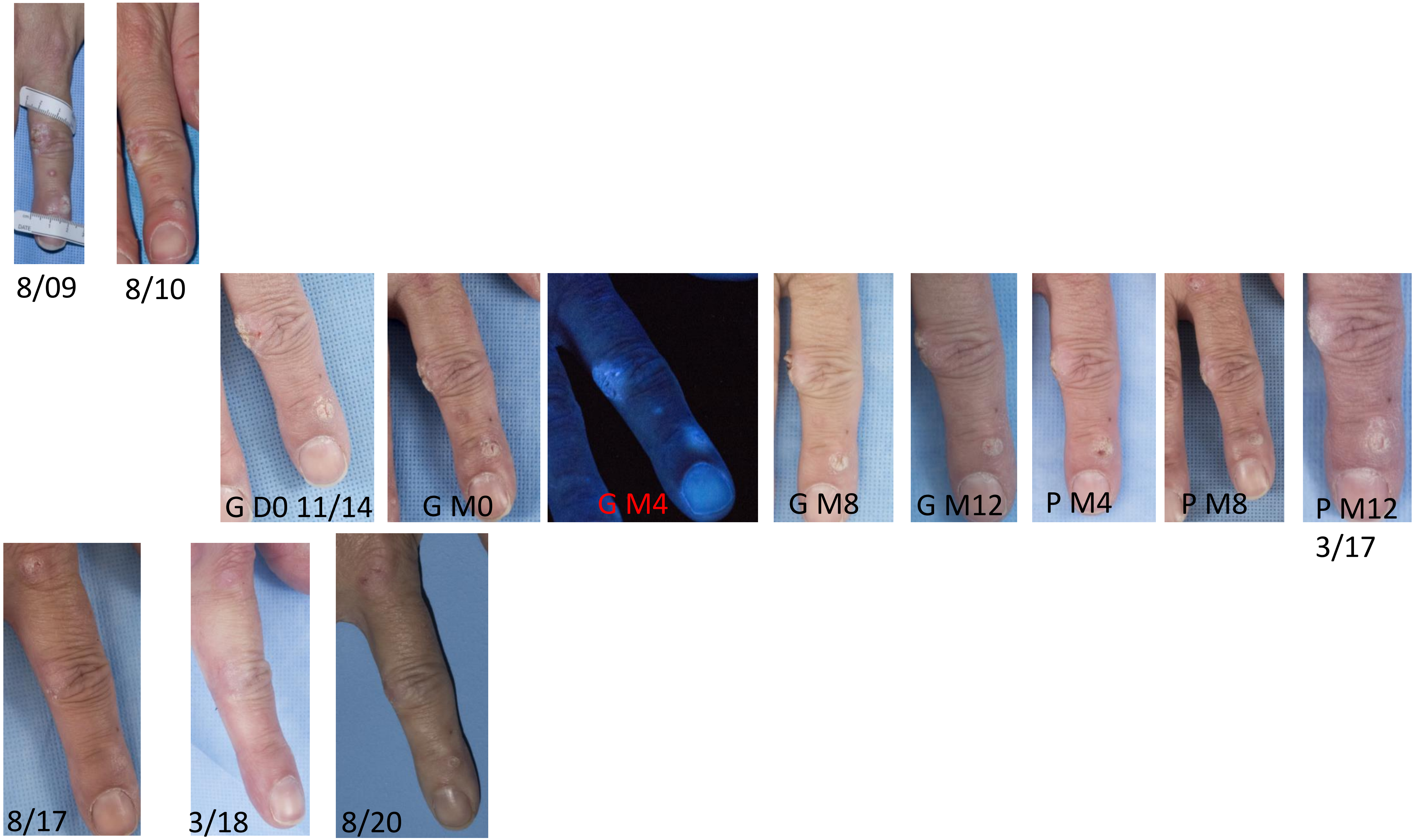

# M02 Right hand, finger 5, DIP joint

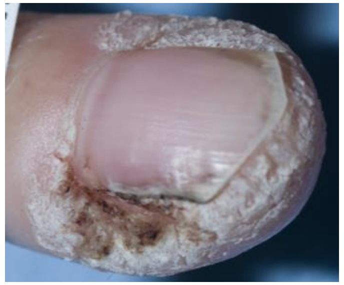

1/09

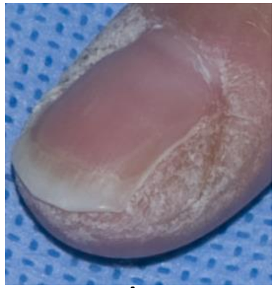

8/09

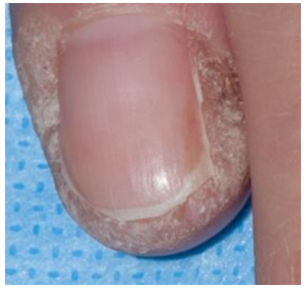

8/10

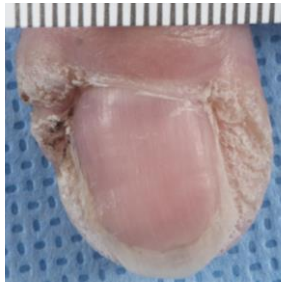

G d0

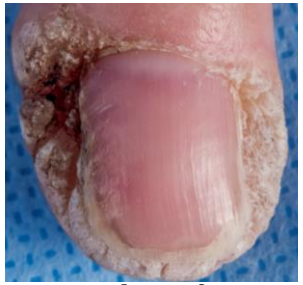

G M0

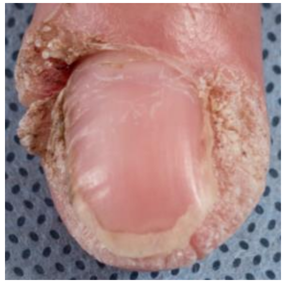

G M4

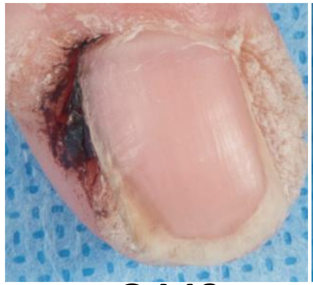

G M8

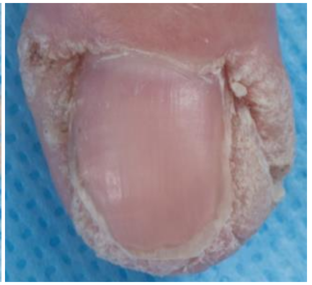

G M12

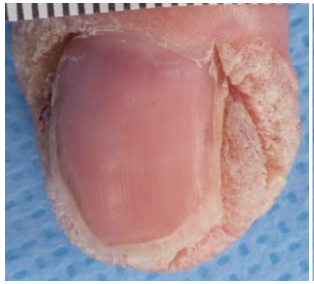

P M0

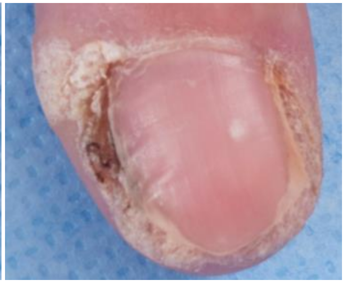

P M4

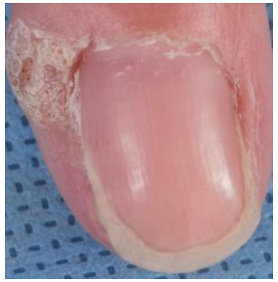

P M8

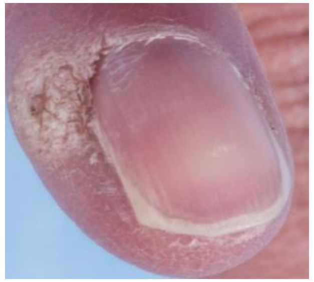

P M12

3/18

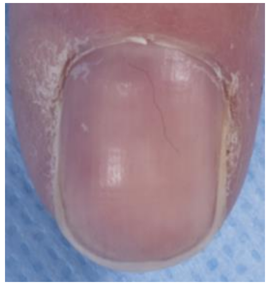

8/20

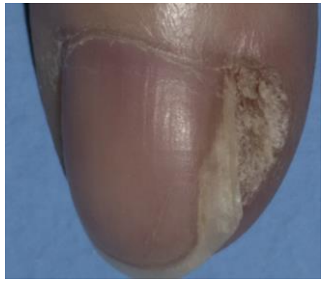

# M02 Left hand, finger 5, DIP region

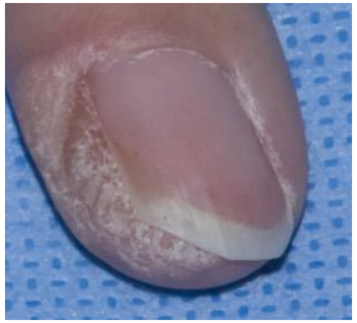
8/09

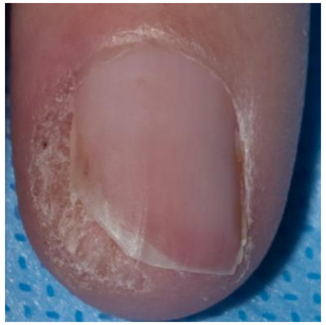
8/10

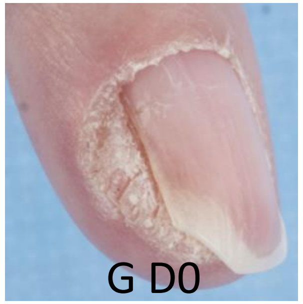
G D0
11/14

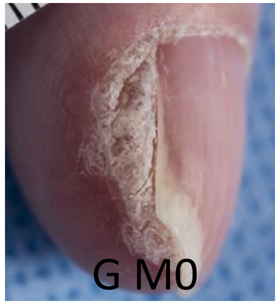
G M0

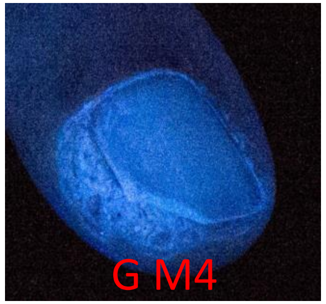
G M4

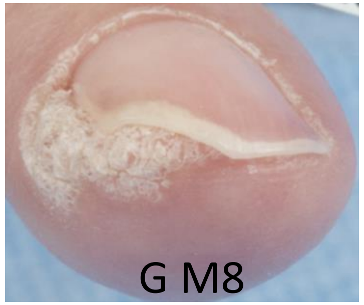
G M8

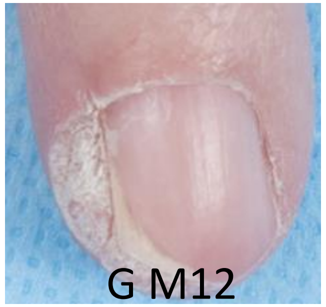
G M12

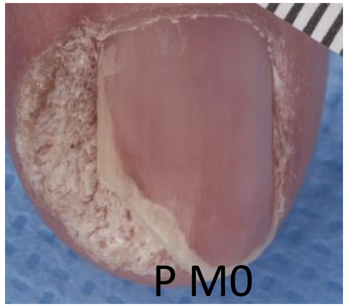
P M0

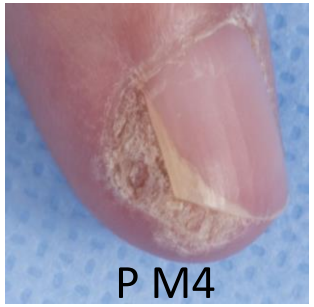
P M4

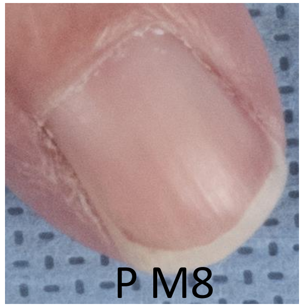
P M8

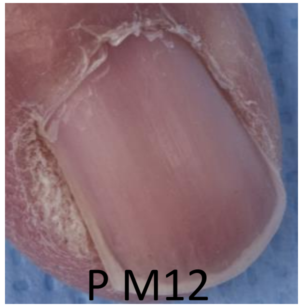
P M12
3/17

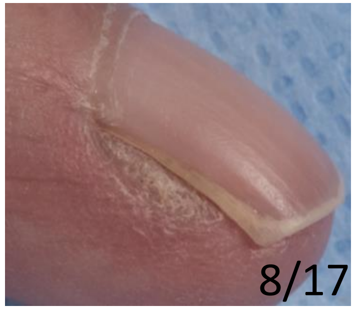
8/17

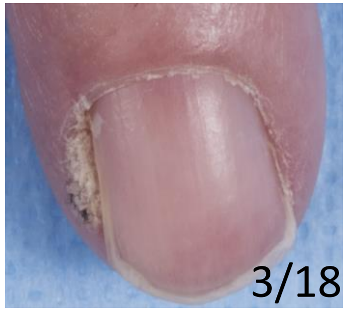
3/18

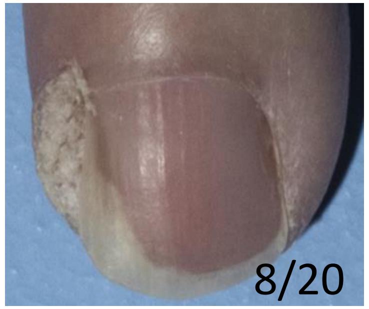
8/20

# M02 Left knee, posterior

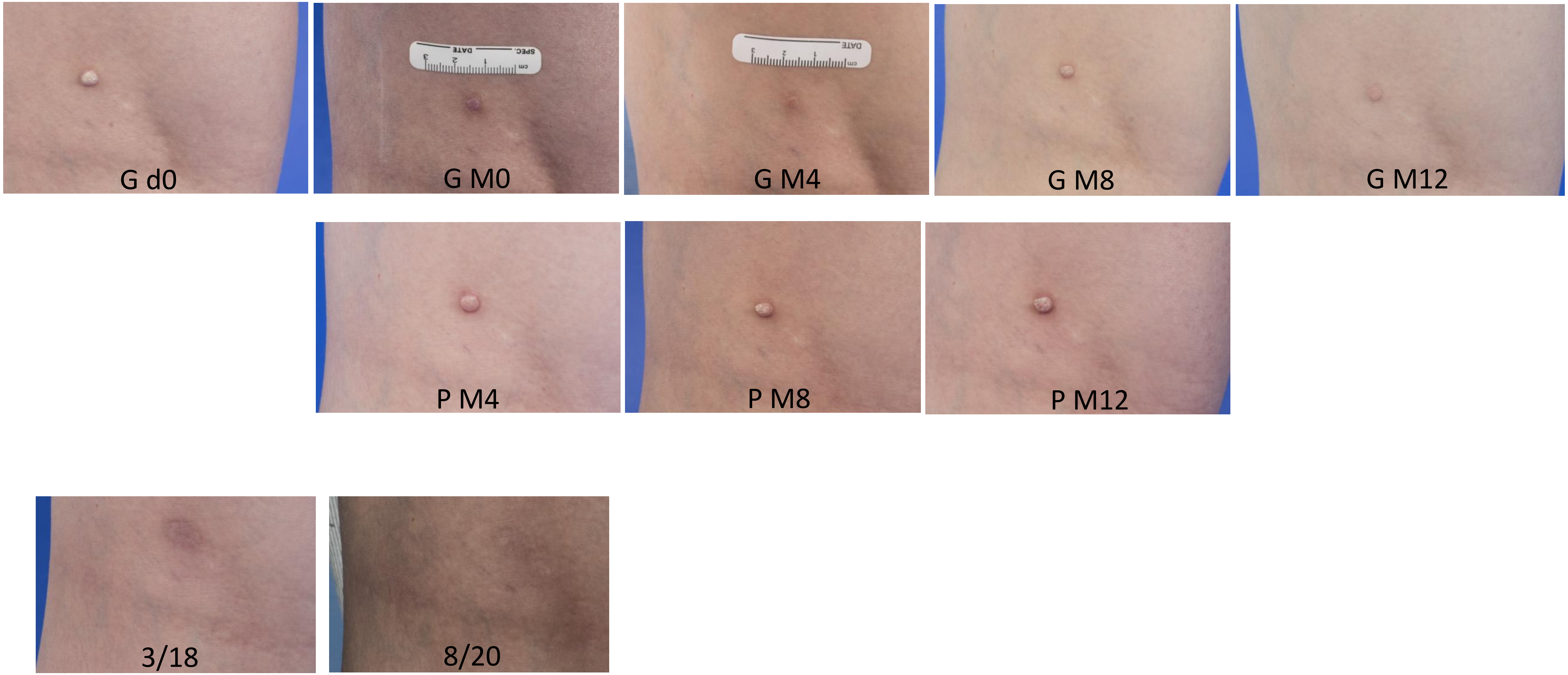

# M02 Left hand, finger 2, DIP region

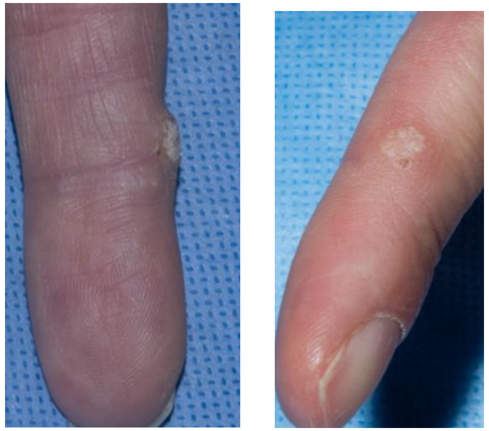

8/2009 8/2010

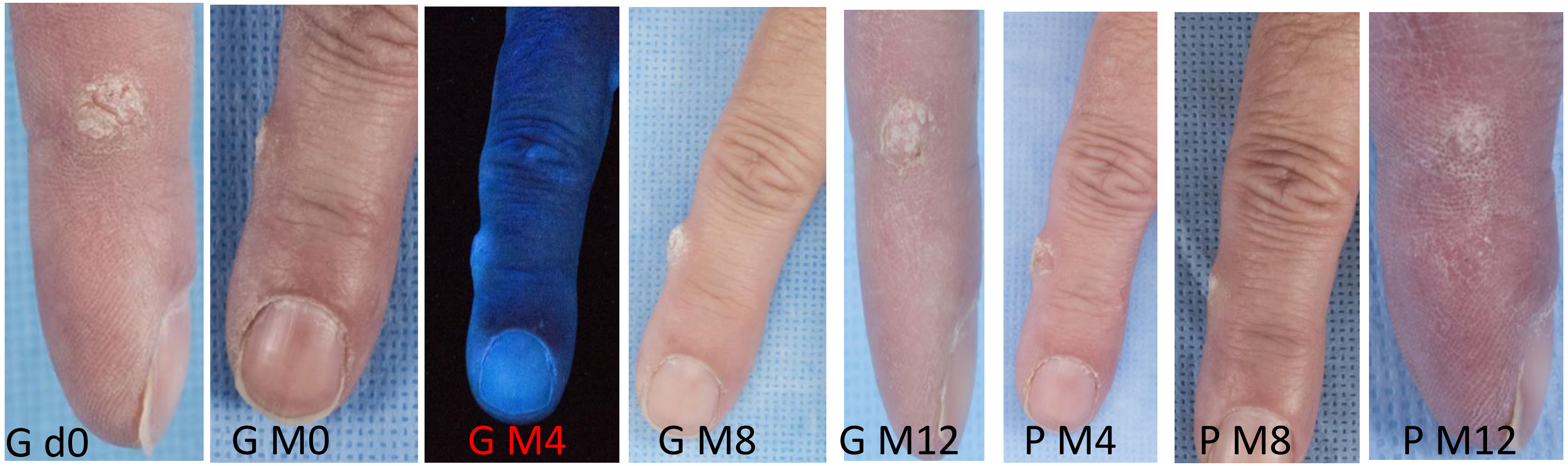


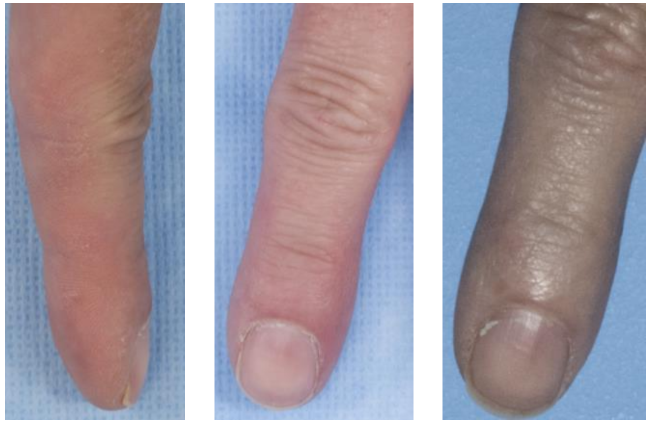

8/2017 3/2018 8/2020

# M02 Left great toe

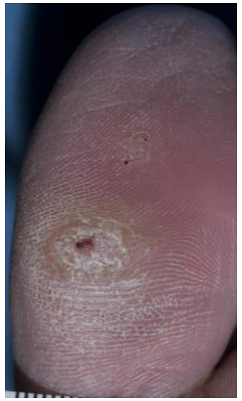
2009

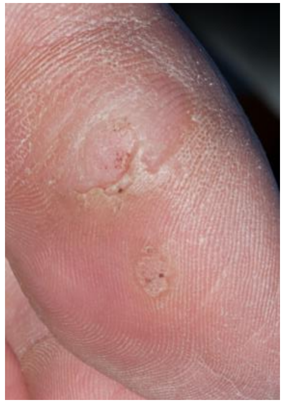
2010

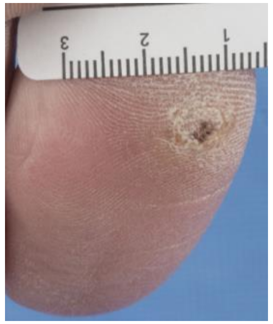
G d0

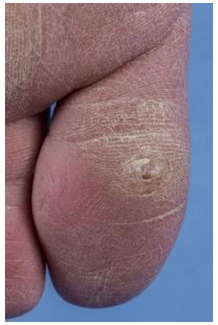
G M0

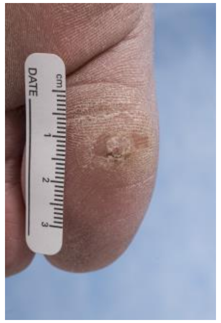
G M4

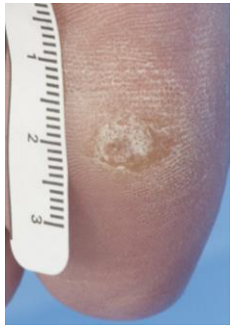
G M8

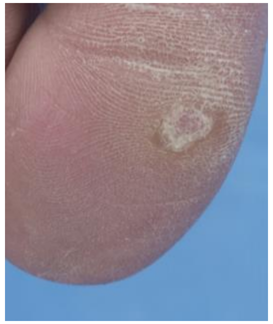
G M12

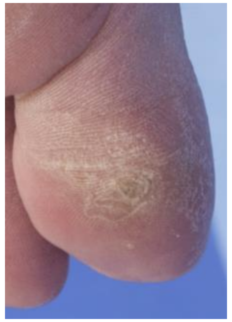
P M4

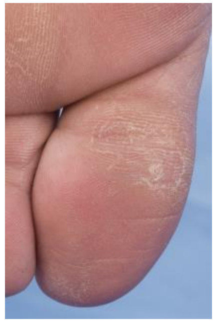
P M8

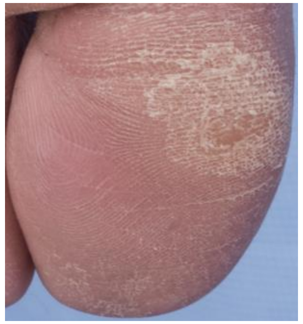
P M12

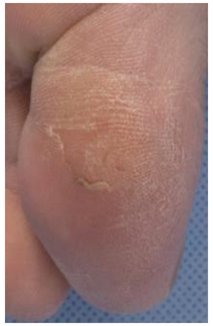
8/2017

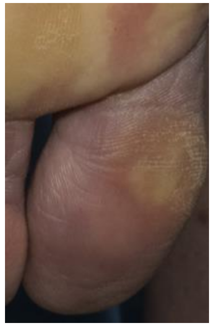
8/2020

# M02 Right great toe

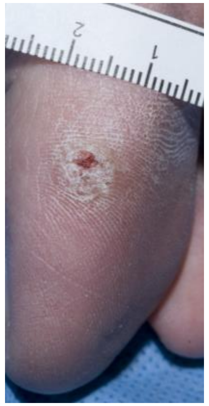
2009

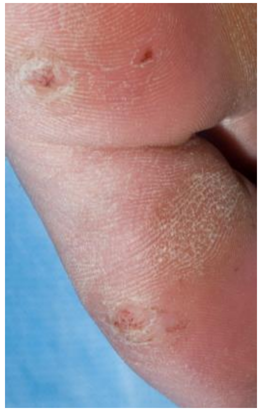
2010

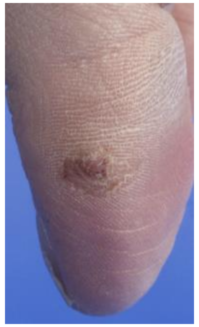
G d0

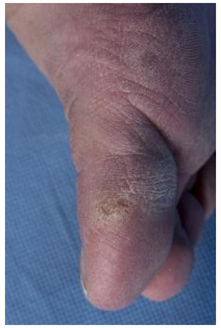
G M0

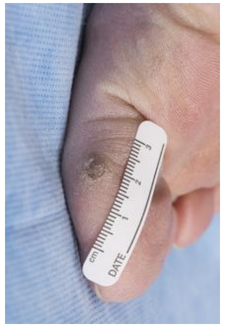
G M4

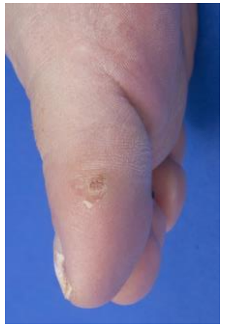
G M8

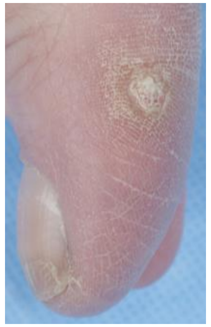
G M12

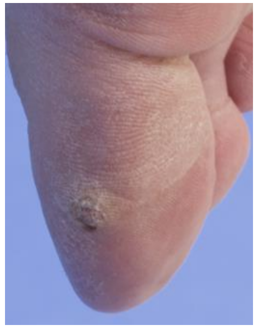
P M4

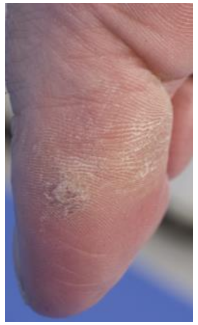
P M8

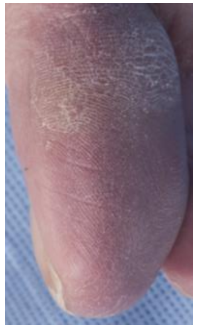
P M12

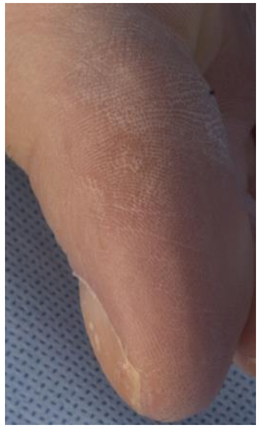
8/2017

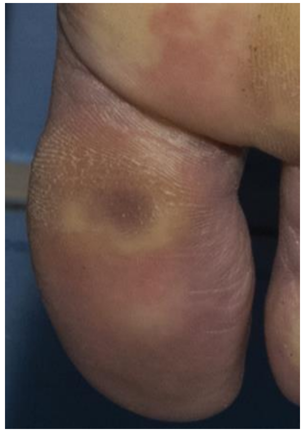
8/2020

# M03 Right knee

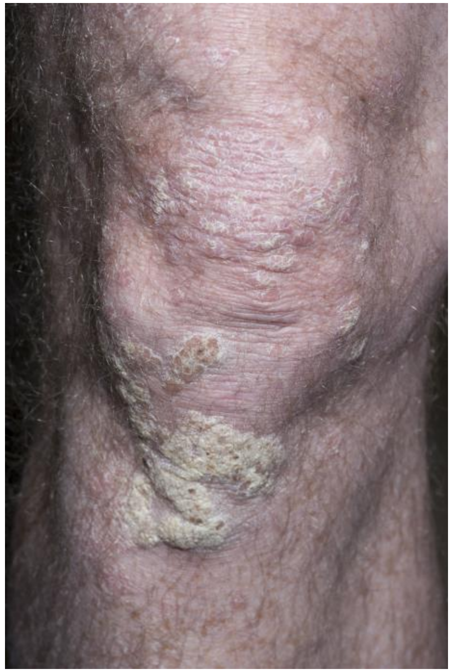

2012

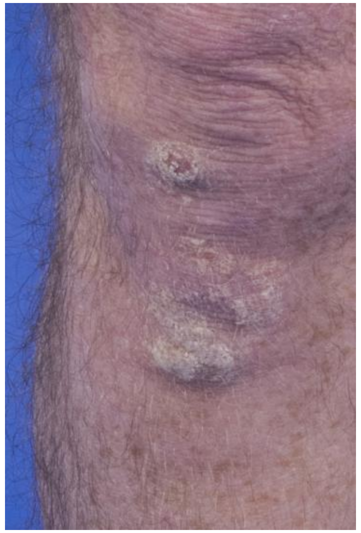

P d0 11/12/14

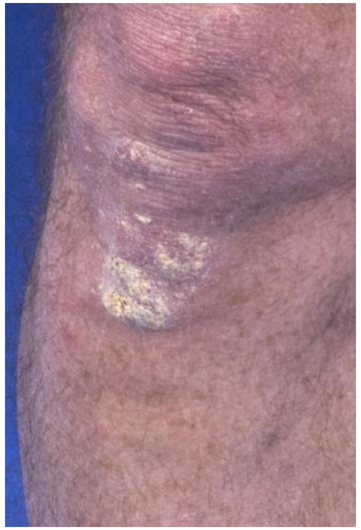

P M0

P M4

P M8

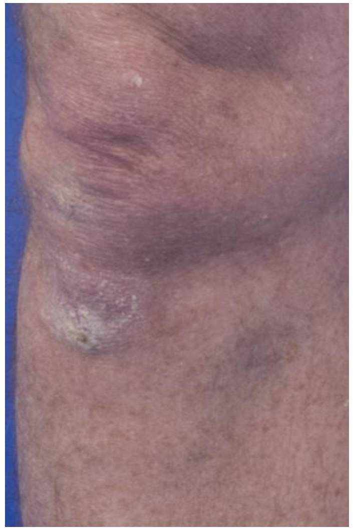

P M12

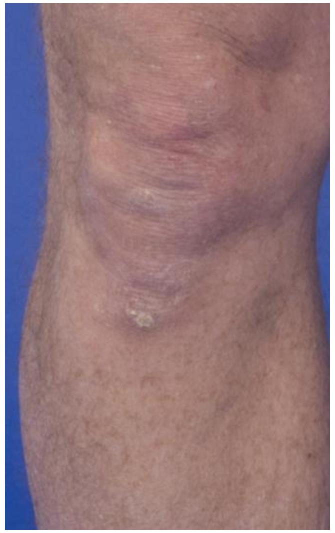

G M0

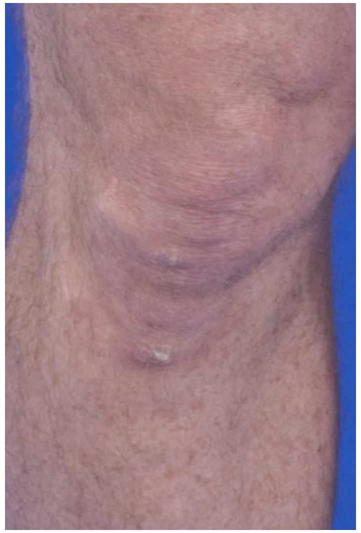

G M4

G M8

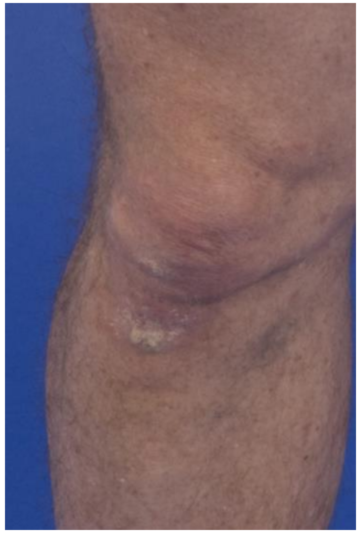

G M12

# M03 Right elbow

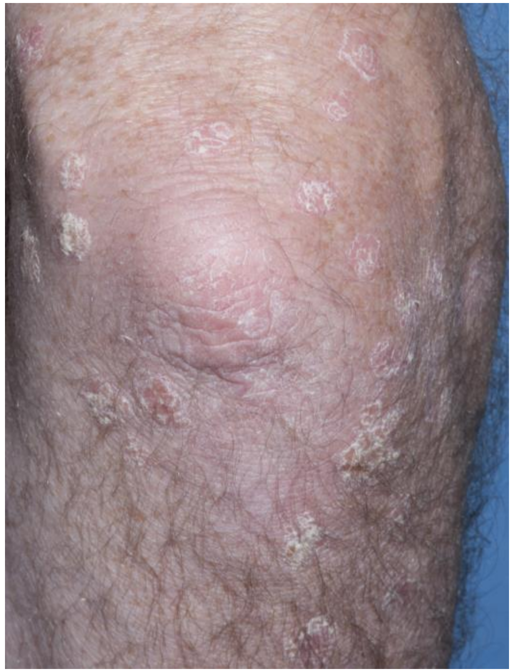
2012

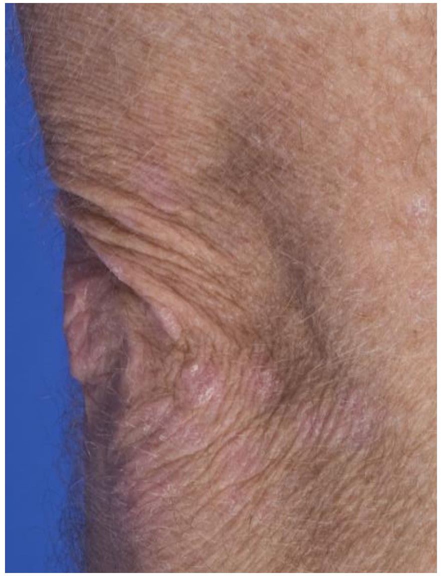
P d0

P M0

P M4

P M8

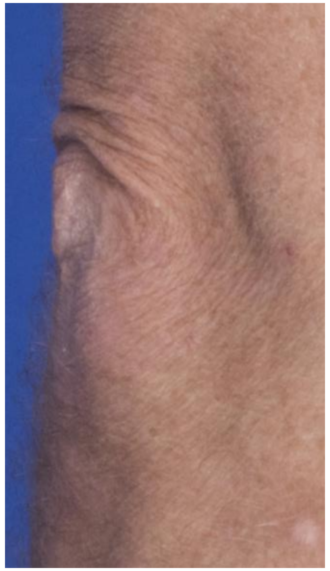
P M12

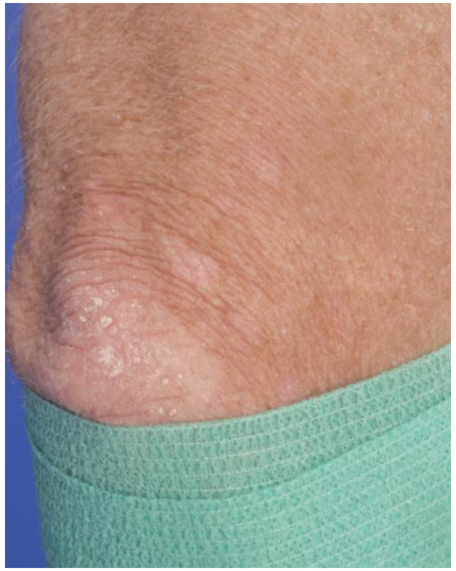
G M0

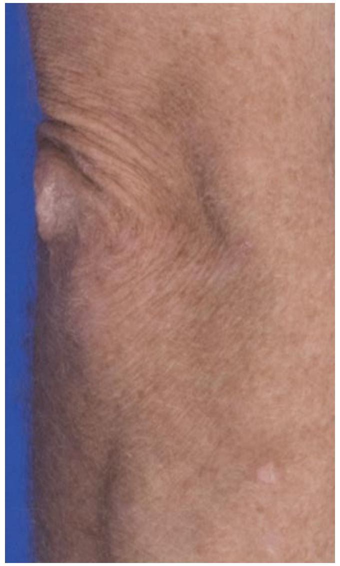
G M4

G M8

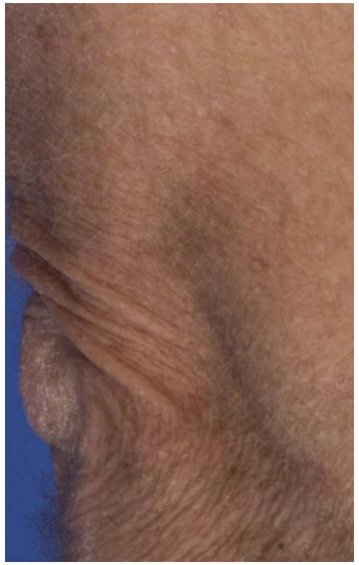
G M12

# M03 Right foot, dorsum

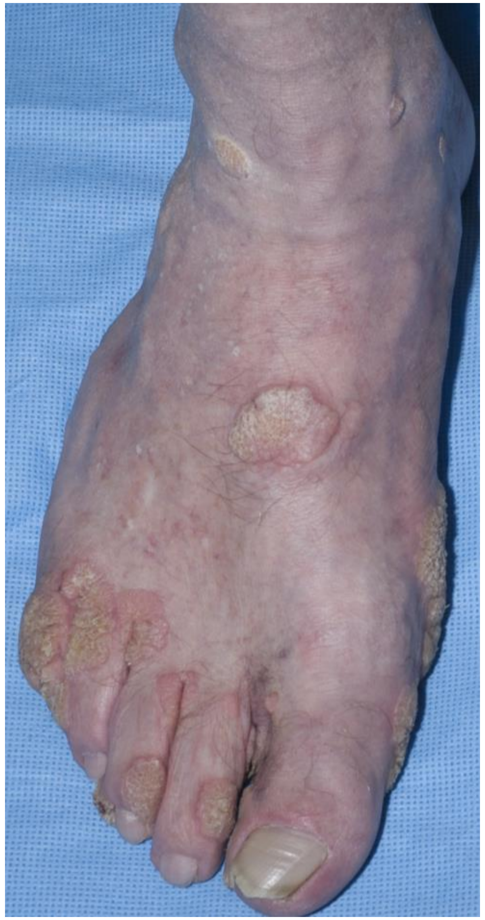

2012

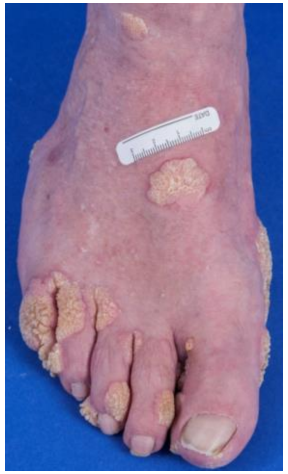

P d0
11/12/14

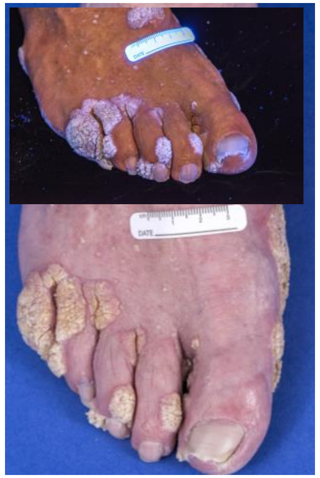

P M0

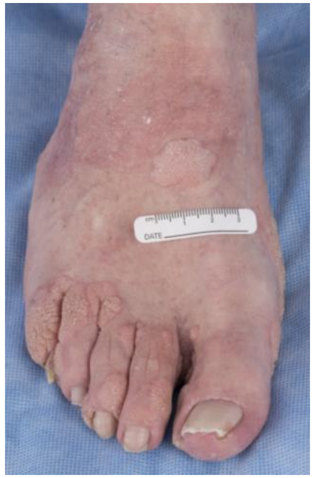

P M4

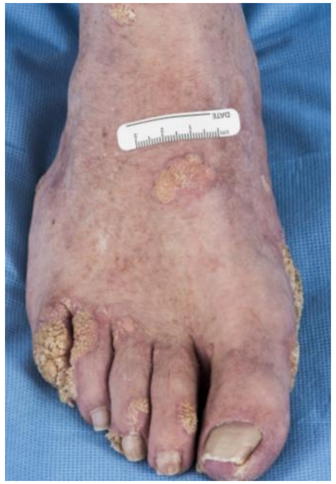

P M8

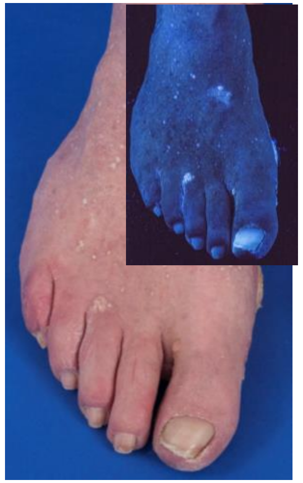

P M12

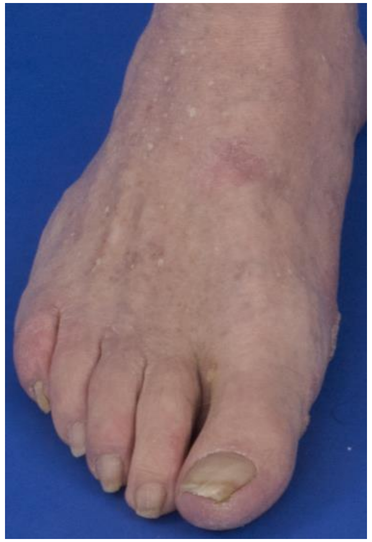

G M0

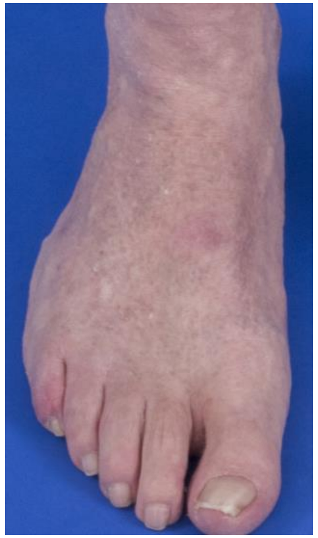

G M4

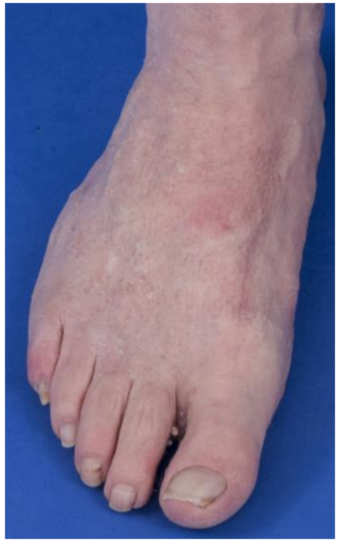

G M8

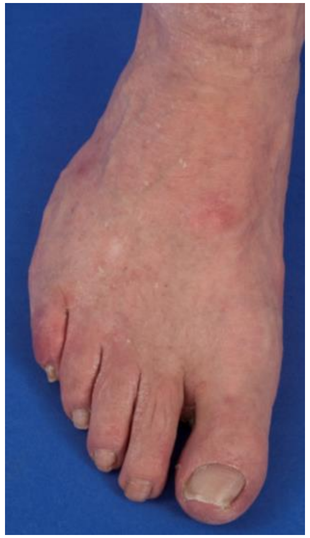

G M12

# M03 Right plantar area

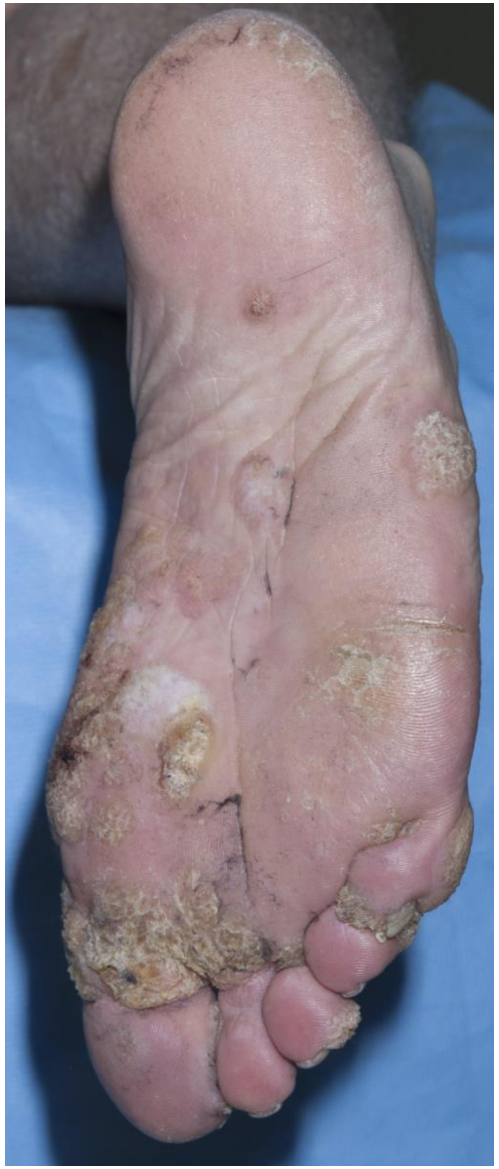

2/2012

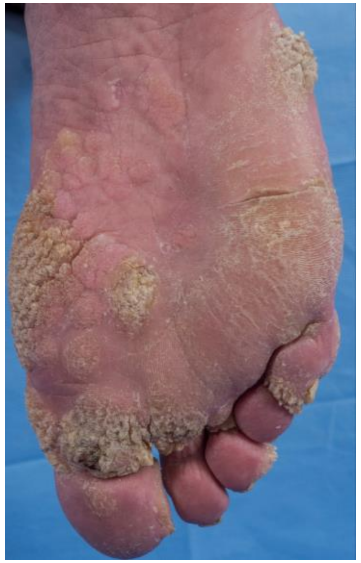

P d0
11/12/14

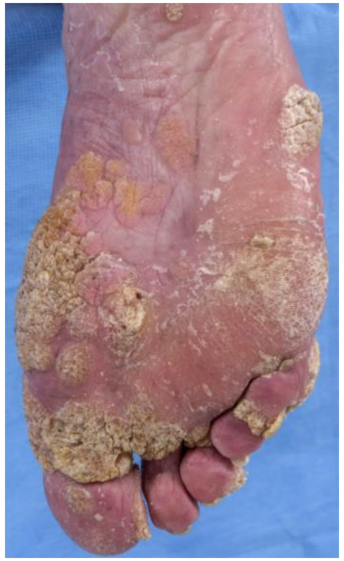

P M0

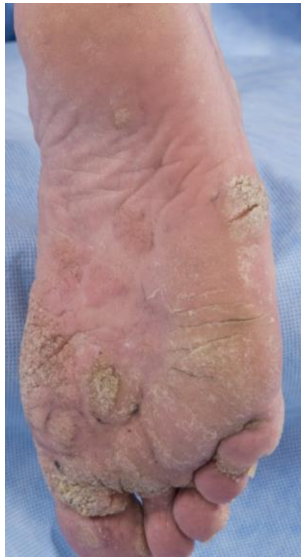

P M4

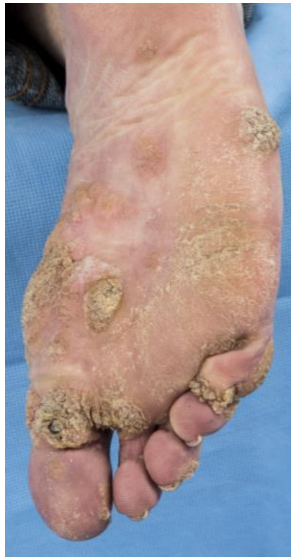

P M8

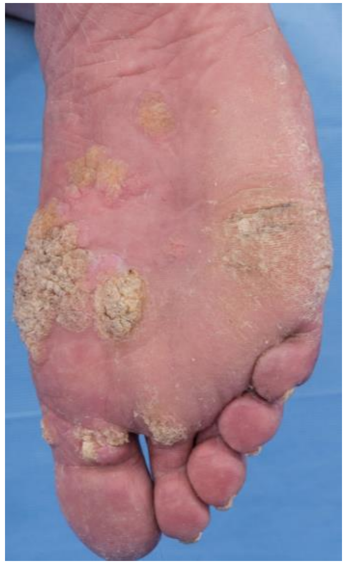

P M12

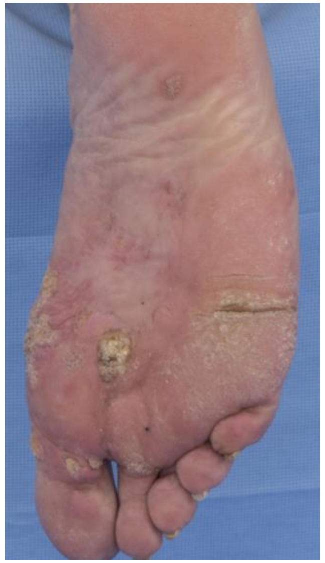

G M0

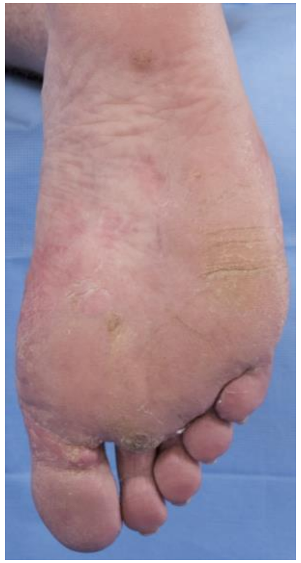

G M4

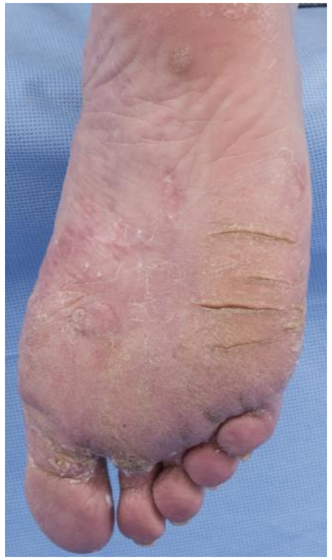

G M8

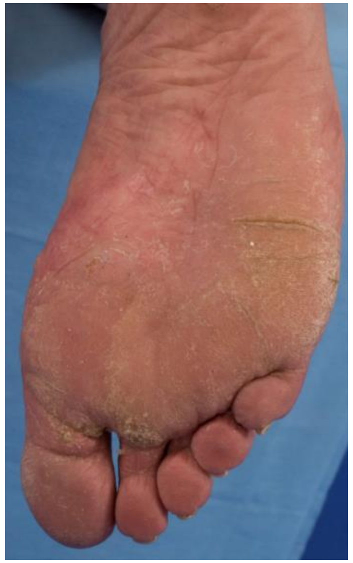

G M12

# M03 Left foot dorsum

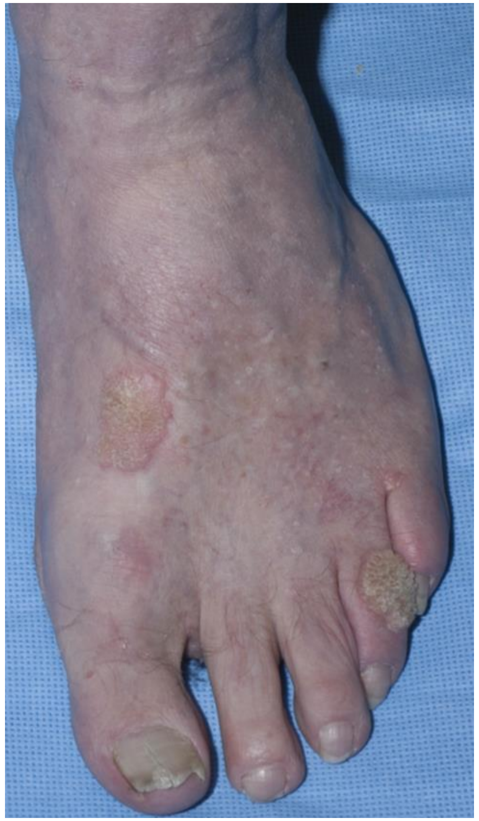

2012

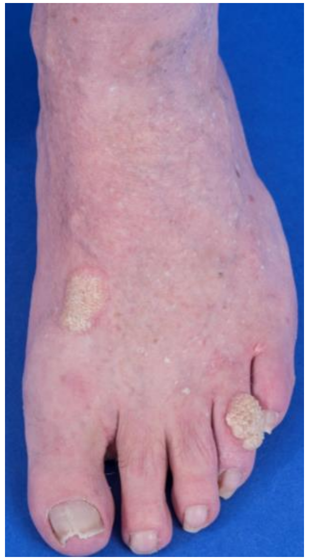

P d0
11/12/14

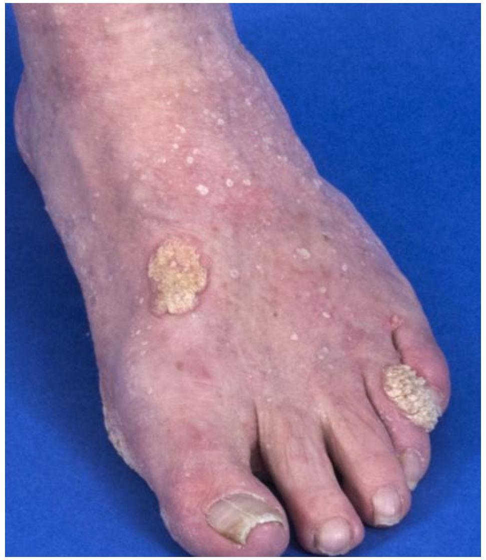

P M0

P M4

P M8

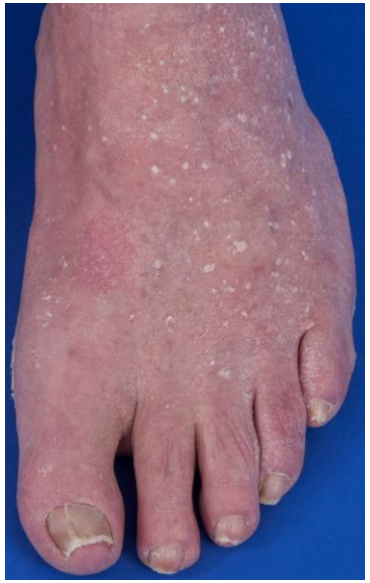

P M12

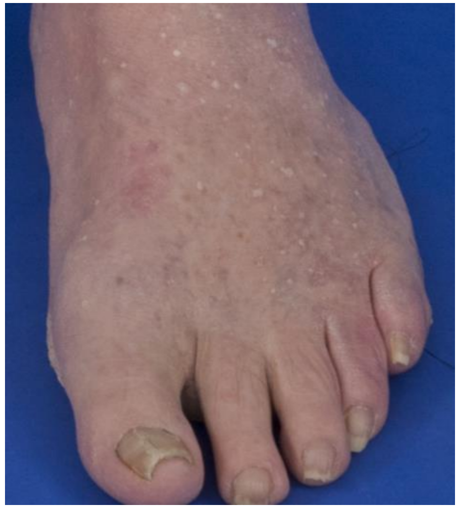

G M0

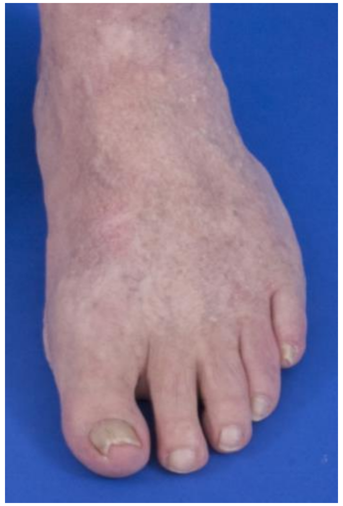

G M4

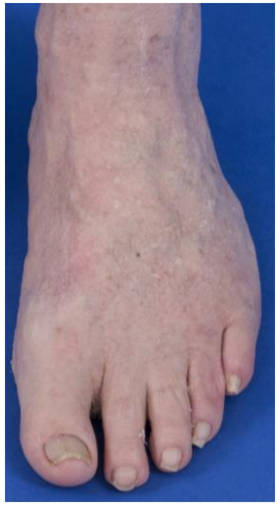

G M8

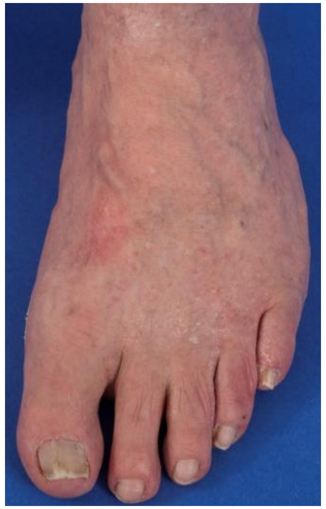

G M12

# M03 Left foot

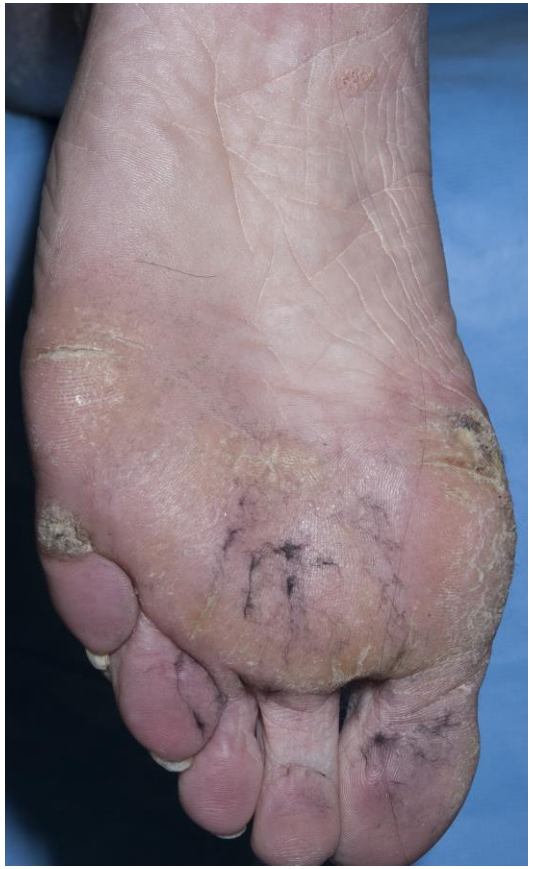

2012

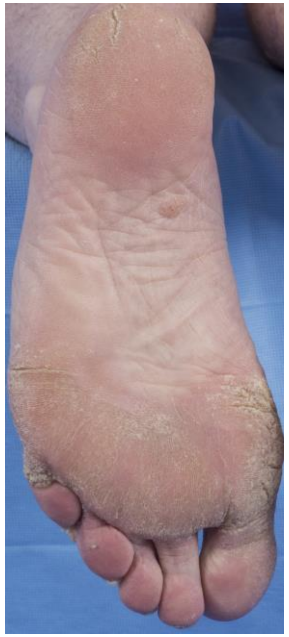

P d0
11/12/14

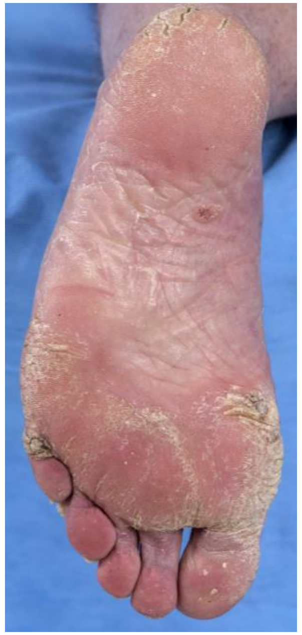

P M0

P M4

P M8

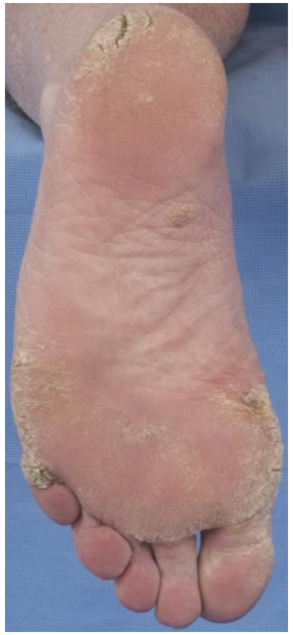

P M12

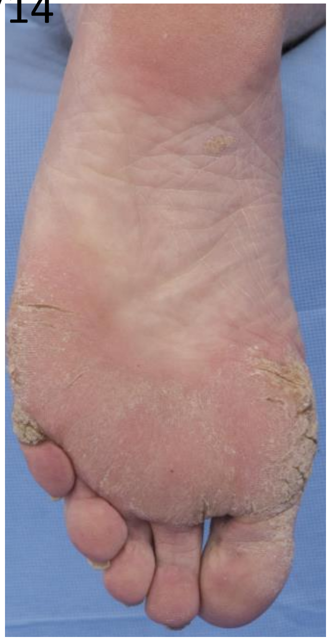

G M0

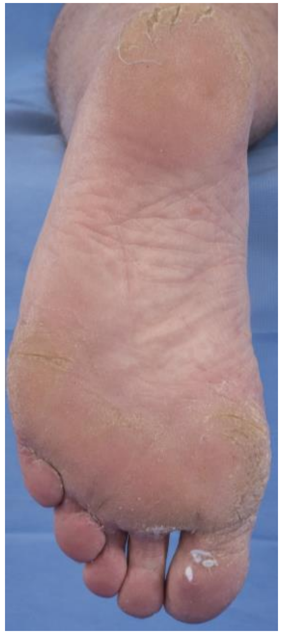

G M4

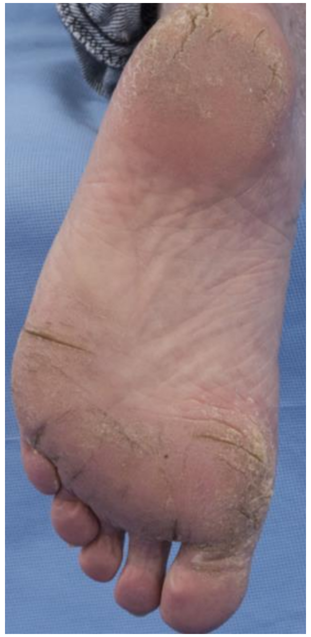

G M8

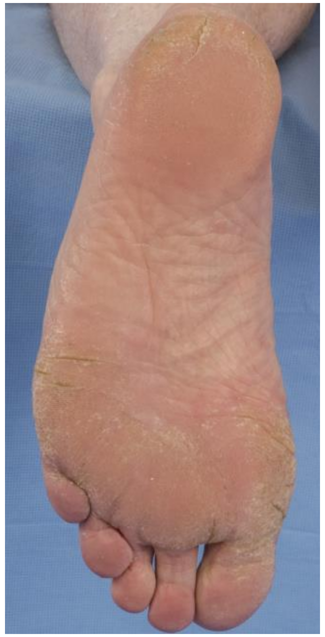

G M12

# M03 Left Achilles tendon

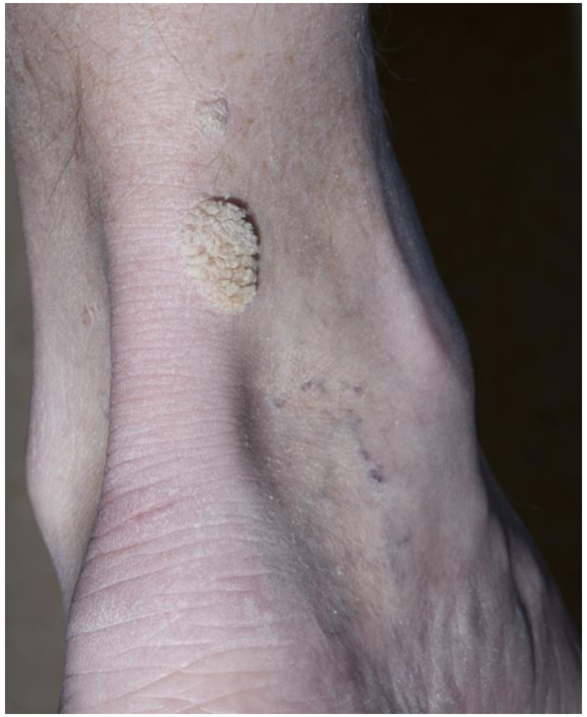

2012

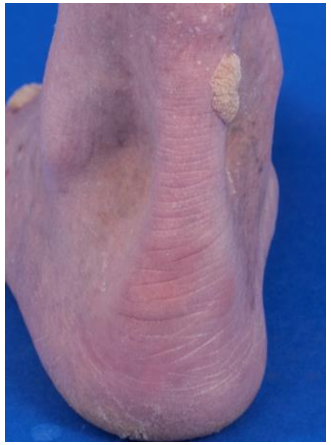

P d0
11/12/14

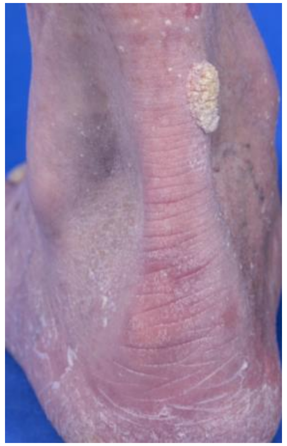

P M0

P M4

P M8

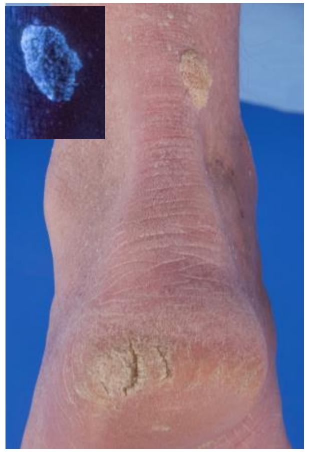

P M12

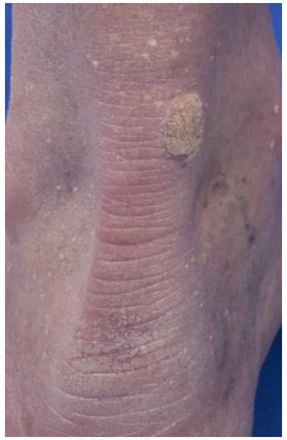

G M0

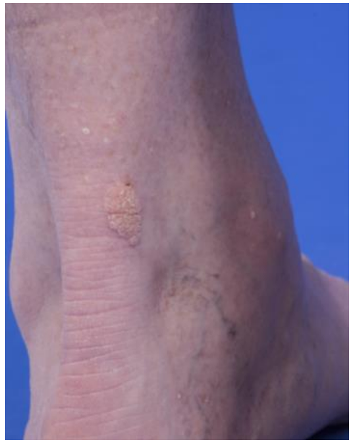

G M4

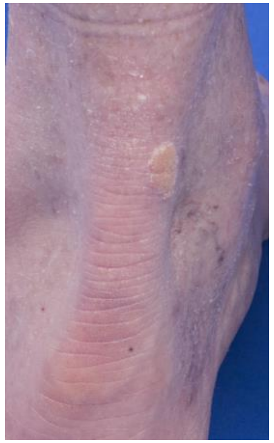

G M8

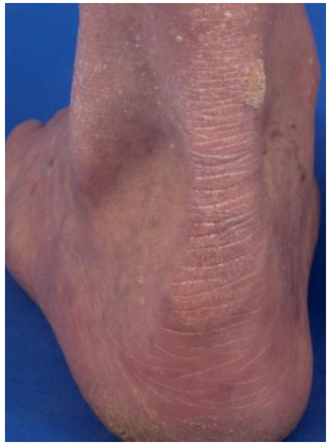

G M12

# M03 Right Hand, Finger 4, PIP and DIP region

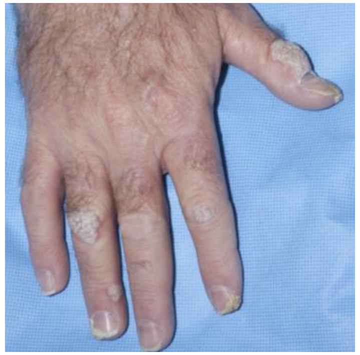

2012

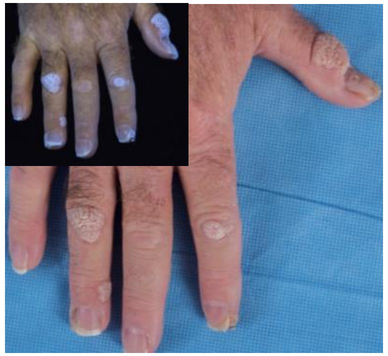

P d0
11/12/14

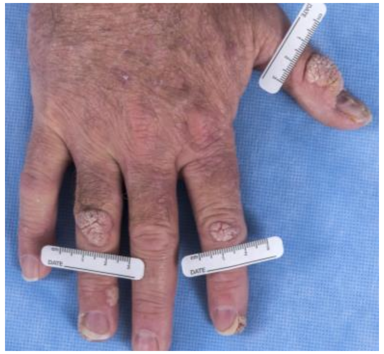

P M0

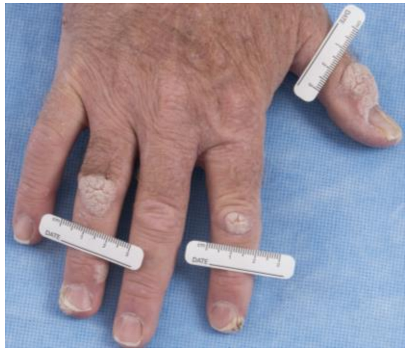

P M4

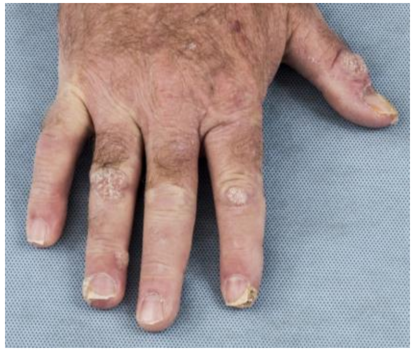

P M8

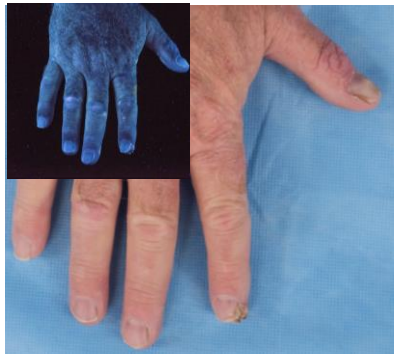

P M12

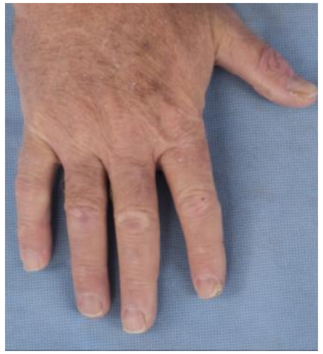

G M0

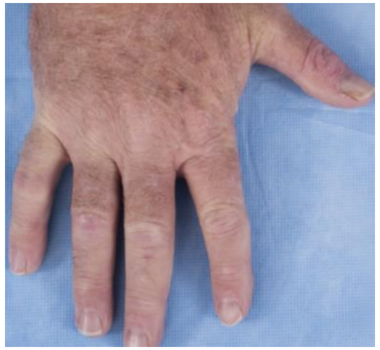

G M4

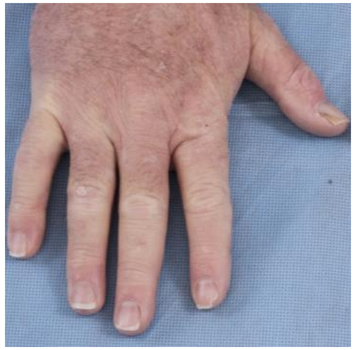

G M8

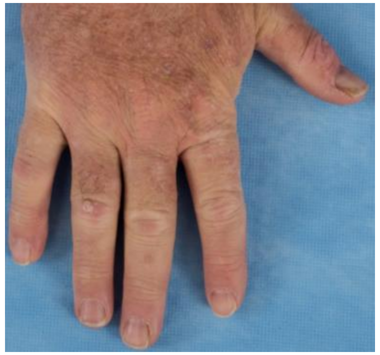

G M12

# M03
## Right Thumb

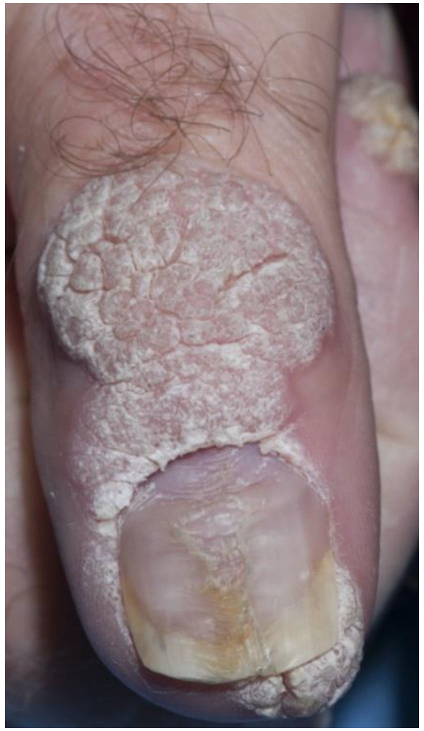

2012

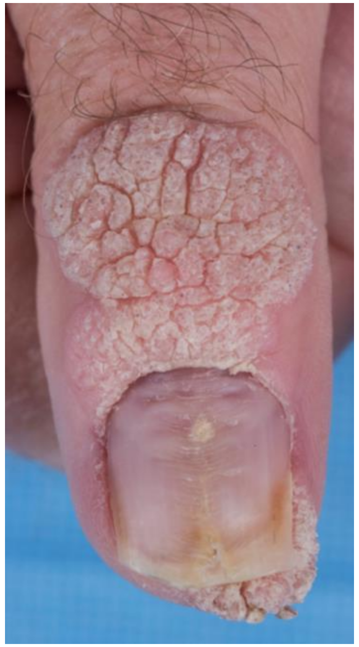

P d0
11/12/14

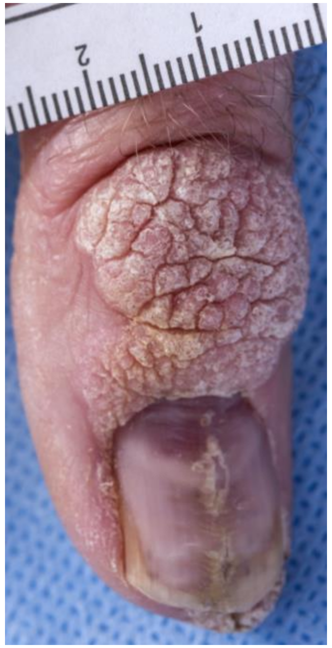

P M0

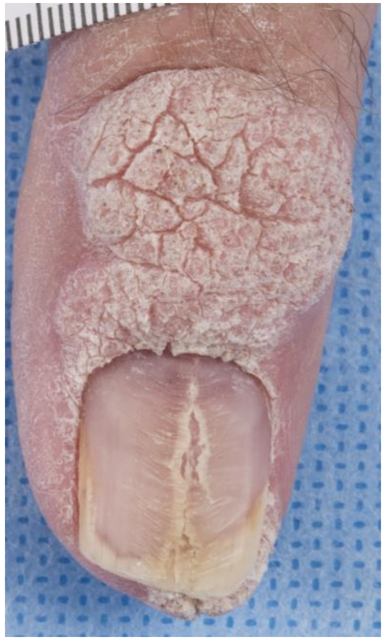

P M4

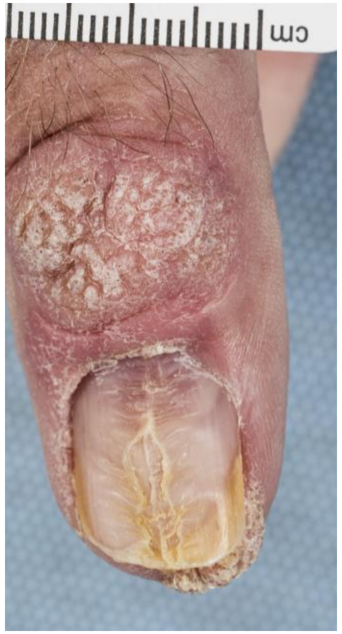

P M8

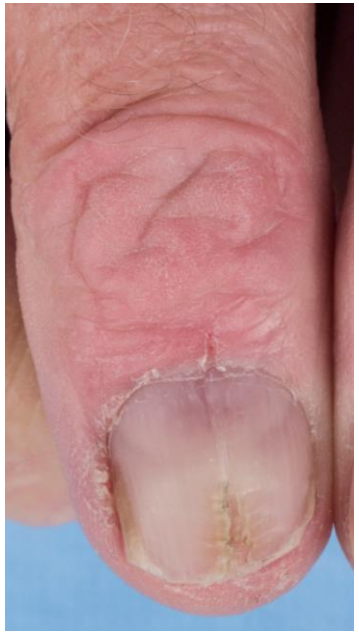

P M12

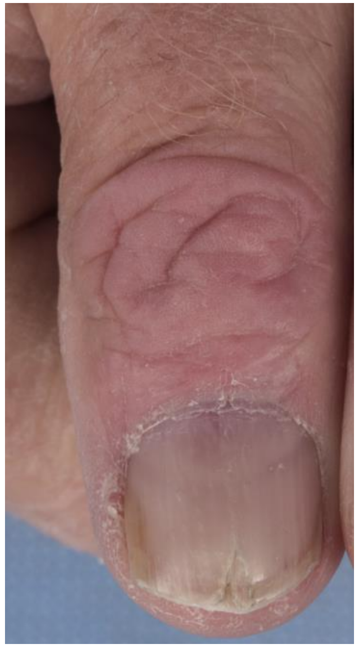

G M0

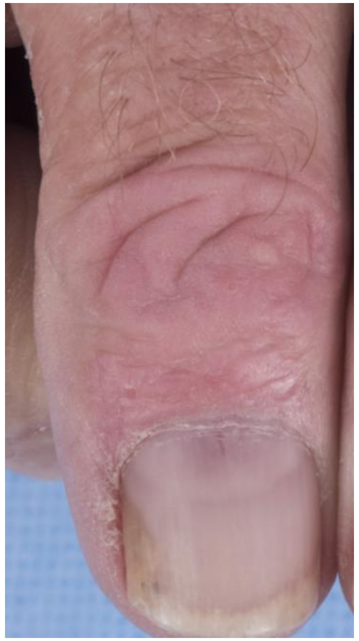

G M4

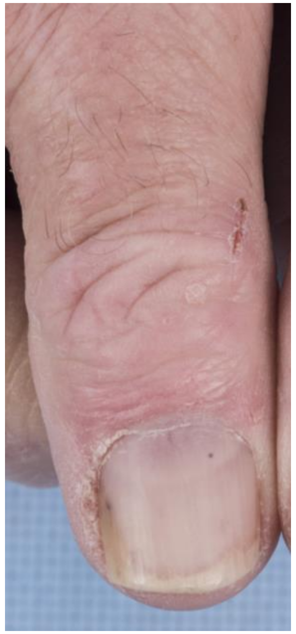

G M8

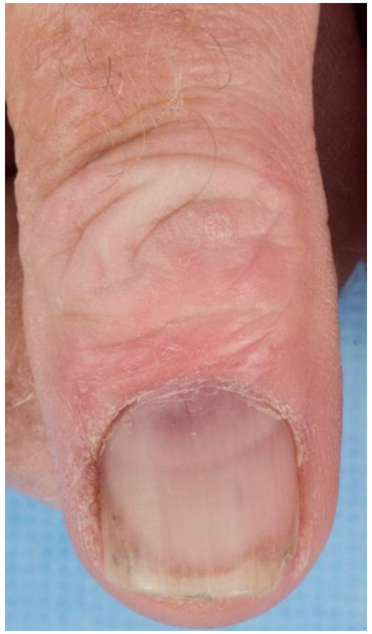

G M12

# M03

Right hand

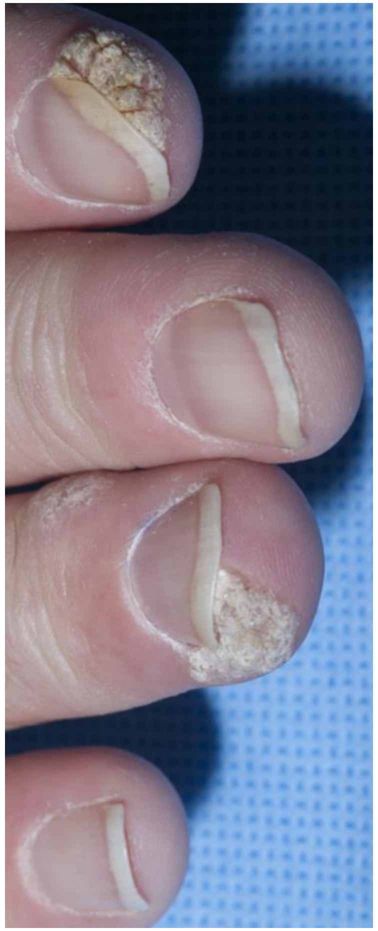

2012

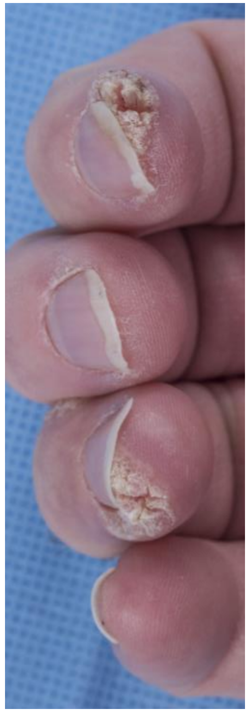

P d0
11/12/14

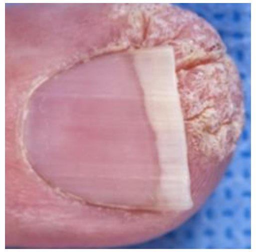

P M0

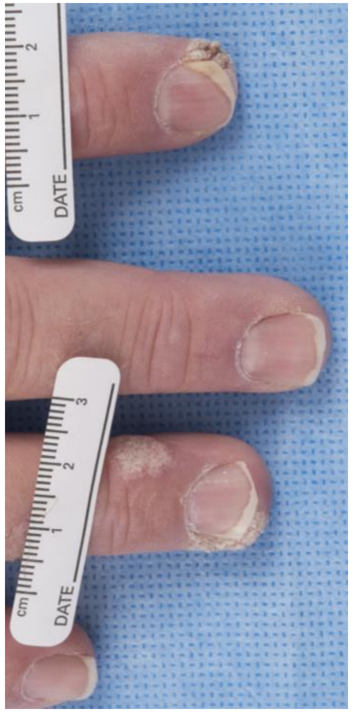


P M4

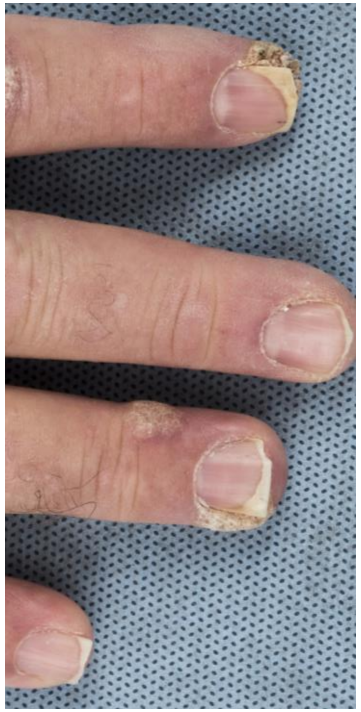

P M8

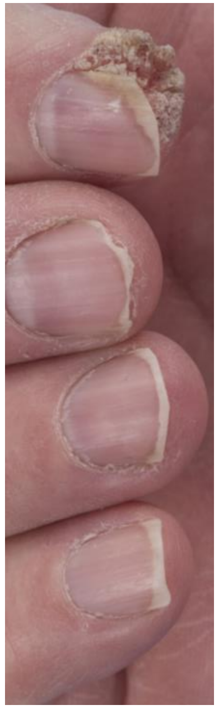

P M12

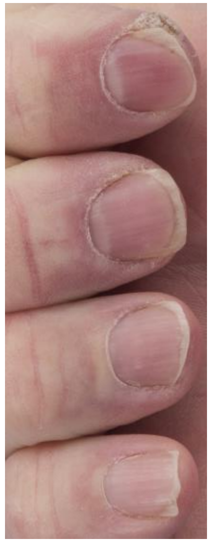

G M0

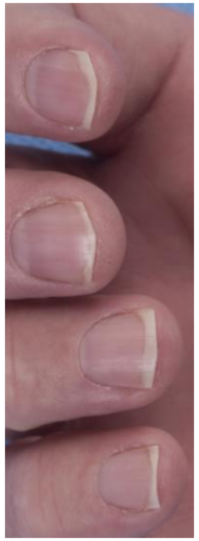

G M4

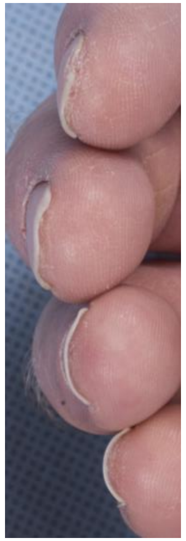

G M8

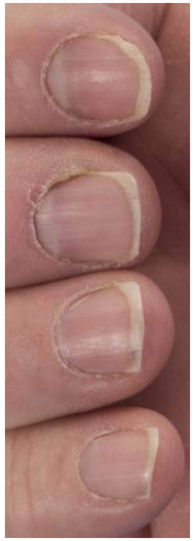

G M12

# M03

Left hand

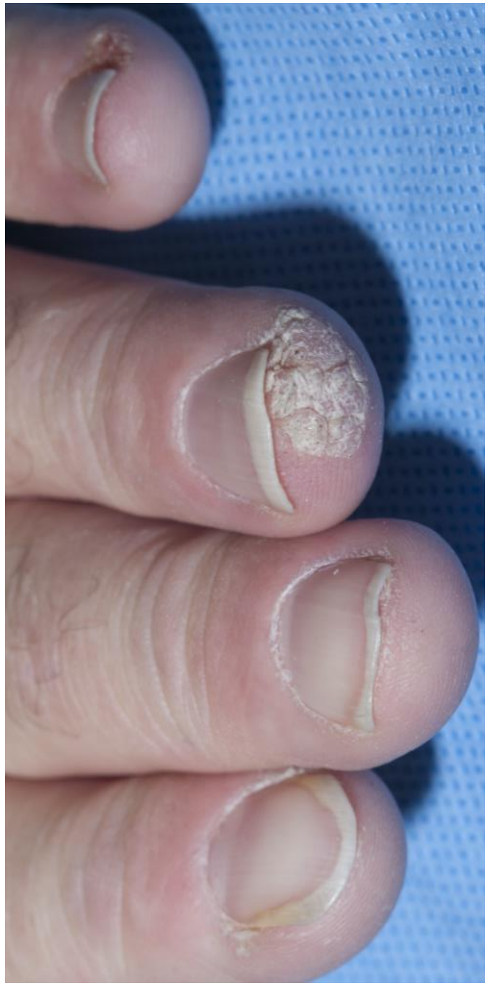

2012

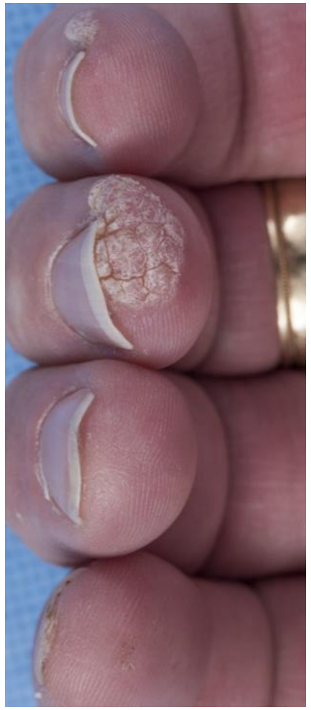

P d0
11/12/14

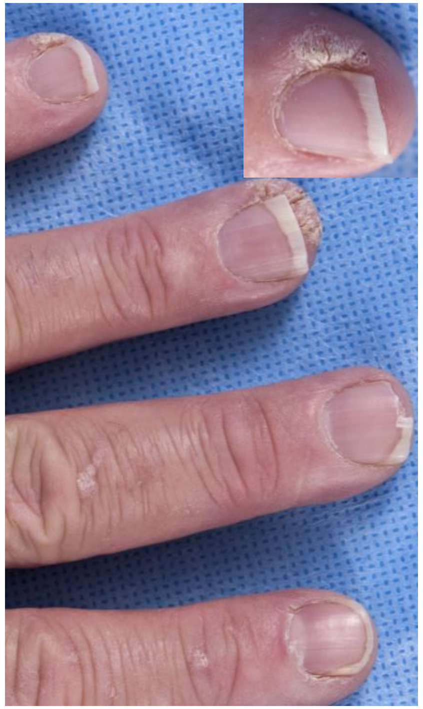

P M0

P M4

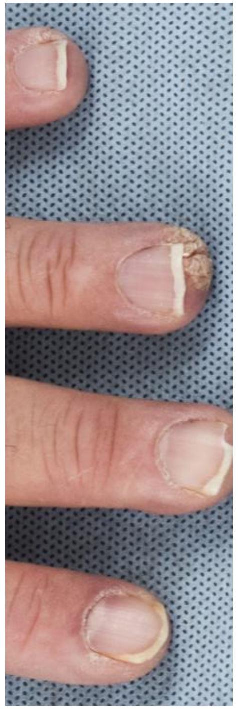

P M8

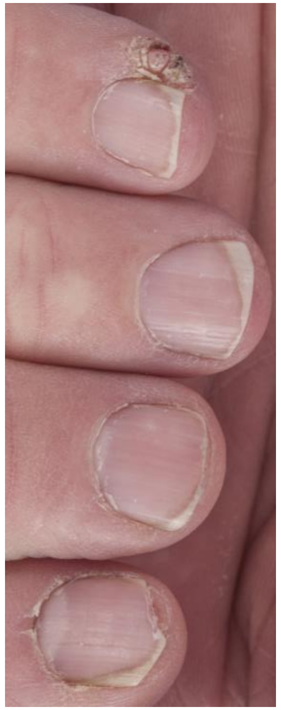

P M12

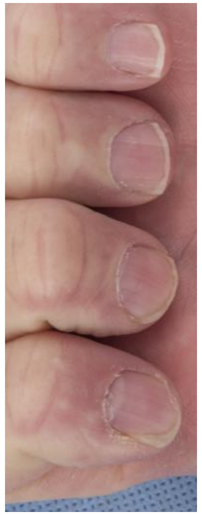

G M0

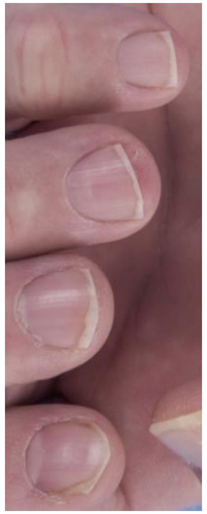

G M4

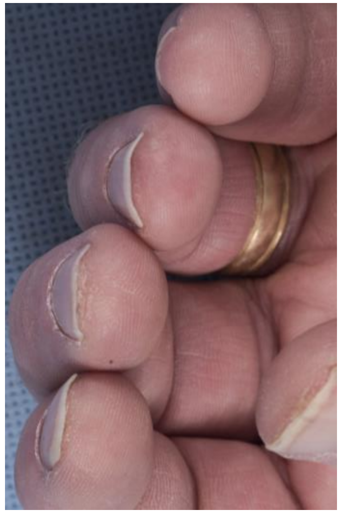

G M8

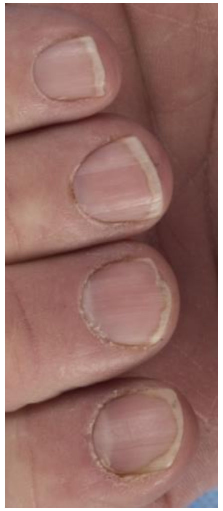

G M12

# M03 Penis, scrotum and left upper thigh

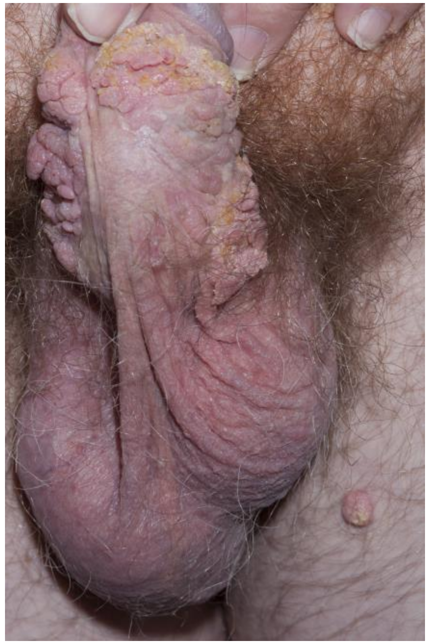

2012

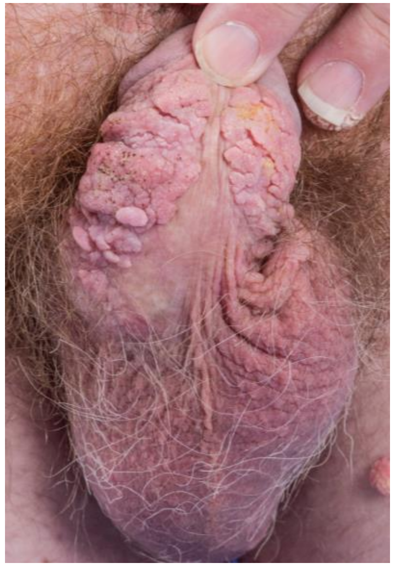

P d0
11/12/14

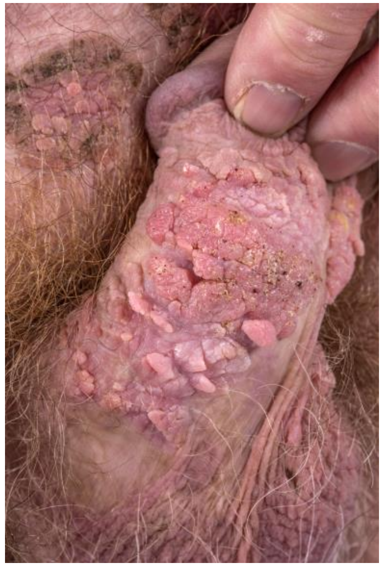

P M0

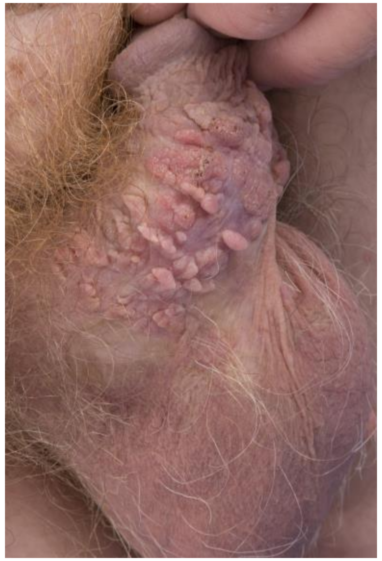

P M4

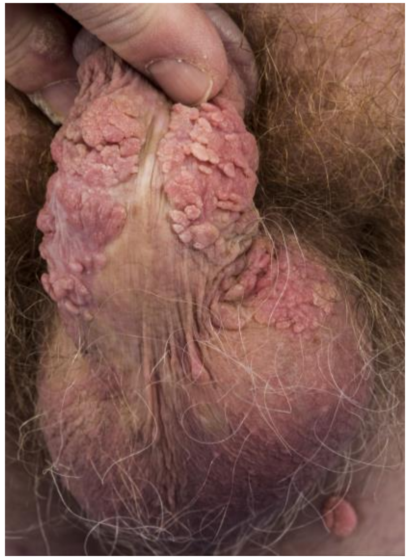

P M8

P M12

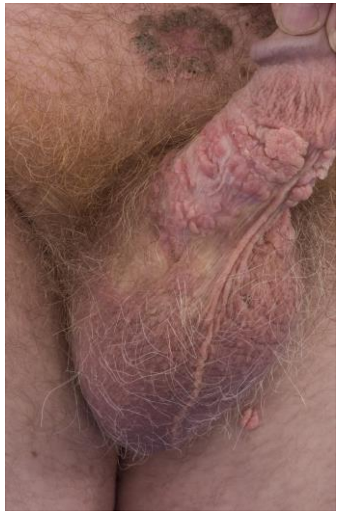

G M0

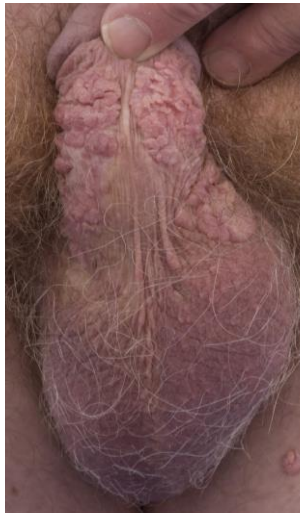

G M4

G M8

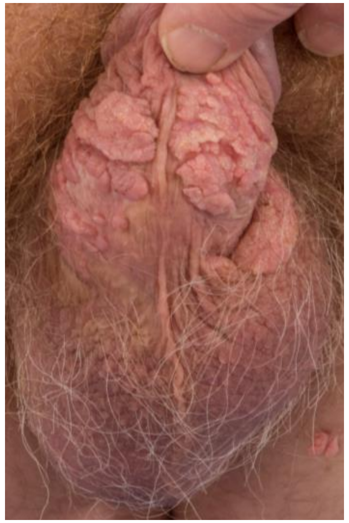

G M12

# M03 Suprapubic area

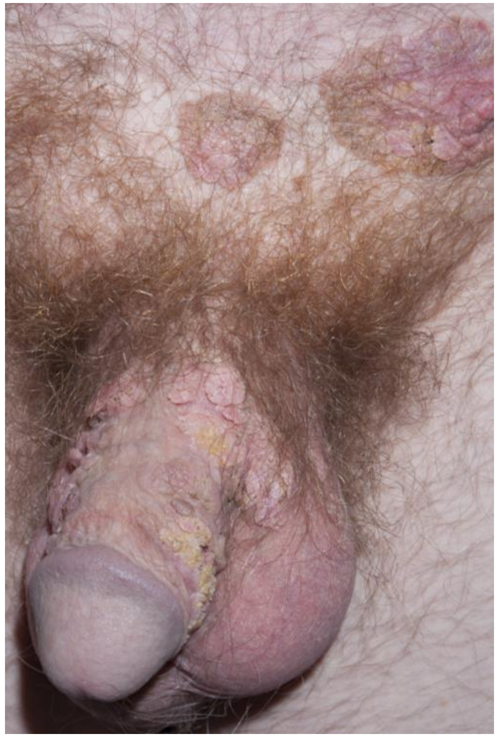

2012

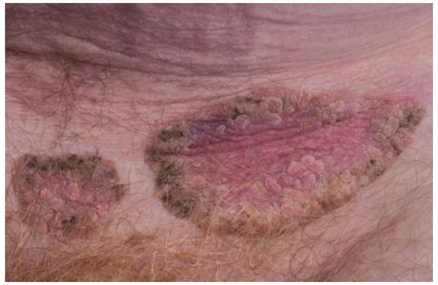

P d0
11/12/14

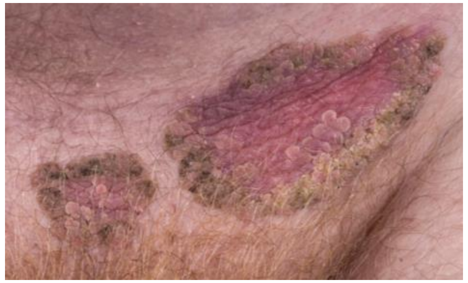

P M0

P M4

P M8

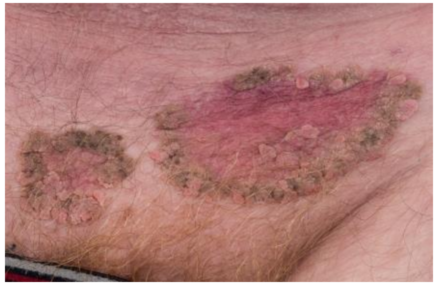

P M12

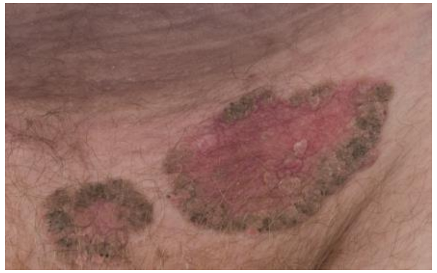

G M0

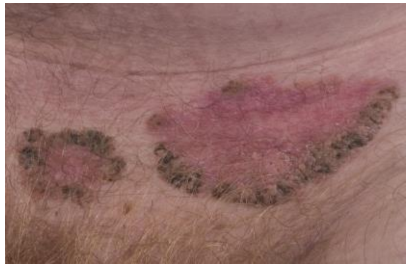

G M4

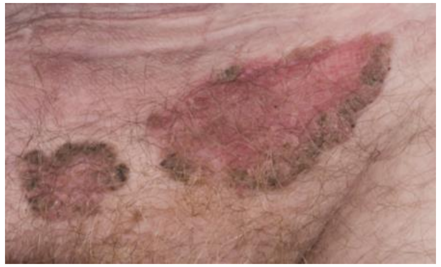

G M8

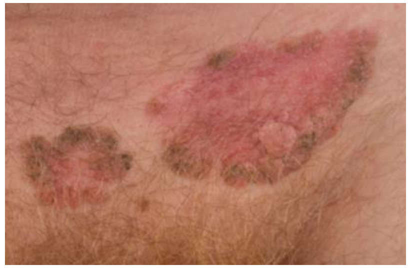

G M12

# M03 Chest area

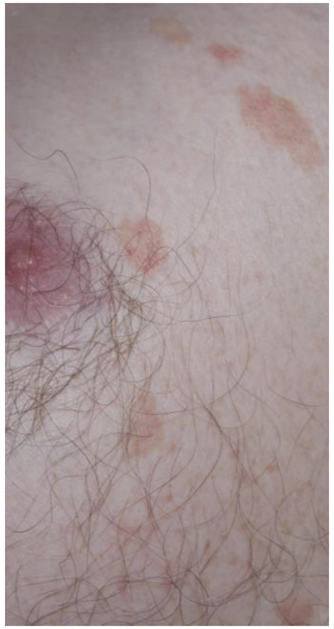
2012

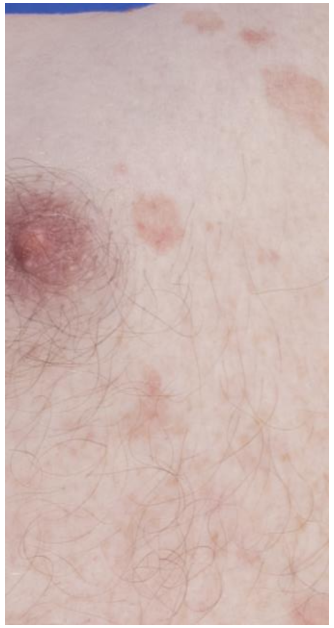
P d0
11/12/14

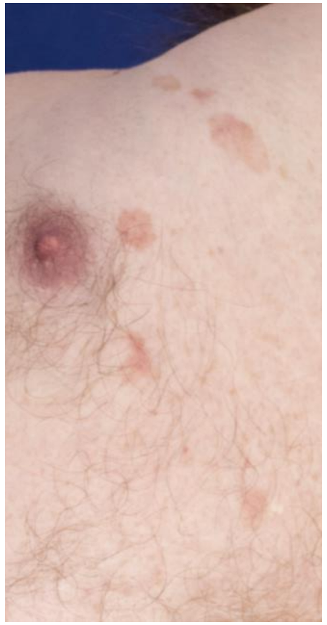
P M0

P M4

P M8

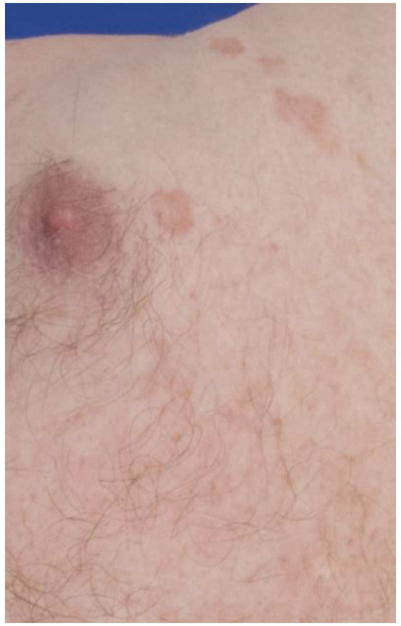
P M12

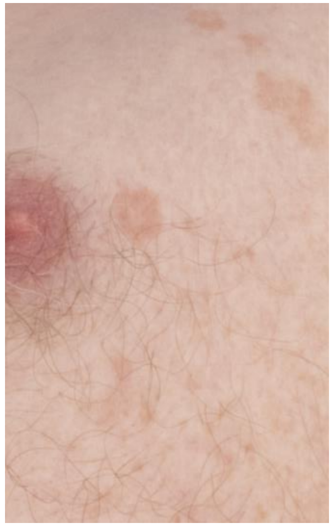
G M0

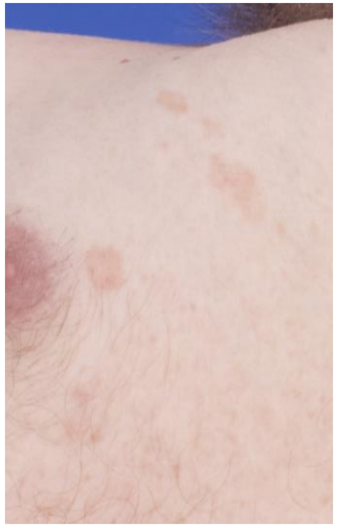
G M4

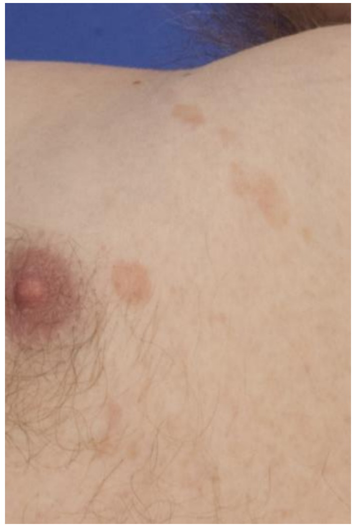
G M12

# M03 Left hand, finger 2

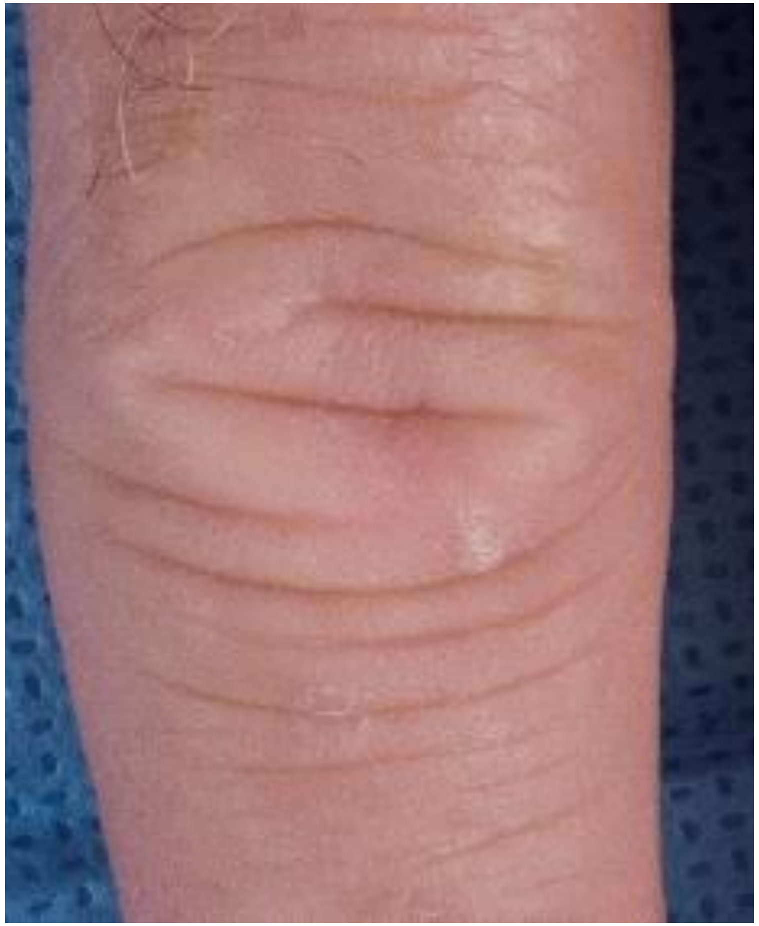

P d0

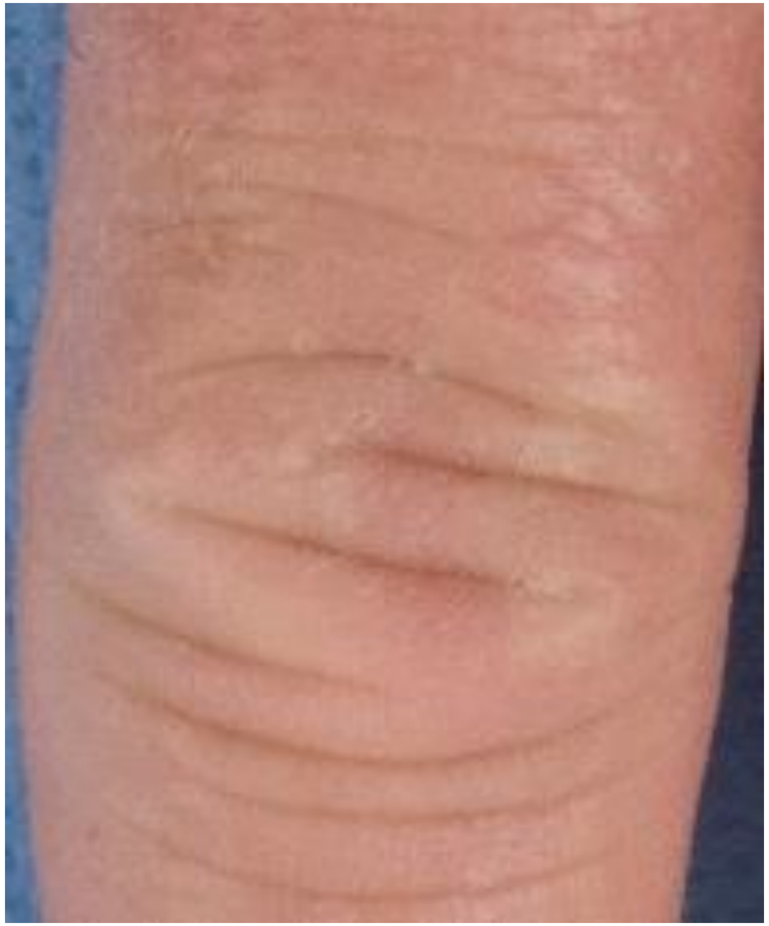

P M12

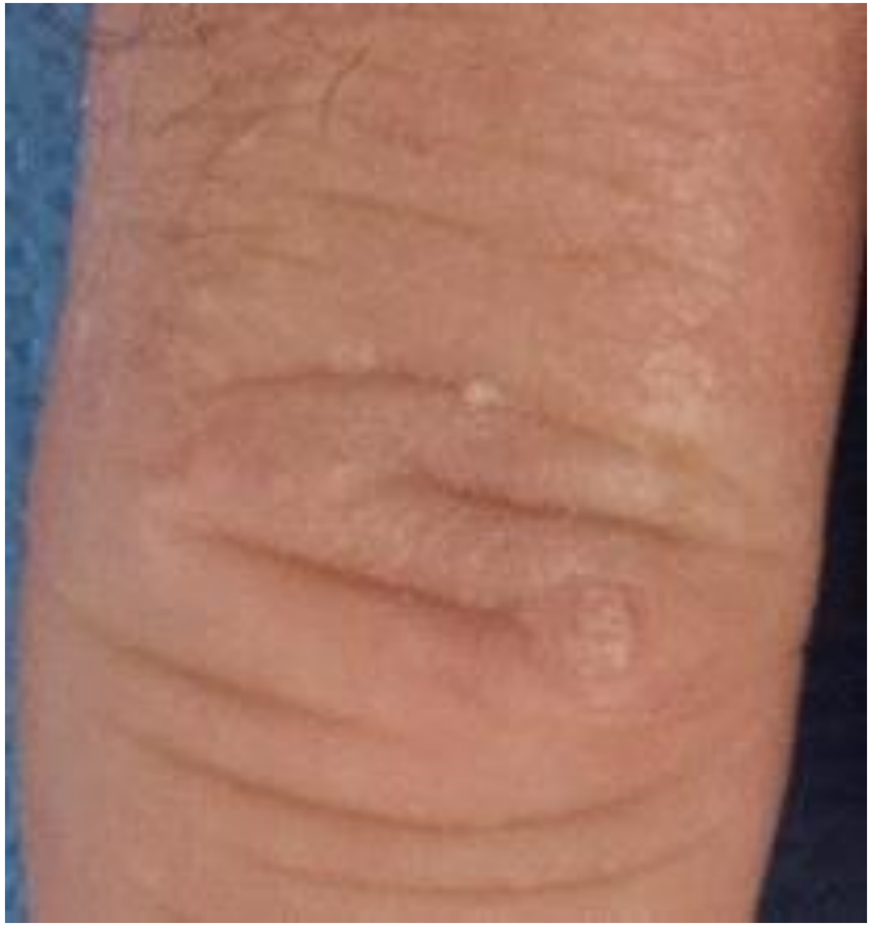

G M12

# M04 Right hand dorsum

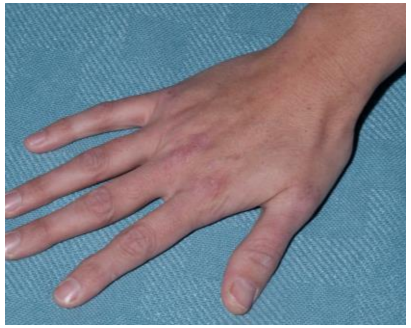

2010

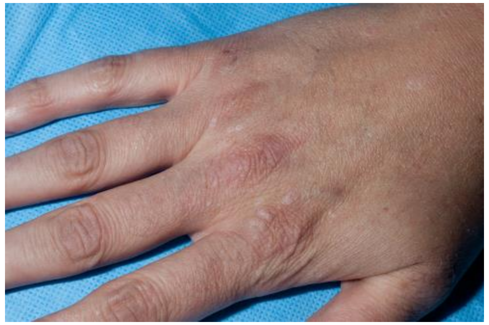

2011

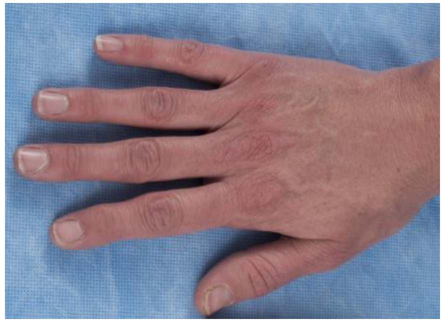

2014

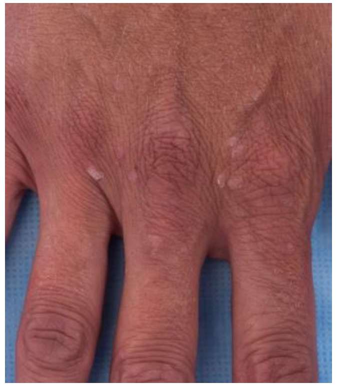

G d0

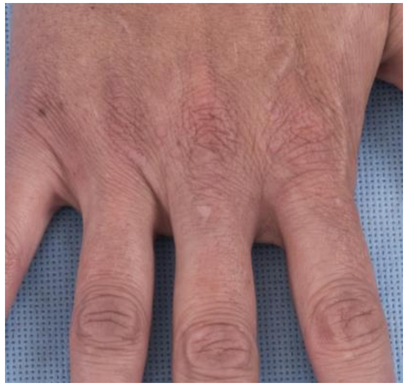

G M0

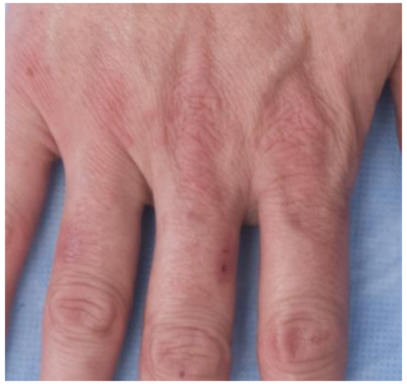

G M4

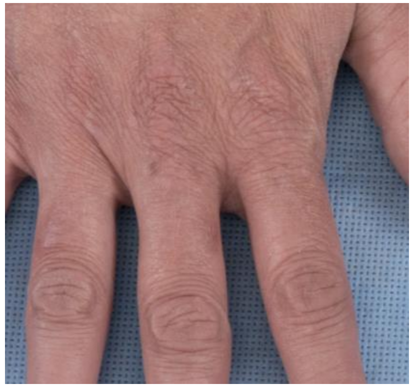

G M8

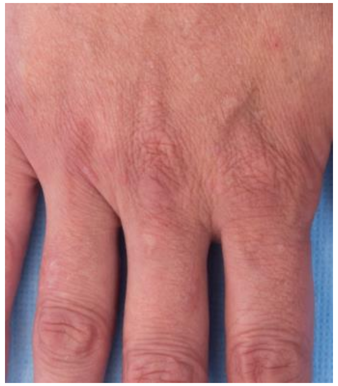

G M12

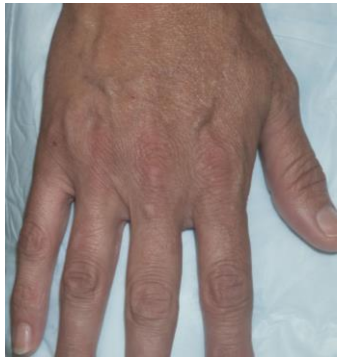

4/19

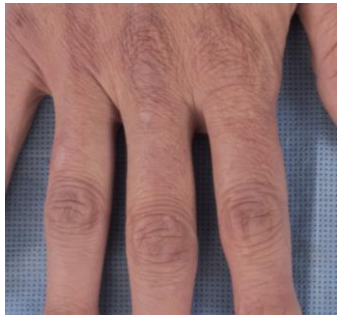

P M0

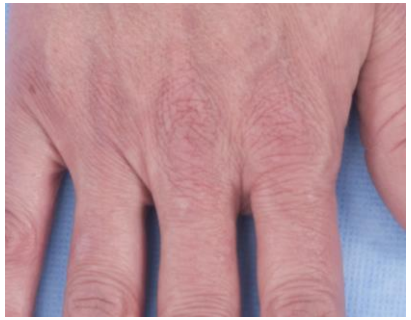

P M4

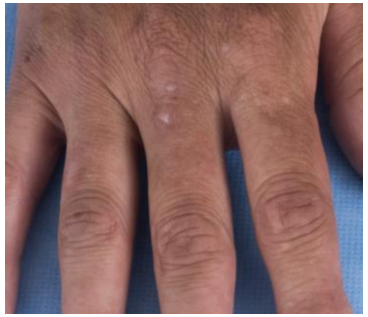

P M8

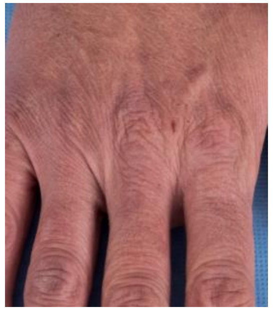

P M12

# M04 Left hand, finger 3 dorsum

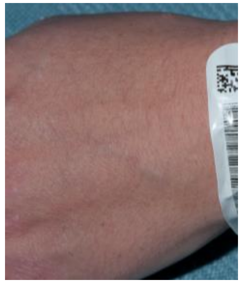
2010

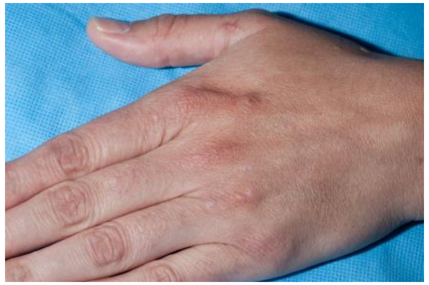
2011

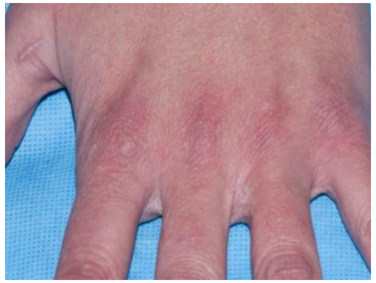
2012

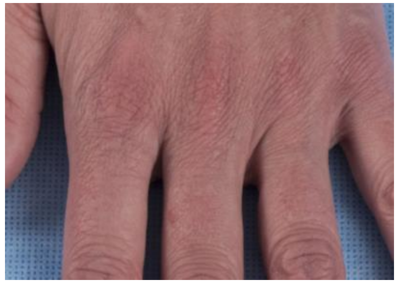
2014

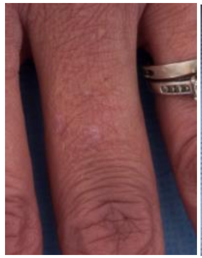
G d0

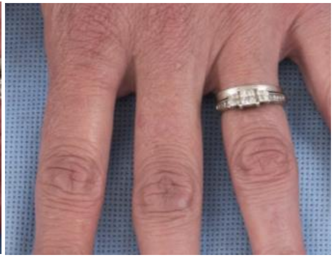
G M0

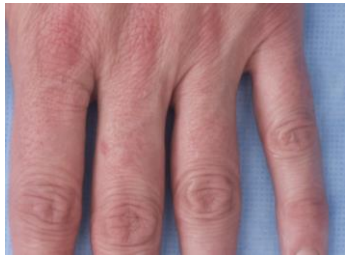
G M4

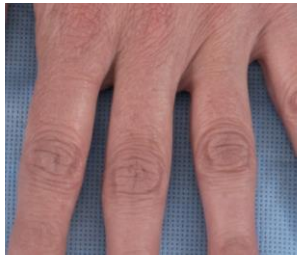
G M8

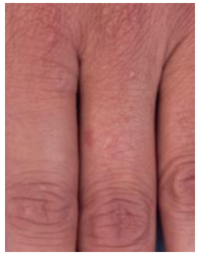
G M12

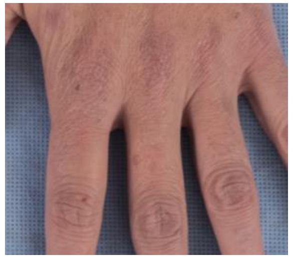
P M0

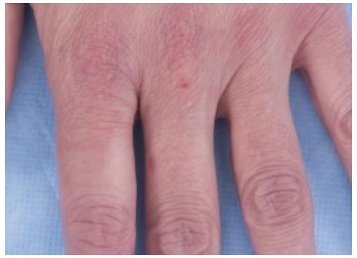
P M4

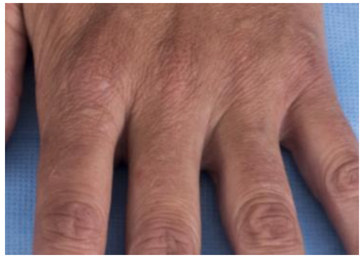
P M8

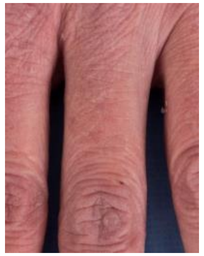
P M12

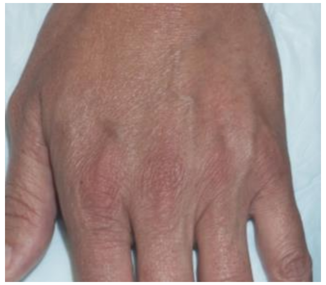
4/19

# M04 Genitalia

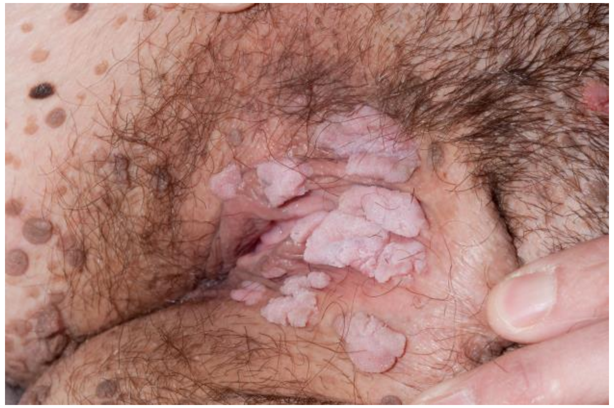

2010

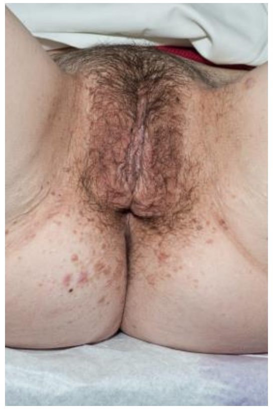

2011

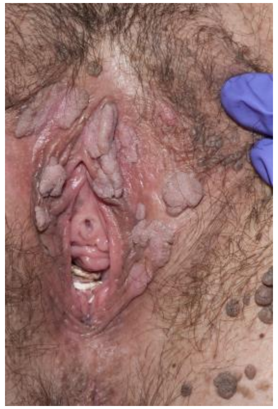

2012

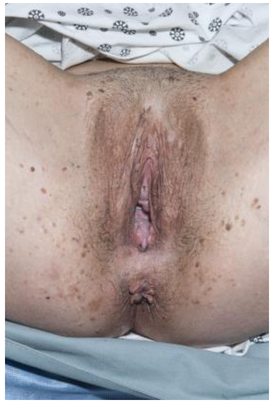

2013
Post surgery

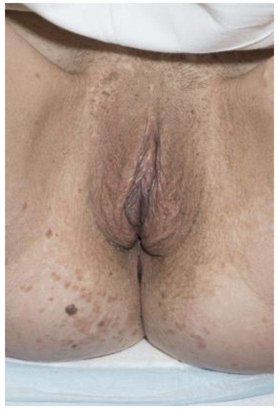

4/19

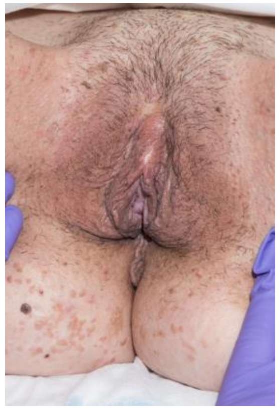

G d0

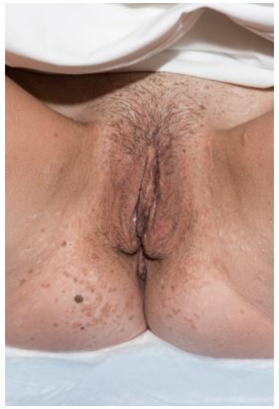

G M12

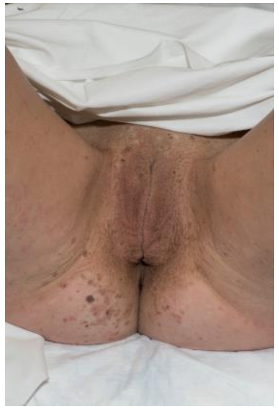

P M12

# M05

Right hand

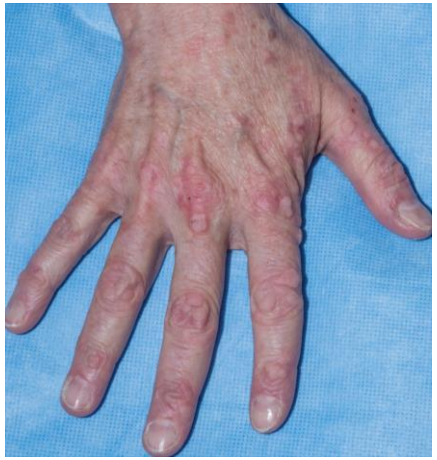

6/2013

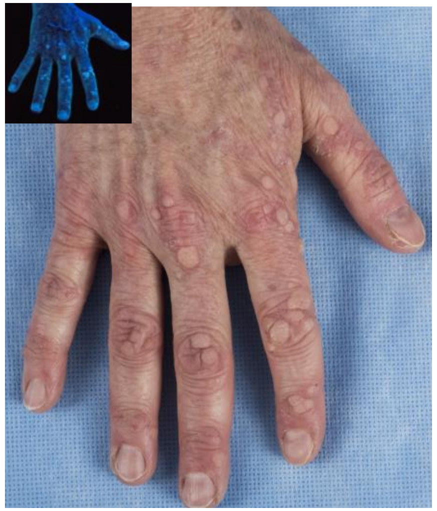

1/2015

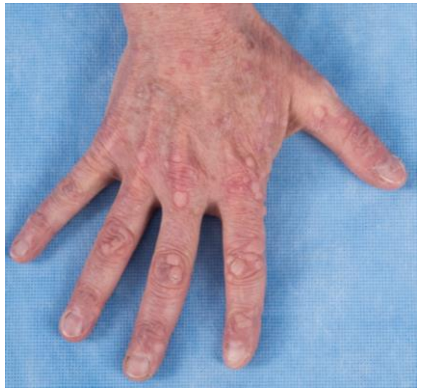

P d0 3/2015

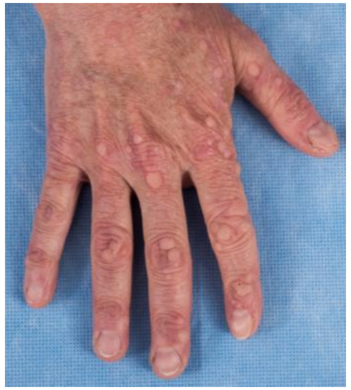

P M0

P M4

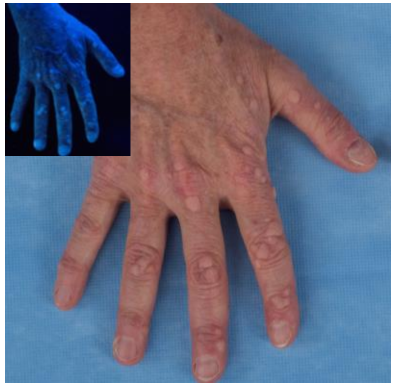

P M8

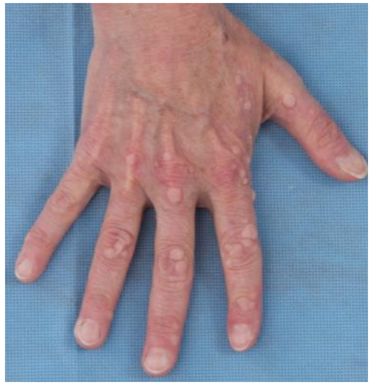

P M12

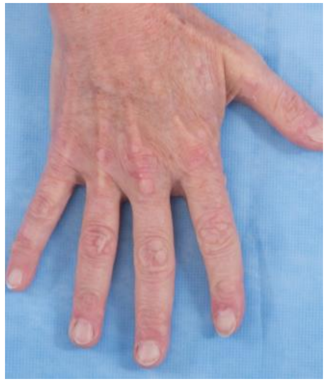

G M0

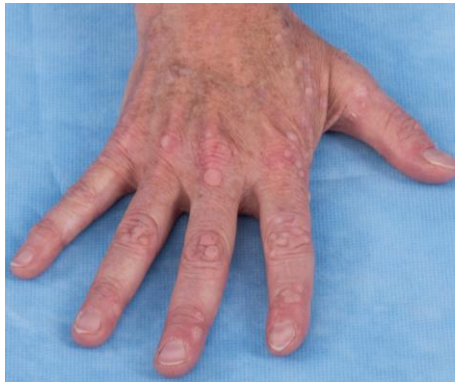

G M4

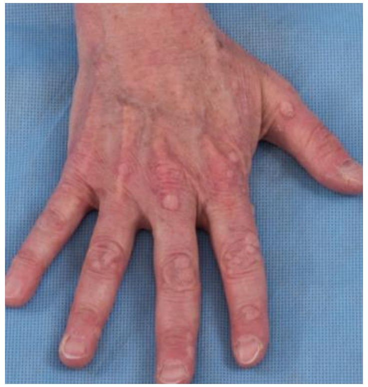

G M8

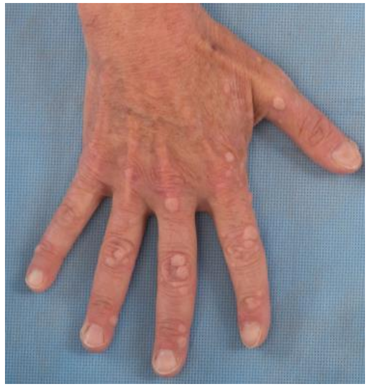

G M12

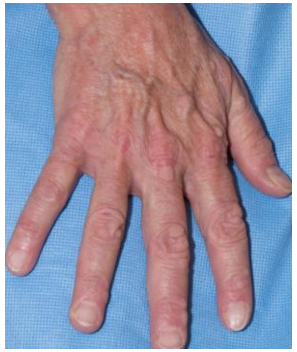

11/2017

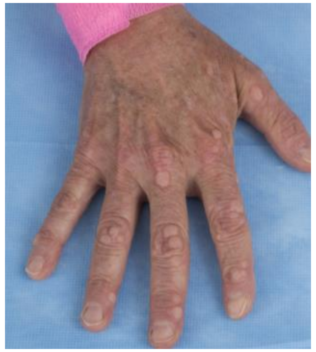

7/2018

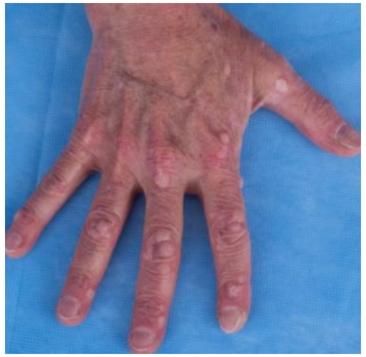

6/2019

# M05
Left hand

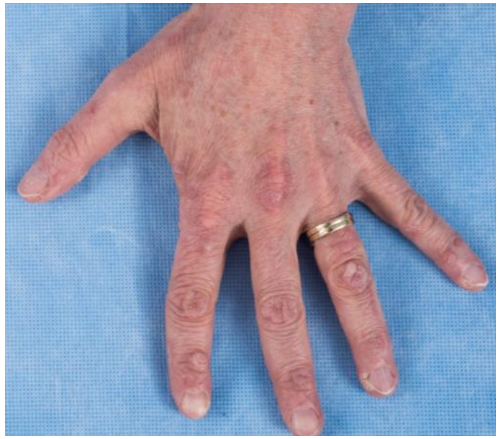
P d0

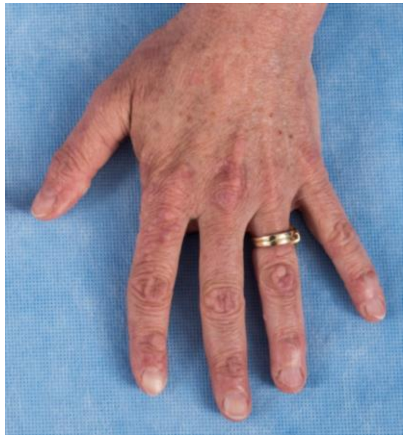
P M0

P M4

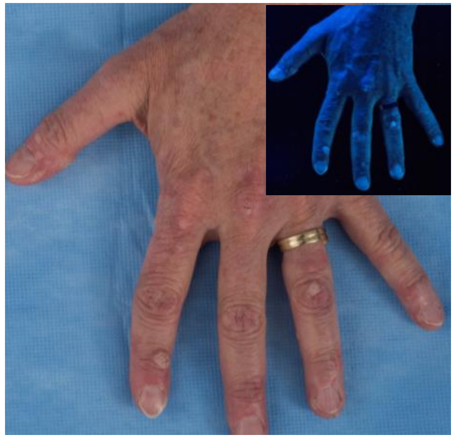
P M8

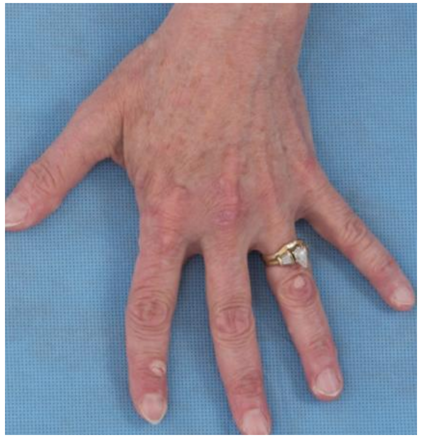
P M12

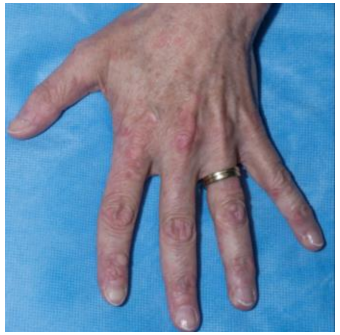
6/2013

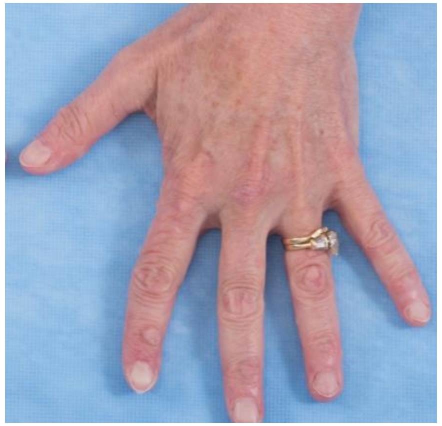
G M0

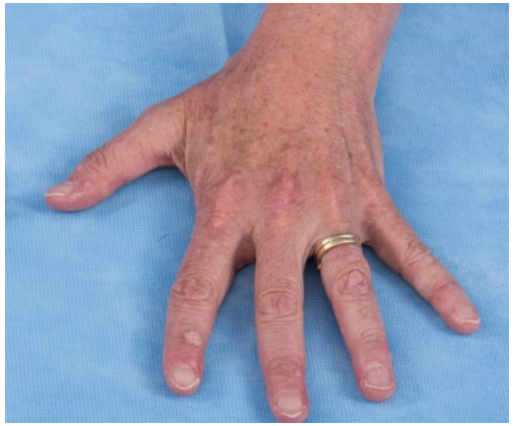
G M4

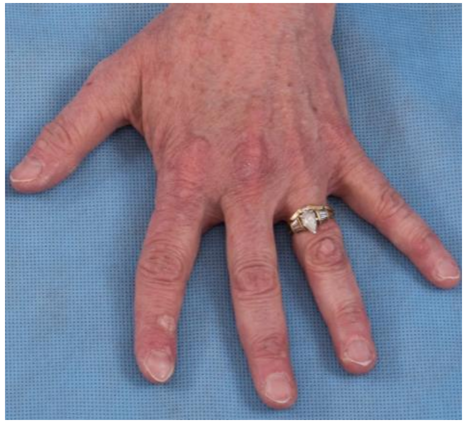
G M8

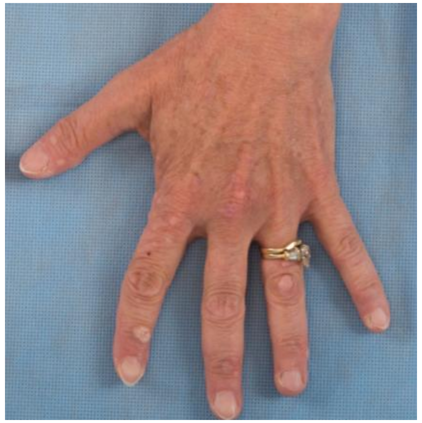
G M12

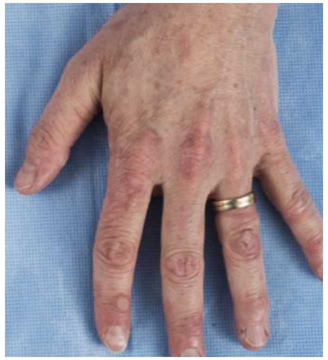
1/2015

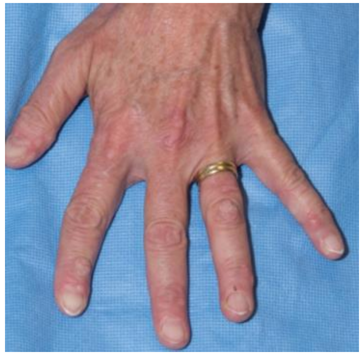
11/2017

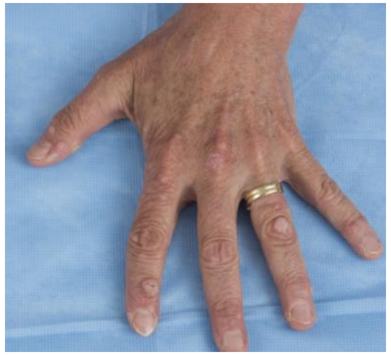
7/2018

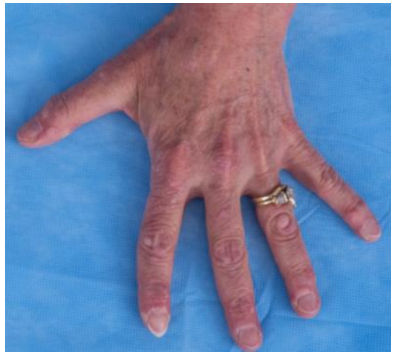
6/2019

# M05

Left pinky

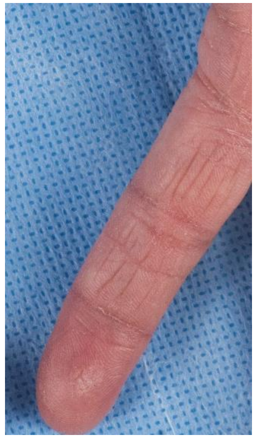

P d0

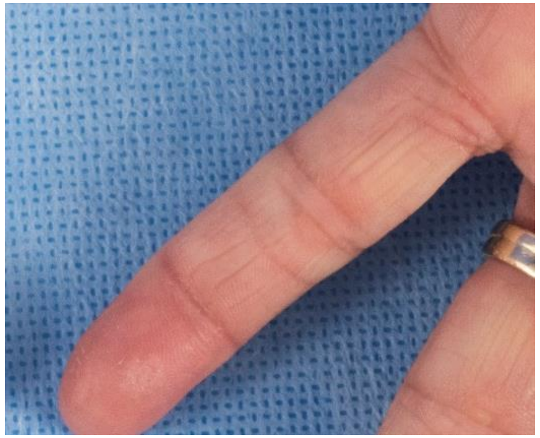

P M0

P M4

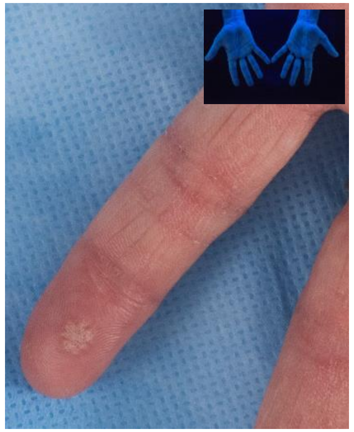

P M8

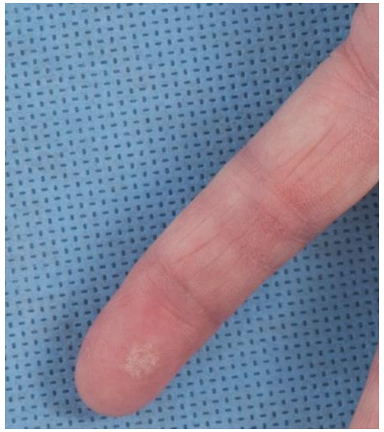

P M12

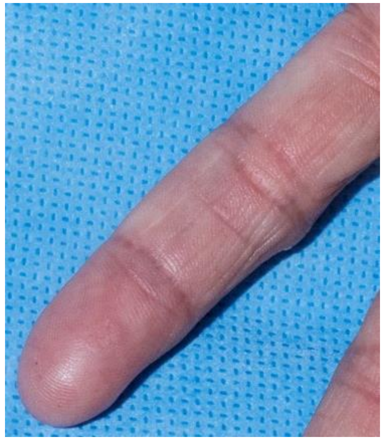

6/2013

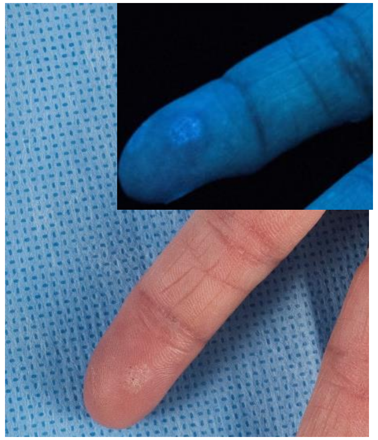

1/2015

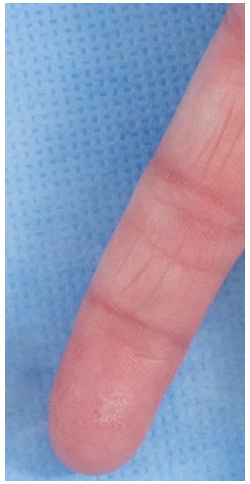

G M0

G M4

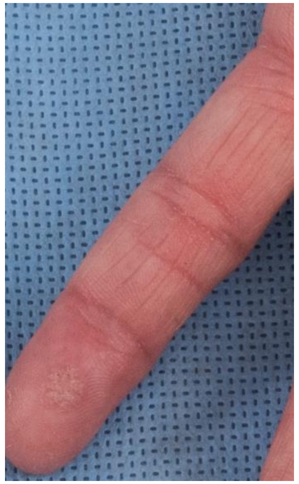

G M8

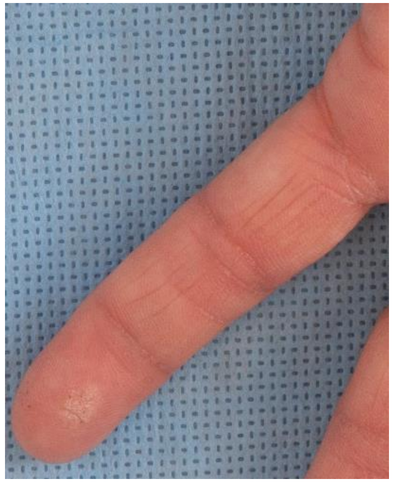

G M12

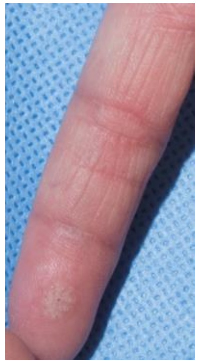

11/2017

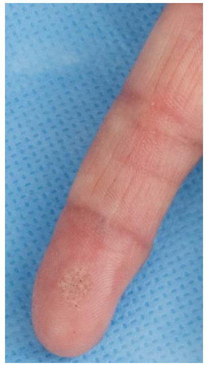

7/2018

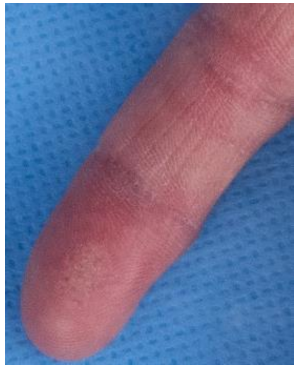

6/2019

# M05 Left foot plantar surface

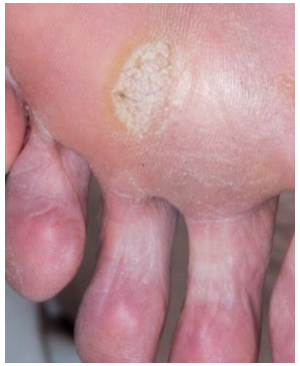

6/2013

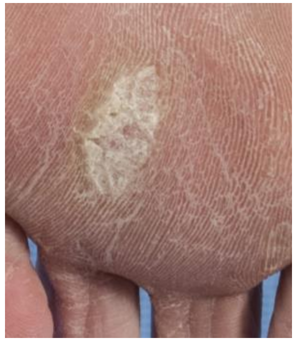

1/2015

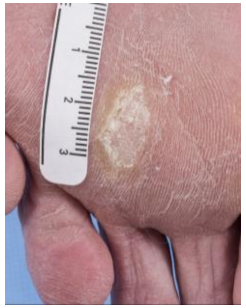

P d0

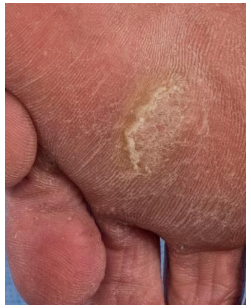

P M0

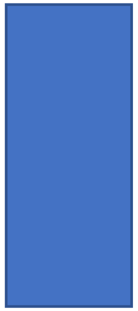

P M4

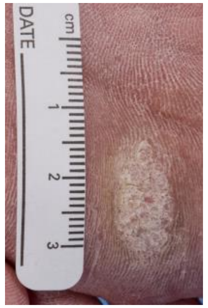


P M8

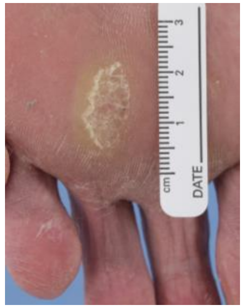


P M12

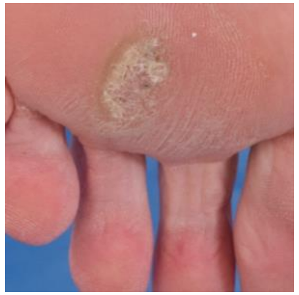

G M0

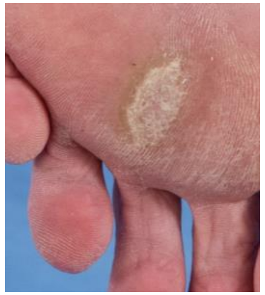

G M4

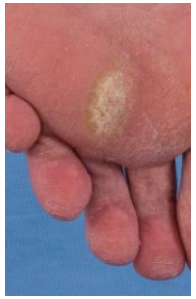

G M8

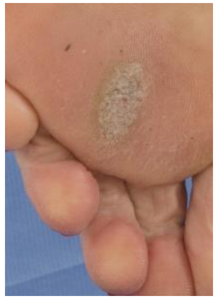

G M12

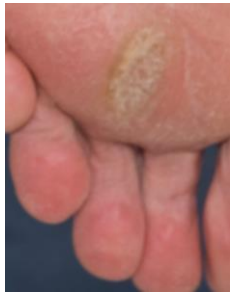

11/2017

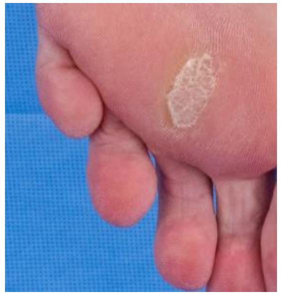

7/2018

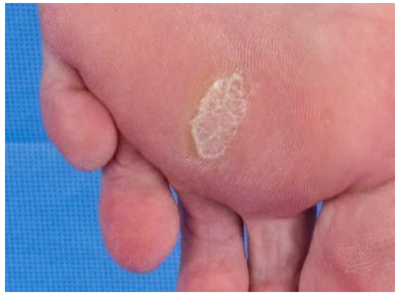

6/2019

# M06 Right thumb

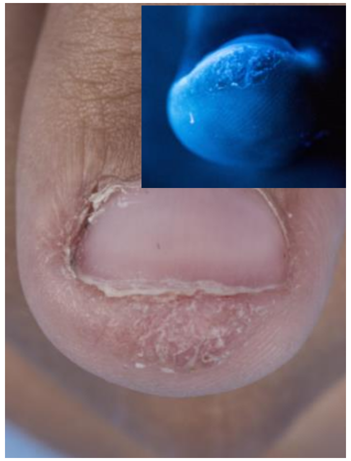
P d0

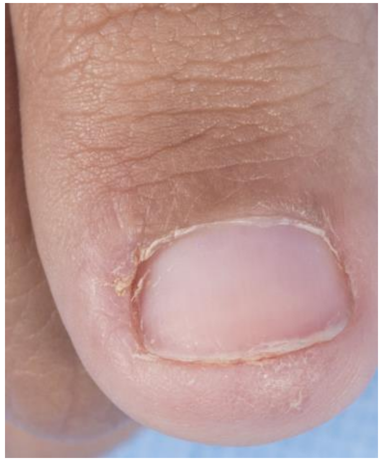
P M0

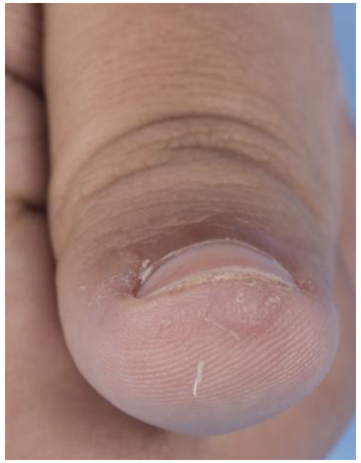
P M4

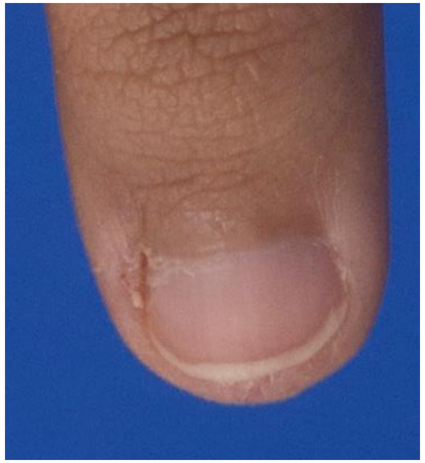
P M8

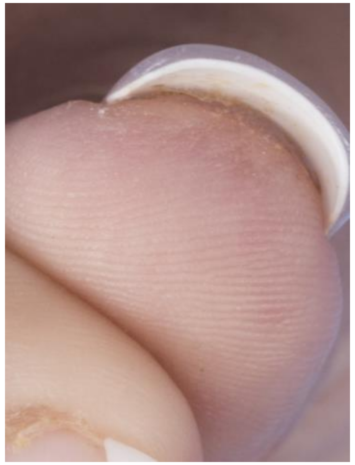
P M12

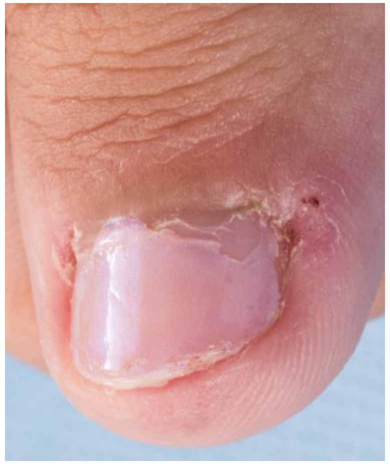
G M0

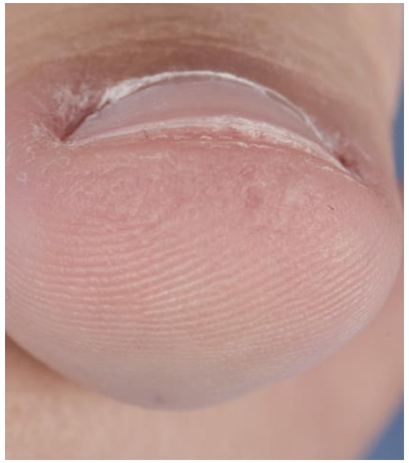
G M4

G M8

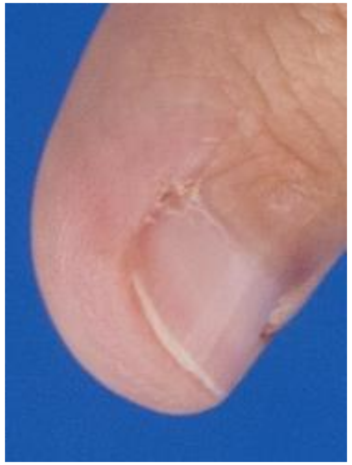
G M12

# M06

Left foot dorsum

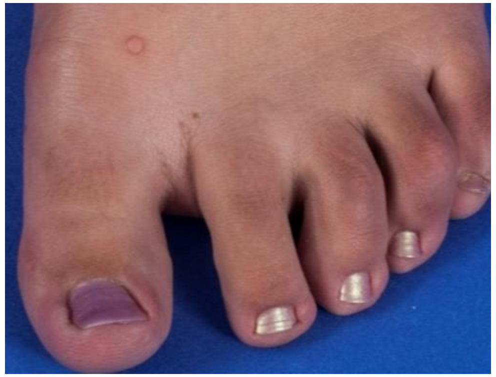

P d0

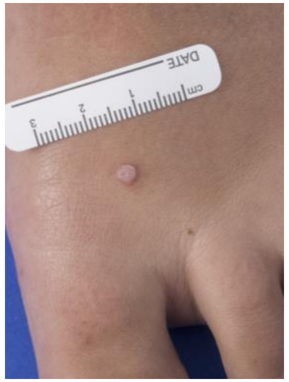

P M0

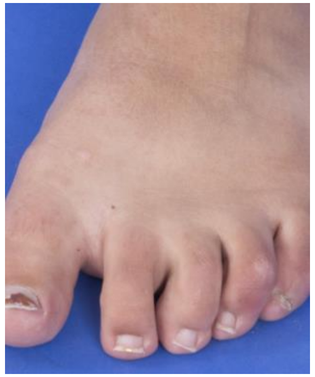

P M4

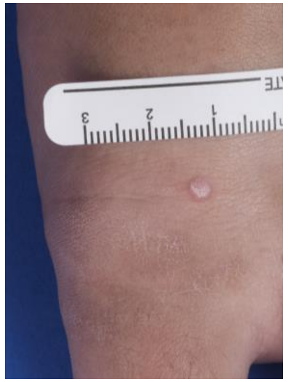

P M12

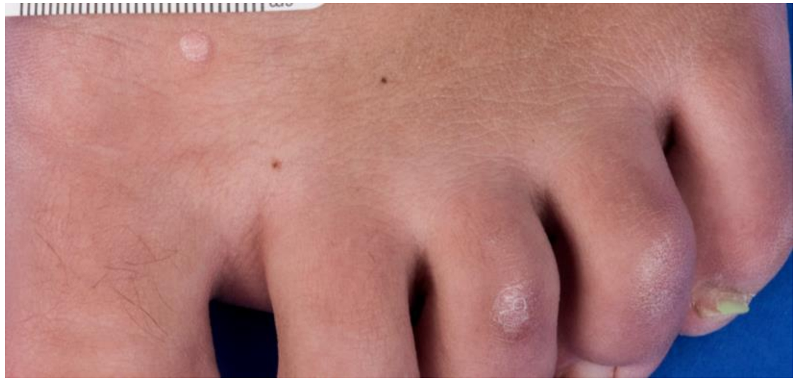

G M0

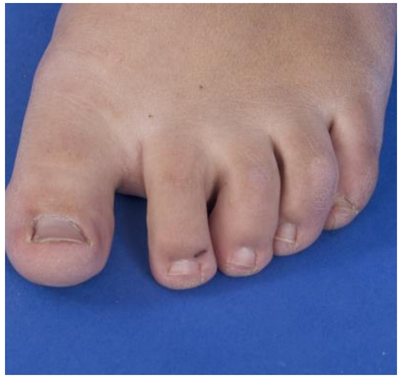

G M4

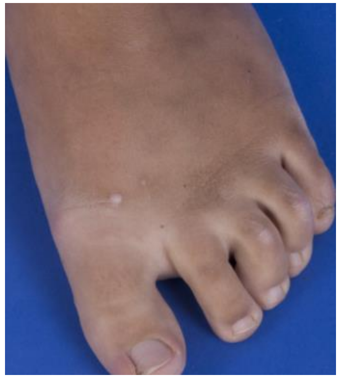

G M8

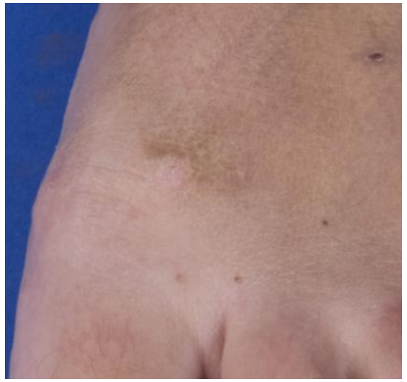

G M12

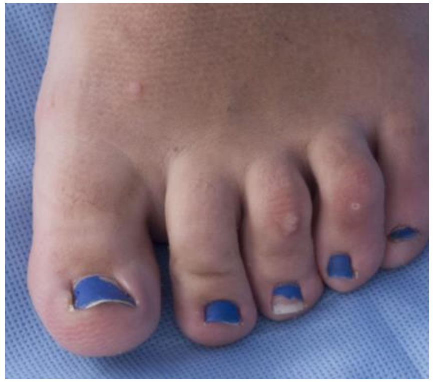

12/2019

# M06 Right elbow

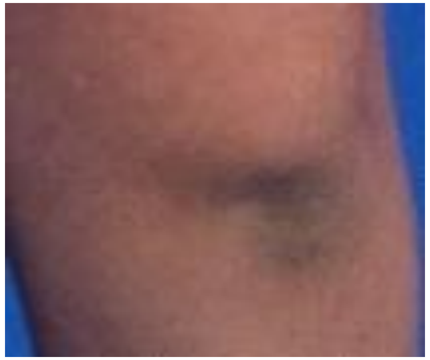

P d0

P M4

P M8

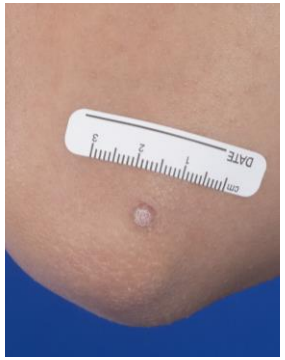

P M12

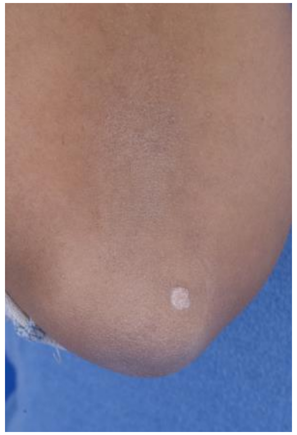

12/2019

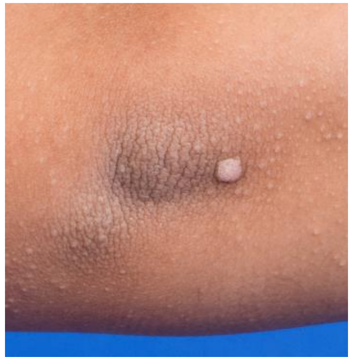

G M0

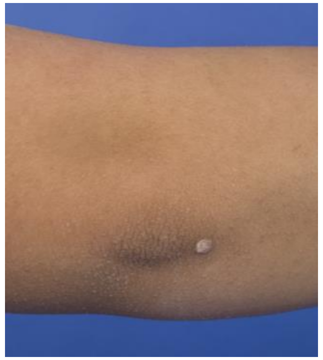

G M4

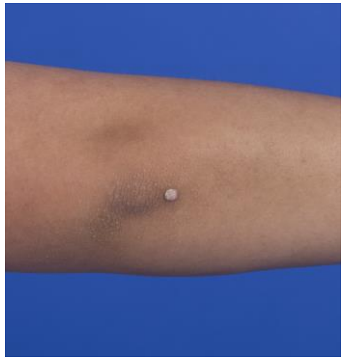

G M8

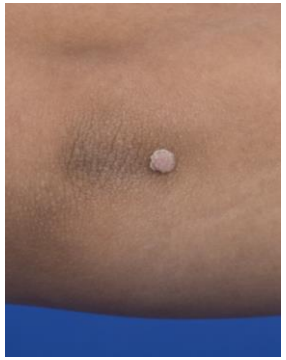

G M12

# M07 Right elbow

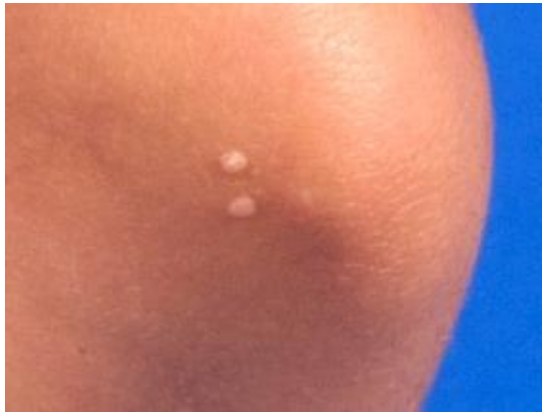

8/14

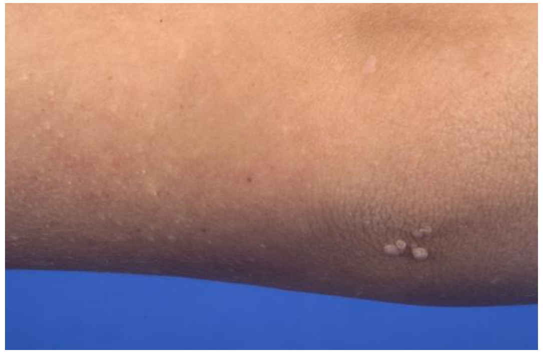

6/15

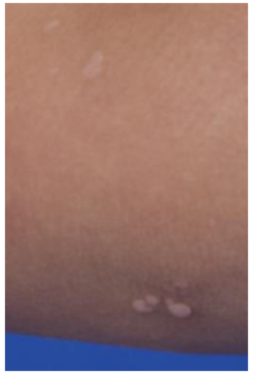

G d0
10/2015

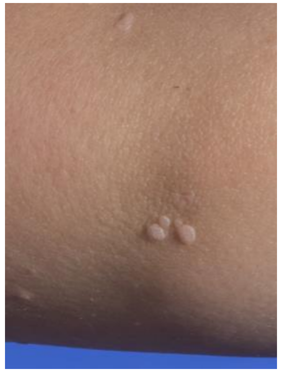

G M0

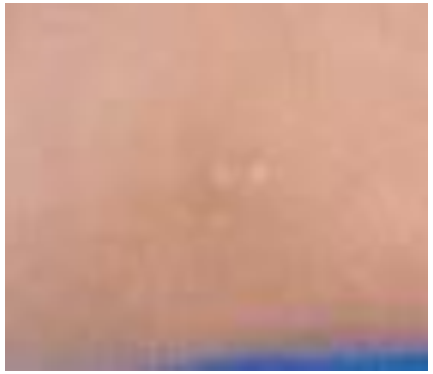

G M4

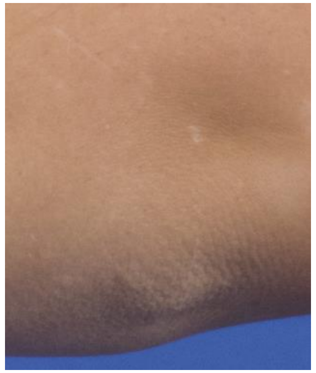

G M12

P M0
Failed on P

# M07 Left elbow

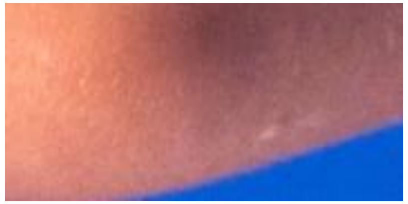

8/14

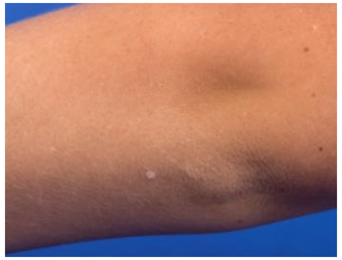

6/15

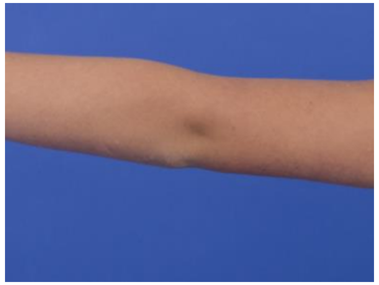

7/17

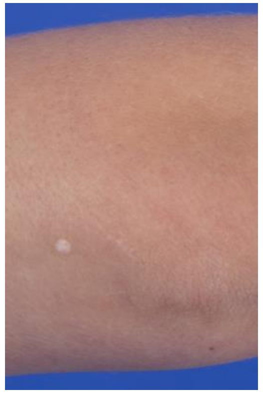

G d0

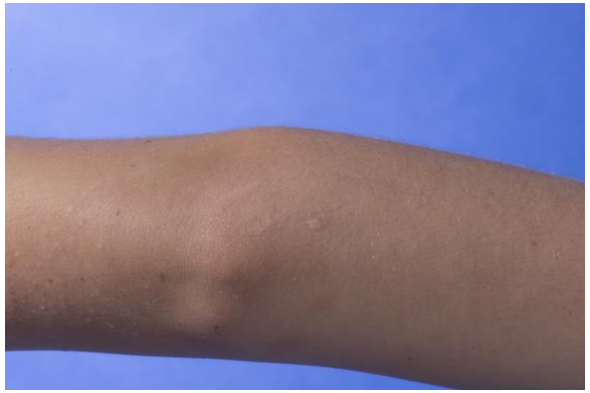

G M0

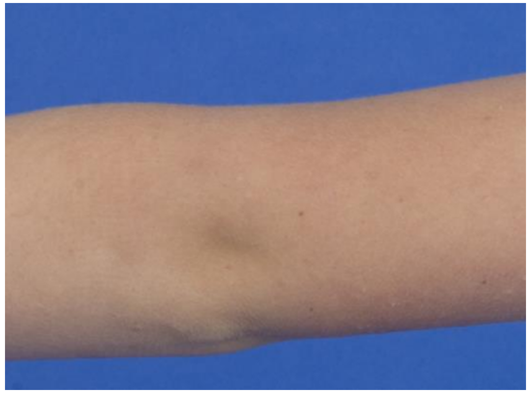

G M4

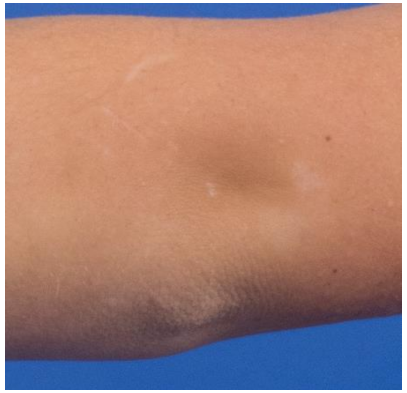

G M12

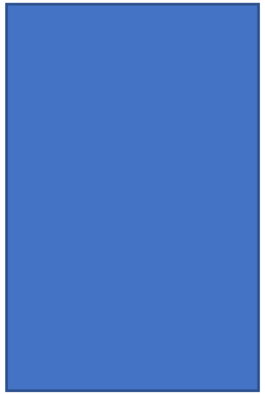

P M0 na
Failed on P

# M07 Buttocks, perirectal area

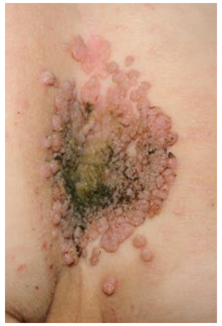

11/09
**Tagamet**
**Aldara**

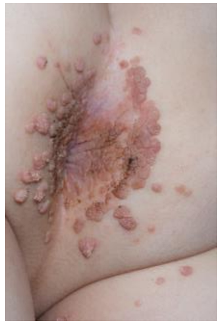

8/10
**Podophyllin**
**IVIG**

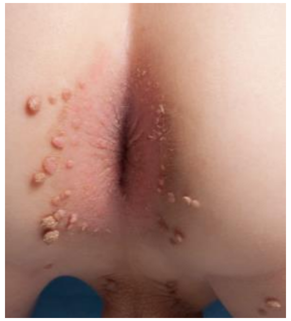

7/11

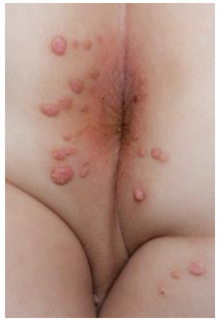

6/12
**Pre-surgical**
**Removal**

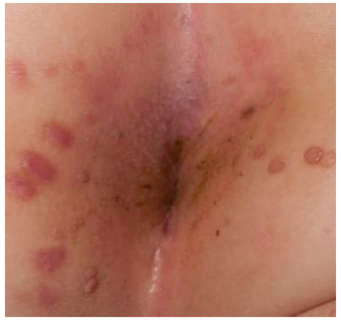

9/7/12
**Post-surgical**
**Removal**

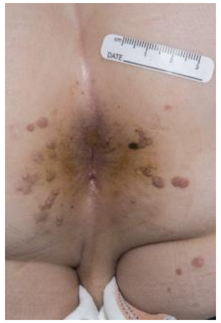

4/13

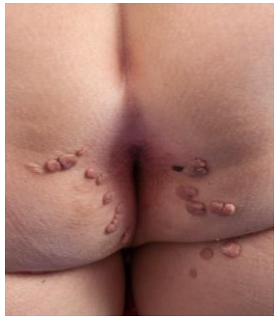

8/14

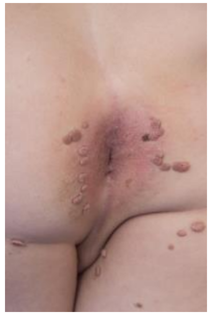

6/15

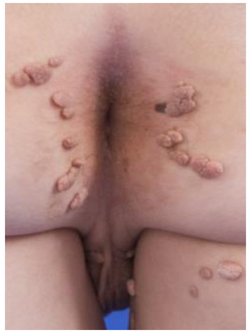

G d0

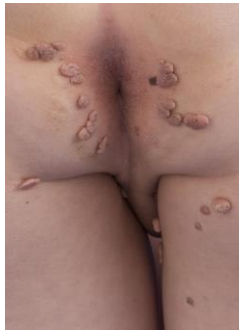

G M0

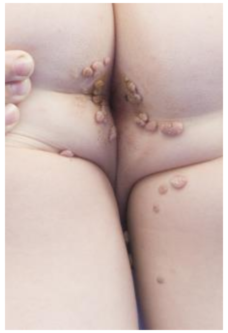

G M4

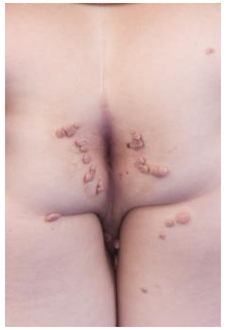

G M8

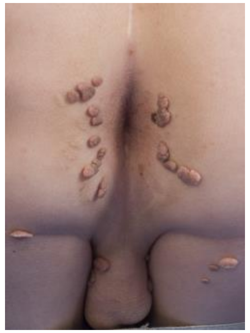

G M12

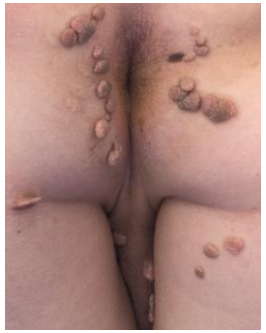

P M0
Failed on P

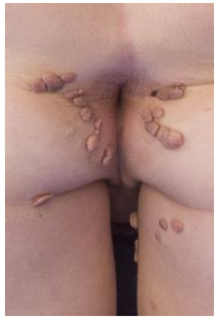

7/17

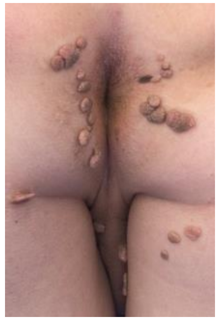

12/17

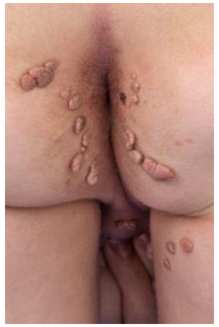

4/18

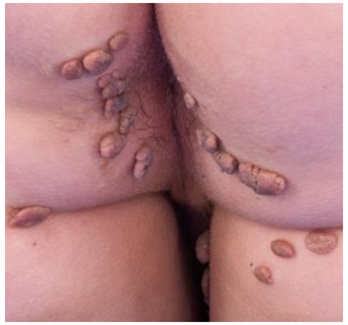

2/19

# M07 Right hand dorsum

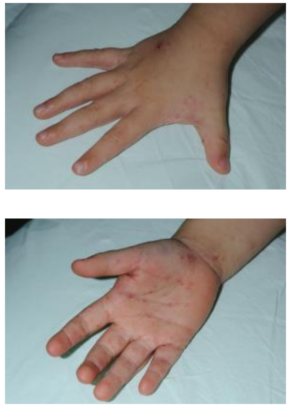

11/09

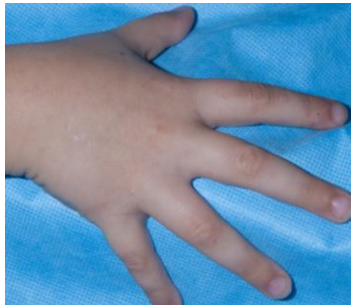

8/10

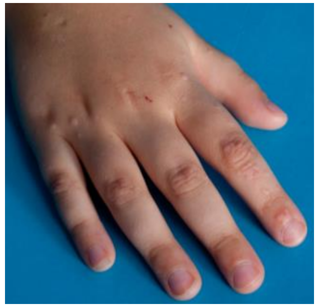

8/11

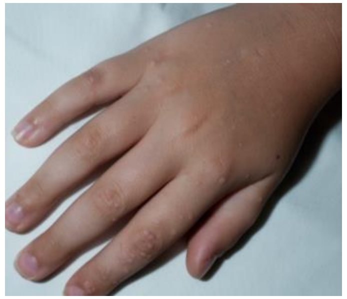

6/12

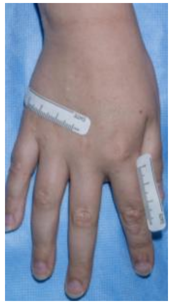

4/13

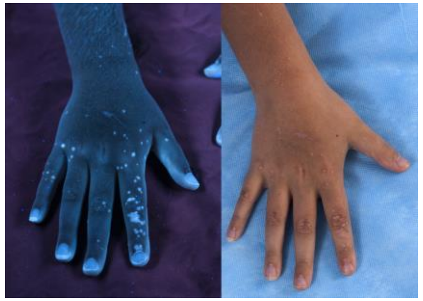

8/14

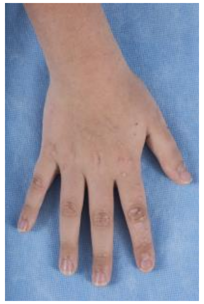

6/15

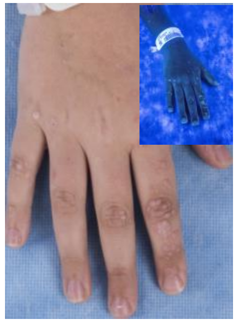

G d0

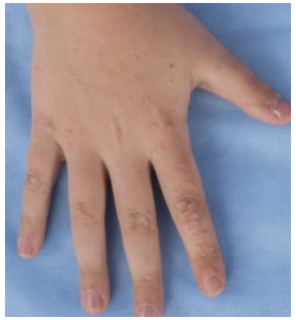

G M0

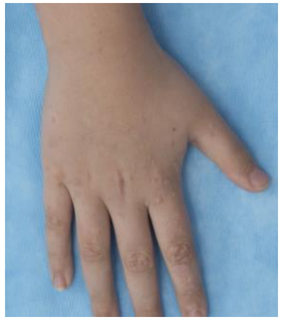

G M4

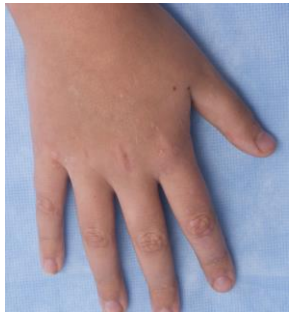

G M8

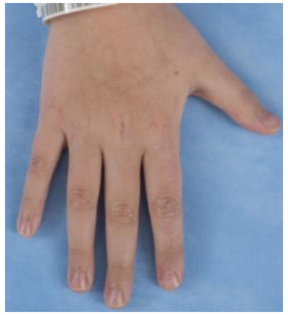

G M12

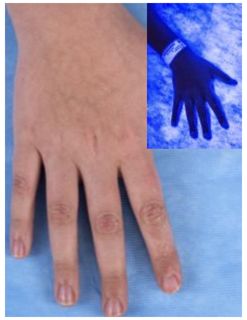

P M0
Failed on P

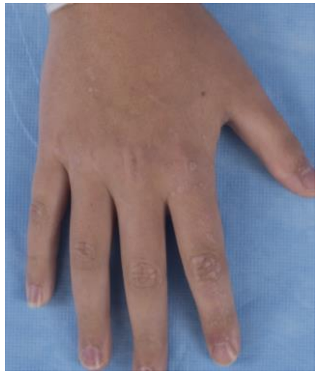

7/17

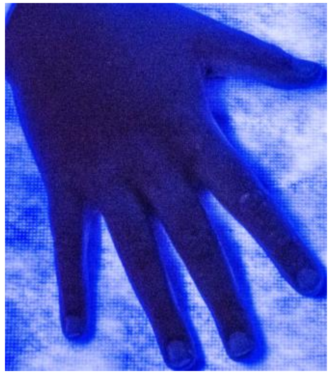

12/17

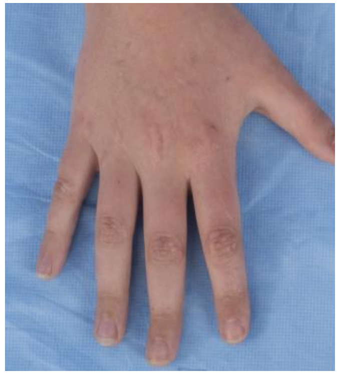

4/18

# M07 Left hand dorsum

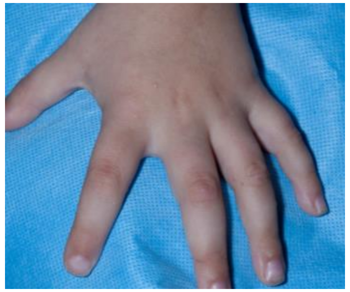

8/10

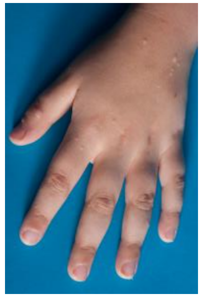

7/11

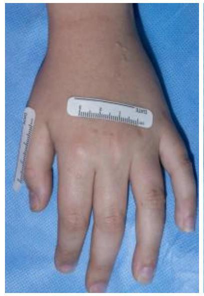

4/13

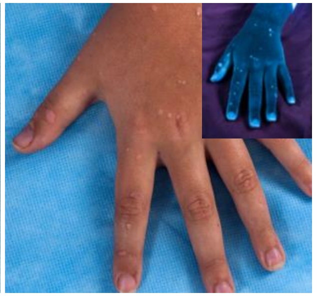

8/14

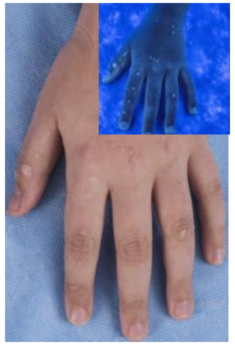

G d0

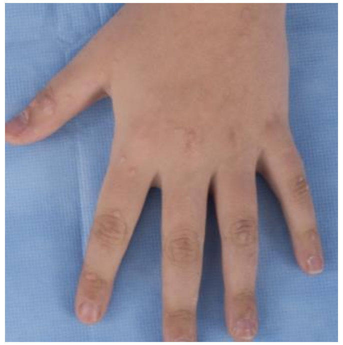

G M0

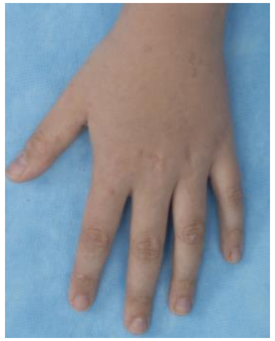

G M4

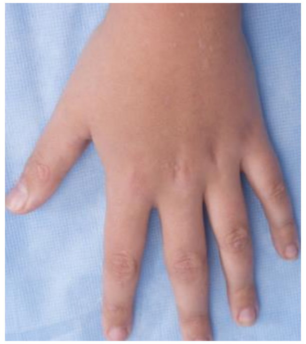

G M8

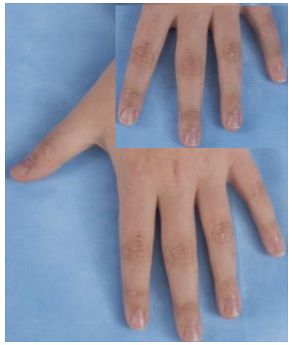

G M12

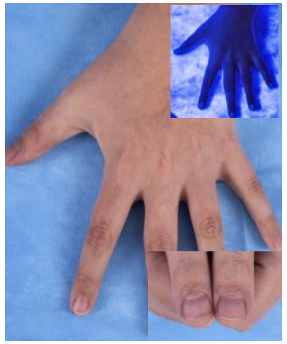

P M0
Failed on P

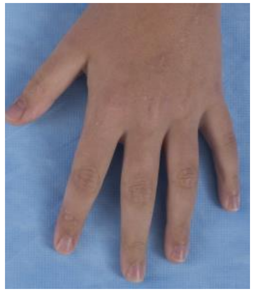

7/17

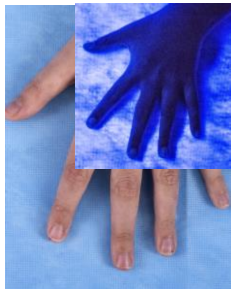

12/17

# M07

Left thumb

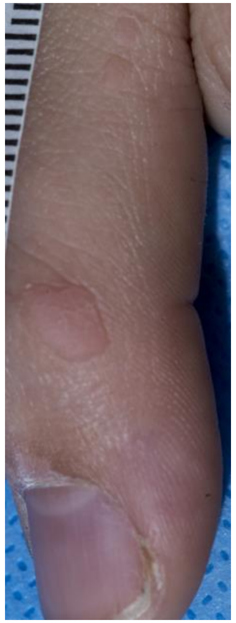

7/2013

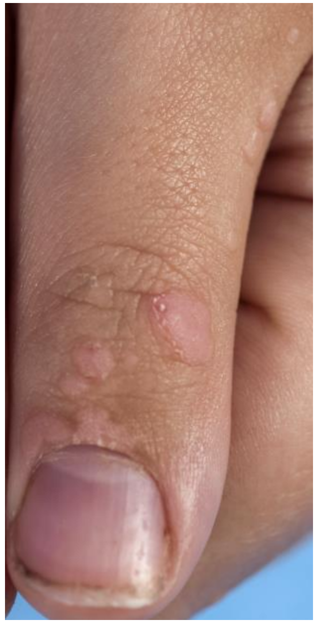

8/2014

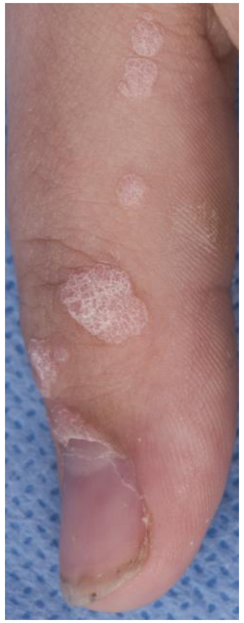

G d0 10/2015

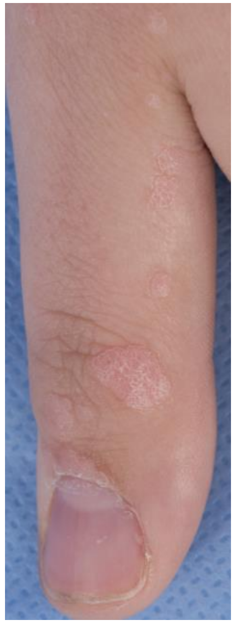

G M0

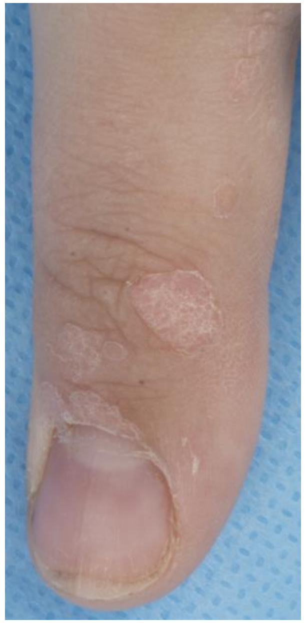

G M4

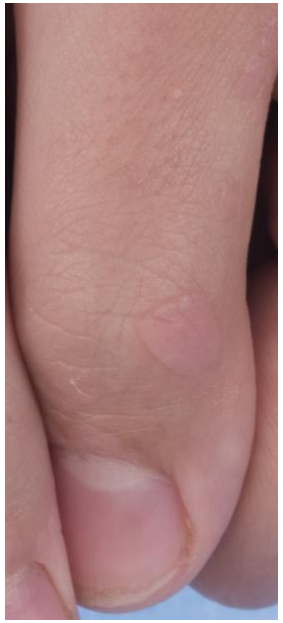

G M8

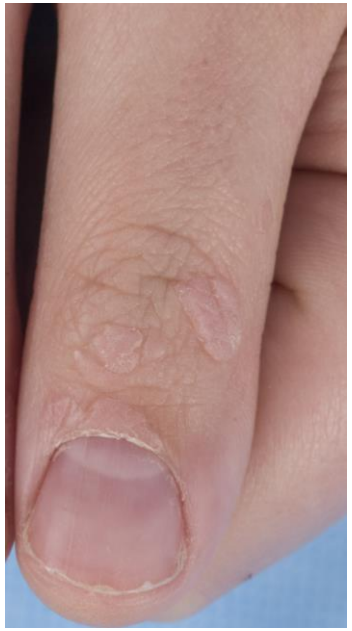

G M12

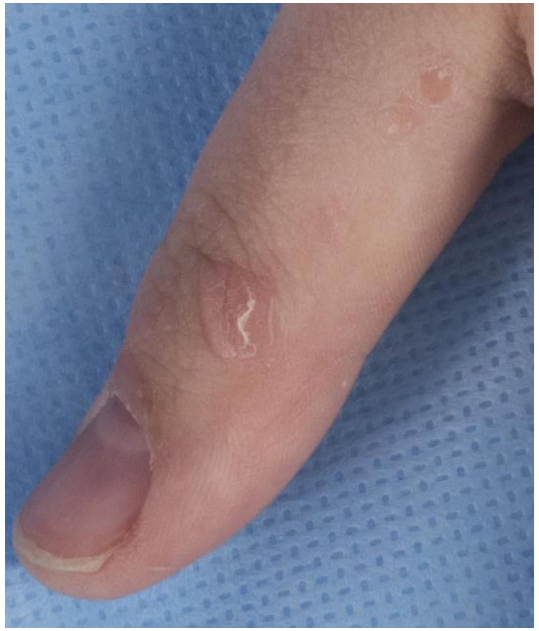

P M0 (failed on P)

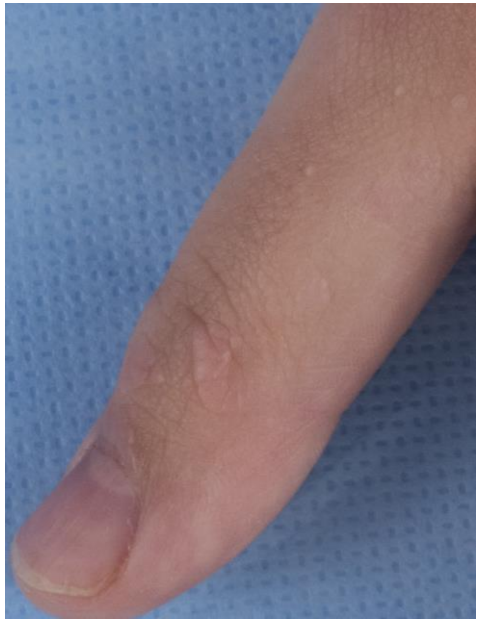

7/2017

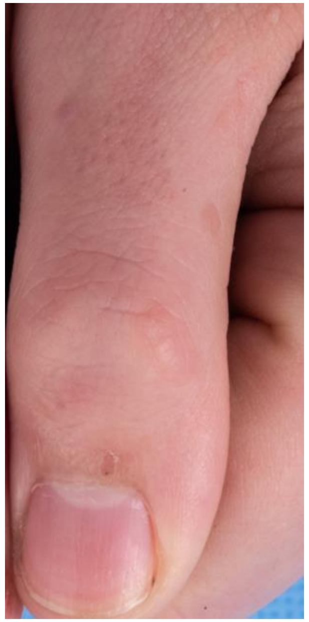

2/2019

# M07 Left foot dorsum

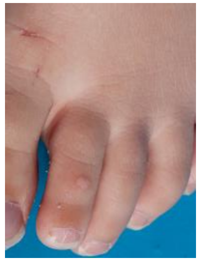
7/11

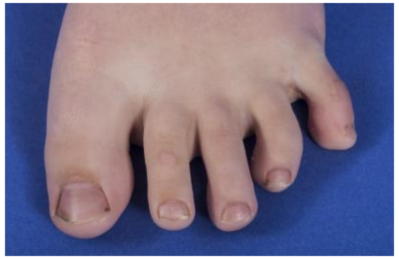
6/15

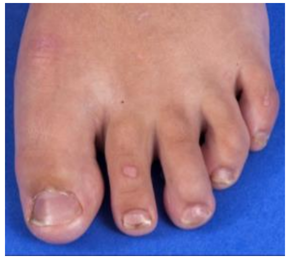
8/14

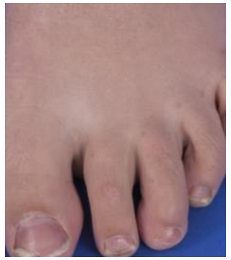
G d0

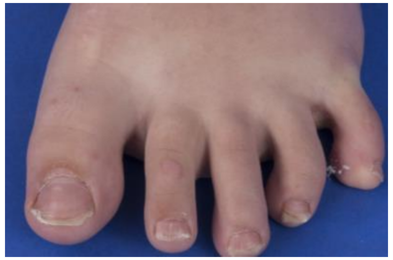
G M0

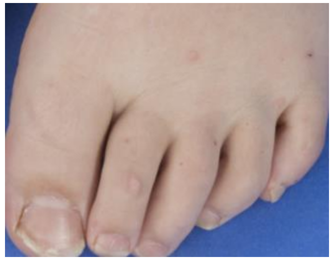
G M4

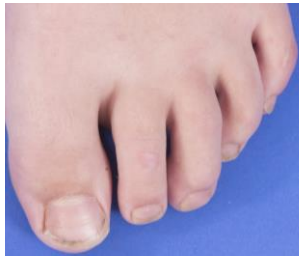
G M8

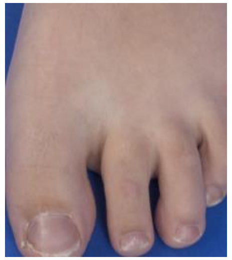
G M12

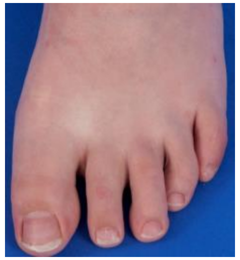
P M0

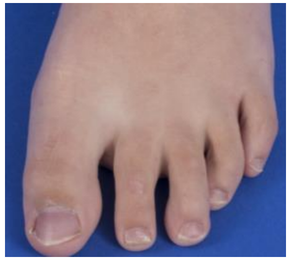
7/17

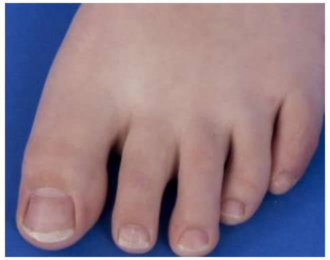
4/18

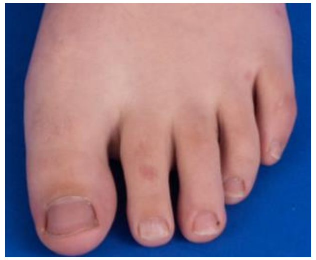
2/19

# M09 Left foot, plantar surface

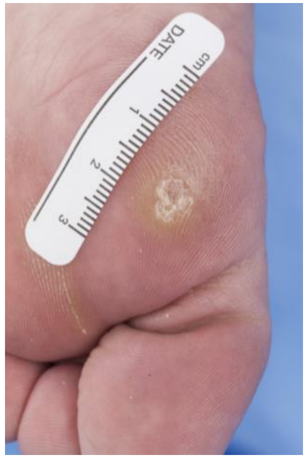

P d0 3/22/16
(Failed on P)

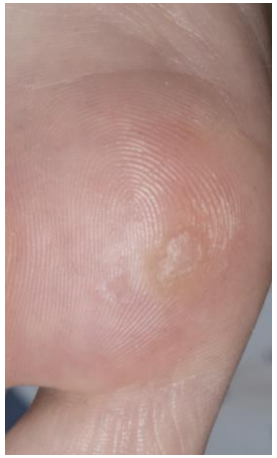

G d0

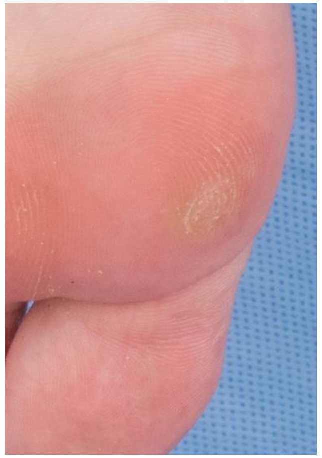

G M0

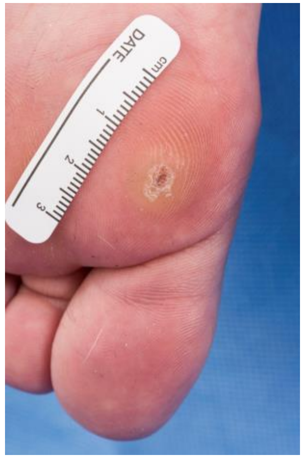

G M4

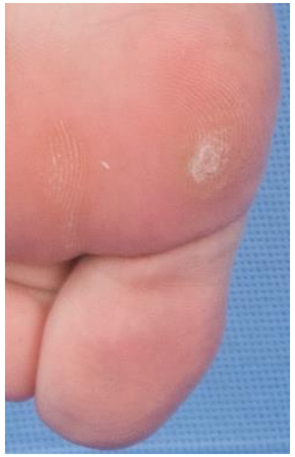

G M8

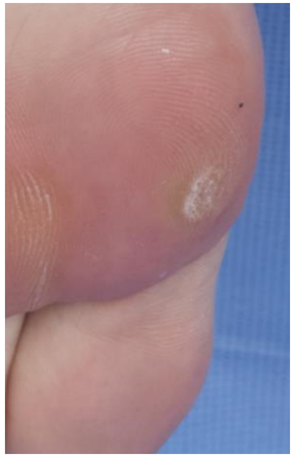

G M12

# M09 **Right** foot, plantar surface

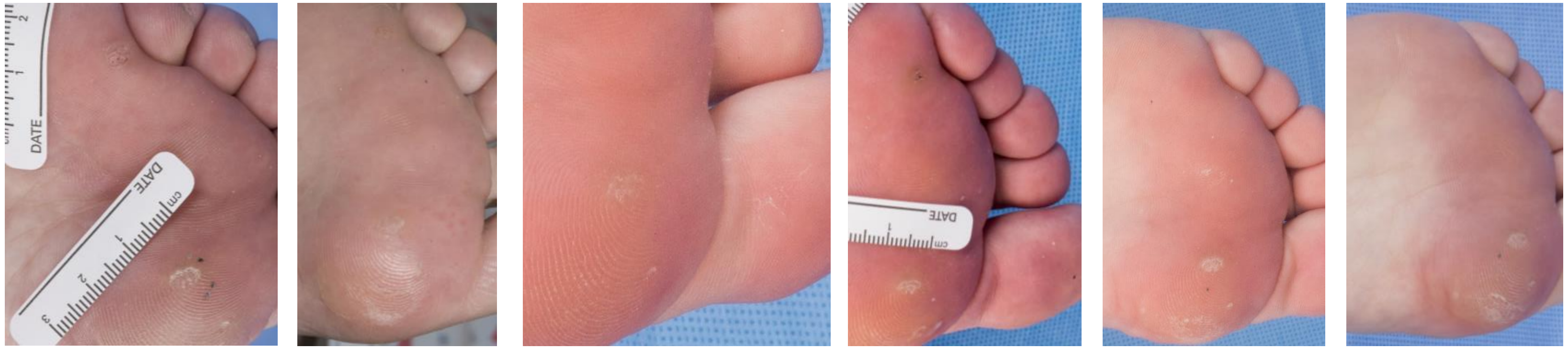


P d0 (Failed on P) | G d0 | G M0 | G M4 | G M8 | G M12

# M12 Left thumb

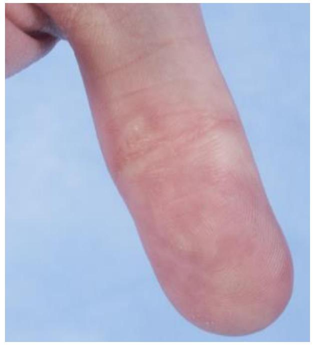

P d0

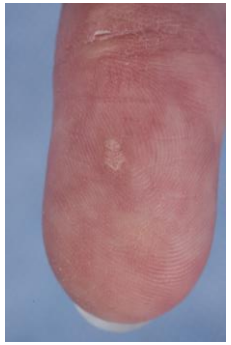

P M0

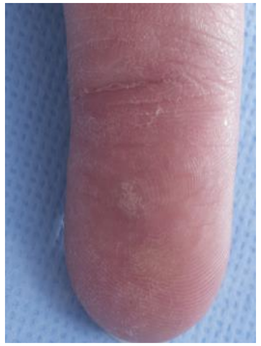

P M4

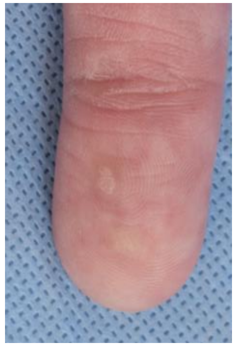

P M8

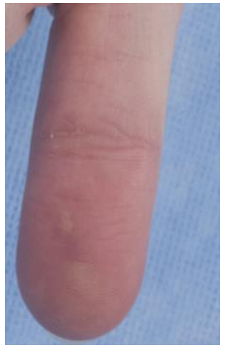

P M12

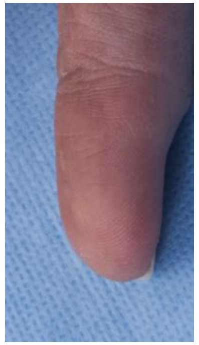

G M0

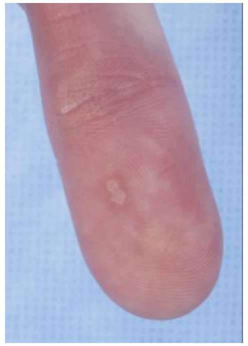

G M4

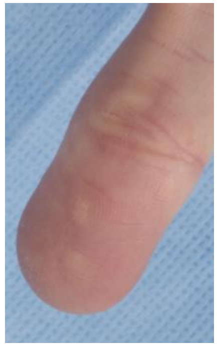

G M8

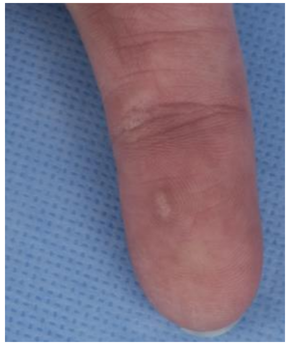

G M12

# M12 Right toes

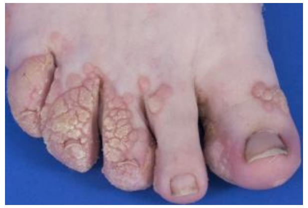
P d0
5/25/16

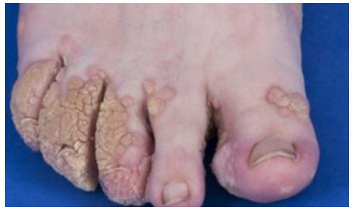
P M0

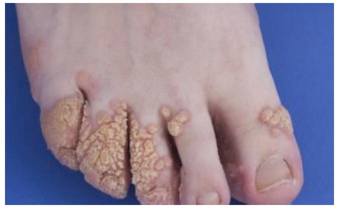
P M4

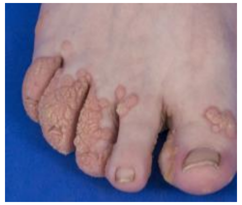
P M8

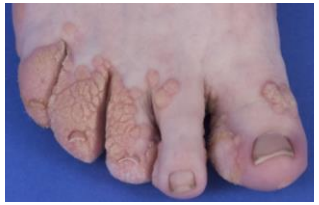
P M12

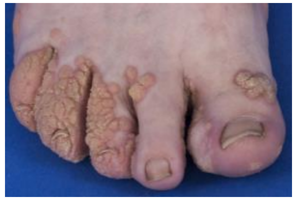
G M0

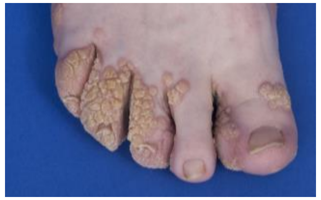
G M4

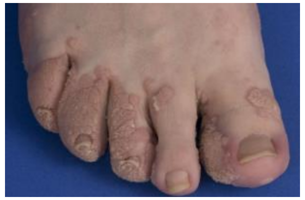
G M8

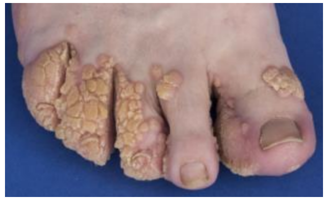
G M12
11/28/18

4/22

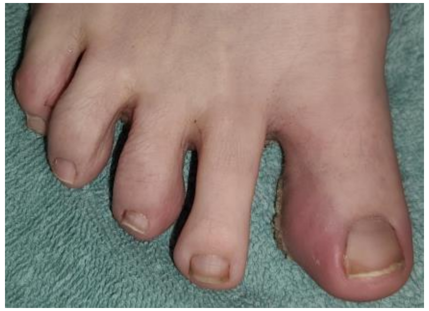

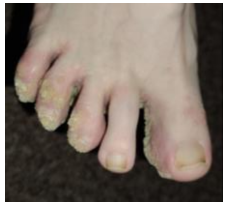
7/20

# M12 Right thumb, subungual region

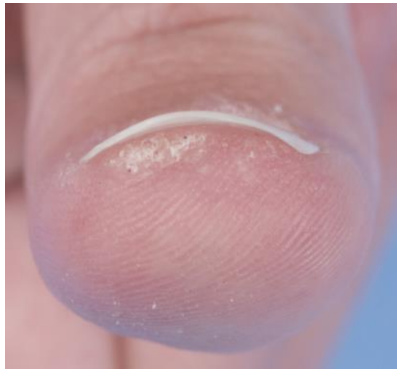
P d0

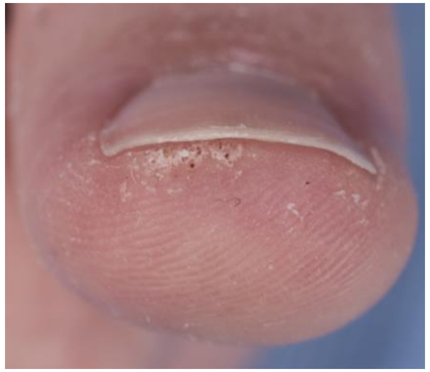
P M0

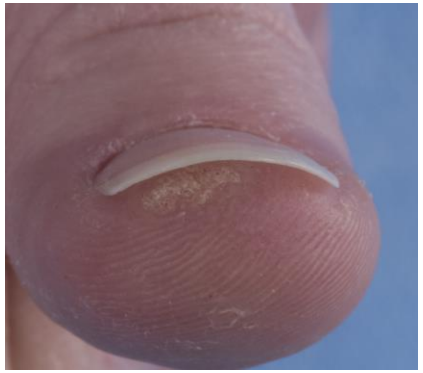
P M4

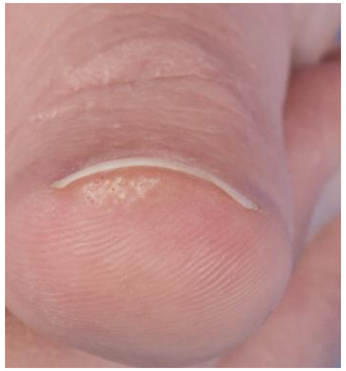
P M8

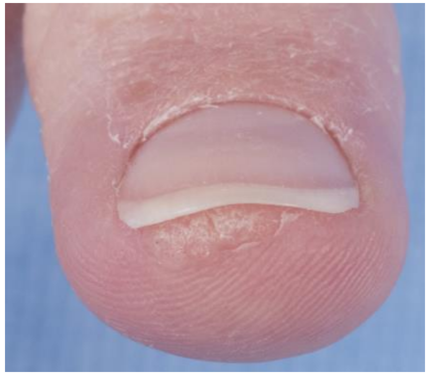
P M12

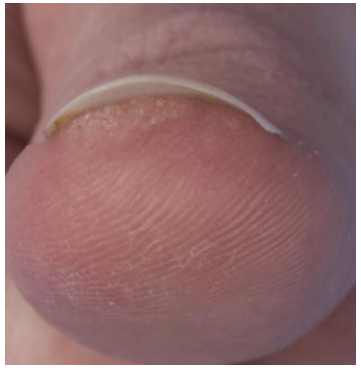
G M0

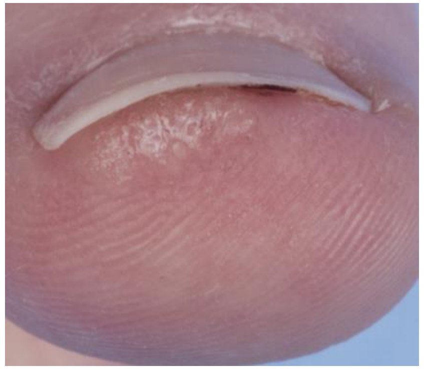
G M4

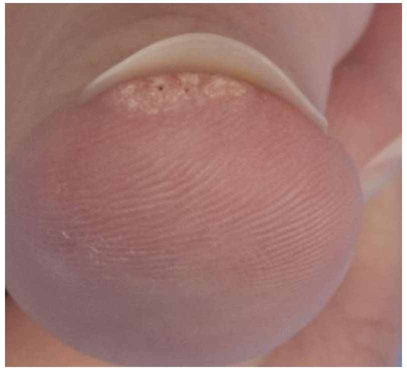
G M8

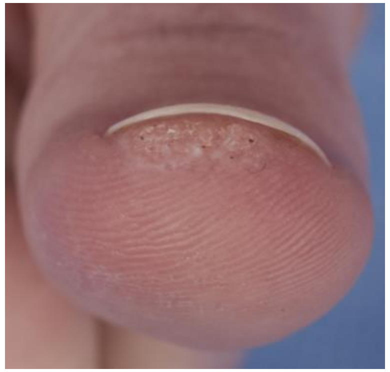
G M12

# M12 Right sole

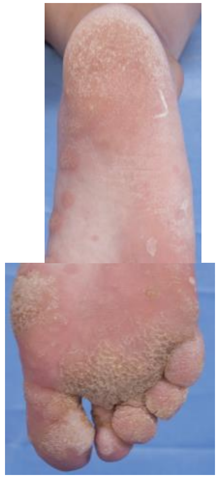

P d0

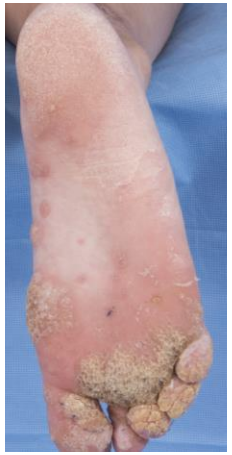

P M0

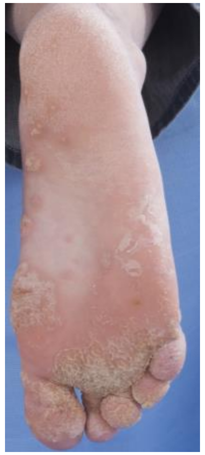

P M4

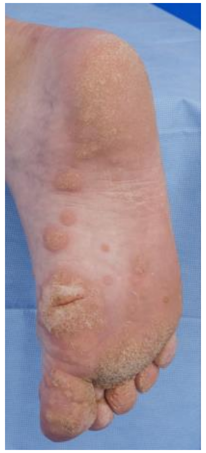

P M8

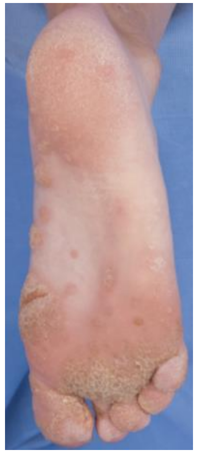

P M12

4/22

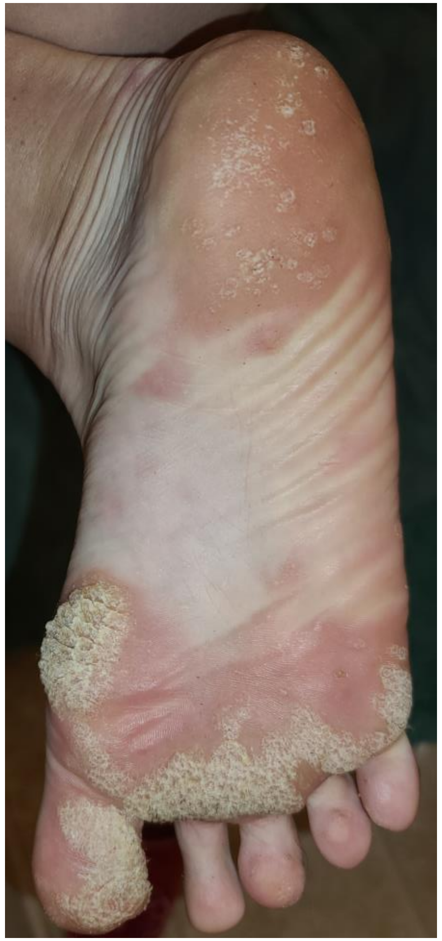

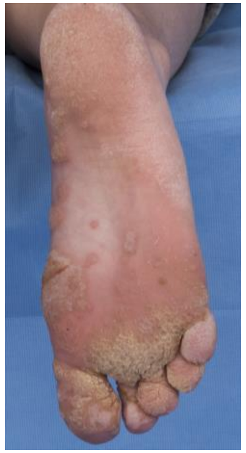

G M0

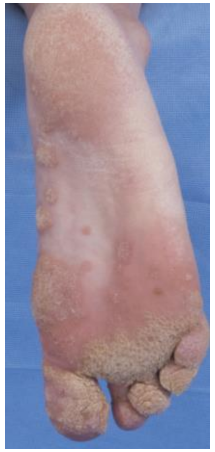

G M4

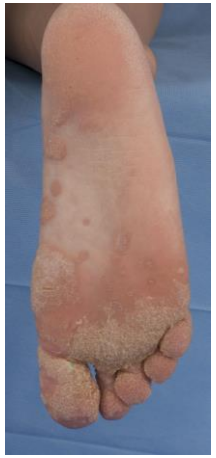

G M8

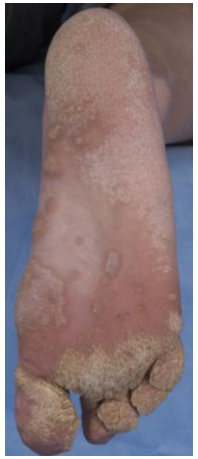

G M12

# M12 Left sole

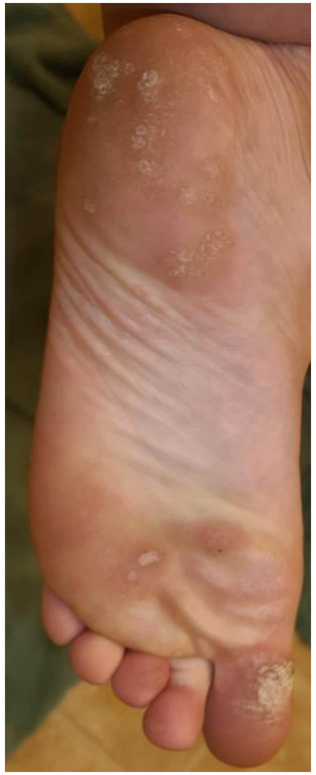

4/22

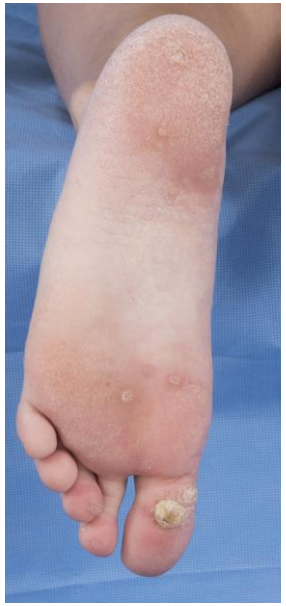

P M0

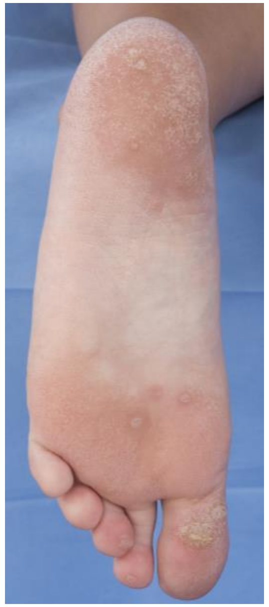

P d0

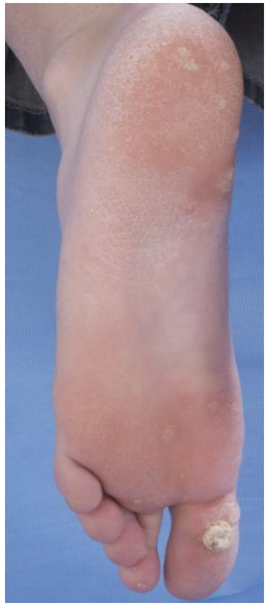

P M4

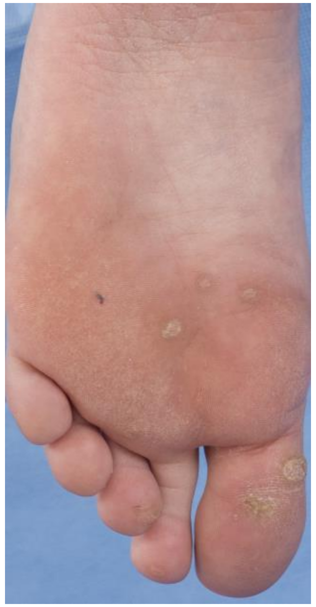

P M8

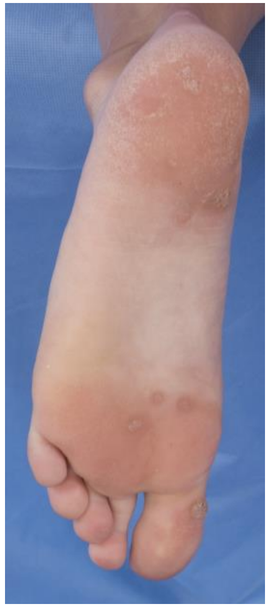

P M12

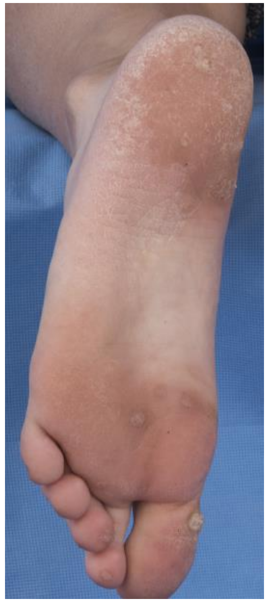

G M0

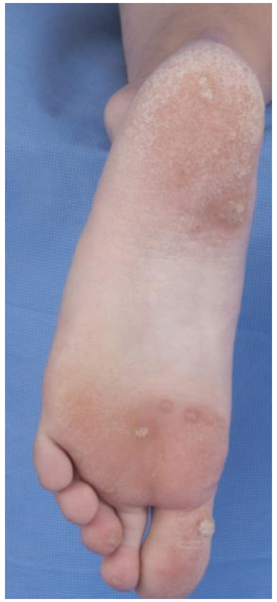

G M4

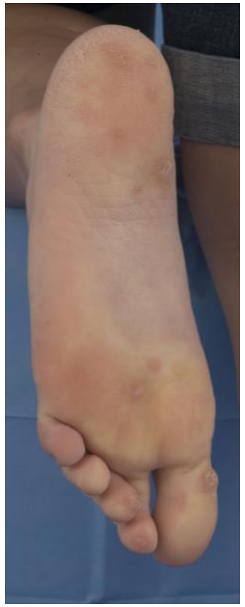

G M8

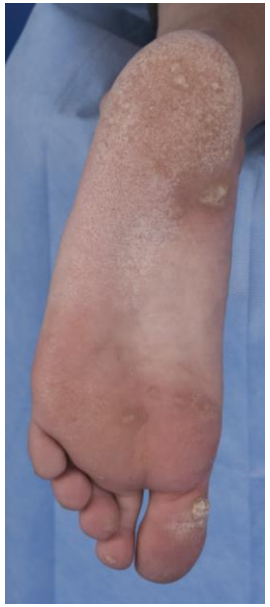

G M12

# M15 Right elbow

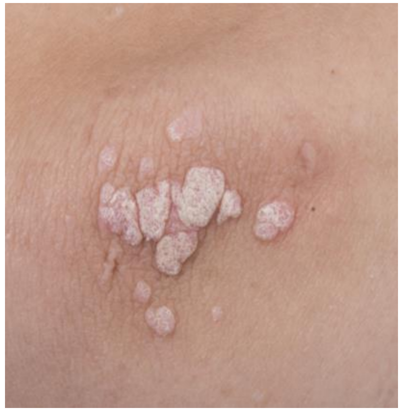

G d0

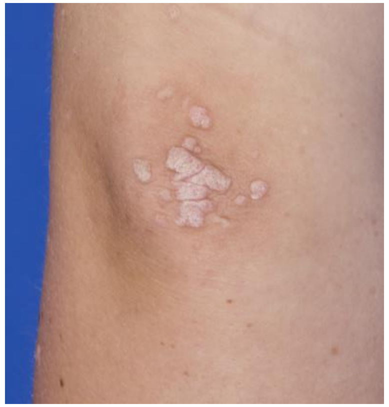

G M0

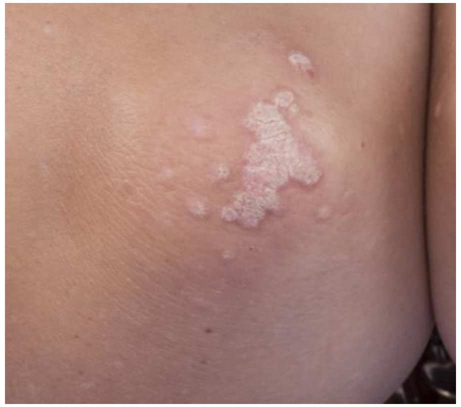

G M4

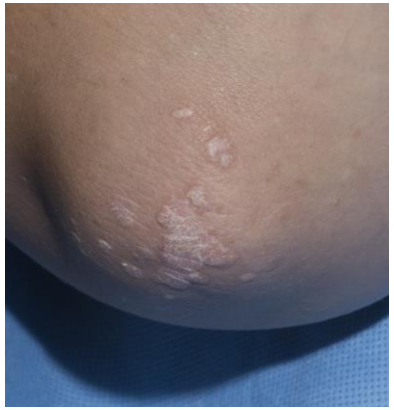

G M8

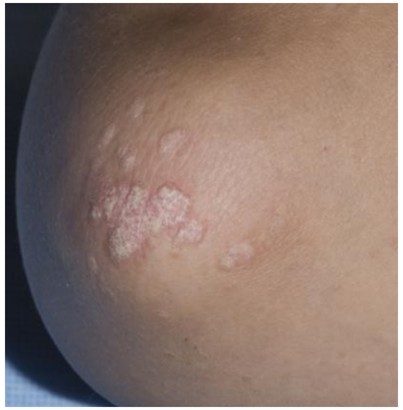

G M12

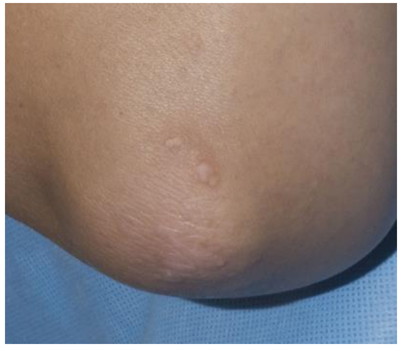

P M0

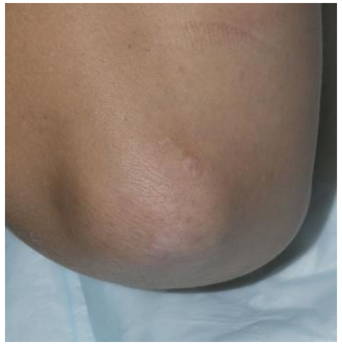

P M4

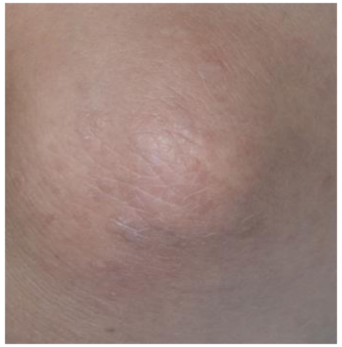

P M8

# M15 Left elbow

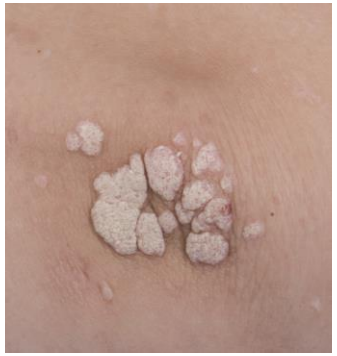

G d0

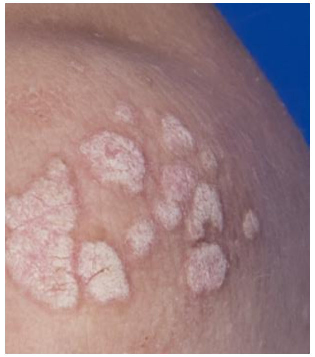

G M0

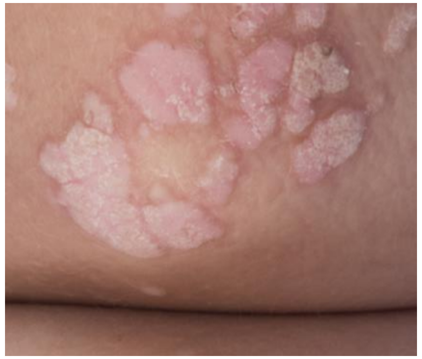

G M4

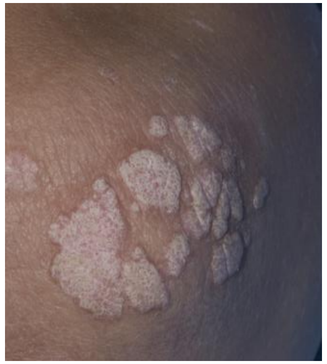

G M8

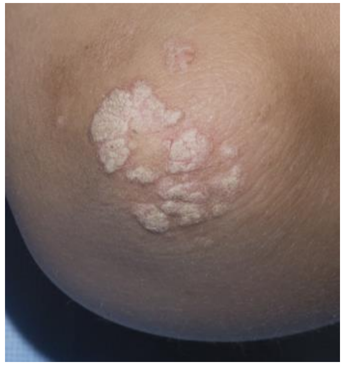

G M12

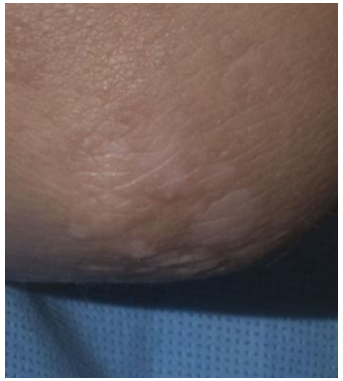

P M0

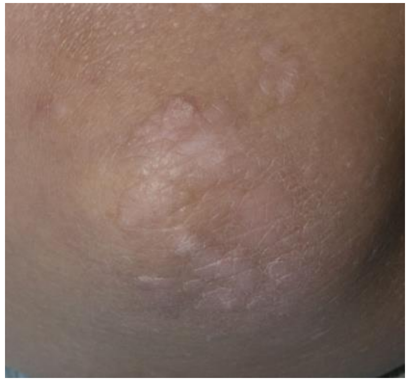

P M4

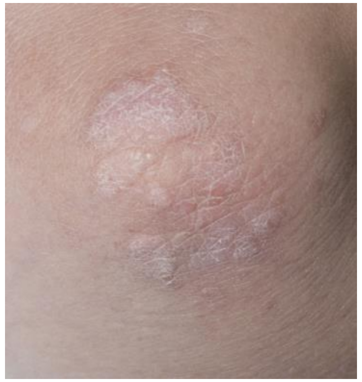

P M8

# M15 Right hand dorsum

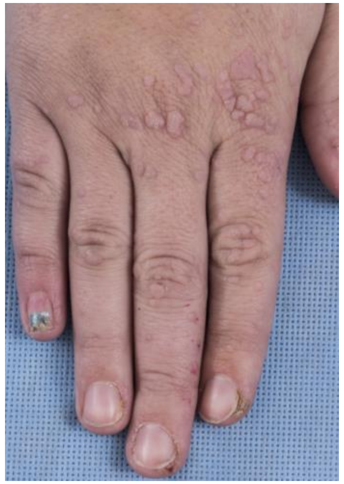
G d0

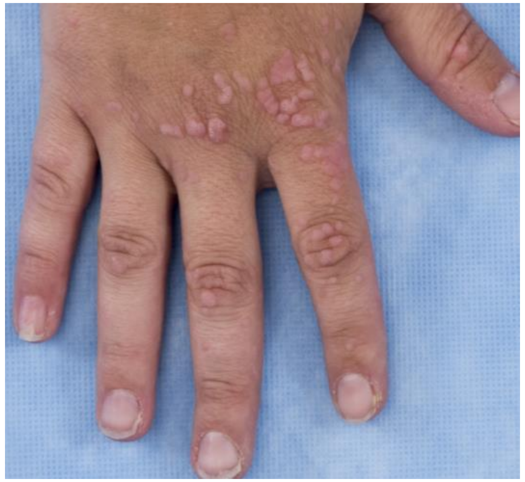
G M0

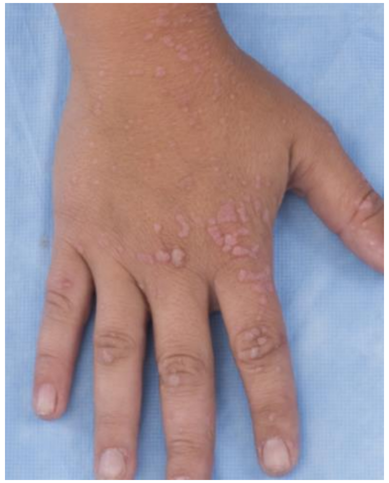
G M4

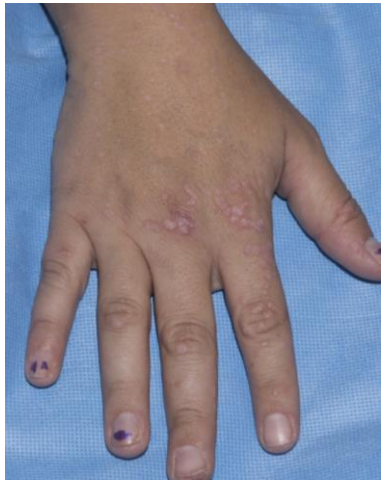
G M8

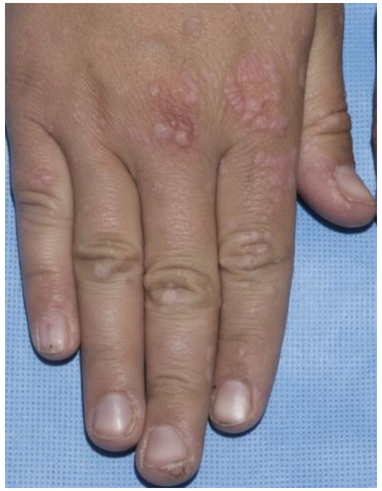
G M12

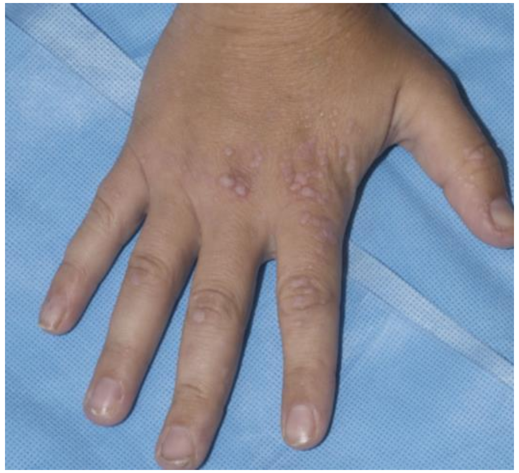
P M0

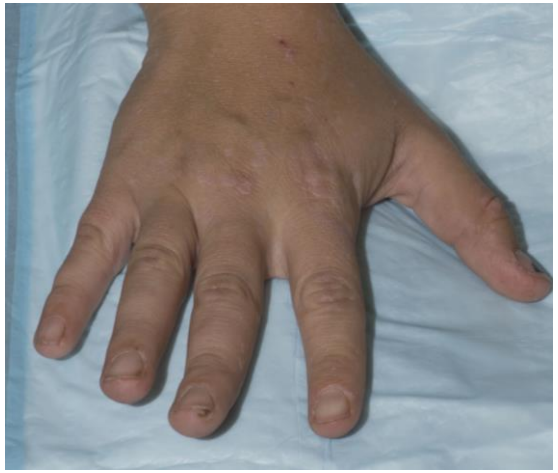
P M4

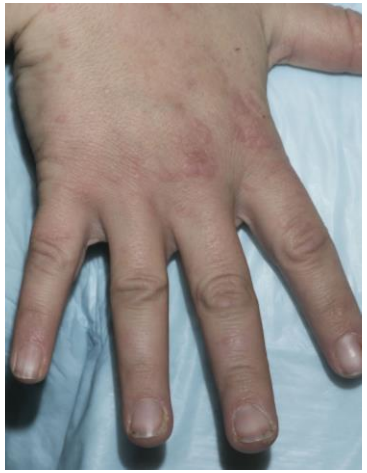
P M8

# M15 Left hand, dorsum

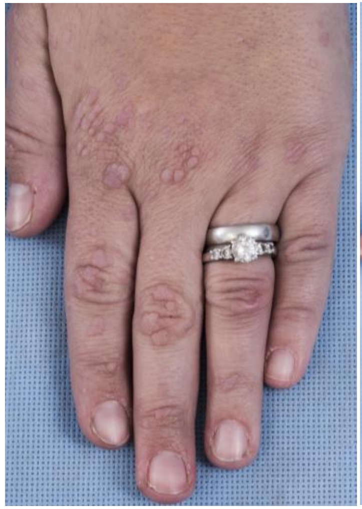

G d0

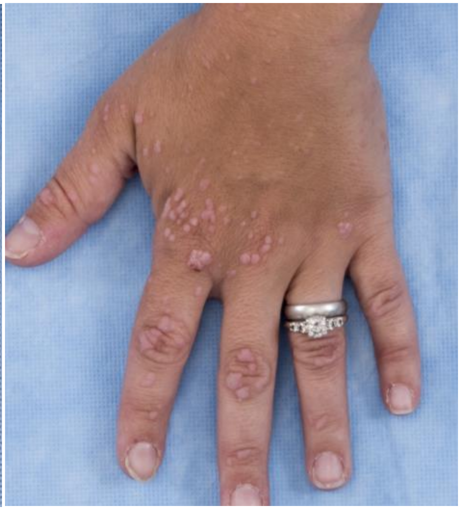

G M0

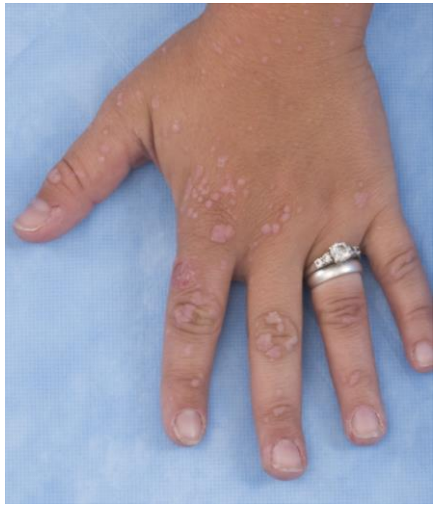

G M4

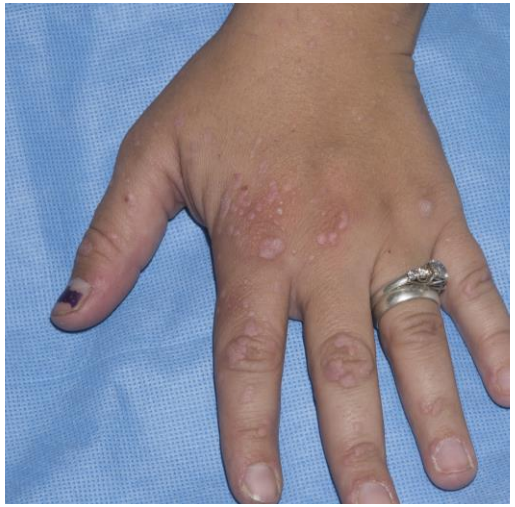

G M8

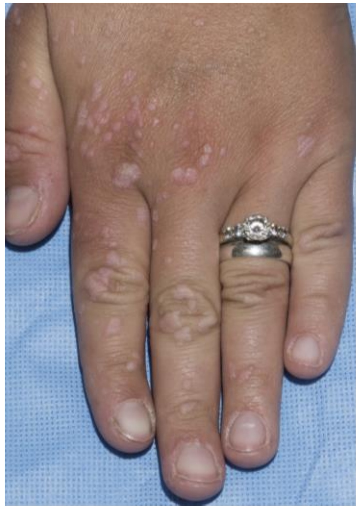

G 12

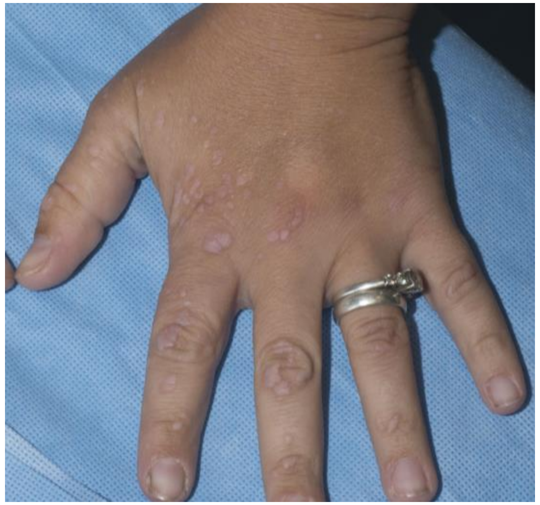

P M0

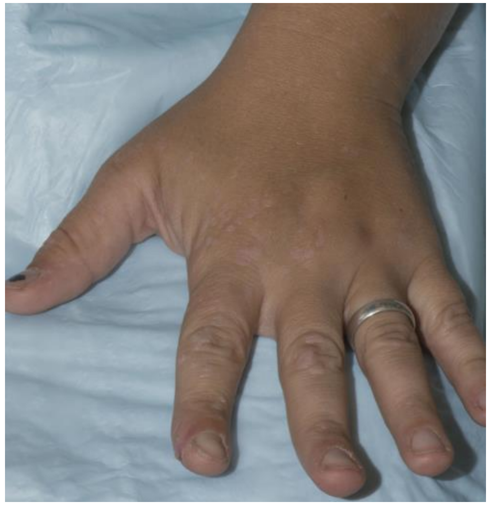

P M4

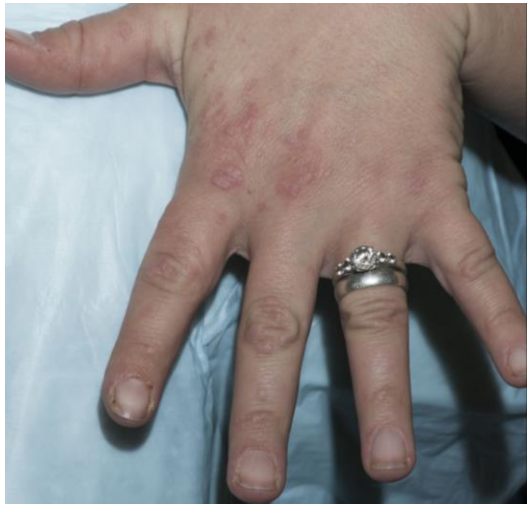

P M8

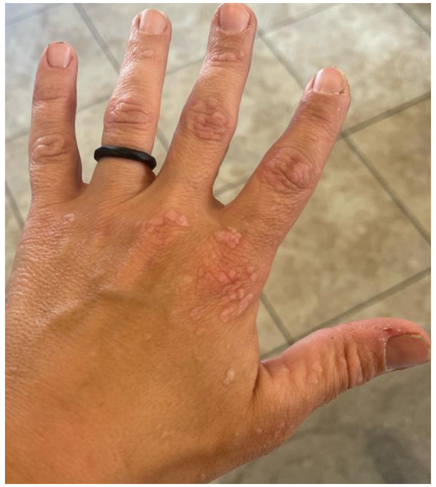

3/2022

# M15 Genitalia

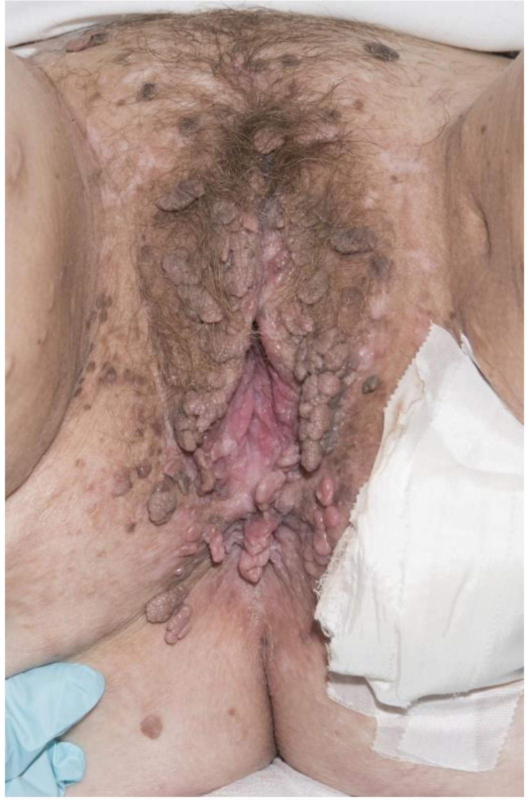

G d0

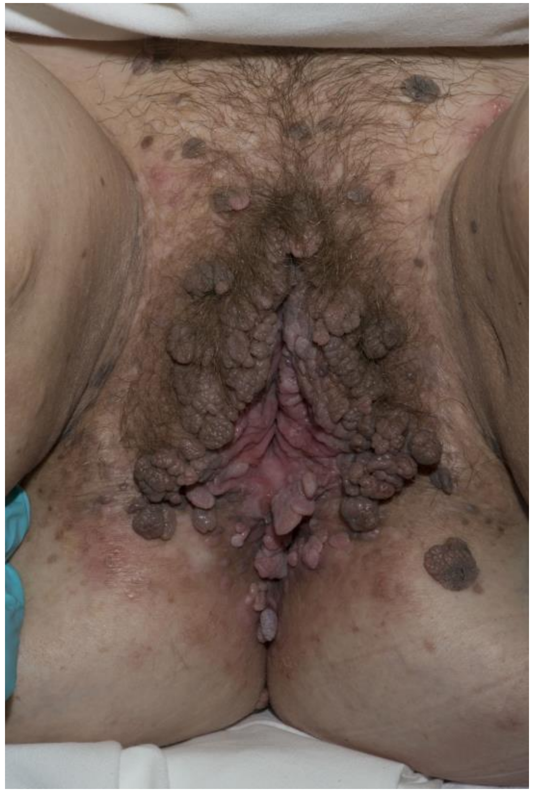

G M0

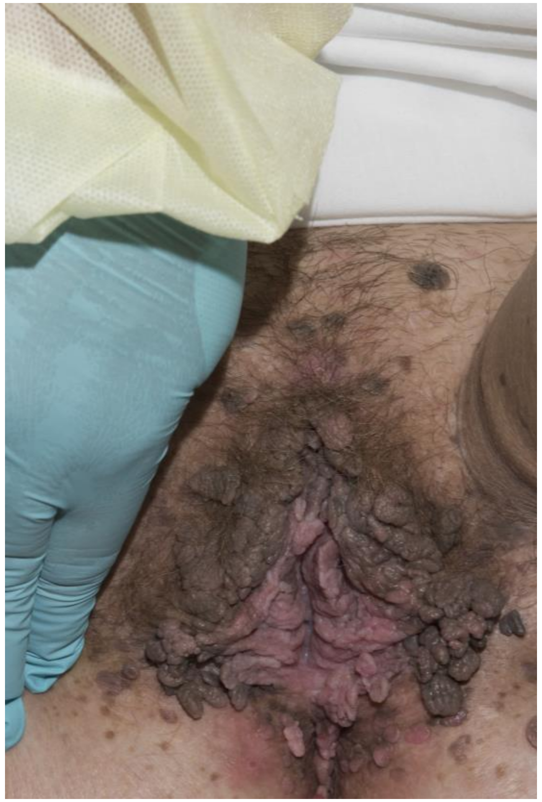

G M12

Post P na

# M17 Left second toe

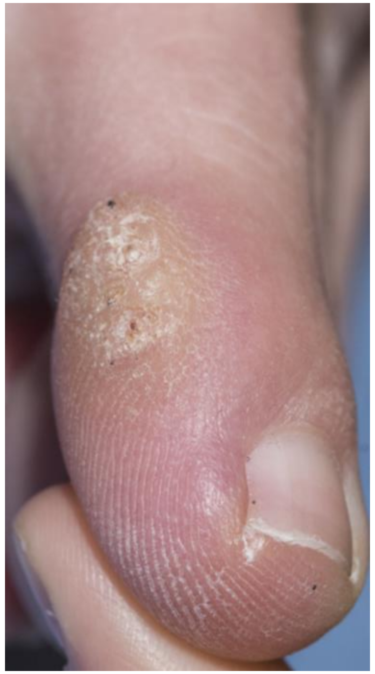

3/2016

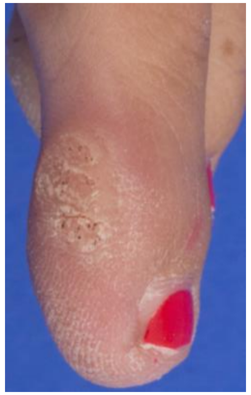

G d0
7/17

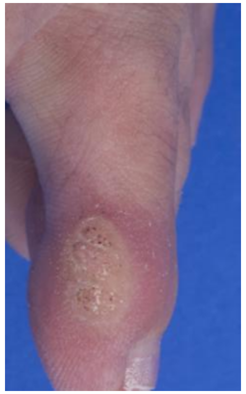

G M0

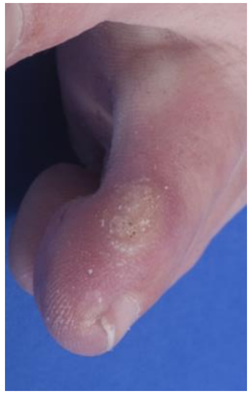

G M4

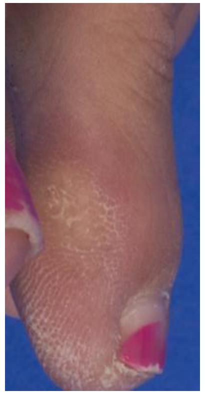

G M8

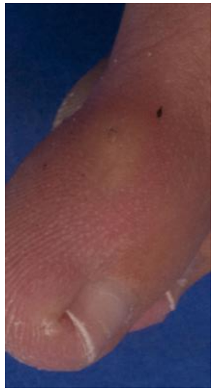

G M12

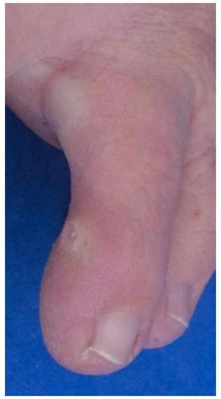

P M0

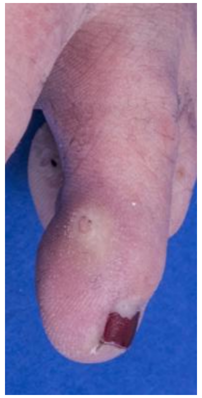

P M4

# M17 Right plantar region

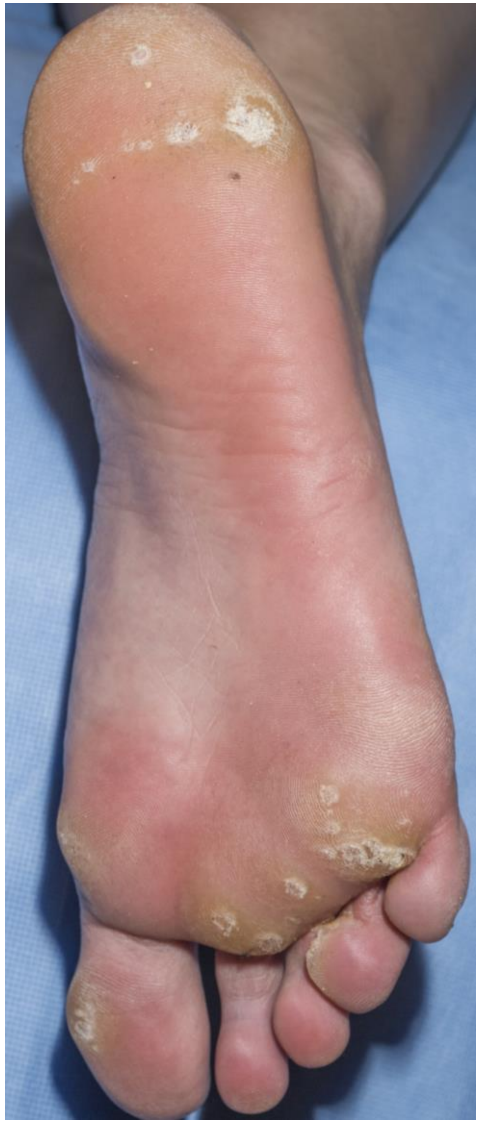

3/2016

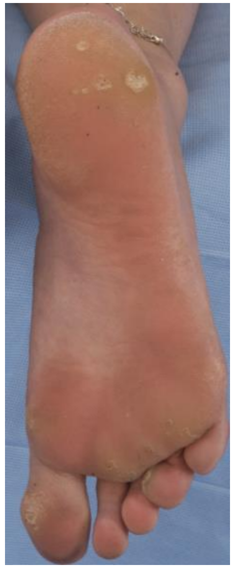

G d0

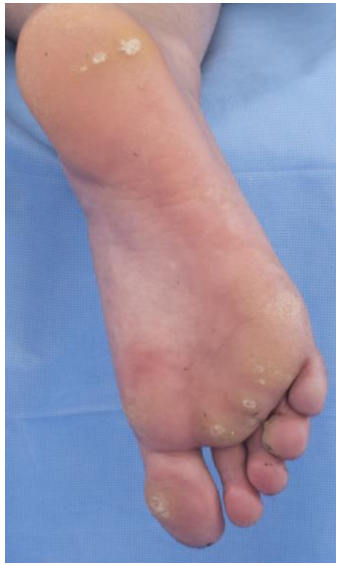

G M0

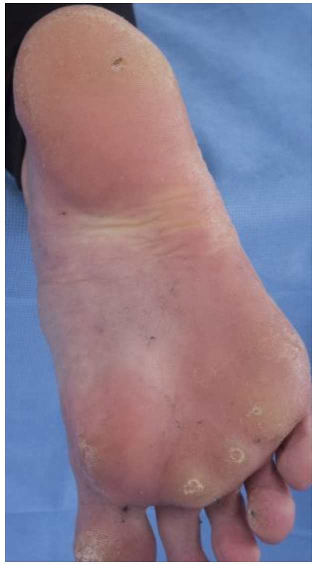

G M4

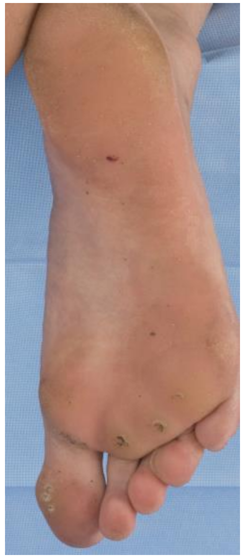

G M8

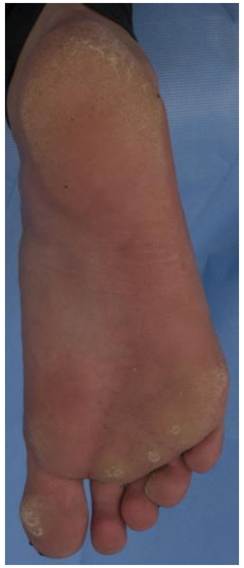

G M12

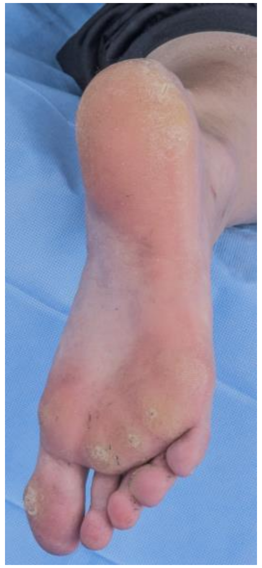

P M0

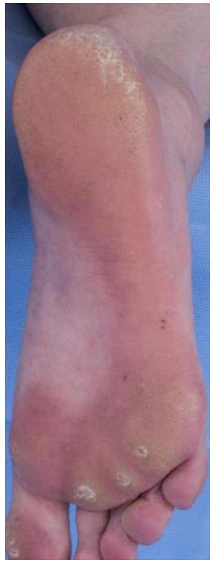

P M4

# M17 Left plantar region

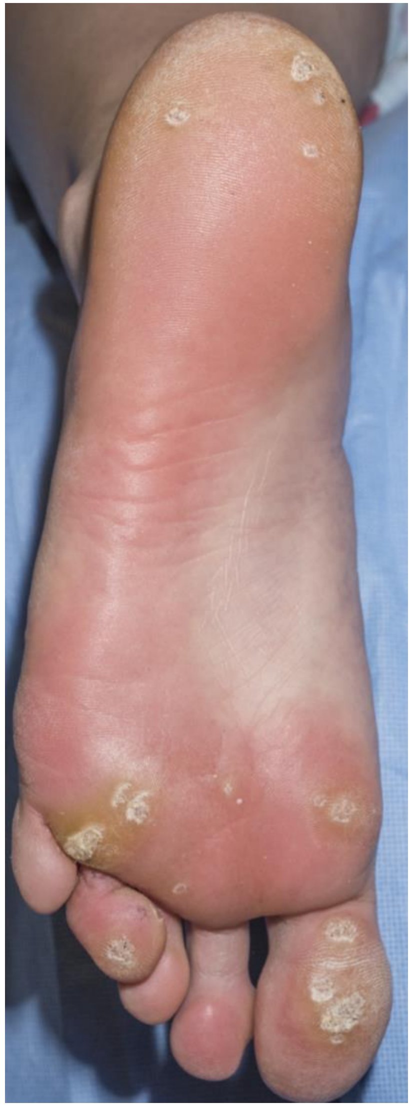

3/2016

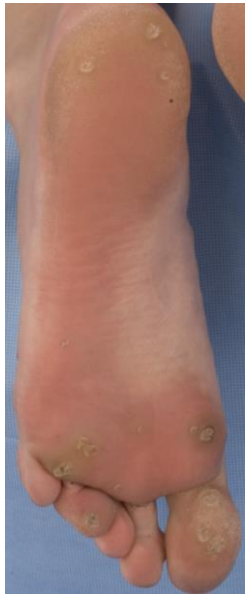

G d0

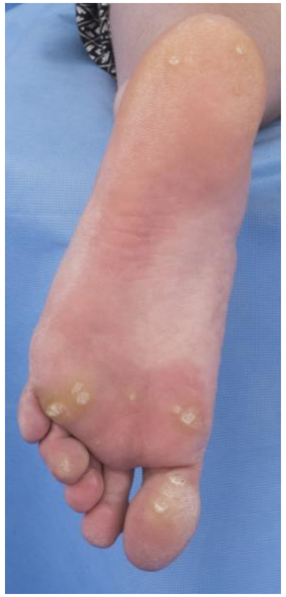

G M0

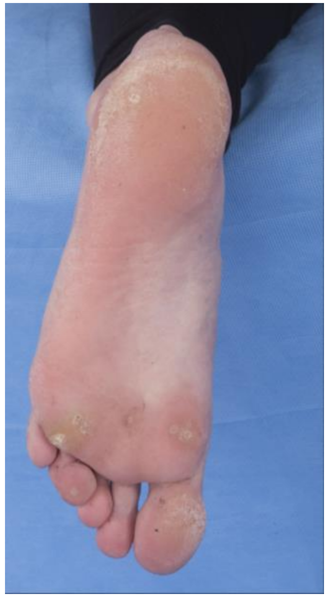

G M4

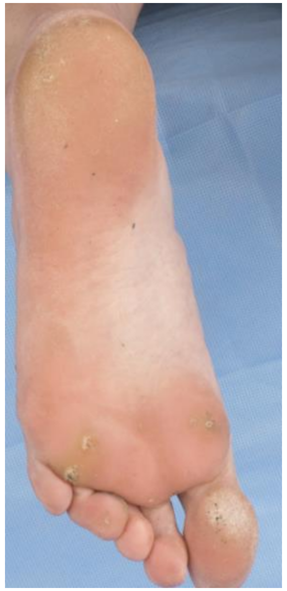

G M8

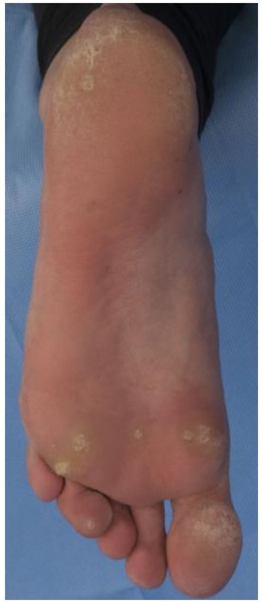

G M12

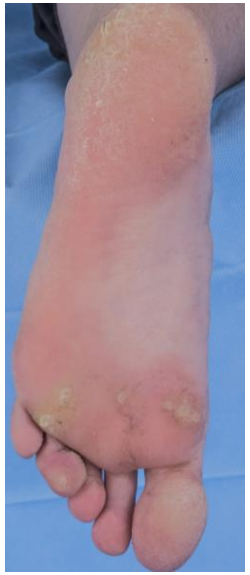

P M0

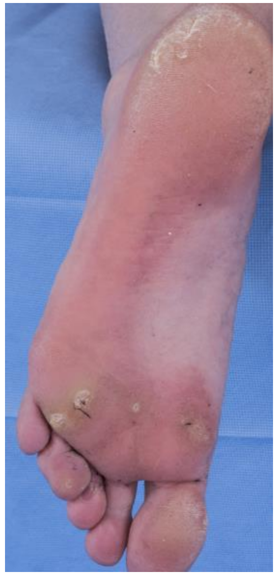

P M4

# M18 Left 2nd finger periungual area

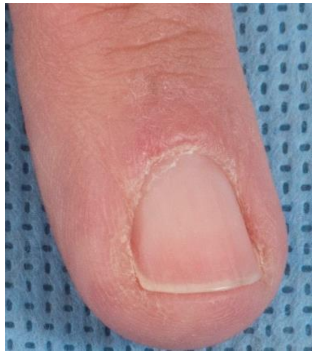

P d0

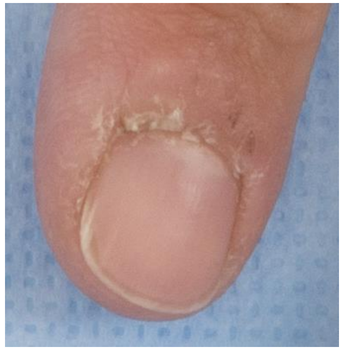

P M0

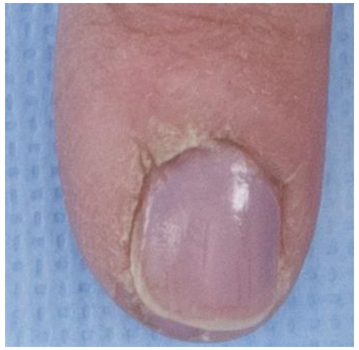

P M4

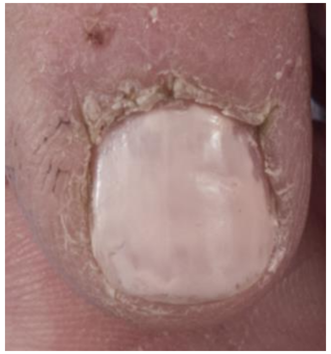

P M8

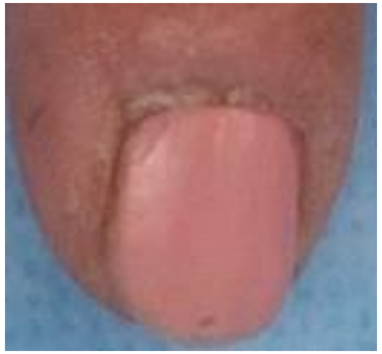

P M12

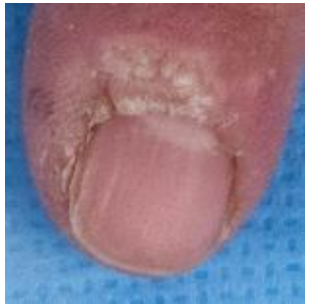

3/2020

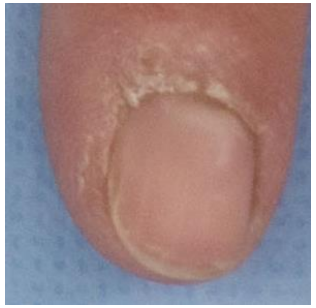

G M0

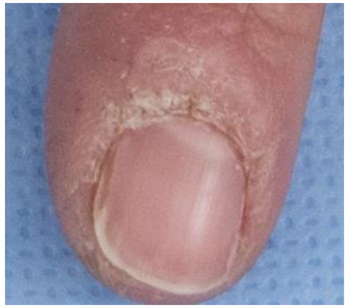

G M4

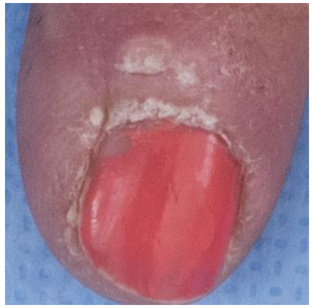

G M8

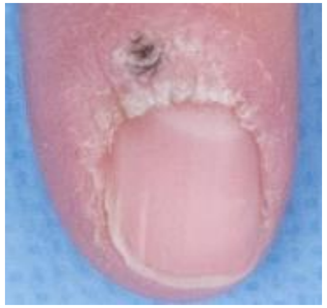

G M12

# M18 Left 4th finger proximal nail fold area

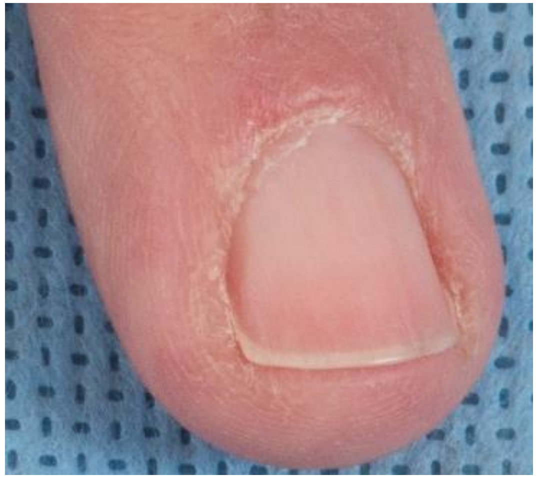

P d0

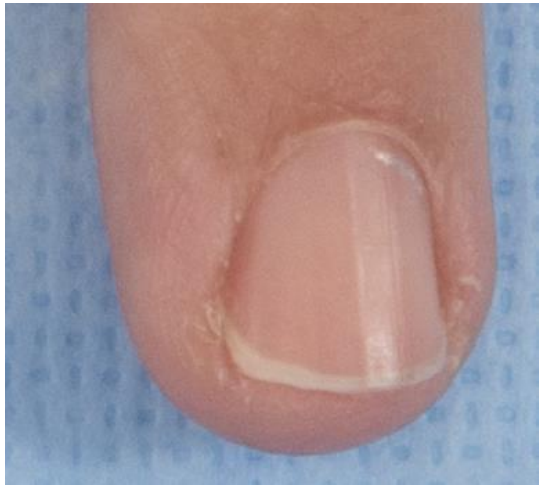

P M0

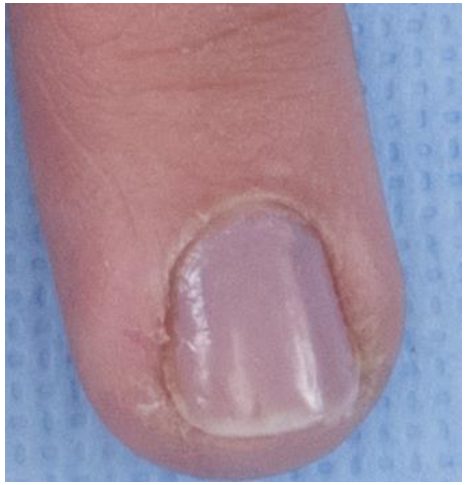

P M4

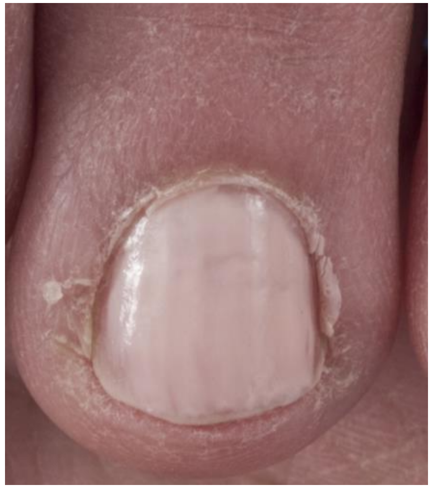

P M8

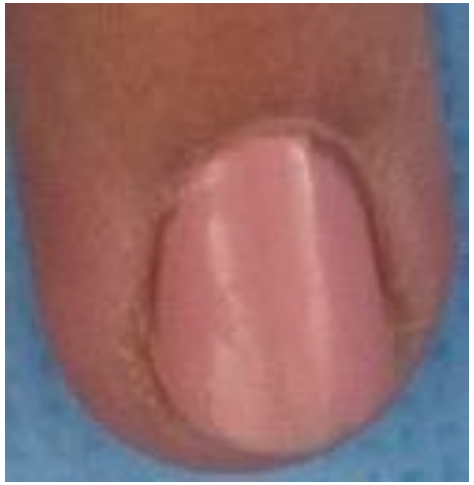

P M12

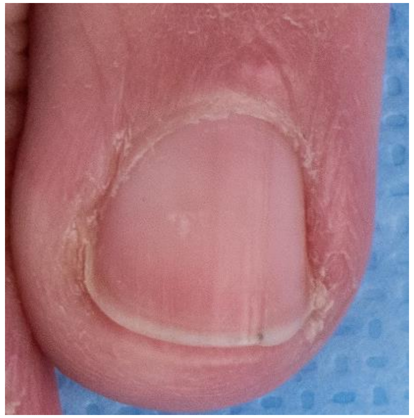

3/2020

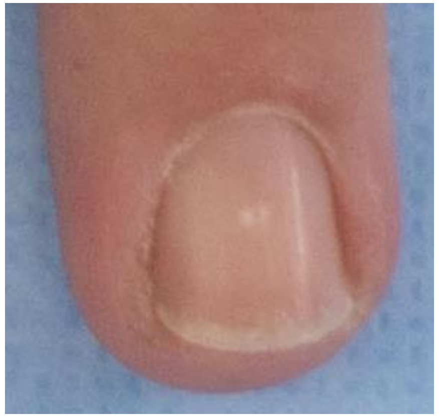

G M0

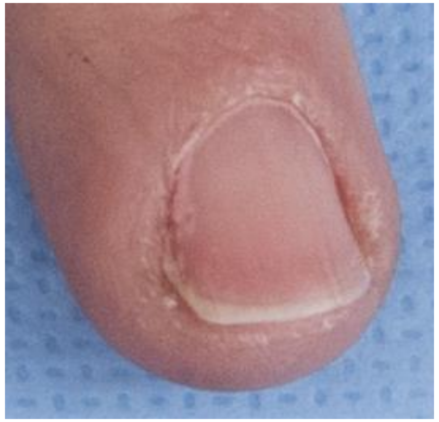

G M4

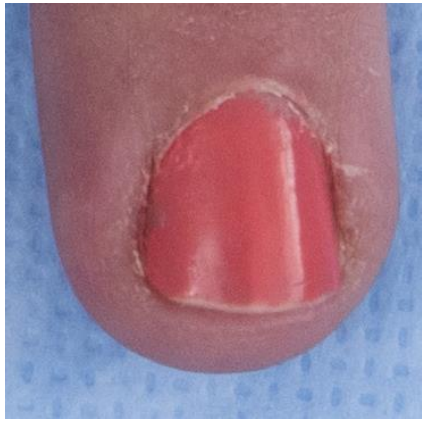

G M8

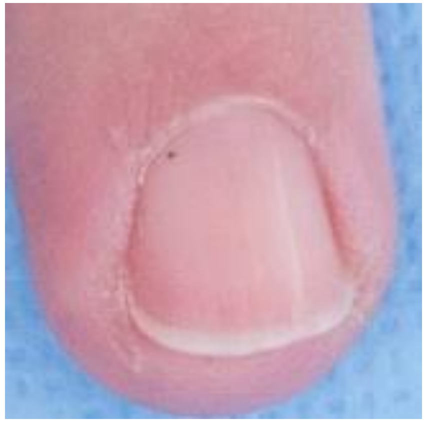

G M12

# M18 Right fingers 1-3, subungual area

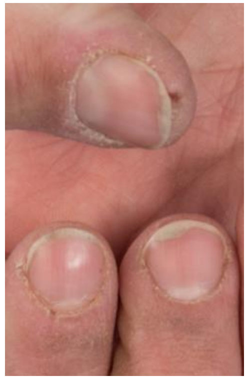
P d0

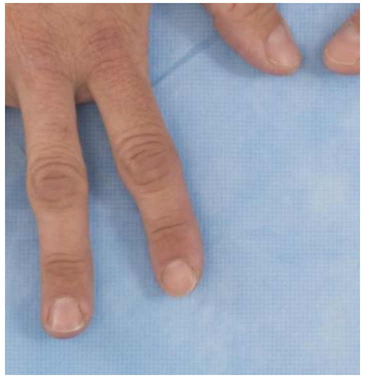
P M0

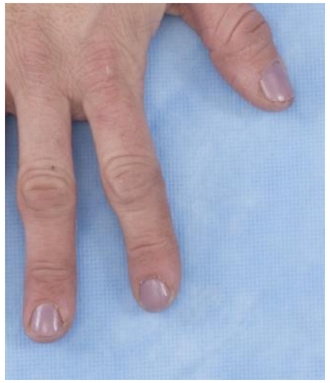
P M4

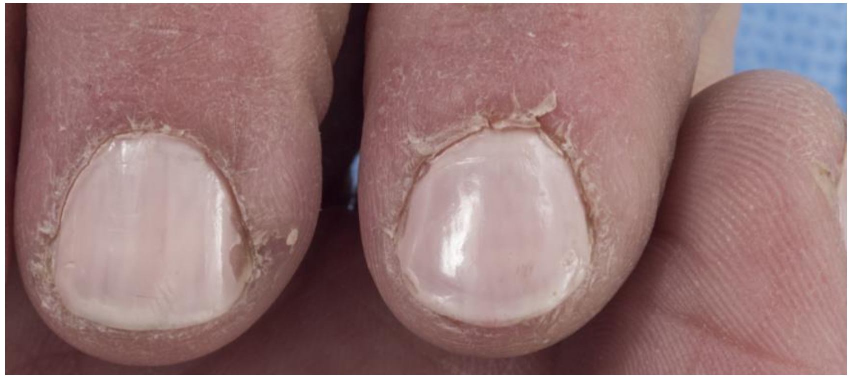
P M8

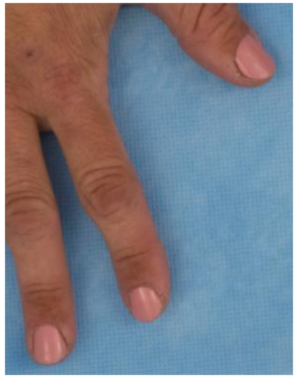
P M12

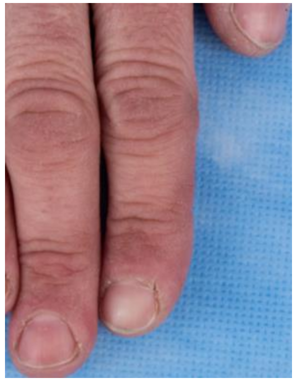
3/2020

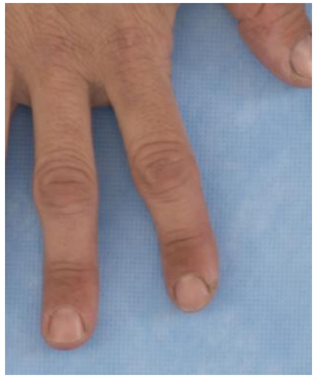
G M0

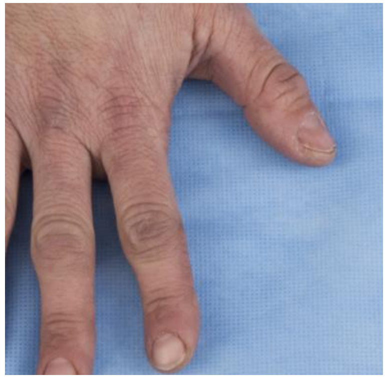
G M4

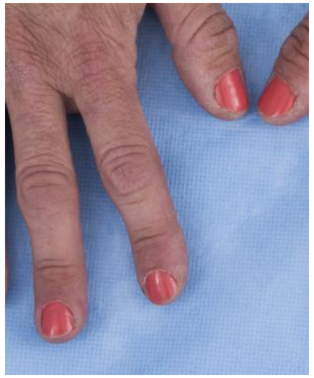
G M8

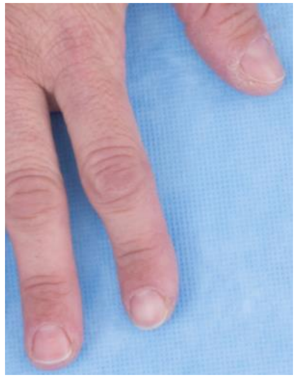
G M12
